# Systematic meta-analyses, field synopsis and global assessment of the evidence of genetic association studies in colorectal cancer

**DOI:** 10.1136/gutjnl-2019-319313

**Published:** 2019-12-09

**Authors:** Zahra Montazeri, Xue Li, Christine Nyiraneza, Xiangyu Ma, Maria Timofeeva, Victoria Svinti, Xiangrui Meng, Yazhou He, Yacong Bo, Samuel Morgan, Sergi Castellví-Bel, Clara Ruiz-Ponte, Ceres Fernández-Rozadilla, Ángel Carracedo, Antoni Castells, Timothy Bishop, Daniel Buchanan, Mark A Jenkins, Temitope O Keku, Annika Lindblom, Fränzel J B van Duijnhoven, Anna Wu, Susan M Farrington, Malcolm G Dunlop, Harry Campbell, Evropi Theodoratou, Wei Zheng, Julian Little

**Affiliations:** 1 School of Epidemiology and Public Health, Faculty of Medicine, University of Ottawa, Ottawa, Ontario, Canada; 2 Centre for Global Health, Usher Institute, The University of Edinburgh, Edinburgh, UK; 3 Department of Epidemiology, College of Preventive Medicine, Third Military Medical University, Chongqing, Chongqing, China; 4 Colon Cancer Genetics Group, Cancer Research UK Edinburgh Centre and Medical Research Council Human Genetics Unit, Medical Research Council Institute of Genetics & Molecular Medicine, The University of Edinburgh, Edinburgh, UK; 5 Jockey Club School of Public Health and Primary Care, The Chinese University of Hong Kong, Shenzhen, Hong Kong; 6 Gastroenterology Department, Institut D'Investigacions Biomèdiques August Pi i Sunyer (IDIBAPS), Hospital Clínic de Barcelona, Universitat de Barcelona, Centro de Investigación Biomédica en Red de Enfermedades Hepáticas y Digestivas (CIBEREHD), Barcelona, Spain; 7 Fundación Pública Galega de Medicina Xenómica, Grupo de Medicina Xenómica, Santiago de Compostela, Spain; Instituto de Investigación Sanitaria de Santiago (IDIS), Centro de Investigación Biomédica en Red de Enfermedades Raras (CIBERER), Santiago de Compostela, Spain; 8 Leeds Institute of Cancer and Pathology, University of Leeds, Leeds, UK; 9 Centre for Epidemiology and Biostatistics, Melbourne School of Population and Global Health, The University of Melbourne, Melbourne, Victoria, Australia; 10 Colorectal Oncogenomics Group, Genetic Epidemiology Laboratory, Department of Pathology, The University of Melbourne, Parkville, Victoria, Australia; 11 Genetic Medicine and Family Cancer Clinic, The Royal Melbourne Hospital, Parkville, Victoria, Australia; 12 Center for Gastrointestinal Biology and Disease, University of North Carolina, Chapel Hill, North Carolina, USA; 13 Department of Clinical Genetics, Karolinska University Hospital, Stockholm, Sweden; 14 Department of Molecular Medicine and Surgery, Karolinska Institutet, Stockholm, Sweden; 15 Division of Human Nutrition, Wageningen University and Research, Wageningen, The Netherlands; 16 University of Southern California, Preventative Medicine, Los Angeles, California, USA; 17 Cancer Research UK Edinburgh Centre, Medical Research Council Institute of Genetics and Molecular Medicine, The University of Edinburgh, Edinburgh, UK; 18 Division of Epidemiology, Vanderbilt University School of Medicine, Nashville, Tennessee, USA

**Keywords:** colorectal cancer

## Abstract

**Objective:**

To provide an understanding of the role of common genetic variations in colorectal cancer (CRC) risk, we report an updated field synopsis and comprehensive assessment of evidence to catalogue all genetic markers for CRC (CRCgene2).

**Design:**

We included 869 publications after parallel literature review and extracted data for 1063 polymorphisms in 303 different genes. Meta-analyses were performed for 308 single nucleotide polymorphisms (SNPs) in 158 different genes with at least three independent studies available for analysis. Scottish, Canadian and Spanish data from genome-wide association studies (GWASs) were incorporated for the meta-analyses of 132 SNPs. To assess and classify the credibility of the associations, we applied the Venice criteria and Bayesian False-Discovery Probability (BFDP). Genetic associations classified as ‘positive’ and ‘less-credible positive’ were further validated in three large GWAS consortia conducted in populations of European origin.

**Results:**

We initially identified 18 independent variants at 16 loci that were classified as ‘positive’ polymorphisms for their highly credible associations with CRC risk and 59 variants at 49 loci that were classified as ‘less-credible positive’ SNPs; 72.2% of the ‘positive’ SNPs were successfully replicated in three large GWASs and the ones that were not replicated were downgraded to ‘less-credible’ positive (reducing the ‘positive’ variants to 14 at 11 loci). For the remaining 231 variants, which were previously reported, our meta-analyses found no evidence to support their associations with CRC risk.

**Conclusion:**

The CRCgene2 database provides an updated list of genetic variants related to CRC risk by using harmonised methods to assess their credibility.

**Summary box**

**​What is already known on this subject?**

- Colorectal cancer (CRC) is a global public health challenge. A large number of genetic association studies have been conducted to assess the potential correlation between common genetic variations and CRC risk.

**​What are the new findings?**

- Using an established framework for grading credibility of genetic associations, we classified 14 independent variants at 12 loci (*MUTYH, SMAD7, TERT, CDH1, RHPN2, BMP2, TGFB1* and common variants tagging loci at 8q24, 8q23.3, 10p14, 11q23.1, 20p12.3) as highly credibly associated with CRC risk. A total of 63 variants at 52 loci were classified as ‘less-credible positive’ SNPs; variants of nine of these genes could be mostly highly prioritised for further investigation. For 231 variants previously reported to be associated with CRC, our meta-analyses found no evidence to support such associations.

**​How might it impact on clinical practice in the foreseeable future?**

- This database will be helpful for future research by promoting the investigation of these variants and corresponding genetic loci in populations other than of European origin, serving as a genetic basis for predicting risk estimates for population groups and providing candidate genes for further functional studies or gene-environment interaction studies.

## Introduction

Colorectal cancer (CRC) is one of the most commonly diagnosed malignancies and the leading cause of cancer deaths in the world, with 1.65 million new cases and about 835 000 deaths in 2015.^1^ The global burden of CRC is expected to increase by 60%, with more than 2.2 million new cases and almost 1.1 million deaths occurring annually by 2030.^2^ The distribution of CRC global burden varies widely, with more than two-thirds of all cases and about 60% of all deaths occurring in countries with a high or very high human development index, including Australia and New Zealand, Europe and North America, while incidence and mortality rates in Africa and South-Central Asia are relatively low.^1 2^ These geographic differences appear to be attributable to the differences in both environmental exposures and the background of genetically determined susceptibility.^3^

It is estimated that 15%–25% of CRC risk variance is attributed to inherited genetic factors, and the first-degree relatives of CRC patients have two to four times higher risk of developing CRC.^4 5^ The inherited genetic risk of CRC can be partly accounted for by a combination of rare high-penetrance mutations and large numbers of common genetic variants each conferring small risk.^6^ Although a number of highly penetrant mutations (eg, DNA mismatch repair genes, *APC*, *SMAD4*, *LKB1/STK11*, *MUTYH*) have been identified to influence CRC susceptibility with large effects, overall they account for only 2%–5% of incident CRC cases in the general population because these mutations are very rare.^7–9^ Candidate gene association studies have investigated the role of a large number of common genetic variants in CRC risk, but only a small number of them have been successfully replicated in subsequent studies.^10 11^

In 2012 and 2014, we reported two independently conducted series of meta-analyses to systematically evaluate associations between CRC and common variants using data from candidate gene studies and genome-wide association studies (GWASs) and identified a number of promising genetic risk variants for CRC risk.^10 11^ Our first field synopsis (published in 2012) reported 16 variants in 13 independent loci (*MUTYH, MTHFR, SMAD7, 8q24, 8q23.3, 11q23.1, 14q22.2, 1q41, 20p12.3, 20q13.33, 3q26.2, 16q22.1, 19q13.1*),^10^ and the second field synopsis (published in 2014) identified 8 additional variants in 5 independent loci (*APC, CHEK2, DNMT3B, MLH1, MUTYH*) that were strongly associated with CRC.^11^ These two synopses used slightly different methodologies, in that the 2012 field synopsis only included variants reported in four or more studies in meta-analyses, whereas the 2014 included variants with three or more studies, and there were some differences in the criteria applied for the evidence appraisal.^10 11^

In this study, we aimed to perform an updated field synopsis for CRC risk by including the most recently published genetic association studies, by following established guidelines^12 13^ and using harmonised methods for evidence appraisal.^14–16^ We systematically captured all published genetic association data on CRC for meta-analyses and subsequently incorporated data from GWAS consortia for interrogation. This study provides an up-to-date and publicly available database for CRC genetics (CRCgene2) and presents these data within a defined statistical and causal inference framework to aid interpretation of the results.^13^ We aimed to provide new insights into the fundamental biological mechanisms involved in colorectal carcinogenesis.

## Methods

### Literature search and data collection

To identify genetic association studies of CRC risk, we searched the Medline database via the Ovid gateway and the search terms comprising medical subject headings (MeSH) and keywords relating to colorectal neoplasms, the MeSH heading ‘genetic predisposition to disease’, and the keywords ‘gene$’ and ‘associate$’ were applied to terms in the entire article. The latest literature search was performed on 21 November 2018. We screened the eligibility of retrieved publications in a three-step parallel review of title, abstract and full text by following the predefined inclusion and exclusion criteria. Thus, each eligible study evaluated the association between a polymorphic genetic variant (with minor allele frequency ≥0.01 in the reference panel of the 1000 genomes) and a sporadic CRC. Studies investigating only premalignant conditions such as adenomas, polyps or dysplastic tissue were excluded. Studies investigating hereditary CRC syndromes, such as familial adenomatous polyposis, hereditary non-polyposis CRC, juvenile polyposis syndrome and Gardner’s syndrome; solely focusing on the progression or histological phenotype of CRC; or studies in animals; were excluded. Case-control, cohort and GWASs were included, while family-based studies were excluded. All included studies were published in English in a peer-reviewed journal; studies only reported as conference abstracts were excluded. Data from the eligible studies were abstracted into two standardised tables, including the key variables with regard to the study identifiers and context, study design and limitations, genotype information and outcome effects.

A list of genetic variants that were investigated in meta-analyses was summarised and data from three GWAS consortia (Scotland, Canada and Spain) were incorporated for meta-analyses, when the genotype data are available for the listed variants. In brief, the Study of CRC in Scotland (SOCCS) is a population-based case-control GWAS that includes 3417 cases and 3500 controls. The Assessment of Risk for Colorectal Tumors In Canada (ARCTIC) is a case-control GWAS database that includes 1231 cases and 1240 controls. The population-based cohort study in Spain comprised two phases (EPICOLON I and EPICOLON II) adding up to 2000 cases and 2000 controls. Restricted candidate gene genotyping data were available from both phases and GWAS data were only accessible from 881 cases and 667 controls from phase 2.^17 18^ More details about these GWAS datasets are present in online supplementary text.

10.1136/gutjnl-2019-319313.supp1 Supplementary data

### Statistical analysis

Meta-analysis was performed for genetic variants with data available from at least three independent studies. Summary crude ORs and 95% CI for allelic, recessive and dominant genetic models were calculated by applying either the fixed-effect model (Mantel-Haenszel method) or the random-effects model (DerSimonian-Laird method) in case of the existence of substantial heterogeneity. The Q statistic (with a threshold of p value <0.05) and I^2^ metric were calculated to quantify between-study heterogeneity. Funnel plot analysis with an Egger test was conducted to test for small study effects. We also estimated the statistical power of each meta-analysis based on the significance level of α=0.05, the effect sizes and the allele frequencies of genetic variants (an integral component of the Bayesian False-Discovery Probability (BFDP) analysis).^19^ All statistical analysis was conducted by using R software (R x64 3.1.0).

### Credibility of the identified genetic associations

We first applied the BFDP^19^ and the Venice criteria^12 13^ to assess the credibility of any observed genetic associations with p<0.05 in at least one genetic model. We then validated these associations in three additional GWAS consortia: Genetics and Epidemiology of Colorectal Cancer Consortium (GECCO),^20^ Colorectal Transdisciplinary Study (CORECT, https://research.fhcrc.org/peters/en/corect-study.html) and Colon Colorectal Cancer Family Registry (CFR).^21^ With meta-analysis of these three GWAS datasets, we validated the observed genetic associations using data from 58 131 CRC cases and 67 347 controls (online supplementary text), and the statistical power of validation was estimated accordingly.

The BFDP assesses the noteworthiness of an observed association. The BFDP was selected rather than the false-positive report probability (FPRP) because it uses more information, defines the noteworthiness threshold explicitly in terms of the costs of false discovery and non-discovery, and does not suffer from the inferential limitations identified for the FPRP.^22^ We calculated BFDP values at two levels of prior probability: a medium/low prior level (0.05 to 10^−3^), close to what would be expected for a candidate gene, and a very low prior level (10^−4^ to 10^−6^), close to what would be expected for a random SNP. A noteworthy threshold was defined as 0.2 based on the assumption that the cost of false discovery would be four times higher than that of false non-discovery.^19^

According to the Venice criteria, the credibility of associations is assessed for three aspects: the amount of evidence, the extent of replication and the protection from bias.^12 13^ We used statistical power to assess the volume of evidence and a grade of A, B and C was assigned, respectively, when statistical power was greater than 80%, 50%–79% or less than 50%. The extent of replication was assessed by the measurement of heterogeneity (I^2^ criterion), and a grade of A, B and C was assigned, respectively, when I^2^ was less than 25%, 25%–49% or greater than 50%.^12^ For protection from bias, a complete assessment is difficult; instead, we considered the following aspects: (1) the phenotype definition was addressed by our inclusion criteria—namely that cases would have newly incident CRC; (2) genotyping error rates are generally low; (3) the criterion of replication across studies in part addresses potential concerns about variation in genotyping quality between studies; and (4) the magnitude of effect of population stratification appears to be small in general.^23^

**Figure 1:**
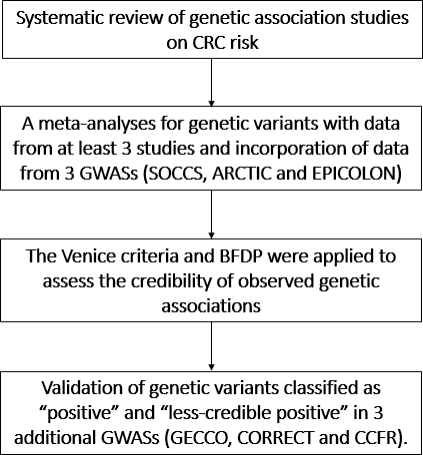
Diagram of the study design. ARCTIC, Assessment of Risk for Colorectal Tumors In Canada; BFDP, Bayesian False-Discovery Probability; CFR, Colorectal Cancer Family Registry; CORECT, Colorectal Transdisciplinary Study; CRC, colorectal cancer; EPICOLON, Gastrointestinal Oncology Group of the Spanish Gastroenterological Association; GECCO, Genetics and Epidemiology of Colorectal Cancer Consortium; GWASs, genome-wide association studies; SOCCS, Study of CRC in Scotland.

Genetic associations were then classified into four categories based on the following criteria. Associations were classified as ‘positive’ if they: (1) were statistically significant at a p value level of 0.05 in at least two of the genetic models, (2) had a BFDP less than 0.20 at least at the p value level of 0.05, (3) had a statistical power greater than 80%, (4) had an I^2^ less than 50%. A class of ‘less-credible positive’, with a less-stringent threshold, was assigned to the associations (1) that were statistically significant at a p value threshold of 0.05 in at least one of the genetic models, but (2) their BFDP was greater than 0.20 or their statistical power was between 50% and 79% or had an I^2^ greater than 50%. Associations with p value large than 0.05 were further classified as ‘null’ or ‘negative’ by assessing if there are more than 5000 cases. After credibility assessment, genetic variants classified as ‘positive’ and ‘less-credible positive’ were sent to the CORECT coordinating centre to validate their associations with CRC risk using additive, dominant and recessive models. At this stage, ‘positive’ associations that failed to be validated (at p<0.05) were downgraded to ‘less-credible positive’. A schematic diagram is shown in figure 1 to demonstrate datasets included in each phase of the analysis.

## Results

### Literature search and data collection

**Figure 2:**
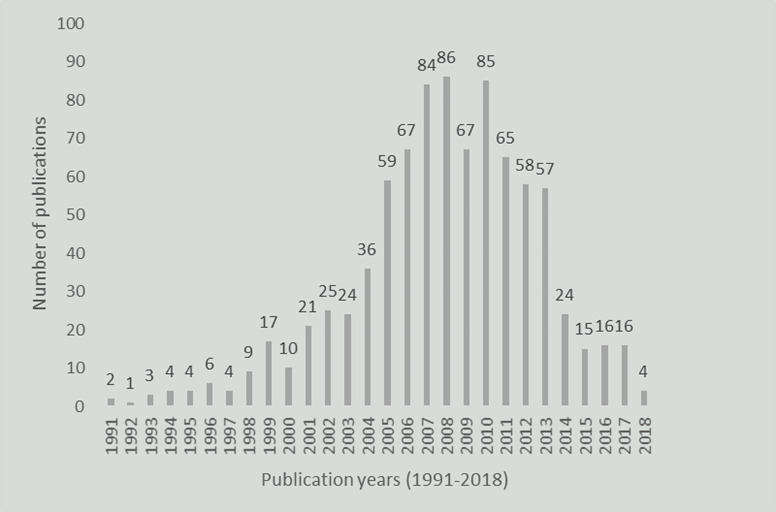
The distribution of included studies published from 1991 to 2018.

A total of 20 900 citations were identified from literature search. Of these, 6770 (32.4%) papers were published after the search period of the most recent field synopsis (31 December 2012).^11^ After eligibility screening, we finally included and extracted data from 869 publications (figure 2), reporting the association of CRC risk with 1063 polymorphisms in 303 different genes, of which 308 polymorphisms were reported in at least three independent studies.

### Meta-analyses

Meta-analyses were conducted for 308 polymorphisms in 158 different loci with data available in three or more candidate or GWA studies (online supplementary table 1). On average, these meta-analyses were based on 6149 CRC cases (median; IQR=2301–7334) and 7337 controls (median; IQR=2809–8885) originating from 8 (median; IQR=4–9) case–control studies. Data from the Scottish, Canadian and/ or Spanish GWAS were incorporated in the meta-analyses for 132 SNPs. Summary crude ORs and 95% CI for the allelic, dominant and recessive models are presented in table 1. Of the meta-analyses for 308 polymorphisms (tagged at different 158 loci), a total of 77 SNPs (25.6%) (tagged in 61 different loci) were identified to have a nominally statistically significant association (p value <0.05) with CRC risk in at least one of the three genetic models and were eligible for credibility assessment using the BFDP^19^ (online supplementary tables 2–4) and the Venice criteria^12 13^ and for validation in the three (GECCO, CORECT and CCFR) GWAS consortia (online supplementary tables 5–7).

10.1136/gutjnl-2019-319313.supp2 Supplementary data

**Table 1:** Summary of ‘credible positive associations’ from meta-analyses and credibility assessment

Gene	Variant	Cases vs controls (number of samples)	Allelic model					Power	Recessive model: var/var vs wt/wt and wt/var						Dominant model: wt/var and var/var vs wt/wt						Classification
			N	Effect size		Heterogeneity			N	Effect size		Heterogeneity		Power	N	Effect size		Heterogeneity		Power	
				OR (95% CI)	P value	I^2^ (95% CI)	P value			OR (95% CI)	P value	I^2^ (95% CI)	P value			OR (95% CI)	P value	I^2^ (95% CI)	P value		
*MUTYH*	rs36053993	28 302 vs 20 935 (19*)	19	1.42 (1.22 to 1.66)	4.89E-06	0 (0 to 33)	0.703	0.99	na	na	na	na	na	na	19	1.31 (1.12 to 1.53)	0.001	0 (0 to 24)	0.837	0.88	Positive
*MUTYH*	rs34612342	28 180 vs 20 923 (19*)	17	1.89 (1.47 to 2.42)	5.72E-07	0 (0 to 62)	0.828	1.00	7	3.40 (1.22 to 9.48)	0.019	0 (0 to 0)	0.998	1.00	17	1.80 (1.39 to 2.31)	6.42E-06	0 (0 to 52)	0.884	1.00	Positive
*SMAD7*	rs12953717	28 006 vs 26 539 (13*)	13	1.11 (1.07 to 1.16)	6.24E-07	49 (0 to 93)	0.024	1.00	13	1.17 (1.08 to 1.26)	8.34E-05	45 (0 to 94)	0.041	1.00	13	1.14 (1.07 to 1.21)	5.00E-05	54 (10 to 95)	0.020	1.00	Positive
*SMAD7*	rs4464148	17 772 vs 17 356 (9*)	9	1.12 (1.08 to 1.16)	2.66E-09	12 (0 to 92)	0.337	1.00	9	1.13 (1.00 to 1.28)	0.054	49 (0 to 98)	0.047	0.97	9	1.16 (1.11 to 1.21)	2.09E-10	2 (0 to 86)	0.416	1.00	Positive
*8q24*	rs10505477	18 562 vs 20 132 (14)	14	1.14 (1.10 to 1.19)	2.04E-11	44 (0 to 82)	0.040	1.00	14	1.16 (1.10 to 1.23)	4.06E-07	35 (0 to 74)	0.092	1.00	14	1.24 (1.17 to 1.32)	1.04E-11	36 (0 to 81)	0.084	1.00	Positive
*20p12.3*	rs961253	22 971 vs 25 270 (14*)	14	1.11 (1.06 to 1.16)	1.58E-05	60 (28 to 91)	0.002	1.00	13	1.14 (1.06 to 1.22)	7.10E-04	36 (0 to 96)	0.098	1.00	14	1.14 (1.07 to 1.21)	4.75E-05	57 (21 to 88)	0.004	1.00	Positive
*8q23.3*	rs16892766	8351 vs 8878 (6*)	6	1.24 (1.15 to 1.34)	1.67E-08	4 (0 to 92)	0.393	0.99	6	1.21 (0.88 to 1.67)	0.240	0 (0 to 85)	0.747	0.22	6	1.27 (1.17 to 1.38)	4.00E-09	3 (0 to 90)	0.400	1.00	Positive
*10p14*	rs10795668	16 763 vs 18 302 (16*)	16	0.88 (0.83 to 0.94)	4.35E-05	67 (34 to 89)	6.94E-05	1.00	16	0.83 (0.77 to 0.89)	4.77E-07	11 (0 to 70)	0.332	1.00	16	0.85 (0.79 to 0.93)	2.27E-04	68 (34 to 87)	3.06E-05	1.00	Positive
*11q23.1*	rs3802842	22 320 vs 22 965 (20*)	20	1.15 (1.11 to 1.20)	5.21E-12	43 (0 to 78)	0.024	1.00	20	1.24 (1.13 to 1.35)	3.62E-06	45 (5 to 86)	0.017	1.00	20	1.19 (1.13 to 1.25)	7.22E-12	33 (0 to 70)	0.075	1.00	Positive
*BMP2*	rs355527	13 539 vs 14 375 (9)	9	1.12 (1.08 to 1.17)	1.65E-10	0 (0 to 0)	0.977	0.99	9	1.18 (1.09 to 1.27)	3.54E-05	0 (0 to 94)	0.458	0.99	9	1.16 (1.10 to 1.21)	2.43E-09	0 (0 to 9)	0.966	1.00	Positive
*CDH1*	rs1862748	17 436 vs 18 418 (11)	11	0.91 (0.88 to 0.94)	1.98E-08	0 (0 to 63)	0.703	0.98	11	0.83 (0.77 to 0.90)	1.59E-06	2 (0 to 68)	0.427	1.00	11	0.91 (0.87 to 0.95)	6.31E-06	0 (0 to 70)	0.511	0.99	Positive
*RHPN2*	rs7259371	15 762 vs 16 700 (9*)	9	0.87 (0.82 to 0.93)	1.65E-05	52 (0 to 90)	0.034	1.00	9	0.85 (0.73 to 0.98)	0.027	17 (0 to 86)	0.295	0.71	9	0.86 (0.80 to 0.92)	3.81E-06	42 (0 to 87)	0.084	1.00	Positive
*TERT*	rs2736100	16 176 vs 18 135 (8)	8	1.07 (1.04 to 1.10)	2.95E-05	0 (0 to 82)	0.526	0.88	8	1.06 (1.00 to 1.13)	0.069	15 (0 to 86)	0.316	0.64	8	1.13 (1.08 to 1.20)	2.03E-06	0 (0 to 64)	0.788	1.00	Positive
*TGFB1*	rs1800469	4021 vs 6024 (10)	10	0.89 (0.80 to 0.99)	0.036	53(2t o 92)	0.023	1.00	10	0.84 (0.74 to 0.94)	0.003	0 (0 to 75)	0.527	1.00	10	0.84 (0.71 to 1.00)	0.051	58 (15 to 92)	0.011	1.00	Positive

*Includes GWAS data from SOCCS.

GWASs, genome-wide association studies; SOCCS, Study of CRC in Scotland.

Credibility assessment indicated 18 variants (5.8% of the meta-analysed SNPs) tagging 16 loci (rs36053993 and rs34612342 in *MUTYH,* rs2066847 in *NOD2,* rs12953717 and rs4464148 in *SMAD7,* rs1569686 in *DNMT3B,* rs2736100 in *TERT,* rs9858822 in *PPAR-gamma,* rs1862748 in *CDH1,* rs7259371 in *RHPN2,* rs355527 in *BMP2,* rs1800469 in *TGFB1*, rs10505477 in 8q24, rs16892766 in 8q23.3, rs3802842 in 11q23.1, rs961253 in 20p12.3, rs10795668 in 10p14, rs4951291 in 1q32.1) had the most credible associations with CRC risk and are therefore referred to as ‘positive’ SNPs (table 1, online supplementary tables 2–4). These findings are based on accrued data on 1224 to 43 652 cases and on 1381 to 60 883 controls, with a median of 17 100 cases per meta-analysis. The linkage disequilibrium (LD) between these ‘positive’ polymorphisms was checked pairwise using the Ensembl LD calculator with reference to the 1000 genomes: phase 3 CEU population and we found two pairs of SNPs (rs355527 and rs961253, rs12953717 and rs4464148) with r^2^ >0.20 (online supplementary tables 8). The other 59 variants (19.2% of the meta-analysed SNPs) in 49 loci with p value <0.05 were classified as ‘less-credible positive’ SNPs, given high heterogeneity, low statistical power or a high possibility of being false positive (BFDP >0.2) for their association with CRC risk (table 2, online supplementary tables 2–4). The summary findings for ‘less-credible positive’ SNPs were based on accrued data on 246 to 51 730 CRC cases and on 399 to 53 589 controls, with a median of at least 4287 CRC cases per meta-analysis.

**Table 2:** Summary of ‘less-credible associations’ from meta-analyses and credibility assessment

Gene	Variant	Cases vs controls (number of samples)	Allelic model						Recessive model: var/var vs wt/wt and wt/var						Dominant model: wt/var and var/var vs wt/wt						Classification
			N	Effect size		Heterogeneity			N	Effect size		Heterogeneity		Power	N	Effect size		Heterogeneity		Power	
				OR (95% CI)	P value	I^2^ (95% CI)	P value	Power		OR (95% CI)	P value	I^2^ (95% CI)	P value			OR (95% CI)	P value	I^2^ (95% CI)	P value		
*MTHFR*	rs1801133	43 652 vs 60 883 (94*†)	91	0.96 (0.93 to 0.99)	8.79E-03	53 (59 to 85)	3.61E-09	0.84	93	0.87 (0.81 to 0.93)	7.36E-05	56 (63 to 86)	6.79E-11	1.00	93	0.98 (0.94 to 1.02)	0.249	48 (52 to 82)	1.98E-07	0.36	Less credible
*miR*	rs895819	1322 vs 1641 (4)	4	1.18 (1.06 to 1.32)	0.003	4 (0 to 95)	0.373	0.09	4	1.50 (1.23 to 1.83)	6.32E-05	1 (0 to 96)	0.385	0.44	4	1.08 (0.93 to 1.26)	0.302	0 (0 to 94)	0.474	0.05	Less credible
*ADH1B*	rs1229984	4449 vs 6995 (9)	9	1.05 (0.94 to 1.18)	0.401	57 (8 to 91)	0.018	0.16	9	1.23 (1.02 to 1.48)	0.028	0 (0 to 70)	0.493	0.68	9	1.04 (0.90 to 1.20)	0.595	60 (14 to 92)	0.010	0.11	Less credible
*XRCC1*	rs25487	13 017 vs 18 166 (37)	37	1.11 (1.03 to 1.19)	0.008	71 (68 to 90)	8.64E-12	0.74	36	1.17 (1.02 to 1.34)	0.025	59 (39 to 80)	3.14E-06	0.91	37	1.11 (1.01 to 1.21)	0.023	63 (58 to 89)	1.44E-07	0.88	Less credible
*HFE*	rs1800562	6547 vs 34 156 (10‡)	10	1.10 (1.01 to 1.20)	0.035	0 (0 to 40)	0.850	0.46	10	1.15 (0.69 to 1.92)	0.582	24 (0 to 84)	0.240	0.12	10	1.10 (1.01 to 1.21)	0.037	0 (0 to 25)	0.937	0.68	Less credible
*MTHFR*	rs1801131	23 523 vs 35 193 (57*)	57	0.98 (0.95 to 1.01)	0.258	22 (0 to 57)	0.075	0.19	57	0.91 (0.84 to 0.98)	0.010	23 (0 to 62)	0.064	0.94	57	1.00 (0.96 to 1.04)	0.970	10 (0 to 48)	0.270	0.50	Less credible
*CYP2C9*	rs1799853	9588 vs 11 428 (12)	12	0.95 (0.90 to 1.01)	0.086	0 (0 to 73)	0.530	0.26	12	1.16 (0.96 to 1.40)	0.127	0 (0 to 73)	0.667	0.39	12	0.93 (0.87 to 0.99)	0.015	0 (0 to 72)	0.452	0.65	Less credible
*CRP*	rs1800947	2853 vs 3381 (3)	2	1.03 (0.88 to 1.20)	0.745	0 (0 to 100)	0.334	0.10	2	3.95 (1.35 to 11.56)	0.012	7 (0 to 99)	0.301	1.00	3	0.96 (0.82 to 1.12)	0.604	0 (0 to 81)	0.911	0.08	Less credible
*CRP*	rs1205	3037 vs 9333 (4)	4	1.07 (1.00 to 1.15)	0.065	0 (0 to 95)	0.479	0.34	4	1.18 (1.02 to 1.37)	0.027	0 (0 to 94)	0.431	0.73	4	1.05 (0.96 to 1.16)	0.288	0 (0 to 90)	0.669	0.21	Less credible
*EGF*	rs4444903	899 vs 976 (5)	5	0.83 (0.64 to 1.07)	0.143	70 (18 to 97)	0.010	0.52	5	0.91 (0.63 to 1.31)	0.601	50 (0 to 92)	0.093	0.13	5	0.65 (0.44 to 0.96)	0.030	67 (12 to 97)	0.016	0.99	Less credible
*ESR2*	rs928554	2574 vs 2977 (3)	3	1.09 (1.01 to 1.18)	0.025	0 (0 to 89)	0.743	0.35	3	1.12 (0.97 to 1.29)	0.118	0 (0 to 93)	0.590	0.36	3	1.13 (1.01 to 1.26)	0.041	0 (0 to 89)	0.876	0.57	Less credible
*HPGD*	rs8752	3968 vs 4830 (3‡)	3	1.05 (0.92 to 1.21)	0.467	80 (23 to 99)	0.007	0.20	3	1.12 (1.00 to 1.26)	0.047	6 (0 to 97)	0.345	0.53	3	1.03 (0.83 to 1.29)	0.786	83 (34 to 99)	0.003	0.10	Less credible
*LIPC*	rs6083	7667 vs 7980 (4‡)	4	0.96 (0.90 to 1.02)	0.172	41 (0 to 95)	0.167	0.23	4	0.89 (0.80 to 0.99)	0.033	22 (0 to 94)	0.278	0.68	4	0.98 (0.91 to 1.05)	0.519	11 (0 to 92)	0.340	0.10	Less credible
*miR*	rs2292832	2355 vs 2571 (5‡)	5	0.94 (0.86 to 1.02)	0.152	0 (0 to 82)	0.650	0.17	5	1.06 (0.89 to 1.27)	0.522	0 (0 to 73)	0.789	0.10	5	0.87 (0.78 to 0.98)	0.021	0 (0 to 88)	0.704	0.68	Less credible
*MSH2*	rs4608577	4308 vs 4011 (3‡)	3	0.94 (0.86 to 1.02)	0.112	0 (0 to 91)	0.795	0.19	3	0.72 (0.56 to 0.92)	0.008	0 (0 to 95)	0.648	0.68	3	0.96 (0.88 to 1.05)	0.406	0 (0 to 62)	0.945	0.14	Less credible
*MSH3*	rs184967	8151 vs 10 103 (4‡)	4	1.08 (1.00 to 1.16)	0.049	35 (0 to 98)	0.204	0.47	4	1.23 (1.02 to 1.49)	0.031	0 (0 to 91)	0.702	0.59	4	1.07 (0.99 to 1.16)	0.098	28 (0 to 98)	0.242	0.54	Less credible
*MSH3*	rs26779	6050 vs 8024 (6‡)	6	1.07 (1.02 to 1.14)	0.013	11 (0 to 92)	0.343	0.46	6	1.13 (0.98 to 1.31)	0.085	36 (0 to 93)	0.166	0.59	6	1.08 (1.01 to 1.15)	0.030	0 (0 to 85)	0.682	0.62	Less credible
*MTHFD1*	rs1950902	9059 vs 11 358 (7‡)	7	0.94 (0.89 to 0.99)	0.026	3 (0 to 82)	0.400	0.39	7	1.01 (0.87 to 1.18)	0.878	0 (0 to 91)	0.767	0.05	7	0.92 (0.86 to 0.98)	0.015	15 (0 to 90)	0.313	0.79	Less credible
*PARP1*	rs1136410	7002 vs 8328 (7‡)	7	1.05 (0.95 to 1.17)	0.300	52 (0 to 98)	0.052	0.24	7	1.23 (1.05 to 1.44)	0.012	0 (0 to 92)	0.475	0.78	7	1.03 (0.93 to 1.15)	0.529	36 (0 to 94)	0.153	0.14	Less credible
*PGR*	rs1042838	5232 vs 5733 (4‡)	4	1.08 (1.01 to 1.16)	0.034	0 (0 to 95)	0.507	0.32	4	0.97 (0.77 to 1.23)	0.819	0 (0 to 85)	0.880	0.06	4	1.11 (1.02 to 1.21)	0.012	0 (0 to 96)	0.450	0.71	Less credible
	rs4951039	13 791 vs 14 288 (7‡)	7	0.91 (0.81 to 1.02)	0.106	81 (49 to 95)	1.48E-05	0.79	7	0.78 (0.66 to 0.93)	0.005	0 (0 to 68)	0.620	0.82	7	0.91 (0.80 to 1.04)	0.163	82 (51 to 96)	9.30E-06	0.94	Less credible
*SCD*	rs7849	2011 vs 2580 (3)	3	0.85 (0.73 to 0.98)	0.025	29 (0 to 98)	0.247	0.53	3	0.64 (0.40 to 1.02)	0.058	41 (0 to 99)	0.185	0.68	3	0.85 (0.71 to 1.02)	0.084	33 (0 to 99)	0.225	0.72	Less credible
*XPC*	rs2228000	2677 vs 4253 (6)	6	0.99 (0.71 to 1.36)	0.929	94 (82 to 99)	1.35E-16	0.05	6	0.95 (0.49 to 1.81)	0.866	91 (71 to 99)	2.18E-10	0.09	6	0.58 (0.41 to 0.82)	0.002	92 (83 to 99)	1.49E-11	1.00	Less credible
*VEGF*	rs699947	4497 vs 5334 (10‡)	10	1.09 (1.00 to 1.19)	0.056	46 (0 to 91)	0.053	0.56	10	1.14 (1.01 to 1.29)	0.029	14 (0 to 89)	0.317	0.74	10	1.09 (0.95 to 1.24)	0.219	47 (0 to 87)	0.047	0.50	Less credible
*IGF1*	rs35767	2792 vs 4988 (3)	3	0.95 (0.80 to 1.14)	0.599	59 (0 to 99)	0.087	0.15	3	0.75 (0.62 to 0.91)	0.003	0 (0 to 97)	0.567	0.79	3	0.98 (0.79 to 1.21)	0.826	60 (0 to 99)	0.081	0.07	Less credible
*NAT2*	rs1799929	1861 vs 1952 (5*)	5	0.84 (0.74 to 0.95)	0.004	20 (0 to 94)	0.289	0.53	5	0.68 (0.47 to 0.99)	0.044	68 (14 to 98)	0.015	0.81	5	0.85 (0.74 to 0.97)	0.016	0 (0 to 85)	0.872	0.61	Less credible
*NAT2*	rs1799930	6446 vs 7193 (8*)	8	1.07 (0.99 to 1.16)	0.099	44 (0 to 96)	0.082	0.25	8	0.99 (0.88 to 1.12)	0.897	0 (0 to 64)	0.812	0.08	8	1.13 (1.00 to 1.27)	0.043	51 (0 to 97)	0.045	0.70	Less credible
*ABCB1* (*MDR1*)	rs9282564	9536 vs 9145 (6‡)	6	1.07 (1.00 to 1.14)	0.053	0 (0 to 77)	0.561	0.29	6	0.88 (0.65 to 1.19)	0.424	18 (0 to 86)	0.299	0.14	6	1.09 (1.01 to 1.17)	0.018	0 (0 to 70)	0.729	0.65	Less credible
*ABCB1* (*MDR1*)	rs1045642	7537 vs 8396 (16)	16	0.94 (0.88 to 1.01)	0.108	46 (1 to 88)	0.024	0.50	16	0.89 (0.81 to 0.97)	0.012	18 (0 to 78)	0.248	0.87	16	0.97 (0.85 to 1.10)	0.608	57 (32 to 91)	0.003	0.14	Less credible
*ABCB1* (*MDR1*)	rs1202168	7000 vs 6485 (5)	5	1.06 (0.99 to 1.14)	0.071	45 (0 to 93)	0.124	0.39	5	1.04 (0.95 to 1.14)	0.350	0 (0 to 81)	0.699	0.14	5	1.12 (1.00 to 1.24)	0.042	52 (0 to 93)	0.080	0.88	Less credible
*ADIPOR1*	rs1342387	2472 vs 2848 (5‡)	5	0.87 (0.78 to 0.98)	0.018	48 (0 to 95)	0.105	0.71	5	0.84 (0.73 to 0.96)	0.012	0 (0 to 62)	0.900	0.68	5	0.85 (0.69 to 1.03)	0.098	63 (0 to 96)	0.029	0.79	Less credible
*AXIN2*	rs2240308	4723 vs 4932 (4‡)	4	1.06 (1.00 to 1.12)	0.044	0 (0 to 92)	0.397	0.30	4	1.07 (0.96 to 1.19)	0.219	17 (0 to 95)	0.306	0.29	4	1.10 (1.01 to 1.21)	0.037	0 (0 to 76)	0.758	0.54	Less credible
*MMP1*	rs1799750	1660 vs 2024 (10)	9	0.78 (0.66 to 0.93)	0.005	59 (8 to 88)	0.012	0.95	9	0.78 (0.61 to 0.99)	0.041	21 (0 to 68)	0.254	0.75	10	0.70 (0.56 to 0.88)	0.002	57 (9 to 88)	0.013	1.00	Less credible
*CYP1A1*	rs1048943	9661 vs 11 774 (19*)	19	1.28 (1.07 to 1.52)	0.006	84 (83 to 96)	1.15E-15	1.00	18	1.26 (1.04 to 1.52)	0.016	0 (0 to 55)	0.456	0.72	19	1.36 (1.08 to 1.72)	0.009	88 (93 to 98)	2.16E-23	1.00	Less credible
*GSTT1*	Null variant	19 133 vs 27 821 (56)	7	0.91 (0.70 to 1.20)	0.508	71 (21 to 99)	0.015	0.49	53	1.17 (1.07 to 1.28)	0.001	70 (71 to 90)	1.62E-15	1.00	6	1.00 (0.73 to 1.37)	0.979	53 (0 to 98)	0.059	0.05	Less credible
*VDR*	rs1544410	14 789 vs 15 922 (16‡)	16	0.83 (0.72 to 0.96)	0.011	93 (91 to 95)	5.59E-40	1.00	16	0.81 (0.67 to 0.98)	0.033	85 (77 to 89)	1.08E-14	1.00	16	0.82 (0.69 to 0.96)	0.015	88 (83 to 91)	2.62E-20	1.00	Less credible
*8q24*	rs6983267	51 730 vs 53 589 (34‡)	34	1.11 (1.06 to 1.17)	1.75E-05	85 (77 to 93)	1.93E-28	1.00	34	1.16 (1.08 to 1.25)	4.92E-05	79 (66 to 89)	2.80E-18	1.00	34	1.16 (1.08 to 1.24)	2.98E-05	80 (69 to 92)	8.68E-19	1.00	Less credible
*RAD18*	rs373572	6560 vs 6906 (5‡)	5	1.15 (1.01 to 1.30)	0.031	75 (33 to 98)	0.003	0.96	5	1.23 (1.01 to 1.49)	0.041	54 (0 to 95)	0.067	0.93	5	1.15 (0.98 to 1.36)	0.080	73 (21 to 98)	0.005	0.98	Less credible
*NQ01*	rs1800566	11 183 vs 12 525 (16‡)	16	1.17 (1.05 to 1.30)	0.006	80 (66 to 94)	1.17E-09	1.00	16	1.21 (0.99 to 1.48)	0.060	55 (17 to 87)	0.004	0.91	16	1.20 (1.06 to 1.37)	0.006	78 (64 to 94)	8.77E-09	1.00	Less credible
*PTGS2*/*COX2*	rs20417	7785 vs 11 371 (18)	17	1.10 (1.00 to 1.2)	0.035	37 (0 to 83)	0.062	0.57	17	1.24 (1.04 to 1.47)	0.016	0 (0 to 46)	0.928	0.64	18	1.12 (1.00 to 1.26)	0.050	51 (14 to 87)	0.019	0.90	Less credible
*NOD2*	rs2066844	3297 vs 3088 (9)	9	1.38 (1.04 to 1.84)	0.026	39 (0 to 91)	0.109	0.66	9	1.97 (0.55 to 7.04)	0.299	0 (0 to 83)	0.689	0.17	9	1.35 (1.02 to 1.78)	0.033	33 (0 to 90)	0.155	0.86	Less credible
*CCND1*	rs17852153	6500 vs 8885 (20)	20	1.10 (1.02 to 1.19)	0.019	58 (45 to 92)	0.001	0.83	20	1.11 (0.97 to 1.28)	0.128	63 (54 to 94)	7.69E-05	0.82	20	1.15 (1.05 to 1.26)	0.003	18 (0 to 76)	0.226	0.94	Less credible
*GSTM1*	Null variant	28 240 vs 38 880 (74)	13	1.12 (0.89 to 1.40)	0.335	70 (22 to 98)	0.005	1.00	6	1.18 (0.90 to 1.55)	0.234	67 (0 to 98)	0.028	1.00	74	1.10 (1.05 to 1.16)	0.001	47 (34 to 75)	6.63E-06	1.00	Less credible
*9p24*	rs719725	13 513 vs 14 999 (14)	14	1.07 (1.02 to 1.11)	0.002	24 (0 to 79)	0.190	0.79	14	1.09 (1.02 to 1.15)	0.006	29 (0 to 75)	0.148	0.94	14	1.09 (1.02 to 1.17)	0.008	0 (0 to 66)	0.521	0.72	Less credible
*ERCC5*	rs17655	9653 vs 11 367 (14)	13	1.07 (1.02 to 1.12)	0.003	0 (0 to 73)	0.626	0.60	13	0.98 (0.89 to 1.08)	0.688	0 (0 to 77)	0.538	0.07	14	1.14 (1.04 to 1.24)	0.004	44 (0 to 85)	0.038	1.00	Less credible
*GH1*	rs2665802	3275 vs 3848 (7)	7	0.89 (0.80 to 0.99)	0.025	49 (0 to 97)	0.069	0.68	7	0.83 (0.71 to 0.98)	0.028	37 (0 to 95)	0.144	0.87	7	0.89 (0.78 to 1.02)	0.103	36 (0 to 96)	0.157	0.62	Less credible
*TP73*	G4C14	858 vs 1173 (4)	4	1.21 (1.04 to 1.40)	0.013	4 (0 to 92)	0.371	0.46	4	1.75 (1.23 to 2.48)	0.002	0 (0 to 94)	0.513	0.91	4	1.10 (0.83 to 1.45)	0.501	55 (0 to 96)	0.083	0.18	Less credible
*TGFB1*	rs4803455	3747 vs 4513 (3‡)	3	1.11 (1.04 to 1.18)	0.001	0 (0 to 71)	0.903	0.66	3	1.12 (1.01 to 1.24)	0.027	0 (0 to 80)	0.845	0.59	3	1.18 (1.06 to 1.30)	0.001	0 (0 to 85)	0.879	0.90	Less credible
*APC*	rs1801155	2389 vs 4223 (5)	5	1.60 (1.21 to 2.11)	0.001	27 (0 to 94)	0.244	0.85	na	na	na	na	na	na	5	1.62 (1.21 to 2.16)	0.001	28 (0 to 94)	0.235	0.99	Less credible
*MLH1*	rs63750447	937 vs 812 (4)	3	2.14 (1.12 to 4.10)	0.022	39 (0 to 99)	0.194	0.75	na	na	na	na	na	na	3	2.17 (1.14 to 4.14)	0.019	36 (0 to 98)	0.208	0.96	Less credible
*VDR*	rs11568820	4001 vs 4682 (5*)	4	1.14 (1.05 to 1.23)	0.001	0 (0 to 92)	0.648	0.71	4	1.37 (1.02 to 1.85)	0.039	35 (0 to 97)	0.203	0.90	5	1.14 (1.04 to 1.24)	0.004	0 (0 to 0)	0.989	0.85	Less credible
*ABCB1* (*MDR1*)	rs1128503	246 vs 399 (3)	3	0.70 (0.51 to 0.96)	0.027	45 (0 to 98)	0.164	0.59	3	0.77 (0.37 to 1.59)	0.483	71 (0 to 99)	0.031	0.31	3	0.52 (0.35 to 0.76)	0.001	0 (0 to 96)	0.617	0.93	Less credible
*ADIPOQ*	rs2241766	1517 vs 1909 (5)	5	1.17 (1.01 to 1.35)	0.033	29 (0 to 97)	0.231	0.50	5	1.04 (0.74 to 1.45)	0.830	14 (0 to 98)	0.325	0.06	5	1.26 (1.09 to 1.47)	0.002	8 (0 to 96)	0.364	0.92	Less credible
*CHEK2*	rs1787996	1687 vs 3370 (3)	3	1.47 (1.12 to 1.92)	0.006	0 (0 to 98)	0.499	0.53	na	na	na	na	na	na	3	1.48 (1.13 to 1.95)	0.005	0 (0 to 98)	0.496	0.81	Less credible
*CHEK2*	1100delC	2417 vs 3615 (4)	4	1.66 (1.01 to 2.73)	0.048	0 (0 to 83)	0.835	0.60	na	na	na	na	na	na	4	1.66 (1.01 to 2.75)	0.047	0 (0 to 83)	0.835	0.86	Less credible
*TP53*	rs1042522	10591 vs 12 673 (30)	30	1.11 (0.99 to 1.24)	0.077	85 (84 to 95)	4.42E-26	0.73	30	1.21 (1.02 to 1.44)	0.026	70 (55 to 88)	2.77E-09	0.98	30	1.11 (0.96 to 1.28)	0.163	82 (84 to 05)	7.58E-20	0.64	Less credible
*TP53*	rs17878362	1812 vs 2319 (6)	6	0.98 (0.76 to 1.26)	0.859	70 (23 to 96)	0.005	0.06	6	1.28 (0.81 to 2.03)	0.282	0 (0 to 79)	0.638	0.22	6	0.95 (0.71 to 1.27)	0.734	71 (25 to 96)	0.004	0.40	Less credible
*KRAS*	rs712	1982 vs 2194 (3)	3	1.22 (0.86 to 1.72)	0.270	90 (55 to 100)	6.87E-05	0.19	3	1.72 (0.73 to 4.06)	0.215	87(44 to 100)	3.74E-04	0.16	3	1.17 (0.84 to 1.65)	0.350	83 (32 to 99)	0.003	0.28	Less credible
*ARLTS1*	rs3803185	2281 vs 3196 (5†)	5	1.09 (1.01 to 1.18)	0.026	0 (0 to 76)	0.803	0.35	5	1.08 (0.90 to 1.29)	0.432	43 (0 to 93)	0.133	0.21	5	1.18 (0.95 to 1.46)	0.131	62 (0 to 96)	0.032	0.77	Less credible
*1q32.1*	rs4951291	15 835 vs 16 724 (9‡)	9	0.91 (0.84 to 0.99)	0.021	68 (22 to 88)	0.002	0.85	9	0.74 (0.62 to 0.87)	2.72E-04	0 (0 to 69)	0.616	0.95	9	0.92 (0.83 to 1.01)	0.077	72 (30 to 90)	4.33E-04	0.91	Less credible
*PPAR-gamma*	rs9858822	2152 vs 2630 (5)	4	1.40 (1.19 to 1.65)	4.69E-05	0 (0 to 98)	0.697	0.96	2	1.69 (1.19 to 2.39)	0.003	0 (0 to 98)	0.801	0.54	4	1.47 (1.19 to 1.83)	4.55E-04	0 (0 to 98)	0.618	1.00	Less credible
*DNMT3B*	rs1569686	1224 vs 1381 (5)	4	0.57 (0.47 to 0.68)	1.81E-09	0 (0 to 0)	0.992	1.00	4	0.93 (0.54 to 1.58)	0.775	0 (0 to 97)	0.414	0.06	5	0.46 (0.36 to 0.59)	2.40E-09	30 (0 to 95)	0.223	1.00	Less credible
*NOD2*	rs2066847	4573 vs 3733 (10)	10	1.39 (1.16 to 1.66)	3.21E-04	0 (0 to 71)	0.547	0.78	10	2.36 (0.69 to 8.13)	0.173	0 (0 to 76)	0.720	0.31	10	1.38 (1.14 to 1.66)	0.001	0 (0 to 74)	0.488	0.96	Less credible

*Includes GWAS data from Ontario.

†Tomlinson 2008 was based on 10 samples.

‡Includes GWAS data from SOCCS.

GWASs, genome-wide association studies; SOCCS, Study of CRC in Scotland.

Polymorphisms classified as either ‘positive’ or ‘less-credible positive’ SNPs were sent for validation in synthesised data from the GECCO, CORECT and CCFR consortia. Validation was only able to be performed for 68 out of 77 polymorphisms, as one ‘positive’ variant (rs34612342 in *MUTYH [Y179C]* gene) was dropped due to its low imputation quality in these GWASs; 5 ‘less-credible positive’ polymorphisms (in *GSTT1*, *GSTM1*, *TP73*, *CHEK2* and *CYP2E1*) had no rs numbers; and 3 polymorphisms (in *TP53* and *KRAS*) were not available in these GWASs; therefore, they were not able to be validated. Of the 17 ‘positive’ SNPs sent for validation, 7 polymorphisms (41.2%) in 6 different loci (rs12953717 and rs4464148 in *SMAD7,* rs355527 in *BMP2,* rs10505477 in *8q24,* rs961253 in *20p12.3,* rs16892766 in *8q23.3,* rs3802842 in *11q23.1*) reached a genome-wide statistical significance (p≤5×10^−8^) in at least one meta-analysis model and 6 polymorphisms (35.3%) in 6 different loci (rs7259371 in *RHPN2,* rs2736100 in *TERT,* rs10795668 in *10p14,* rs36053993 in *MUTYH,* rs1862748 in *CDH1* and rs1800469 in *TGFB1*) reached a nominally statistical significance (p<0.05) in at least one meta-analysis model. However, the remaining 4 polymorphisms (23.5%) in 4 different loci (rs2066847 in *NOD2,* rs1569686 in *DNMT3B,* rs9858822 in *PPAR-gamma* and rs4951291 in *1q32.1*) failed in the GWAS validation of all genetic models (online supplementary tables 5–7) and they were therefore downgraded to ‘less-credible positive’. Of the 51 ‘less-credible positive’ SNPs sent for validation, 2 polymorphisms (3.9%) in 2 different loci (rs6983267 in 8q24 and rs1801155 in *APC*) reached a genome-wide statistical significance (p≤5×10^−8^) in at least one meta-analysis model and 11 polymorphisms (21.6%) in 11 different loci (rs1136410 in *PARP1,* rs11568820 in *VDR,* rs1342387 in *ADIPOR1,* rs719725 in *TPD52L3,* rs20417 in *PTGS2/COX2,* rs2665802 in *GH1,* rs4803455 in *TGFB1,* rs7849 in *SCD,* rs8752 in *HPGD,* rs36053993 in *MUTYH* and rs928554 in *ESR2*) reached a nominally statistical significance (p<0.05) in at least one meta-analysis model, whereas the remaining 38 polymorphisms (74.5%) failed in the GWAS validation of all genetic models (online supplementary tables 5–7). Overall, 26 (33.8%) out of the 77 nominally significant polymorphisms tested via meta-analysis were successfully validated in these GWASs (p<0.05).

Funnel plots were produced for all ‘credible’ (online supplementary figures 1–14) and ‘less-credible’ SNPs (online supplementary figures 15–77) for their summary estimates in allelic model. Small study effects were reported for 2 (14.3%) of the 14 ‘positive’ polymorphisms (rs36053993 in *MUTYH* and rs4464148 in *SMAD7*) and for 10 (15.9%) of 63 ‘less-credible’ polymorphisms (rs1229984 in *ADH1B*, rs25487 in *XRCC1*, rs2240308 in *AXIN2*, rs1799750 in *MMP1*, rs1048943 in *CYP1A1*, rs373572 in *RAD18*, rs1800566 in *NQ01*, rs2066844 in *NOD2*, rs2665802 in *GH1*, rs712 in *KRAS*). Therefore, their reported association with CRC risk should be interpreted with caution.

10.1136/gutjnl-2019-319313.supp3 Supplementary data

The remaining 231 polymorphisms (75.0%) assessed in meta-analyses were reported to have no statistically significant association (p>0.05) with CRC risk in all three genetic models (online supplementary table 9), based on accrued data on 192 to 21 929 cases and 251 to 24 054 controls, with median of at least 4017 cases per meta-analysis. Of them, 148 polymorphisms were classified as having negative associations with CRC risk because the number of cases was less than 5000 for which the null results could be due to limited statistical power, while another 83 SNPs with p>0.05 and the number of cases>5000 were classified as null variants with adequate statistical power (online supplementary table 9).

## Discussion

This systematic, comprehensive field synopsis of genetic association studies on CRC updates the two previous field synopses^10 11^ with application of harmonised methods for evidence appraisal and further validation of the identified genetic associations in three GWAS consortia. Specifically, we extracted and collated data for 1063 polymorphisms in 303 different genes from 869 publications and performed up-to-date meta-analyses for 308 variants in 158 different genes that had data from at least three independent studies available for analysis. After credibility assessment and validation, we identified a total of 12 genetic loci credibly associated with CRC risk, of which 6 loci (*MUTYH, SMAD7,* 8q24, 8q23.3, 11q23.1, 20p12.3) were also classified as credibly associated with CRC risk in the previous field synopses and the other 6 loci (*TGFB1, TERT, CDH1, RHPN2, BMP2* and 10p14) are novel findings as they have not been assessed or reported as credible risk loci in the previous field synopses.

We note that a synopsis was undertaken of literature on genetic associations with CRC published in the period 2012–2017.^24^ Our analysis differs from that study, in that it includes and updates data from our previous field synopses,^10 11^ includes data from three GWA studies in the meta-analysis and further validates the findings in three GWA consortia.

Similar to our previous field synopses,^10 11^ the present study reported two SNPs at 8q24 locus (rs6983267 and rs10505477) with strong evidence supporting significant associations with CRC risk and with these associations further replicated in three GWAS consortia. In biopsies of the rectum, sigmoid colon and cecum mucosa, proliferation has been reported to be higher among homozygotes for the risk alleles of the rs6983267 and rs10505477 variants compared with those with other genotypes in the general population.^25^ The rs6983267 variant has been assessed as having a highly credible association with colorectal adenomas.^26 27^ In fine-mapping and bioinformatic analysis performed within the GECCO-CCFR consortia, the rs6983267 variant was appraised as having a strong functional evidence.^28^ The rs6983267 may be a somatic target in CRC^29^ and may be associated with enhanced responsiveness to Wnt signalling.^30^ Furthermore, rs6983267 has also been found to be associated with other types of cancer, including prostate cancer.^31–33^ Interaction with the *MYC* proto-oncogene has been controversial,^34–37^ but in functional studies in cell lines, interaction between enhancer elements in the 8q24 locus and the *MYC* promoter, via transcription factor Tcf-4 binding and allele-specific regulation of *MYC* expression, has been demonstrated.^38^ Expression levels of one of these, *CARLo-5*, in normal colon tissue have been found to be statistically significantly correlated with rs6983267, and chromosome conformation capture analysis of genomic DNA from CRC-derived cell lines provided evidence of physical interaction between the active regulatory region of the *CARLo-5* promoter and the *MYC* enhancer region.^39^ Since the end of our search period (21 November 2018), an analysis of GWAS data on 22 775 cases and 47 731 controls from 14 studies in East Asia detected a genome-wide significant association with the rs6983267 variant.^40^ In addition, in a combined meta-analysis of up to 58 131 cases and 67 347 controls from the GECCO, CORECT and CCFR consortia, in which imputed variants from a whole-genome sequencing analysis and Haplotype Reference Consortium panel variants were included, analysis in the 8q24.21 region conditioned on the rs6983267 and rs7013278 variants identified a genome-wide significant association with the rs4313119.^41^

Two genetic polymorphisms (rs12953717 and rs4464148) tagging in *SMAD7* were identified to be associated with CRC with highly credible evidence. Associations with rs12953717 and rs4464148 were successfully validated in the three GWAS consortia. In fine-mapping and bioinformatics analysis performed within the GECCO-CCFR consortia, rs4464148 was appraised as having less strong functional evidence than the highly correlated rs9932005 variant, which is located within 5 kb away.^28^ The SMAD7 protein is an inhibitor for the TGF-ß signalling pathway.^42 43^ There were highly credible associations with the rs1862748 (tagging *CDH1*), rs355527 (*BMP2*), rs961253 (*BMP2*) and rs7259371 (*RHPN2*) variants, which are TGF-ß related, and replicated in the data from the GWAS consortia. In the recent East Asian analysis, the association with the rs961253 (*BMP2*) variant was replicated, as well as additional variants of *SMAD7* (rs7229639, rs4939827), *CDH1* (rs9929218), *BMP2* (rs4813802) and *RHPN2* (rs10411210).^40^ In the analysis from the GECCO, CORECT and CCFR consortia, conditioned on the rs4813802 and rs189583 variants of *BMP2* as well as each other, novel associations with the *BMP2* variants rs28488 and rs994308 were detected.^41^

Additionally, two variants (rs34612342 and rs36053993) tagging the *MUTYH* gene were highly credibly associated with increased CRC risk, of which rs36053993 was validated in the three GWAS consortia data, while rs34612342 was not tested due to its poor imputation quality. The *MUTYH* gene is known to be involved in the dysfunction of base-excision repair, which is the major pathway for repairing oxidative damage. This biological pathway in which the *MUTYH* gene is involved contributes to the development of multiple colorectal adenomas and carcinomas (*MUTYH*-associated polyposis (MAP) syndrome).^44^ In our analysis, the p values were nominally significant for all models for the rs34612342 variant and for the allelic and dominant models for the rs36053993 variant. For the former variant, the magnitude of effect was greatest for the recessive model, although with wide CIs, which is intriguing as MAP, in which highly penetrant mutations are implicated, and is inherited in an autosomal recessive manner.^45^

Highly credible associations were also reported for four variants tagging four different genes that are involved in inflammation or immune response. First, a positive association with the rs3802842 variant in the 11q23.1 was identified for CRC risk and this association was replicated in the data from the GWAS consortia and in the recent East Asian analysis.^40^ Fine-mapping and bioinformatics analysis performed within the GECCO-CCFR consortia support this variant with strong functional evidence.^28^ Fine mapping identified two genes *COLCA1* and *COLCA2* arranged on opposite strands and sharing a regulatory region containing rs3802842.^46^ It is reported that carrying the risk allele of rs3802842 is associated with the expression levels of *COLCA1* and *COLCA2,* which is further correlated with lymphocyte infiltration of colonic lamina propia. Further, in an expression quantitative trait locus analysis in colorectal tissue, there were signals for *COLCA1* and *COLCA2*.^47^ The polymorphism rs10795668 in 10p14 locus tagging *GATA3* gene was reported with a highly credible association with CRC, and this association was further replicated in the GWAS consortia and in the recent East Asian analysis.^40^ However, in fine-mapping and bioinformatics analysis, this variant presents with weak functional evidence.^28^ Another less-credible positive association with the rs9858822 variant of *PPARγ* was identified; however, this association was not replicated in the GWAS consortia. Bioinformatics analysis showed that PPARγ and its ligands have been found to block proinflammatory genes in colon cancer cell lines, activated macrophages and monocytes.^48^ *PPARγ* is also involved in lipid metabolism, adipocyte differentiation, and glucose homeostasis and insulin sensitivity.^48^ A less-credible positive association also reported one variant (rs2066847) in *NOD2* gene; however, this association was not replicated. Evidence from experimental studies in mice investigating the role of *Nod2* in colorectal tumour risk has also been inconsistent.^49–51^ The recent analyses in East Asia,^40^ the GECCO, CORECT and CCFR consortia^41^ and of five UK studies and a further 10 from the COGENT consortium^47^ identified new associations in the major histocompatibility region.

The remaining two variants with highly credible associations with CRC risk included rs2736100 tagging *TERT* and rs16892766 at 8q23.3 tagging *EIF3H*. These variants were all validated in the GWAS consortia datasets. Fine-mapping and bioinformatics analysis performed within the GECCO-CCFR consortia supported these variants with strong functional evidence, for which polymorphism rs2736100 in *TERT* gene has been reported to be associated with telomere length and the risk of different types of cancer and chronic diseases other than cancer.^52^ For the association with the rs16892766 variant at 8q23.3 tagging *EIF3H*, another variant rs16888589 was previously reported with the lowest p value for the association with CRC in this locus.^25^ Functional analysis and chromosome conformation capture analysis in CRC cell lines have found that the genomic region harbouring rs16888589 increases *EIF3H* expression,^53^ but analysis of expression quantitative trait loci around rs16892766 suggested that *UTP23* rather than *EIF3H* is the target of genetic variation associated with CRC in this region.^54^

The less credible associations with 63 variants of 52 genes involved the following pathways—adhesion (*AXIN2*, *MMP1*); alcohol metabolism (*ADH1B*); angiogenesis (*VEGF*); blood clotting (*SERPINE1*); DNA repair (*CHEK2, ERCC5, MSH2, MSH3, PARP1, RAD18, XPC, XRCC1*); hormone metabolism (*ESR2, GH1, PGR*); inflammation and immune response (*CRP, HPGD, PTGS2/COX2*); inhibition of cell growth (*CCND1, EGF, TGFB1*); iron metabolism (*HFE*); lipid metabolism (*ADIPOQ, ADIPOR1, LIPC, SCD*); one-carbon metabolism (*MTHFR*, *MTFD1, MTRR*); substrate metabolism (*ABCB1; CYP1A1, CYP2C9, CYP2E1, GSTM1, GSTT1, NAT2, NQ01*); tumour suppression (*ARLTS1, miR, TP73*); vitamin D metabolism (*VDR*)—common low penetrance variants at 1q32.1 (rs4951039, *LINC00303*) and 9p24 (rs719725); and the common rs1801155 (I1307K) variant of *APC,* for which large numbers of rare variants have been identified,^55^ and rs63750447 (V384D) variant of *MLH1*, for which rare variants confer a high risk of Lynch syndrome.^56^ These variants were classified as less-credible SNPs because of either the substantial heterogeneity or the high possibility of false positive; however, we would like to highlight a number of less-credible genetic loci (*PARP1, MYC, VDR, ADIPOR1, APC, PTGS2/COX2, SCD, HPGD* and *ESR2*), which were replicated in the GWAS data and for which their linked pathways are worthy of further investigation in future studies.

Updating field synopses is challenging because genetic analysis is such a fast-moving field. Recent trends highlighted by three articles published since we completed our search in November 2018 include the extension of consortia to increase statistical power to detect and replicate associations,^47^ the investigation of populations other than of European origin,^40^ and the use of whole-genome sequencing and more comprehensive reference panels to extend the range of genetic variants considered to include those that are rare or of low frequency.^41^ These three articles have added new loci for CRC susceptibility and indicate that most of the risk loci previously associated with CRC in populations of European origin are also associated with CRC risk in East Asian populations.

We checked whether the 14 variants we classified as ‘highly credible’ were replicated in the paper of Law *et al*.^47^ For one variant, rs34612342 *MUTYH*, there was poor imputation quality. However, as there was no satisfactory proxy for this, we report the available information, which supported the association (p=0.029). All the remaining 13 variants had p values for association less than 5.0×10^−5^ with no more than moderate heterogeneity. Such an empirical comparison was not appropriate with the data of Huyghe *et al* 41 because of the considerable overlap of included participants.

In summary, we have conducted a comprehensive study to capture and meta-analyse all SNP data for common genetic variants. The analysis clearly identifies 14 variants at 12 loci for which there is robust evidence of their impact on CRC risk, 63 variants at 52 loci for which further evidence through international collaboration should be generated and 231 variants for which the overall evidence does not support any association with CRC risk. With increasing availability of data from multiple SNPs, it is clear that studies to test associations must achieve very high levels of statistical stringency. Nonetheless, the analysis here provides a resource for mining available data and puts into context the sample sizes required for the identification of true associations for common genetic variants. Future resequencing studies are expected to identify rarer variants (eg, prevalence 0.05%–5%) with intermediate or perhaps even large effects, and GWAS of structural variation will likely identify deletions, amplifications and other copy number variations that may also influence CRC risk.^6^ This study highlights a number of common genetic variants that could be incorporated into genetic risk-prediction algorithms as further risk factors are identified and highlights the loci at which further research effort should be targeted. All data are available from the CRCgene2 database.
