# Supplementary material for: Systematic meta-analyses, field synopsis and global assessment of the evidence of genetic association studies in colorectal cancer
Source: Gut. 2019 Dec 9;69(8):1460–71. doi: 10.1136/gutjnl-2019-319313 (PMC7398467; doi:10.1136/gutjnl-2019-319313)
Supplement: Supplementary data [file gutjnl-2019-319313supp001.pdf]

Supplementary Text

1. GWAS datasets (SOCCS, ARCTIC and EPICOLON) that were incorporated into the meta-analyses:

The Scottish Colorectal Cancer Study (SOCCS) is a population-based case-control study of colorectal cancer. It was funded by the Cancer Research UK (CR-UK), the Medical Research Council (MRC) and the Chief Scientist Office of the Scottish Executive (CSO) and was headed by Professors Malcolm G Dunlop, Harry Campbell and Mary E Porteous. The recruitment for this study commenced in February 1999 and ended in December 2006. In total 3,417 colorectal cancer cases and 3,500 controls were recruited in the study.

The Assessment of Risk for Colorectal Tumors In Canada (ARCTIC), aimed to explore the genetic susceptibility to colon cancer. It is co-led by Dr. Zanke and Dr. Tom Hudson of the McGill University and Genome Quebec Innovation Centre, Canada. Through the Ontario Familial Colorectal Tumor Registry, the ARCTIC program have collected a large repository of colon tumor tissue and blood from families with colon tumors. Genotype data were made available for 1,231 cases and 1,240 controls.

The EPICOLON consortium was initiated in 1999 by the Gastrointestinal Oncology Group of the Spanish Gastroenterology Association. It recruited consecutive, unselected, population-based colorectal cancer (CRC) cases and control subjects matched by age and gender without personal or familial history of cancer all over Spain. This epidemiological, prospective and multicentre study collected extensive clinical data and biological samples from ∼2000 CRC cases and 2000 controls in Phases 1 and 2 involving 25 and 14 participating hospitals, respectively.

2. GWAS datasets (GECCO, CORECT, and CCFR) that were used for the validation of associations with “positive” and “less-credible” SNPs.

GECCO (Genetics and Epidemiology of Colorectal Cancer Consortium) is a collaborative effort of researchers from North America, Australia, and Europe, using data from over 40,000 participants. GECCO aimed to find related variants to CRC by replicating and characterizing GWAS findings. GECCO group studied the impact of common and rare genetic variants across the entire genome by performing whole genome sequencing and genotyping.

CORECT (Colorectal Transdisciplinary Study) is based on investigating and identifying loci for CRC to characterize the biologic basis of inherited susceptibility and recognize how genetic variation may be quantified and modified by environmental risk factors. CORECTis part of Genetic Associations and Mechanisms in Oncology (GAME-ON) with goal of providing a rigorous knowledge base that would enable clinical translation and public health dissemination of cancer GWAS findings.

CCFR (Colon Cancer Family Registry) was funded by National Cancer Institute with collaboration of 6 centers - Fred Hutchinson Cancer Research Center (FHCRC), Lunenfeld-Tanenbaum Research Institute (LTRI), Mayo Clinic, University of Hawaii (UHI), University of Melbourne (Australasia), and University of Southern California Consortium (USC). CCFR dedicated to the establishment of a comprehensive collaborative infrastructure for interdisciplinary studies in the genetic epidemiology of colorectal cancer. This includes data from approximately 30,500 total subjects (10,500 probands and 20,000 unaffected and affected relatives and unrelated controls).
