# Supplementary material for: Systematic meta-analyses, field synopsis and global assessment of the evidence of genetic association studies in colorectal cancer
Source: Gut. 2019 Dec 9;69(8):1460–71. doi: 10.1136/gutjnl-2019-319313 (PMC7398467; doi:10.1136/gutjnl-2019-319313)
Supplement: Supplementary data [file gutjnl-2019-319313supp002.pdf]

Supplementary Table S1. List of genes and variants that were selected for meta-analysis (sorted by gene pathway).

Gene	Variant	rs number	Cases vs. controls (number of samples)	Ref allele	Ref allele freq cases	Ref allele freq controls
**Adhesion molecules**						
CDH1	C-160A	rs16260	7261 vs. 7230(10)	C	0.73	0.73
CDH1		rs1862748	17436 vs. 18418(11)	C	0.71	0.69
MMP1	G-1607GG	rs1799750	1660 vs. 2024(10)	G	0.63	0.60
MMP3	AAAAA-1612AAAAAA	rs3025058	1212 vs. 1631(8)	5A	0.66	0.66
MMP2	C-1306T	rs243865	1215 vs. 1347 (8)	C	0.80	0.82
MMP7	181A/Gæ	rs11568818	2139 vs. 2355 (9*)	A	0.65	0.66
MMP9	R279Q	rs17576	1520 vs. 1753 (4*)	A	0.67	0.64
MMP9	1562C/T	rs3918242	621 vs. 904 (5)	C	0.89	0.87
CX3CR1	V249I	rs3732379	3548 vs. 3649(3*)	C	0.72	0.72
CX3CR1	T280M	rs3732378	6110 vs. 6335(4*)	G	0.83	0.83
DLG5	R30Q	rs1248696	3672 vs. 3618(3*)	C	0.90	0.89
ICAM1	R241G	rs1799969	3275 vs. 3368(3*)	G	0.88	0.89
ICAM1	K469E	rs5498	6391 vs. 6611(6*)	A	0.59	0.58
AXIN2	P50S	rs2240308	4723 vs. 4932 (4*)	G	0.51	0.52
**Alcohol metabolism**						
ADH1B	Arg47His	rs1229984	4449 vs. 6995(9)	His	0.80	0.81
ADH1C	Ile349Val (1045A>G)	rs698	4545 vs. 18180(9**)	Ile (A)	0.67	0.62
ALDH2	Glu487Lys	rs671	5561 vs. 8415(14)	Glu (G)	0.87	0.84
ALDH2	355G>A	rs886205	2595 vs. 2993 (4*)	G	0.84	0.85
**Angiogenesis**						
VEGF	936 C>T	rs3025039	3090 vs. 3147(11)	C	0.80	0.81
VEGF	G634C	rs2010963	2426 vs. 2387(7)	C	0.62	0.60
VEGF	460C>T	rs833061	989 vs. 897 (4)	T	0.56	0.59
VEGF	1154G>A	rs1570360	1150 vs. 1342 (4)	G	0.72	0.72
VEGF	2578C>A	rs699947	4497 vs. 5334 (10*)	C	0.55	0.56
ACE	I/D	x	649 vs. 6556 (4)	del	0.52	0.53
**Base-excision repair**						
MGMT	Leu84Phe	rs12917	8296 vs. 11815(9*)	C	0.86	0.87
MGMT	Il3143Val	rs2308321	2863 vs. 8040(6)	A	0.88	0.88
MUTYH	G396D	rs36053993	28302 vs. 20935(19*)	G	0.99	0.99
MUTYH	Y179C	rs34612342	28180 vs. 20923(19*)	A	1.00	1.00
MUTYH	V22M	rs3219484	16671 vs. 14985 (13*)	G	0.93	0.93
MUTYH	G972C	rs3219489	17936 vs. 19154 (20)	G	0.74	0.75
OGG1	Ser326Cys	rs1052133	8090 vs. 10486 (21)	C	0.69	0.67
XRCC1	Arg194Trp	rs1799782	7643 vs. 11883(25)	C	0.81	0.82
XRCC1	Arg280His	rs25489	6510 vs. 7889(10)	G	0.94	0.93
XRCC1	Arg399Gln	rs25487	13017 vs. 18166(37)	G	0.67	0.68
XRCC2	Arg188His	rs3218536	5977 vs. 6174 (4*)	G	0.91	0.92
XRCC3	Thr241Met	rs861539	7995 vs. 9886(21)	T	0.70	0.70
ERCC1	Asn118Asn	rs11615	4231 vs. 4928(7*)	T	0.62	0.62
ERCC1	8092C>A	rs3212986	1815 vs. 1858(4*)	C	0.73	0.75
ERCC2	D312N	rs1799793	6722 vs. 9585(11)	G	0.65	0.67
ERCC2	Lys751Gln	rs13181	10239 vs. 13833(24*)	A	0.67	0.68
ERCC4	Arg415Gln	rs1800067	6896 vs. 7431(8*)	G	0.93	0.92
ERCC5	D1104H	rs17655	9653 vs. 11367(14)	G	0.70	0.72
APE1	Glu51His	rs1048945	3224 vs. 3369 (3)	G	0.96	0.96
APE1	Asp148Gl	rs1130409	5786 vs. 6550 (11)	T	0.54	0.55
XPA		rs1800975	2308 vs. 2871(6**)	G	0.67	0.66
CHEK2	c.470T>C	rs1787996	1687 vs. 3370 (3)	T	0.97	0.98
CHEK2	1100delC	x	2417 vs. 3615 (4)	non-carriers	0.99	0.99
EXO1	T439M	rs4149963	6597 vs. 6842(5*)	C	0.92	0.92
EXO1	P757L	rs9350	5459 vs. 5926(4*)	C	0.83	0.83
MSH2	IVS12-6T>C	rs2303428	4371 vs. 4590(5)	T	0.90	0.89
MSH2	118T>C	rs2303425	1775 vs. 2206(3)	T	0.87	0.87
MSH2	Gly322Asp	rs4987188	3719 vs. 3852(6)	G	0.98	0.98
MSH2		rs1981929	4319 vs. 4009(3*)	A	0.60	0.60
MSH2		rs4608577	4308 vs. 4011(3*)	T	0.83	0.82
MSH2		rs4952887	4321 vs. 4011(3*)	C	0.91	0.91
MSH3	Q949R	rs184967	8151 vs. 10103(4*)	G	0.84	0.85
MSH3	A1036T	rs26779	6050 vs. 8024(6*)	C	0.67	0.69
MSH6	G39E	rs1042821	4936 vs. 8223(6)	G	0.82	0.83
MSH6	276A>G (P92P)	rs1800932	12214 vs. 10598(9)	A	0.82	0.82
MSH6	540T>C (D180D)	rs1800935	14234 vs. 12089(11*)	T	0.71	0.70
MSH6		rs1800936	3867 vs. 4019(3*)	C	0.85	0.87
MSH6		rs3136245	4314 vs. 4013(3*)	C	0.80	0.79
MSH6		rs3136329	4305 vs. 3996(3*)	T	0.58	0.58
MSH6		rs6713506	4306 vs. 3995(3*)	G	0.84	0.84
NBS1	185G>C	rs1805794	3948 vs. 4316(5)	C	0.65	0.64
WRN	V114I	rs2230009	7662 vs. 7908(4*)	G	0.94	0.94
WRN	L1074F	rs2725362	4681 vs. 4833(3)	G	0.56	0.55
WRN	C1367R	rs1346044	8548 vs. 8816(5*)	T	0.75	0.75
XPC	Ala499Val	rs2228000	2677 vs. 4253(6)	G	0.70	0.68
XPC	Lys939Gln	rs2228001	7650 vs. 9769(13*)	T	0.60	0.62

PMS2		rs6463524	3713 vs. 3374(3)	C	0.82	0.81
PMS2		rs2286680	4320 vs. 4010(3*)	C	0.86	0.86
PMS2		rs235060	4315 vs. 4006(3*)	T	0.76	0.75
RAD18	905G>A	rs373572	6560 vs. 6906(5*)	G	0.70	0.71
RAD51	G135C	rs1801320	853 vs. 840(5)	G	0.59	0.67
ATM	D1853N	rs1801516	3477 vs. 6951 (3*)	G	0.86	0.84
**Inflammation/immune response**						
IL6	174G>C	rs1800795	7858 vs. 9345(13§**)	G	0.61	0.60
IL8	251T/A	rs4073	6060 vs. 7435(12**)	T	0.54	0.54
IL10	1082G/A	rs1800896	4510 vs. 5363(9*)	A	0.50	0.50
IL1B	T31C	rs1143627	1251 vs. 2124 (5)	G	0.65	0.66
IL1B	511 C>T	rs16944	577 vs. 828 (3)	A	0.65	0.64
IL4	C590T	rs2057768	1123 vs. 1483 (5)	C	0.82	0.80
IL4R	R431C	rs1805012	3801 vs. 3962 (3*)	T	0.88	0.89
IL4R	S503P	rs1805015	3607 vs. 3692 (3*)	T	0.83	0.83
IL4R	Q576R	rs1801275	1764 vs. 1747 (4*)	A	0.81	0.81
IL6	597G>A	rs1800797	2619 vs. 3396 (3)	G	0.57	0.57
IL6R	D358A	rs8192284	3070 vs. 20173 (3)	A	0.60	0.69
IL10	592 C/A	rs1800872	1598 vs. 2760 (7)	C	0.77	0.78
IL16	Pro434Ser	rs4072111	6160 vs. 6643 (4*)	C	0.88	0.88
PPAR-gamma		rs9858822	2152 vs. 2630 (5)	A	0.91	0.90
PPAR-gamma	C1431T	rs3856806	8098 vs. 11100(11*)	C	0.87	0.88
PPAR-gamma	Pro12Ala	rs1801282	15855 vs. 24348(22§**)	C	0.90	0.90
PTGS1/COX1	50C>T	rs3842787	5754 vs. 7916 (4)	C	0.95	0.95
PTGS1/COX1	G213G	rs5788	4087 vs. 4808 (3)	C	0.86	0.86
PTGS1/COX1	Leu237Met	rs5789	8164 vs. 10969 (6*)	C	0.90	0.91
PTGS1/COX1	262A>T	rs10306135	4644 vs. 6893 (3)	A	0.85	0.85
PTGS1/COX1	268A>G	rs6478565	4418 vs. 5909 (3*)	A	0.84	0.83
PTGS1/COX1	R8W	rs1236913	9511 vs. 11189 (6*)	T	0.93	0.93
PTGS2/COX2	78C>T	rs4648261	4687 vs. 6951 (3)	C	0.97	0.97
PTGS2/COX2	565A>G	rs2745557	4300 vs. 5825 (5)	G	0.79	0.81
PTGS2/COX2	C1629G	rs20424	3590 vs. 4927 (3)	G	0.98	0.98
PTGS2/COX2		rs2206593	5391 vs. 6096 (3*)	G	0.88	0.88
PTGS2/COX2	A1195G	rs689466	7790 vs. 11652(12)	A	0.78	0.77
PTGS2/COX2	A1803G	rs4648298	2359 vs. 2538(5)	A	0.97	0.97
PTGS2/COX2	C427T	rs5275	12396 vs. 15884(13*)	T	0.66	0.66
PTGS2/COX2	G306C	rs5277	5740 vs. 7509(7)	G	0.84	0.84
PTGS2/COX2	G765C	rs20417	7785 vs. 11371(18)	G	0.83	0.85
PTGS2/COX2	T1532C	rs5273	720 vs. 1163(4)	val/val	na	na
TNF-alpha	308G>A	rs1800629	2980 vs. 4102(13)	G	0.84	0.86
TNF-alpha	238G>A	rs361525	292 vs. 351 (4)	G	0.99	0.96
TNF-alpha	857C>T	rs1799724	845 vs. 832 (4)	C	0.87	0.87
TNF-alpha	1031T>C	rs1799964	1959 vs. 1948 (3§)	T	0.80	0.79
NOD2	3020incC	rs5743293	4573 vs. 3733(10)	G	0.96	0.97
NOD2	G908R	rs2066845	3303 vs. 3088(9)	G	0.99	0.99
NOD2	R702W	rs2066844	3297 vs. 3088(9)	C	0.96	0.97
CRP	L184L	rs1800947	2853 vs. 3381(3)	C	0.94	0.94
CRP	2042C>T	rs1205	3037 vs. 9333(4)	C	0.63	0.67
CTLA4	49A>G	rs231775	777 vs. 1356(5)	A	0.61	0.60
DNMT3B	C149T	rs2424913	2398 vs. 3740(5)	C	0.59	0.56
DNMT3B	G579T	rs1569686	1224 vs. 1381(5)	T	0.89	0.83
NFKB1	94 ins/delATTG	rs28362491	2897 vs. 4961(11)	94 ins	0.56	0.57
NFKB1A		rs696	2310 vs. 3018(4*)	C	0.59	0.59
TLR4	Asp299Gly	rs4986790	4550 vs. 5432(10*)	A	0.93	0.94
TLR4	Thr399Ile	rs4986791	3387 vs. 4072(6)	C	0.94	0.94
CASP8	-CTTACT(	rs3834129	7226 vs. 11366(12)	del	0.58	0.59
MBL2	R52C	rs5030737	1185 vs. 1199(4)	C	0.93	0.94
MBL2	Codon54	rs1800450	6362 vs. 6511(5*)	G	0.86	0.87
MBL2	Codon57	rs1800451	819 vs. 843(3)	G	0.95	0.94
MBL2	X/Y 221	rs7096206	826 vs. 871(3)	G	0.81	0.80
MBL2	variant H/L	rs11003125	822 vs. 870(3)	G	0.67	0.68
MBL2	PQ	rs7095891	822 vs. 870(3)	G	0.74	0.71
RNASEL	Arg462G	rs486907	3524 vs. 3789(3*)	G	0.66	0.66
**Cell growth, differentiation, apoptosis**						
CCND1	870A	rs17852153	6500 vs. 8885(20)	G	0.47	0.48
TGFB1	C509T	rs1800469	4021 vs. 6024(10)	T	0.66	0.61
TGFBR1	TGFBR1*6A	rs11466445	6705 vs. 8162(12**)	C	0.90	0.91
TGFB1	G800A	rs1800468	834 vs. 1579 (3)	G	0.85	0.71
TGFB1	Leu10Pro	rs1800470	998 vs. 1383 (4)	A	0.57	0.57
TGFB1		rs4803455	3747 vs. 4513 (3*)	C	0.49	0.52
TGFBR1	Int7G24A	rs334354	1012 vs. 1923 (3)	G	0.74	0.70
EGF	A61G	rs4444903	899 vs. 976(5)	G	0.58	0.53
PARP1	Val762Ala	rs1136410	7002 vs. 8328(7*)	A	0.81	0.79
CDKN1A	3UTRC>T	rs1059234	1036 vs. 1512 (3)	C	0.78	0.69
CDKN1A	codon31C	rs1801270	1038 vs. 1513 (3)	C	0.79	0.70
KRAS	let-7 KRAS	rs712	1982 vs. 2194 (3)	C	0.66	0.65
BMP2		rs355527	13539 vs. 14375(9)	C	0.66	0.69

BMP4	V152A	rs17563	3505 vs. 2518 (4)	C	0.50	0.45
TERT		rs2736100	16176 vs. 18135(8)	G	0.49	0.51
Survivin31	31G/C	rs9904341	1452 vs. 1475(4)	G	0.49	0.54
**Insulin related**						
IGF1	CA-repeat	n/a	9335 vs. 11089(9)	19/19	0.63	0.61
IGFBP3	202A>C	rs2854744	10880 vs. 15046(12)	A	0.50	0.52
IGF1	C1410T	rs35767	2792 vs. 4988 (3)	C	0.75	0.75
IGF1		rs6214	1248 vs. 3005 (3*)	C	0.58	0.61
IGFBP1	Ile253Met	rs4619	6537 vs. 6826 (4*)	A	0.64	0.64
IGFBP3	Ala32Gly	rs2854746	4484 vs. 7577 (6)	C	0.51	0.51
IGFBP3		rs3110697	3079 vs. 3860 (3*)	G	0.59	0.59
IRS	G972R	rs1801278	9726 vs. 10756(9**)	G	0.92	0.93
IRS2	G1057D	rs1805097	3890 vs. 4778(3)	G	0.63	0.62
**Iron metabolism**						
HFE	C282T	rs1800562	6547 vs. 34156(10*)	C	0.92	0.93
HFE	H63D	rs1799945	1495 vs. 2038 (5)	C	0.86	0.88
**Mitotic control**						
AURKA	F31I	rs2273535	5221 vs. 4806 (5)	T	0.76	0.76
**Carbohydrate metabolism**						
MTHFR	C677T	rs1801133	43652 vs. 60883(94§**)	C	0.67	0.66
MTHFR	A1298C	rs1801131	23523 vs. 35193(57§)	A	0.70	0.69
MTHFR	1793G>A	rs2274976	2860 vs. 3414 (5*§)	G	0.95	0.94
MTRR	524 C>T	rs1532268	4457 vs. 5807 (4*)	C	0.63	0.63
MTRR	1049A>G	rs162036	6473 vs. 7859 (4*)	A	0.88	0.89
MTRR	G1763A	rs10380	9022 vs. 10910 (7*)	G	0.90	0.90
MTHFD1	Arg134Lys	rs1950902	9059 vs. 11358(7*)	G	0.82	0.82
MTHFD1	R653Q	rs2236225	11673 vs. 15250(10*)	G	0.54	0.54
MTR	A2756G	rs1805087	18831 vs. 24357 (28*§)	A	0.81	0.81
MTRR	A66G	rs1801394	10175 vs. 15506 (21)	A	0.50	0.50
TS	TSER	rs34743033	5860 vs. 7540 (11)	3R/3R	0.63	0.63
TS	Ts1494del6	rs34489327	5786 vs. 6856 (8)	ins/ins	0.68	0.68
**Rare, high penetrance**						
APC	E1317Q	rs1801166	7161 vs. 8140 (7)	G	0.99	0.99
APC	D1822V	rs459552	21929 vs. 20943 (22**)	Asp	0.77	0.78
MLH1	A655G	rs1799977	15398 vs. 16730 (20*)	A	0.69	0.69
MLH1	-93 G>A	rs1800734	21837 vs. 18831 (15*§)	G	0.77	0.78
APC	I1307K	rs1801155	2389 vs. 4223 (5)	T	0.97	0.97
MLH1	V384D	rs63750447	937 vs. 812 (4)	T	0.96	0.98
MLH1		rs3774332	7556 vs. 7299 (4*)	A	0.92	0.92
**Substrate metabolism**						
CYP1A1	2454A>G	rs1048943	9661 vs. 11774 (19§)	A	0.89	0.89
CYP1A1	3698T>C	rs4646903	9725 vs. 12104 (20)	T	0.84	0.82
CYP1A2	163C>A	rs762551	9669 vs. 10852 (16)	A	0.70	0.70
CYP1B1	4326C>G	rs1056836	10318 vs. 11251 (12)	C	0.55	0.55
CYP2C9	430C>T	rs1799853	9588 vs. 11428 (12)	C	0.86	0.85
CYP2C9	1075A>C	rs1057910	15444 vs. 17426 (13*)	A	0.92	0.91
CYP2E1	1053C>T	rs2031920	5216 vs. 6026 (11§)	C	0.90	0.89
CYP2E1	1293G>C	rs3813867	5035 vs. 6688 (10)	G	0.93	0.92
CYP1A1	Thr461Asp	rs1799814	4389 vs. 4621 (4§)	C	0.96	0.96
CYP1A2	1545T>C	rs2470890	3638 vs. 3885 (4)	T	0.60	0.59
CYP1A2	3858G>A	rs2069514	1283 vs. 1305 (5)	G	0.80	0.80
CYP1B1	119G>T	rs1056827	3153 vs. 3199 (4)	G	0.70	0.71
CYP1B1	142C>G	rs10012	5336 vs. 5850 (6)	C	0.70	0.71
CYP1B1	4390A>G	rs1800440	11152 vs. 11918 (9*)	A	0.82	0.82
CYP17A1	T34C	rs743572	5009 vs. 5163 (3)	T	0.60	0.60
CYP2C19	681G>A	rs4244285	3385 vs. 3498 (5)	G	0.86	0.85
CYP2D6	G1846A	rs3892097	1153 vs. 1806 (4)	G	0.81	0.80
CYP2E1	96-bp insertion		1614 vs. 2396 (6)	non-ins	0.86	0.89
CYP2E1	7632T>A	rs6413432	3893 vs. 4643 (8)	T	0.87	0.85
CYP3A4	C4358G	rs11773597	5005 vs. 5155 (3)	G	0.93	0.93
CYP3A4	G4713A	rs2740574	5138 vs. 5295 (4)	A	0.96	0.96
GSTA1	69C>T	rs3957357	806 vs. 1103 (4)	C	0.61	0.62
GSTM1	K173N	rs1065411	828 vs. 873 (3)	G	0.61	0.62
GSTM3	delAGG	rs1799735	3505 vs. 4108 (5)	ins	0.84	0.84
GSTM1	Null variant	n/a	28240 vs. 38880 (74)	present	0.57	0.61
GSTP1	Ile105Val	rs1695	16373 vs. 20872 (38*)	IA	0.70	0.71
GSTP1	Ala114Val	rs1138272	12144 vs. 12586 (13*§)	C	0.91	0.91
GSTT1	Null variant	n/a	19133 vs. 27821 (56)	present	0.63	0.62
NAT1	slow/rapid	n/a	4791 vs. 6628 (15)	slow	0.69	0.68
NAT2	slow/rapid	n/a	8057 vs. 10431 (45)	slow	0.63	0.62
NAT1	459G>A	rs4986990	2253 vs. 2768 (3)	G	0.98	0.98
NAT1	1088T>A	rs1057126	2251 vs. 2747 (3)	T	0.78	0.78
NAT2	G286E	rs1799931	2510 vs. 3130 (5)	G	0.95	0.95
NAT2	T341C	rs1801280	2470 vs. 2971 (5)	T	0.58	0.55
NAT2	481C>T	rs1799929	1861 vs. 1952 (5§)	C	0.60	0.55
NAT2	590G>A	rs1799930	6446 vs. 7193 (8§)	G	0.70	0.71
NQ01	Pro187Ser (C609T)	rs1800566	11183 vs. 12525 (16*)	C	0.78	0.78

NQ01	Arg139Trp	rs1131341	4436 vs. 4725 (4)	C	0.96	0.96
EPHX1	Y113H	rs1051740	11718 vs. 13560(19*)	T	0.69	0.69
EPHX1	Arg139His	rs2234922	6570 vs. 7960(14*)	A	0.81	0.80
FOLH1	His475Tyr	x	3389 vs. 4333(4)	His	0.82	0.83
GPX1	Pro198Leu	rs1050450	1974 vs. 2481(4)	C	0.72	0.70
GPX4		rs713041	1733 vs. 1519(3)	C	0.60	0.60
HPGD		rs8752	3968 vs. 4830(3*)	A	0.57	0.58
HPGD		rs9312555	3976 vs. 4831(3*)	A	0.85	0.85
HPGD		rs17360144	3962 vs. 4826(3*)	A	0.93	0.93
LCT	13910C>T	rs4988235	1927 vs. 2182(6)	C	0.55	0.55
NOS3	Glu298Asp	rs1799983	2301 vs. 2478(5)	G	0.78	0.77
NOS3	Y786C	rs2070744	1576 vs. 1782(3)	T	0.81	0.79
ABCB1 (MDR1)	61A>G	rs9282564	9536 vs. 9145 (6*)	A	0.89	0.90
ABCB1 (MDR1)	1199G>A	rs2229109	6143 vs. 6104 (5§)	G	0.95	0.95
ABCB1 (MDR1)	C1236T	rs1128503	246 vs. 399 (3)	C	0.55	0.42
ABCB1 (MDR1)	G2677A	rs2032582	1008 vs. 1100 (5)	G	0.39	0.43
ABCB1 (MDR1)	3435T>C	rs1045642	7537 vs. 8396 (16)	T	0.52	0.51
ABCB1 (MDR1)		rs868755	2958 vs. 3255 (3)	G	0.56	0.58
ABCB1 (MDR1)		rs1202168	7000 vs. 6485 (5)	C	0.57	0.59
ABCB1 (MDR1)		rs2214102	7865 vs. 7919 (5*)	C	0.92	0.92
ABCB1 (MDR1)		rs3789243	2724 vs. 3385 (4*§)	G	0.50	0.51
UGT1A1	3156G>A	rs10929302	1560 vs. 1952(3)	G	0.64	0.65
UGT1A1	3279T>G	rs4124874	1560 vs. 1987(3)	T	0.58	0.58
UGT1A1	UGT1A1*28	x	3344 vs. 4159(8)		0.66	0.66
UGT1A6	T181A	rs2070959	4773 vs. 5070(4*)	A	0.68	0.69
UGT1A6	R184S	rs1105879	7334 vs. 7774(5*)	A	0.66	0.67
UGT1A7		x	2316 vs. 2918(6)		0.56	0.58
SULT1A1	c.638G>A	rs9282861	4636 vs. 6291(12)	G	0.68	0.67
SULT1A2	Asn235Thr	rs1059491	1806 vs. 1986(3)	A	0.67	0.68
SHMT1	C1420T	rs1979277	1697 vs. 1994(5)	C	0.70	0.68
CBS	844ins68	rs5742905	2197 vs. 2579 (7)	T	0.94	0.94
ALOX12	Q261R	rs1126667	3736 vs. 4610 (7*)	G	0.55	0.57
PLA2G2A	c.435+230C>T	rs11677	5887 vs. 6774(4*)	G	0.88	0.88
SOD2	V16A	rs4880	2496 vs. 2283(4)	C	0.64	0.64
COMT	V158M	rs4680	9830 vs. 10176 (7*)	G	0.51	0.50
**Tumour supressor genes**						
TP53	Arg72Pro	rs1042522	10591 vs. 12673 (30)	G	0.66	0.68
TP53	intron 3 16bp	rs17878362	1812 vs. 2319 (6)	Del	0.85	0.85
MDM2	309 T/G	rs2279744	3564 vs. 2835 (10)	G	0.52	0.53
TP73	G4C14	x	858 vs. 1173(4)	wild	0.72	0.76
ARLTS1	T442C	rs3803185	2281 vs. 3196 (5**)	T	0.50	0.54
ARLTS1	G446A	rs3430134	1010 vs. 1862 (3)	G	0.99	0.99
miR	pre-miR-27a	rs895819	1322 vs. 1641(4)	T	0.59	0.60
miR	149 C-T	rs2292832	2355 vs. 2571(5*)	C	0.69	0.68
miR	146a C-G	rs2910164	5127 vs. 4110(8)	G	0.70	0.53
miR	196a2 C-T	rs11614913	2101 vs. 2961(7)	C	0.50	0.50
miR	499	rs3746444	717 vs. 1055(3)	A	0.78	0.83
**Vit D and Ca metabolism**						
VDR	BsmI (60890GA)	rs1544410	14789 vs. 15922 (16*)	G	0.61	0.59
VDR	TaqI	rs731236	2091 vs. 2257 (9)	C	0.63	0.65
VDR	23005G>A	rs2238136	4584 vs. 4621 (5*)	G	0.73	0.73
VDR	27823C>T	rs2228570	16386 vs. 18814 (17**)	C	0.60	0.59
VDR	29648A>G	rs11568820	4001 vs. 4682 (5§)	G	0.77	0.79
VDR	61888G>T	rs7975232	3301 vs. 3402 (5)	C	0.54	0.56
**Common low penetrance**						
SMAD7	rs4939827	rs4939827	43924 vs. 44264 (21*)	T	0.59	0.56
SMAD7	rs12953717	rs12953717	28006 vs. 26539 (13*)	C	0.62	0.64
SMAD7	rs4464148	rs4464148	17772 vs. 17356 (9*)	T	0.63	0.66
8q24	rs6983267	rs6983267	51730 vs. 53589 (34***)	A	0.50	0.53
8q24	rs10505477	rs10505477	18562 vs. 20132 (14)	C	0.46	0.49
9p24	rs719725	rs719725	13513 vs. 14999 (14)	C	0.38	0.39
20p12.3	rs961253	rs961253	22971 vs. 25270 (14*)	C	0.66	0.68
8q23.3	rs16892766	rs16892766	8351 vs. 8878 (6*)	A	0.89	0.91
10p14	rs10795668	rs10795668	16763 vs. 18302 (16*)	G	0.69	0.67
11q23.1	rs3802842	rs3802842	22320 vs. 22965 (20*)	A	0.66	0.69
1q32.1	rs4951291	rs4951291	15835 vs. 16724(9*)	C	0.87	0.85
**Lipids metabolism**						
SCD		rs7849	2011 vs. 2580(3)	A	0.83	0.82
LIPC	N215S	rs6083	7667 vs. 7980(4*)	G	0.64	0.63
ADIPOQ	45 T>G	rs2241766	1517 vs. 1909 (5)	T	0.75	0.77
ADIPOQ	276 G>T	rs1501299	2221 vs. 3035 (8)	G	0.71	0.69
ADIPOQ	11377C>G	rs266729	2173 vs. 2582 (5)	C	0.72	0.71
ADIPOR1	+5843 G>A	rs1342387	2472 vs. 2848 (5*)	G	0.59	0.56
ADIPOR1		rs7539542	1098 vs. 1301 (3)	G	0.45	0.43
**Blood clotting**						
SERPINE1		rs1799889	2241 vs. 2735(3)	4G	0.56	0.55

**Hormone metabolism**						
GH1	1663T>A	rs2665802	3275 vs. 3848(7)	T	0.57	0.55
PGR	V660L	rs1042838	5232 vs. 5733(4*)	C	0.83	0.84
ESR1	351A>G	rs9340799	5043 vs. 6090(7)	A	0.65	0.64
ESR1	397 T>C	rs2234693	2792 vs. 3288(6)	T	0.55	0.53
ESR1		rs2077647	2594 vs. 3086(4)	T	0.53	0.53
ESR1		rs3798577	2593 vs. 3100(4)	T	0.54	0.54
ESR2		rs928554	2574 vs. 2977(3)	T	0.57	0.59
SHBG	D356N	rs6259	6312 vs. 6820(5*)	G	0.88	0.84
**Calcium sensing receptor**						
CASR	A986S	rs1801725	6000 vs. 6425 (4*)	G	0.87	0.87
CASR	R990G	rs1042636	9265 vs. 10905 (5*)	A	0.92	0.91
**Leptin coding gene and receptor**						
LEP	G19A	rs2167270	1839 vs. 2429(3)	G	0.63	0.62
LEP	G2548A	rs7799039	2324 vs. 2776(3)	G	0.57	0.56
LEPR	Q223R	rs1137101	4059 vs. 4225(4*)	A	0.55	0.55
**G-protein signaling_RhoB regulation**						
RHPN2		rs7259371	15762 vs. 16700(9*)	A	0.84	0.82
**Transmembrane transporter**						
SLC19A1	80G>A	rs1051266	4289 vs. 5817(6)	G	0.54	0.55
SLC22A4	979C>T	rs1050152	3820 vs. 4749(5*)	C	0.51	0.52
**Transcription factor**						
TCF7L2	47833C>T	rs7903146	5059 vs. 21289(6*)	C	0.75	0.74
HIF1A	1772 C>T	rs11549465	6036 vs. 8066(5*)	C	0.91	0.91
**Nucleic acid binding and ATPase activity**						
BLM	ASH	x	1741 vs. 11043 (3)	wild	0.99	1.00
**Others**						
15q13.3		rs4779584	6994 vs. 7280(7*)	C	0.56	0.57
		rs4951039	13791 vs. 14288(7*)	A	0.86	0.85

a) ref allele frequency taken from 1000 genomes data
* Includes GWAS data from SOCCS
§ Includes GWAS data from Ontario
** Tomlinson 2008 was based on 10 samples

Supplementary Table S2. The Bayesian false-discovery probabilities (BFDP) for allelic model.

			ALLELIC MODEL										
				Effect size		Heterogeneity		BFDP					
**Gene**	**Variant**	**Cases vs. controls (number of samples)**	**N**	**OR (95% CI)**	***P* value**	**I^2 (95% CI)**	***P value***	**0.05**	**0.01**	**1.00E-03**	**1.00E-04**	**1.00E-06**	**1.00E-07**
11q23.1	rs3802842	22320 vs. 22965 (20*)	20	1.15 (1.11, 1.20)	5.21E-12	43 (0, 78)	0.024	2.30E-07	1.20E-06	1.21E-05	1.21E-04	0.01	0.11
8q24	rs10505477	18562 vs. 20132 (14)	14	1.14 (1.10, 1.19)	2.04E-11	44 (0, 82)	0.040	3.71E-06	1.94E-05	1.95E-04	1.95E-03	0.16	0.66
	rs355527	13539 vs. 14375(9)	9	1.12(1.08,1.17)	1.65E-10	0(0,0)	0.977	4.97E-04	2.59E-03	0.03	0.21	0.96	1.00
DNMT3B	rs1569686	1224 vs. 1381(5)	4	0.57(0.47,0.68)	1.81E-09	0(0,0)	0.992	3.63E-06	1.89E-05	1.91E-04	1.91E-03	0.16	0.66
SMAD7	rs4464148	17772 vs. 17356 (9*)	9	1.12 (1.08, 1.16)	2.66E-09	12 (0, 92)	0.337	5.10E-07	2.66E-06	2.68E-05	2.68E-04	0.03	0.21
8q23.3	rs16892766	8351 vs. 8878 (6*)	6	1.24 (1.15, 1.34)	1.67E-08	4 (0, 92)	0.393	6.52E-05	3.40E-04	3.42E-03	0.03	0.77	0.97
	rs1862748	17436 vs. 18418(11)	11	0.91(0.88,0.94)	1.98E-08	0(0,63)	0.703	2.34E-05	1.22E-04	1.23E-03	0.01	0.55	0.92
MUTYH	rs34612342	28180 vs. 20923(19*)	17	1.89(1.47,2.42)	5.72E-07	0(0,62)	0.828	3.37E-03	0.02	0.15	0.64	0.99	1.00
SMAD7	rs12953717	28006 vs. 26539 (13*)	13	1.11 (1.07, 1.16)	6.24E-07	49 (0, 93)	0.024	3.00E-03	0.15	0.64	0.95	0.99	0.99
MUTYH	rs36053993	28302 vs. 20935(19*)	19	1.42(1.22,1.66)	4.89E-06	0(0,33)	0.703	0.01	0.06	0.38	0.86	1.00	1.00
20p12.3	rs961253	22971 vs. 25270 (14*)	14	1.11 (1.06, 1.16)	1.58E-05	60 (28, 91)	0.002	4.15E-03	0.02	0.18	0.69	1.00	1.00
	rs7259371	15762 vs. 16700(9*)	9	0.87(0.82,0.93)	1.65E-05	52(0,90)	0.034	0.03	0.15	0.64	0.95	1.00	1.00
8q24	rs6983267	51730 vs. 53589 (34*)	34	1.11 (1.06, 1.17)	1.75E-05	85 (77, 93)	1.93E-28	0.08	0.31	0.82	0.98	1.00	1.00
TERT	rs2736100	16176 vs. 18135(8)	8	1.07(1.04,1.10)	2.95E-05	0(0,82)	0.526	2.98E-03	0.02	0.14	0.61	0.99	1.00
10p14	rs10795668	16763 vs. 18302 (16*)	16	0.88 (0.83, 0.94)	4.35E-05	67 (34, 89)	0.000	0.09	0.35	0.85	0.98	1.00	1.00
PPAR-gamma	rs9858822	2152 vs. 2630 (5)	4	1.40 (1.19, 1.65)	4.69E-05	0 (0, 98)	0.697	0.05	0.21	0.72	0.96	1.00	1.00
NOD2	rs2066847	4573 vs. 3733(10)	10	1.39(1.16,1.66)	3.21E-04	0(0,71)	0.547	0.16	0.49	0.91	0.99	1.00	1.00
TGFB1	rs4803455	3747 vs. 4513 (3*)	3	1.11 (1.04, 1.18)	0.001	0 (0, 71)	0.903	0.35	0.74	0.97	1.00	1.00	1.00
VDR	rs11568820	4001 vs. 4682 (5§)	4	1.14 (1.05, 1.23)	0.001	0 (0, 92)	0.648	0.29	0.68	0.96	1.00	1.00	1.00
APC	rs1801155	2389 vs. 4223 (5)	5	1.60 (1.21, 2.11)	0.001	27 (0, 94)	0.244	0.30	0.96	1.00	1.00	1.00	1.00
9p24	rs719725	13513 vs. 14999 (14)	14	1.07 (1.02, 1.11)	0.002	24 (0, 79)	0.190	0.25	0.63	0.94	0.99	1.00	1.00
ERCC5	rs17655	9653 vs. 11367(14)	13	1.07(1.02,1.12)	0.003	0(0,73)	0.626	0.73	0.93	0.99	1.00	1.00	1.00
miR	rs895819	1322 vs. 1641(4)	4	1.18(1.06,1.32)	0.003	4(0,95)	0.373	0.58	0.99	1.00	1.00	1.00	1.00
NAT2	rs1799929	1861 vs. 1952 (5§)	5	0.84 (0.74, 0.95)	0.004	20 (0, 94)	0.289	0.19	0.93	0.99	1.00	1.00	1.00
MMP1	rs1799750	1660 vs. 2024(10)	9	0.78(0.66,0.93)	0.005	59(8,88)	0.012	0.65	0.91	0.99	1.00	1.00	1.00
NQ01	rs1800566	11183 vs. 12525 (16*)	16	1.17 (1.05, 1.30)	0.006	80 (66, 94)	1.17E-09	0.58	0.88	0.99	1.00	1.00	1.00
CHEK2	rs1787996	1687 vs. 3370 (3)	3	1.47 (1.12, 1.92)	0.006	0 (0, 98)	0.499	0.68	0.92	0.99	1.00	1.00	1.00
CYP1A1	rs1048943	9661 vs. 11774 (19§)	19	1.28 (1.07, 1.52)	0.006	84 (83, 96)	1.15E-15	0.63	0.99	0.99	1.00	1.00	1.00
XRCC1	rs25487	13017 vs. 18166(37)	37	1.11(1.03,1.19)	0.008	71(68,90)	8.64E-12	0.55	0.99	1.00	1.00	1.00	1.00
MTHFR	rs1801133	43652 vs. 60883(94§**)	91	0.96(0.93,0.99)	0.009	53(59,85)	3.61E-09	0.69	0.99	1.00	1.00	1.00	1.00
VDR	rs1544410	14789 vs. 15922 (16*)	16	0.83(0.72, 0.96)	0.011	93 (91, 95)	5.59E-40	0.78	0.95	0.99	1.00	1.00	1.00
MSH3	rs26779	6050 vs. 8024(6*)	6	1.07(1.02,1.14)	0.013	11(0,92)	0.343	0.94	0.99	1.00	1.00	1.00	1.00
TP73	G4C14	858 vs. 1173(4)	4	1.21(1.04,1.40)	0.013	4(0,92)	0.371	0.75	0.94	0.99	1.00	1.00	1.00
ADIPOR1	rs1342387	2472 vs. 2848 (5*)	5	0.87 (0.78, 0.98)	0.018	48 (0, 95)	0.105	0.86	0.97	1.00	1.00	1.00	1.00
CCND1	rs17852153	6500 vs. 8885(20)	20	1.10(1.02,1.19)	0.019	58(45,92)	0.001	0.87	0.97	1.00	1.00	1.00	1.00
	rs4951291	15835 vs. 16724(9*)	9	0.91(0.84,0.99)	0.021	68(22,88)	0.002	0.90	0.98	1.00	1.00	1.00	1.00
MLH1	rs63750447	937 vs. 812 (4)	3	2.14 (1.12, 4.10)	0.022	39 (0, 99)	0.194	0.91	0.98	1.00	1.00	1.00	1.00
ESR2	rs928554	2574 vs. 2977(3)	3	1.09(1.01,1.18)	0.025	0(0,89)	0.743	0.92	0.98	1.00	1.00	1.00	1.00
SCD	rs7849	2011 vs. 2580(3)	3	0.85(0.73,0.98)	0.025	29(0,98)	0.247	0.86	0.97	1.00	1.00	1.00	1.00
GH1	rs2665802	3275 vs. 3848(7)	7	0.89(0.80,0.99)	0.025	49(0,97)	0.069	0.90	0.98	1.00	1.00	1.00	1.00
MTHFD1	rs1950902	9059 vs. 11358(7*)	7	0.94(0.89,0.99)	0.026	3(0,82)	0.400	0.91	0.98	1.00	1.00	1.00	1.00
NOD2	rs2066844	3297 vs. 3088(9)	9	1.38(1.04,1.84)	0.026	39(0,91)	0.109	0.87	0.97	1.00	1.00	1.00	1.00
ARLTS1	rs3803185	2281 vs. 3196 (5)	5	1.09 (1.01, 1.18)	0.026	0 (0, 76)	0.803	0.92	0.98	1.00	1.00	1.00	1.00
ABCB1 (MDR1)	rs1128503	246 vs. 399 (3)	3	0.70 (0.51, 0.96)	0.027	45 (0, 98)	0.164	0.87	0.97	1.00	1.00	1.00	1.00
ADIPOQ	rs2241766	1517 vs. 1909 (5)	5	1.17 (1.01, 1.35)	0.030	29 (0, 97)	0.231	0.88	0.97	1.00	1.00	1.00	1.00
RAD18	rs373572	6560 vs. 6906(5*)	5	1.15(1.01,1.30)	0.031	75(33,98)	0.003	0.87	0.97	1.00	1.00	1.00	1.00

PGR	rs1042838	5232 vs. 5733(4*)	4	1.08(1.01,1.16)	0.034	0(0,95)	0.507	0.93	0.99	1.00	1.00	1.00	1.00
PTGS2/COX2	rs20417	7785 vs. 11371(18)	17	1.10(1.00,1.20)	0.035	37(0,83)	0.062	0.87	1.00	1.00	1.00	1.00	1.00
TGFB1	rs1800469	4021 vs. 6024(10)	10	0.89(0.80,0.99)	0.036	53(2,92)	0.023	0.75	0.99	0.99	1.00	1.00	1.00
HFE	rs1800562	6547 vs. 34156(10*)	10	1.10(1.01,1.20)	0.040	0(0,40)	0.850	0.91	0.98	1.00	1.00	1.00	1.00
AXIN2	rs2240308	4723 vs. 4932 (4*)	4	1.06 (1.00, 1.12)	0.040	0 (0, 92)	0.397	0.94	0.99	1.00	1.00	1.00	1.00
CHEK2	1100delC	2417 vs. 3615 (4)	4	1.66 (1.01, 2.73)	0.048	0 (0, 83)	0.835	0.92	0.98	1.00	1.00	1.00	1.00
MSH3	rs184967	8151 vs. 10103(4*)	4	1.08(1.00,1.16)	0.049	35(0,98)	0.204	0.93	0.99	1.00	1.00	1.00	1.00

* Includes GWAS data from SOCCS
§ Includes GWAS data from Ontario
** Tomlinson 2008 was based on 10 samples

Supplementary Table S3. The Bayesian false-discovery probabilities (BFDP) for dominant model.

			**DOMINANT MODEL**										
				**Effect size**		**Heterogeneity**		**BFDP**					
**Gene**	**Variant**	**Cases vs. controls (number of samples)**	**N**	**OR (95% CI)**	***P* value**	**I^2 (95% CI)**	***P value***	**0.05**	**0.01**	**1.00E-03**	**1.00E-04**	**1.00E-06**	**1.00E-07**
11q23.1	rs3802842	22320 vs. 22965 (20*)	20	1.19 (1.13, 1.25)	7.22E-12	33 (0, 70)	0.075	8.26E-09	4.31E-08	4.34E-07	4.35E-06	4.35E-04	4.33E-03
8q24	rs10505477	18562 vs. 20132 (14)	14	1.24 (1.17, 1,32)	1.04E-11	36 (0, 81)	0.084	2.82E-08	1.47E-07	1.48E-06	1.48E-05	1.48E-03	0.01
SMAD7	rs4464148	17772 vs. 17356 (9*)	9	1.16 (1.11, 1.21)	2.09E-10	2 (0, 86)	0.416	1.14E-08	5.92E-08	5.98E-07	5.98E-06	5.98E-04	0.01
DNMT3B	rs1569686	1224 vs. 1381(5)	5	0.46(0.36,0.59)	2.40E-09	30(0,95)	0.223	4.60E-05	2.40E-04	2.41E-03	0.02	0.71	0.96
	rs355527	13539 vs. 14375(9)	9	1.16(1.10,1.21)	2.43E-09	0(0,9)	0.966	1.14E-08	5.92E-08	5.98E-07	5.98E-06	5.98E-04	0.01
8q23.3	rs16892766	8351 vs. 8878 (6*)	6	1.27 (1.17, 1.38)	4.00E-09	3 (0, 90)	0.400	2.23E-05	1.16E-04	1.17E-03	0.01	0.54	0.92
TERT	rs2736100	16176 vs. 18135(8)	8	1.13(1.08,1.20)	2.03E-06	0(0,64)	0.788	0.05	0.22	0.74	0.97	1.00	1.00
	rs7259371	15762 vs. 16700(9*)	9	0.86(0.80,0.92)	3.81E-06	42(0,87)	0.084	0.01	0.05	0.35	0.84	1.00	1.00
	rs1862748	17436 vs. 18418(11)	11	0.91(0.87,0.95)	6.31E-06	0(0,70)	0.511	0.02	0.09	0.51	0.91	1.00	1.00
MUTYH	rs34612342	28180 vs. 20923(19*)	17	1.80(1.39,2.31)	6.42E-06	0(0,52)	0.884	0.02	0.08	0.45	0.89	1.00	1.00
8q24	rs6983267	51730 vs. 53589 (34*)	34	1.16 (1.08, 1.24)	2.98E-05	80 (69, 92)	0.000	0.01	0.05	0.37	0.85	1.00	1.00
20p12.3	rs961253	22971 vs. 25270 (14*)	14	1.14 (1.07, 1.21)	4.75E-05	57 (21, 88)	4.00E-03	0.01	0.07	0.44	0.89	1.00	1.00
SMAD7	rs12953717	28006 vs. 26539 (13*)	13	1.14 (1.07, 1.21)	5.00E-05	54 (10, 95)	0.020	0.01	0.42	0.88	0.99	1.00	1.00
10p14	rs10795668	16763 vs. 18302 (16*)	16	0.85 (0.79, 0.93)	2.27E-04	68 (34, 87)	3.06E-05	0.18	0.54	0.92	0.99	1.00	1.00
PPAR-gamma	rs9858822	2152 vs. 2630 (5)	4	1.47 (1.19, 1.83)	4.55E-04	0 (0, 98)	0.618	0.29	0.68	0.95	1.00	1.00	1.00
GSTM1	Null variant	28240 vs. 38880 (74)	74	1.10 (1.05, 1.16)	0.001	47 (34, 75)	6.63E-06	0.19	0.93	0.99	0.99	1.00	1.00
MUTYH	rs36053993	28302 vs. 20935(19*)	19	1.31(1.12,1.53)	6.27E-04	0(0,24)	0.837	0.25	0.64	0.95	0.99	1.00	1.00
NOD2	rs2066847	4573 vs. 3733(10)	10	1.38(1.14,1.66)	7.22E-04	0(0,74)	0.488	0.27	0.65	0.95	0.99	1.00	1.00
ABCB1 (MDR1)	rs1128503	246 vs. 399 (3)	3	0.52 (0.35, 0.76)	8.66E-04	0 (0, 96)	0.617	0.57	0.87	0.99	1.00	1.00	1.00
TGFB1	rs4803455	3747 vs. 4513 (3*)	3	1.18 (1.06, 1.30)	0.001	0 (0, 85)	0.879	0.29	0.68	0.96	1.00	1.00	1.00
APC	rs1801155	2389 vs. 4223 (5)	5	1.62 (1.21, 2.16)	0.001	28 (0, 94)	2.35E-01	0.32	0.96	1.00	1.00	1.00	1.00
XPC	rs2228000	2677 vs. 4253(6)	6	0.58(0.41,0.82)	0.002	92(83,99)	0.000	0.65	0.91	0.99	1.00	1.00	1.00
MMP1	rs1799750	1660 vs. 2024(10)	10	0.70(0.56,0.88)	0.002	57(9,88)	0.013	0.53	0.85	0.98	1.00	1.00	1.00
ADIPOQ	rs2241766	1517 vs. 1909 (5)	5	1.26 (1.09, 1.47)	0.002	8 (0, 96)	0.364	0.55	0.86	0.98	1.00	1.00	1.00
CCND1	rs17852153	6500 vs. 8885(20)	20	1.15(1.05,1.26)	0.003	18(0,76)	0.226	0.55	0.86	0.98	1.00	1.00	1.00
ERCC5	rs17655	9653 vs. 11367(14)	14	1.14(1.04,1.24)	0.004	44(0,85)	3.82E-02	0.52	0.85	0.98	1.00	1.00	1.00
VDR	rs11568820	4001 vs. 4682 (5§)	5	1.14 (1.04, 1.24)	0.004	0 (0, 0)	0.989	0.52	0.85	0.98	1.00	1.00	1.00
CHEK2	rs1787996	1687 vs. 3370 (3)	3	1.48 (1.13, 1.95)	0.005	0 (0, 98)	0.496	0.70	0.93	0.99	1.00	1.00	1.00
NQ01	rs1800566	11183 vs. 12525 (16*)	16	1.20 (1.06, 1.37)	0.006	78 (64, 94)	0.000	0.70	0.92	0.99	1.00	1.00	1.00
9p24	rs719725	13513 vs. 14999 (14)	14	1.09 (1.02, 1.17)	0.008	0 (0, 66)	5.21E-01	0.87	0.97	1.00	1.00	1.00	1.00
CYP1A1	rs1048943	9661 vs. 11774 (19§)	19	1.36 (1.08, 1.72)	0.009	88 (93, 98)	0.000	0.75	0.99	1.00	1.00	1.00	1.00
PGR	rs1042838	5232 vs. 5733(4*)	4	1.11(1.02,1.21)	0.012	0(0,96)	0.450	0.86	0.97	1.00	1.00	1.00	1.00
CYP2C9	rs1799853	9588 vs. 11428 (12)	12	0.93 (0.87, 0.99)	0.015	0 (0, 72)	0.452	0.91	0.98	1.00	1.00	1.00	1.00
VDR	rs1544410	14789 vs. 15922 (16*)	16	0.82 (0.69, 0.96)	0.015	88 (83, 91)	0.000	0.79	0.95	0.99	1.00	1.00	1.00
MTHFD1	rs1950902	9059 vs. 11358(7*)	7	0.92(0.86,0.98)	0.015	15(0,90)	3.13E-01	0.82	0.96	1.00	1.00	1.00	1.00
NAT2	rs1799929	1861 vs. 1952 (5§)	5	0.85 (0.74, 0.97)	0.016	0 (0, 85)	0.872	0.51	0.98	1.00	1.00	1.00	1.00
ABCB1 (MDR1)	rs9282564	9536 vs. 9145 (6*)	6	1.09 (1.01, 1.17)	0.018	0 (0, 70)	0.729	0.87	0.97	1.00	1.00	1.00	1.00
MLH1	rs63750447	937 vs. 812 (4)	3	2.17 (1.14, 4.14)	0.019	36 (0, 98)	0.208	0.91	0.98	1.00	1.00	1.00	1.00
miR	rs2292832	2355 vs. 2571(5*)	5	0.87(0.78,0.98)	0.021	0(0,88)	0.704	0.86	0.97	1.00	1.00	1.00	1.00
XRCC1	rs25487	13017 vs. 18166(37)	37	1.11(1.01,1.21)	0.023	63(58,89)	0.000	0.82	1.00	1.00	1.00	1.00	1.00
MSH3	rs26779	6050 vs. 8024(6*)	6	1.08(1.01,1.15)	0.030	0(0,85)	0.682	0.88	0.97	1.00	1.00	1.00	1.00
EGF	rs4444903	899 vs. 976(5)	5	0.65(0.44,0.96)	0.030	67(12,97)	0.016	0.89	0.98	1.00	1.00	1.00	1.00
SERPINE1	rs1799889	2241 vs. 2735(3)	3	0.87(0.77,0.99)	0.030	0(0,98)	0.372	0.89	0.98	1.00	1.00	1.00	1.00
NOD2	rs2066844	3297 vs. 3088(9)	9	1.35(1.02,1.78)	0.033	33(0,90)	0.155	0.88	0.97	1.00	1.00	1.00	1.00
HFE	rs1800562	6547 vs. 34156(10*)	10	1.10(1.01,1.21)	0.037	0(0,25)	0.937	0.93	0.99	1.00	1.00	1.00	1.00
AXIN2	rs2240308	4723 vs. 4932 (4*)	4	1.10 (1.01, 1.21)	0.037	0 (0, 76)	0.758	0.93	0.99	1.00	1.00	1.00	1.00

ESR2	rs928554	2574 vs. 2977(3)	3	1.13(1.01,1.26)	0.041	0(0,89)	0.876	0.88	0.98	1.00	1.00	1.00	1.00
ABCB1 (MDR1)	rs1202168	7000 vs. 6485 (5)	5	1.12 (1.00, 1.24)	0.042	52 (0, 93)	0.080	0.89	0.98	1.00	1.00	1.00	1.00
NAT2	rs1799930	6446 vs. 7193 (8§)	8	1.13 (1.00, 1.27)	0.043	51 (0, 97)	0.045	0.88	1.00	1.00	1.00	1.00	1.00
CHEK2	1100delC	2417 vs. 3615 (4)	4	1.66 (1.01, 2.75)	0.047	0 (0, 83)	8.35E-01	0.92	0.98	1.00	1.00	1.00	1.00

* Includes GWAS data from SOCCS
§ Includes GWAS data from Ontario
** Tomlinson 2008 was based on 10 samples

Supplementary Table S4. The Bayesian false-discovery probabilities (BFDP) for recessive model.

			RECESSIVE MODEL										
				Effect size		Heterogeneity		BFDP					
**Gene**	**Variant**	**Cases vs. controls (number of samples)**	**N**	**OR (95% CI)**	***P* value**	**I^2 (95% CI)**	***P value***	**0.05**	**0.01**	**1.00E-03**	**1.00E-04**	**1.00E-06**	**1.00E-07**
8q24	rs10505477	18562 vs. 20132 (14)	14	1.16 (1.10, 1.23)	4.06E-07	35 (0, 74)	0.092	7.59E-04	3.94E-03	0.04	0.29	0.98	1.00
10p14	rs10795668	16763 vs. 18302 (16*)	16	0.83 (0.77, 0.89)	4.77E-07	11 (0, 70)	0.332	1.88E-04	9.81E-04	0.01	0.09	0.91	0.99
	rs1862748	17436 vs. 18418(11)	11	0.83(0.77,0.90)	1.59E-06	2(0,68)	0.427	0.01	0.03	0.22	0.74	1.00	1.00
11q23.1	rs3802842	22320 vs. 22965 (20*)	20	1.24 (1.13, 1.35)	3.62E-06	45 (5, 86)	0.017	7.04E-04	3.66E-03	0.04	0.27	0.97	1.00
	rs355527	13539 vs. 14375(9)	9	1.18(1.09,1.27)	3.54E-05	0(0,94)	0.458	0.01	0.04	0.31	0.82	1.00	1.00
8q24	rs6983267	51730 vs. 53589 (34*)	34	1.16 (1.08, 1.25)	4.92E-05	79 (66, 89)	2.80E-18	0.06	0.26	0.78	0.97	1.00	1.00
miR	rs895819	1322 vs. 1641(4)	4	1.50(1.23,1.83)	6.32E-05	1(0,96)	0.385	0.04	0.70	0.96	1.00	1.00	1.00
MTHFR	rs1801133	43652 vs. 60883(94§**)	93	0.87(0.81,0.93)	7.36E-05	56(63,86)	6.79E-11	0.00	0.16	0.65	0.95	1.00	1.00
SMAD7	rs12953717	28006 vs. 26539 (13*)	13	1.17 (1.08, 1.26)	8.34E-05	45 (0, 94)	0.041	0.02	0.57	0.93	0.99	1.00	1.00
	rs4951291	15835 vs. 16724(9*)	9	0.74(0.62,0.87)	2.72E-04	0(0,69)	0.616	0.14	0.46	0.90	0.99	1.00	1.00
20p12.3	rs961253	22971 vs. 25270 (14*)	13	1.14 (1.06, 1.22)	0.001	36 (0, 96)	0.098	0.10	0.36	0.85	0.98	1.00	1.00
GSTT1	Null variant	19133 vs. 27821 (56)	53	1.17 (1.07, 1.28)	0.001	70 (71, 90)	1.62E-15	0.24	0.94	0.99	0.99	1.00	1.00
TP73	G4C14	858 vs. 1173(4)	4	1.75(1.23,2.48)	0.002	0(0,94)	0.513	0.63	0.90	0.99	1.00	1.00	1.00
PPAR-gamma	rs9858822	2152 vs. 2630 (5)	2	1.69 (1.19, 2.39)	0.003	0 (0, 98)	0.801	0.70	0.92	0.99	1.00	1.00	1.00
IGF1	rs35767	2792 vs. 4988 (3)	3	0.75 (0.62, 0.91)	0.003	0 (0, 97)	0.567	0.58	0.88	0.99	1.00	1.00	1.00
TGFB1	rs1800469	4021 vs. 6024(10)	10	0.84 (0.74,0.94)	0.003	0(0,75)	0.527	0.19	0.93	0.99	1.00	1.00	1.00
	rs4951039	13791 vs. 14288(7*)	7	0.78(0.66,0.93)	0.005	0(0,68)	0.620	0.65	0.91	0.99	1.00	1.00	1.00
9p24	rs719725	13513 vs. 14999 (14)	14	1.09 (1.02, 1.15)	0.006	29 (0, 75)	0.148	0.52	0.85	0.98	1.00	1.00	1.00
MSH2	rs4608577	4308 vs. 4011(3*)	3	0.72(0.56,0.92)	0.008	0(0,95)	0.648	0.75	0.94	0.99	1.00	1.00	1.00
MTHFR	rs1801131	23523 vs. 35193(57§)	57	0.91(0.84, 0.98)	0.010	23(0,62)	0.064	0.68	0.99	0.99	1.00	1.00	1.00
PARP1	rs1136410	7002 vs. 8328(7*)	7	1.23(1.05,1.44)	0.012	0(0,92)	0.475	0.75	0.94	0.99	1.00	1.00	1.00
ABCB1 (MDR1)	rs1045642	7537 vs. 8396 (16)	16	0.89 (0.81, 0.97)	0.012	18 (0, 78)	0.248	0.76	0.94	0.99	1.00	1.00	1.00
ADIPOR1	rs1342387	2472 vs. 2848 (5*)	5	0.84 (0.73, 0.96)	0.012	0 (0, 62)	0.900	0.76	0.94	0.99	1.00	1.00	1.00
CRP	rs1800947	2853 vs. 3381(3)	2	3.95(1.35,11.56)	0.012	7(0,99)	0.301	0.93	0.99	1.00	1.00	1.00	1.00
CYP1A1	rs1048943	9661 vs. 11774 (19§)	18	1.26 (1.04, 1.52)	0.016	0 (0, 55)	0.456	0.80	1.00	1.00	1.00	1.00	1.00
PTGS2/COX2	rs20417	7785 vs. 11371(18)	17	1.24(1.04,1.47)	0.016	0(0,46)	0.928	0.78	0.99	1.00	1.00	1.00	1.00
MUTYH	rs34612342	28180 vs. 20923(19*)	7	3.40(1.22,9.48)	0.019	0(0,0)	0.998	0.93	0.99	1.00	1.00	1.00	1.00
MTRR	rs10380	9022 vs. 10910 (7*)	6	1.38 (1.04, 1.82)	0.025	0 (0, 90)	0.560	0.85	0.97	1.00	1.00	1.00	1.00
XRCC1	rs25487	13017 vs. 18166(37)	36	1.17(1.02,1.34)	0.025	59(39,80)	3.14E-06	0.85	1.00	1.00	1.00	1.00	1.00
	rs7259371	15762 vs. 16700(9*)	9	0.85(0.73,0.98)	0.027	17(0,86)	0.295	0.86	0.97	1.00	1.00	1.00	1.00
TGFB1	rs4803455	3747 vs. 4513 (3*)	3	1.12 (1.01, 1.24)	0.027	0 (0, 80)	0.845	0.89	0.98	1.00	1.00	1.00	1.00
CRP	rs1205	3037 vs. 9333(4)	4	1.18(1.02,1.37)	0.027	0(0,94)	0.431	0.87	0.97	1.00	1.00	1.00	1.00
GH1	rs2665802	3275 vs. 3848(7)	7	0.83(0.71,0.98)	0.028	37(0,95)	0.144	0.86	0.97	1.00	1.00	1.00	1.00
ADH1B	rs1229984	4449 vs. 6995(9)	9	1.23(1.02,1.48)	0.028	0(0,70)	0.493	0.86	1.00	1.00	1.00	1.00	1.00
VEGF	rs699947	4497 vs. 5334 (10*)	10	1.14 (1.01, 1.29)	0.029	14 (0, 89)	0.317	0.90	0.98	1.00	1.00	1.00	1.00
MSH3	rs184967	8151 vs. 10103(4*)	4	1.23(1.02,1.49)	0.031	0(0,91)	0.702	0.88	0.97	1.00	1.00	1.00	1.00
LIPC	rs6083	7667 vs. 7980(4*)	4	0.89(0.80,0.99)	0.033	22(0,94)	0.278	0.90	0.98	1.00	1.00	1.00	1.00
VDR	rs1544410	14789 vs. 15922 (16*)	16	0.81 (0.67, 0.98)	0.033	85 (77, 89)	1.08E-14	0.87	0.97	1.00	1.00	1.00	1.00
VDR	rs11568820	4001 vs. 4682 (5§)	4	1.37 (1.02, 1.85)	0.039	35 (0, 97)	0.203	0.89	0.98	1.00	1.00	1.00	1.00
MMP1	rs1799750	1660 vs. 2024(10)	9	0.78(0.61,0.99)	0.041	21(0,68)	0.254	0.89	0.98	1.00	1.00	1.00	1.00
RAD18	rs373572	6560 vs. 6906(5*)	5	1.23(1.01,1.49)	0.041	54(0,95)	0.067	0.88	0.97	1.00	1.00	1.00	1.00
NAT2	rs1799929	1861 vs. 1952 (5§)	5	0.68 (0.47, 0.99)	0.044	68(14, 98)	0.015	0.12	0.88	0.99	1.00	1.00	1.00
HPGD	rs8752	3968 vs. 4830(3*)	3	1.12(1.00,1.26)	0.047	6(0,97)	0.345	0.93	0.99	1.00	1.00	1.00	1.00

* Includes GWAS data from SOCCS
§ Includes GWAS data from Ontario
** Tomlinson 2008 was based on 10 samples

Supplementary Table S5. Validation test for allelic model with meta-GWAS of GECCO, CORECT and CCRF.

rsid	SNP_Name	Effect allele	Other allele	Direction	Meta-beta	Meta-se	Meta-pval	Meta-power	GECCO_beta	GECCO_se	GECCO_pval	GECCO_R2 Imputation	CORECT_beta	CORECT_se	CORECT_pval	CORECT_R2 Imputation
rs6983267	8:128413305_G/T	t	g	--	-0.139	0.012	1.14E-30	1.00	-0.130	0.018	1.36E-12	0.972	-0.145	0.016	9.64E-20	1.011
rs4939827	18:46453463_T/C	t	c	++	0.139	0.012	3.28E-30	0.95	0.117	0.018	1.39E-10	0.988	0.156	0.016	9.05E-22	1.047
rs10505477	8:128407443_A/G	a	g	++	0.136	0.012	1.61E-29	1.00	0.131	0.018	1.02E-12	0.959	0.140	0.016	2.10E-18	1.008
rs12953717	18:46453929_C/T	t	c	++	0.116	0.012	2.09E-21	1.00	0.110	0.018	2.35E-09	0.970	0.121	0.016	1.34E-13	1.036
rs16892766	8:117630683_A/C	a	c	--	-0.178	0.021	4.93E-17	1.00	-0.202	0.033	6.62E-10	0.983	-0.160	0.028	8.28E-09	1.031
rs3802842	11:111171709_C/A	a	c	--	-0.100	0.013	5.34E-14	1.00	-0.104	0.020	3.63E-07	0.948	-0.097	0.017	2.88E-08	1.011
rs4464148	18:46459032_T/C	t	c	--	-0.093	0.013	1.90E-12	1.00	-0.070	0.020	4.01E-04	0.961	-0.112	0.018	3.30E-10	1.063
rs355527	20:6388068_T/C	t	c	++	0.091	0.013	3.29E-12	1.00	0.088	0.019	4.28E-06	1.003	0.093	0.018	1.65E-07	1.037
rs1801155	5:112175211_T/A	a	t	++	0.577	0.088	4.84E-11	1.00	0.387	0.208	0.06291	0.653	0.618	0.097	1.69E-10	1.007
rs961253	20:6404281_C/A	a	c	++	0.079	0.013	6.08E-10	1.00	0.077	0.019	4.05E-05	0.988	0.080	0.017	3.62E-06	1.045
rs10795668	10:8701219_G/A	a	g	--	-0.066	0.013	2.63E-07	1.00	-0.033	0.019	0.087	0.993	-0.091	0.017	9.06E-08	1.007
rs2736100	5:1286516_C/A	a	c	++	0.062	0.012	3.23E-07	1.00	0.065	0.019	0.001	0.922	0.061	0.016	1.56E-04	1.008
rs7259371	19:33534641_G/A	a	g	--	-0.067	0.015	3.62E-06	1.00	-0.063	0.023	0.006	1.003	-0.070	0.019	1.77E-04	1.097
rs1862748	16:68832943_C/T	t	c	--	-0.049	0.013	1.75E-04	1.00	-0.065	0.020	0.001	0.992	-0.036	0.017	0.038	1.027
rs719725	9:6365683_A/C	a	c	++	0.043	0.012	4.25E-04	1.00	0.064	0.019	0.001	0.983	0.027	0.016	0.094	1.002
rs8752	4:175412477_C/T	t	c	--	-0.038	0.012	0.002	0.99	-0.020	0.018	0.280	0.983	-0.052	0.016	0.001	1.038
rs1800469	19:41860296_A/G	a	g	--	-0.039	0.013	0.002	1.00	-0.022	0.019	0.258	0.999	-0.052	0.017	0.002	1.033
rs7849	10:102122603_T/C	t	c	++	0.044	0.015	0.004	1.00	0.059	0.024	0.013	0.983	0.033	0.020	0.096	1.007
rs20417	1:186650321_C/G	c	g	--	-0.043	0.016	0.008	1.00	-0.049	0.024	0.045	0.988	-0.039	0.022	0.080	1.019
rs36053993	1:45797228_C/T	t	c	-+	0.198	0.088	0.024	1.00	-0.001	0.118	0.993	0.835	0.447	0.132	0.001	0.797
rs928554	14:64694195_C/T	t	c	--	-0.026	0.012	0.029	1.00	-0.026	0.018	0.148	0.995	-0.026	0.016	0.103	1.005
rs4803455	19:41851509_C/A	a	c	++	0.027	0.013	0.031	1.00	0.035	0.020	0.079	0.816	0.022	0.016	0.178	1.002
rs11568820	12:48302545_C/T	t	c	+-	0.031	0.015	0.037	1.00	0.080	0.023	0.000	0.967	-0.004	0.019	0.840	1.039
rs10380	5:7897191_C/T	t	c	--	-0.035	0.019	0.068	0.74	-0.024	0.030	0.429	0.997	-0.043	0.025	0.086	0.990
rs2665802	17:61995030_A/T	a	t	++	0.021	0.013	0.090	1.00	0.020	0.019	0.307	0.878	0.022	0.016	0.174	0.972
rs1042838	11:100933412_C/A	a	c	--	-0.027	0.017	0.108	0.99	-0.006	0.025	0.822	1.000	-0.044	0.022	0.049	1.033
rs9344	11:69462910_G/A	a	g	++	0.021	0.013	0.116	1.00	0.043	0.021	0.035	0.770	0.005	0.017	0.769	0.940
rs1799853	10:96702047_C/T	t	c	++	0.028	0.019	0.128	0.89	0.019	0.027	0.469	0.997	0.036	0.026	0.157	0.983
rs1048943	15:75012985_T/C	t	c	--	-0.043	0.029	0.133	1.00	-0.077	0.051	0.129	0.921	-0.027	0.034	0.429	1.075
rs1544410	12:48239835_C/T	t	c	--	-0.018	0.013	0.143	1.00	-0.017	0.018	0.354	0.998	-0.019	0.017	0.255	1.066
rs3803185	13:50205025_T/C	t	c	+-	-0.017	0.012	0.155	1.00	0.002	0.018	0.900	0.974	-0.033	0.016	0.042	1.051
rs2066844	16:50745926_C/T	t	c	++	0.044	0.032	0.168	1.00	0.059	0.043	0.170	0.901	0.025	0.047	0.589	0.920
rs4444903	4:110834110_A/G	a	g	--	-0.017	0.012	0.175	1.00	-0.003	0.019	0.889	0.973	-0.028	0.016	0.092	1.042
rs34612342*	1:45798475_T/C	t	c	?-	-1.395	1.074	0.194	1.00	-0.041	0.582	0.944	0.163	-1.395	1.074	0.194	0.097
rs1229984	4:100239319_T/C	t	c	--	-0.031	0.025	0.199	0.93	-0.076	0.050	0.131	0.815	-0.018	0.028	0.530	1.200
rs1342387	1:202914356_T/C	t	c	+-	-0.016	0.012	0.201	1.00	0.004	0.018	0.821	0.991	-0.031	0.016	0.055	0.958
rs1801133	1:11856378_G/A	a	g	--	-0.016	0.013	0.220	0.93	-0.003	0.021	0.889	0.826	-0.024	0.017	0.144	1.013
rs1205	1:159682233_C/T	t	c	++	0.014	0.013	0.254	0.99	0.020	0.019	0.292	1.002	0.010	0.017	0.553	1.041
rs4608577	2:47703984_T/G	t	g	-+	-0.016	0.016	0.310	0.98	-0.041	0.024	0.081	0.993	0.004	0.021	0.844	1.005
rs1801280	8:18257854_T/C	t	c	--	-0.012	0.012	0.330	0.14	-0.022	0.018	0.225	0.994	-0.003	0.017	0.844	1.094
rs1800947	1:159683438_C/G	c	g	--	-0.025	0.026	0.333	0.24	-0.001	0.042	0.989	0.766	-0.041	0.033	0.222	0.982
rs2241766	3:186570892_T/G	t	g	-+	0.017	0.019	0.381	1.00	-0.009	0.029	0.746	0.961	0.036	0.025	0.148	0.861
rs1128503	7:87179601_A/G	a	g	++	0.010	0.012	0.390	1.00	0.010	0.018	0.576	0.996	0.011	0.016	0.514	1.019
rs1569686	20:31367079_G/T	t	g	-+	-0.011	0.012	0.400	1.00	-0.024	0.018	0.197	0.999	0.001	0.017	0.965	1.113
rs1799929	8:18257994_C/T	t	c	+-	0.010	0.013	0.410	1.00	0.025	0.018	0.176	0.989	-0.002	0.017	0.891	1.087
rs1202168	7:87195962_G/A	a	g	++	0.010	0.012	0.420	0.99	0.014	0.018	0.434	0.988	0.006	0.017	0.704	0.977
rs1799930	8:18258103_G/A	a	g	-+	-0.010	0.013	0.433	0.99	-0.024	0.020	0.232	1.005	0.000	0.018	0.989	1.012
rs9282564	7:87229440_T/C	t	c	--	-0.015	0.019	0.436	0.95	-0.046	0.035	0.196	0.748	-0.002	0.023	0.933	1.145

rs373572	3:8955389_C/T	t	c	+-	0.010	0.013	0.437	1.00	0.043	0.020	0.033	0.996	-0.015	0.018	0.409	1.076
rs35767	12:102875569_A/G	a	g	+-	0.011	0.016	0.490	0.97	0.030	0.025	0.227	0.970	-0.002	0.020	0.919	1.027
rs2292832	2:241395503_T/C	t	c	--	-0.010	0.015	0.493	0.99	-0.015	0.020	0.457	0.922	-0.005	0.021	0.828	0.688
rs2240308	17:63554591_G/A	a	g	++	0.009	0.013	0.501	0.99	0.015	0.023	0.518	0.650	0.006	0.016	0.712	1.028
rs1799750	11:102670495_TC/T	t	c	+-	-0.008	0.013	0.502	1.00	0.014	0.019	0.480	0.885	-0.024	0.016	0.138	0.937
rs1800566	16:69745145_G/A	a	g	++	0.009	0.015	0.536	1.00	0.016	0.023	0.493	1.000	0.004	0.019	0.817	1.044
rs1800562	6:26093141_G/A	a	g	+-	-0.016	0.028	0.562	0.99	0.016	0.038	0.682	0.971	-0.051	0.040	0.202	1.022
rs909460	18:55254310_C/T	t	c	--	-0.088	0.165	0.596	1.00	-0.187	0.337	0.579	0.837	-0.056	0.189	0.767	0.815
rs25487	19:44055726_T/C	t	c	--	-0.007	0.013	0.598	1.00	-0.007	0.020	0.730	0.915	-0.007	0.017	0.690	1.008
rs26779	5:80059203_C/T	t	c	-+	-0.006	0.012	0.640	0.99	-0.029	0.018	0.115	1.001	0.013	0.016	0.440	0.998
rs184967	5:80149981_A/G	a	g	+-	-0.008	0.017	0.652	0.99	0.012	0.024	0.615	1.012	-0.026	0.023	0.270	1.015
rs6083	15:58838010_A/G	a	g	-+	0.006	0.014	0.667	0.93	-0.001	0.020	0.974	0.886	0.012	0.019	0.531	0.855
rs9858822	3:12411238_A/C	a	c	+-	0.055	0.128	0.668	1.00	0.278	0.234	0.235	0.916	-0.040	0.152	0.794	0.987
rs895819	19:13947292_T/C	t	c	++	0.005	0.013	0.696	1.00	0.004	0.020	0.851	0.921	0.006	0.018	0.723	0.951
rs699947	6:43736389_A/C	a	c	--	-0.004	0.012	0.744	1.00	-0.008	0.018	0.668	0.960	-0.001	0.017	0.961	0.971
rs4951291	1:204006538_T/C	t	c	+-	0.006	0.024	0.808	0.99	0.029	0.028	0.290	0.952	-0.058	0.045	0.204	0.851
rs1801131	1:11854476_T/G	t	g	--	-0.003	0.013	0.824	0.38	-0.003	0.020	0.880	0.969	-0.003	0.017	0.870	1.012
rs4951039	1:204006597_G/A	a	g	-+	-0.005	0.024	0.830	0.99	-0.028	0.028	0.311	0.951	0.057	0.045	0.212	0.853
rs1950902	14:64882380_A/G	a	g	+-	-0.003	0.016	0.844	0.98	0.004	0.024	0.870	0.985	-0.008	0.021	0.686	1.007
rs2228000	3:14199887_G/A	a	g	-+	0.002	0.014	0.871	0.13	-0.016	0.021	0.444	0.997	0.016	0.018	0.375	1.011
rs1136410	1:226555302_A/G	a	g	+-	-0.001	0.016	0.970	0.94	0.029	0.025	0.250	0.980	-0.020	0.020	0.321	1.055
rs17655	13:103528002_G/C	c	g	+-	0.000	0.014	0.977	0.99	0.026	0.022	0.226	1.004	-0.020	0.018	0.286	1.064
rs1045642	7:87138645_A/G	a	g	+-	0.000	0.012	0.987	0.99	0.015	0.018	0.410	0.978	-0.012	0.016	0.454	1.026

* rs34612342 was dropped due to low imputation quality.

Supplementary Table S6. Validation test for dominant model with meta-GWAS of GECCO, CORECT and CCRF.

rsid	SNP_Name	Effect allele	Other allele	Direction	Meta-beta	Meta-se	Meta-pval	Meta-power	GECCO_beta	GECCO_se	GECCO_pval	GECCO_R2 Imputation	CORECT_beta	CORECT_se	CORECT_pval	CORECT_R2 Imputation
rs4939827	18:46453463_T/C	t	c	++	0.181	0.019	8.84E-21	0.94	0.163	0.029	1.08E-08	0.988	0.196	0.026	9.62E-14	1.047
rs6983267	8:128413305_G/T	t	g	--	-0.165	0.019	7.17E-18	1.00	-0.140	0.029	1.48E-06	0.972	-0.184	0.026	4.78E-13	1.011
rs10505477	8:128407443_A/G	a	g	++	0.166	0.020	1.82E-17	1.00	0.154	0.030	2.35E-07	0.959	0.174	0.026	1.26E-11	1.008
rs16892766	8:117630683_A/C	a	c	--	-0.193	0.023	3.68E-17	0.93	-0.217	0.035	5.39E-10	0.983	-0.175	0.030	8.29E-09	1.031
rs12953717	18:46453929_C/T	t	c	++	0.142	0.019	1.75E-14	1.00	0.138	0.028	1.13E-06	0.970	0.144	0.024	3.10E-09	1.036
rs3802842	11:111171709_C/A	a	c	--	-0.118	0.017	4.86E-12	1.00	-0.118	0.026	7.50E-06	0.948	-0.119	0.023	1.43E-07	1.011
rs1801155	5:112175211_T/A	a	t	++	0.594	0.091	6.28E-11	1.00	0.420	0.215	0.051	0.653	0.631	0.100	2.94E-10	1.007
rs4464148	18:46459032_T/C	t	c	--	-0.099	0.018	1.73E-08	1.00	-0.086	0.026	0.001	0.961	-0.110	0.024	3.73E-06	1.063
rs355527	20:6388068_T/C	t	c	++	0.096	0.017	2.07E-08	1.00	0.088	0.026	0.001	1.003	0.103	0.023	8.04E-06	1.037
rs10795668	10:8701219_G/A	a	g	--	-0.089	0.017	1.52E-07	1.00	-0.049	0.026	0.053	0.993	-0.119	0.023	1.20E-07	1.007
rs961253	20:6404281_C/A	a	c	++	0.083	0.017	1.74E-06	1.00	0.069	0.026	0.008	0.988	0.095	0.023	5.22E-05	1.045
rs7259371	19:33534641_G/A	a	g	--	-0.077	0.018	1.71E-05	0.99	-0.072	0.027	0.008	1.003	-0.082	0.024	7.19E-04	1.097
rs2736100	5:1286516_C/A	a	c	++	0.086	0.020	1.72E-05	0.99	0.090	0.031	0.004	0.922	0.083	0.026	0.001	1.008
rs7849	10:102122603_T/C	t	c	++	0.056	0.018	0.002	1.00	0.067	0.027	0.015	0.983	0.048	0.024	0.040	1.007
rs719725	9:6365683_A/C	a	c	++	0.049	0.017	0.004	1.00	0.078	0.026	0.003	0.983	0.027	0.023	0.234	1.002
rs20417	1:186650321_C/G	c	g	--	-0.053	0.019	0.005	0.99	-0.065	0.028	0.021	0.988	-0.043	0.025	0.089	1.019
rs1862748	16:68832943_C/T	t	c	--	-0.048	0.017	0.005	1.00	-0.067	0.026	0.009	0.992	-0.032	0.023	0.155	1.027
rs1800469	19:41860296_A/G	a	g	--	-0.044	0.017	0.011	1.00	-0.015	0.026	0.574	0.999	-0.067	0.023	0.004	1.033
rs8752	4:175412477_C/T	t	c	--	-0.046	0.018	0.012	1.00	-0.007	0.027	0.810	0.983	-0.077	0.024	0.002	1.038
rs11568820	12:48302545_C/T	t	c	++	0.037	0.018	0.035	1.00	0.074	0.027	0.006	0.967	0.010	0.023	0.682	1.039
rs1799853	10:96702047_C/T	t	c	++	0.039	0.021	0.058	0.96	0.021	0.030	0.490	0.997	0.056	0.029	0.049	0.983
rs1544410	12:48239835_C/T	t	c	--	-0.033	0.018	0.067	1.00	-0.039	0.027	0.146	0.998	-0.028	0.024	0.251	1.066
rs10380	5:7897191_C/T	t	c	--	-0.039	0.021	0.069	0.99	-0.020	0.033	0.537	0.997	-0.051	0.028	0.063	0.990
rs1048943	15:75012985_T/C	t	c	--	-0.052	0.031	0.094	0.99	-0.083	0.052	0.112	0.921	-0.035	0.038	0.360	1.075
rs1042838	11:100933412_C/A	a	c	--	-0.031	0.019	0.101	0.15	-0.006	0.028	0.831	1.000	-0.053	0.026	0.042	1.033
rs36053993	1:45797228_C/T	t	c	-+	0.135	0.093	0.145	1.00	-0.061	0.123	0.621	0.835	0.392	0.141	0.005	0.797
rs3803185	13:50205025_T/C	t	c	+-	-0.028	0.020	0.170	0.99	0.010	0.029	0.742	0.974	-0.061	0.028	0.028	1.051
rs928554	14:64694195_C/T	t	c	--	-0.024	0.018	0.188	1.00	-0.028	0.027	0.312	0.995	-0.020	0.024	0.387	1.005
rs2066844	16:50745926_C/T	t	c	++	0.043	0.033	0.192	0.98	0.045	0.045	0.319	0.901	0.041	0.049	0.399	0.920
rs34612342*	1:45798475_T/C	t	c	?-	-1.394	1.075	0.195	0.82	-0.040	0.583	0.945	0.163	-1.394	1.075	0.195	0.097
rs9344	11:69462910_G/A	a	g	++	0.027	0.021	0.197	1.00	0.063	0.033	0.053	0.770	0.002	0.027	0.938	0.940
rs9282564	7:87229440_T/C	t	c	--	-0.031	0.025	0.214	0.58	-0.055	0.039	0.160	0.748	-0.015	0.032	0.652	1.145
rs1800947	1:159683438_C/G	c	g	--	-0.033	0.028	0.233	1.00	-0.007	0.045	0.878	0.766	-0.049	0.035	0.164	0.982
rs373572	3:8955389_C/T	t	c	+-	0.020	0.017	0.245	1.00	0.056	0.026	0.029	0.996	-0.010	0.023	0.673	1.076
rs4803455	19:41851509_C/A	a	c	+-	0.022	0.020	0.276	1.00	0.071	0.033	0.031	0.816	-0.007	0.025	0.771	1.002
rs1229984	4:100239319_T/C	t	c	-+	-0.032	0.030	0.292	1.00	-0.107	0.054	0.047	0.815	0.002	0.036	0.955	1.200
rs1202168	7:87195962_G/A	a	g	++	0.018	0.019	0.325	0.74	0.022	0.027	0.414	0.988	0.015	0.025	0.558	0.977
rs1045642	7:87138645_A/G	a	g	+-	0.019	0.020	0.326	1.00	0.045	0.029	0.123	0.978	-0.002	0.027	0.934	1.026
rs4444903	4:110834110_A/G	a	g	+-	-0.018	0.018	0.339	0.99	0.002	0.027	0.950	0.973	-0.034	0.025	0.176	1.042
rs1205	1:159682233_C/T	t	c	++	0.016	0.017	0.349	1.00	0.017	0.026	0.501	1.002	0.015	0.023	0.513	1.041
rs1128503	7:87179601_A/G	a	g	++	0.017	0.018	0.354	1.00	0.016	0.027	0.560	0.996	0.018	0.024	0.470	1.019
rs1799929	8:18257994_C/T	t	c	++	0.017	0.019	0.364	1.00	0.024	0.027	0.381	0.989	0.011	0.026	0.671	1.087
rs2240308	17:63554591_G/A	a	g	++	0.019	0.022	0.375	0.99	0.023	0.038	0.541	0.650	0.017	0.027	0.513	1.028
rs1136410	1:226555302_A/G	a	g	+-	0.015	0.019	0.405	1.00	0.039	0.028	0.163	0.980	-0.003	0.025	0.910	1.055
rs4608577	2:47703984_T/G	t	g	-+	-0.015	0.018	0.416	1.00	-0.040	0.027	0.138	0.993	0.006	0.024	0.812	1.005
rs1800562	6:26093141_G/A	a	g	+-	-0.023	0.029	0.426	0.44	0.010	0.040	0.796	0.971	-0.061	0.043	0.153	1.022
rs2241766	3:186570892_T/G	t	g	-+	0.016	0.021	0.446	0.33	-0.016	0.032	0.615	0.961	0.042	0.028	0.138	0.861
rs1801280	8:18257854_T/C	t	c	--	-0.014	0.019	0.461	0.21	-0.012	0.028	0.657	0.994	-0.015	0.026	0.553	1.094

rs1799750	11:102670495_TC/T	t	c	+-	-0.014	0.020	0.489	1.00	0.021	0.030	0.495	0.885	-0.040	0.026	0.131	0.937
rs1342387	1:202914356_T/C	t	c	+-	0.013	0.019	0.506	1.00	0.044	0.028	0.121	0.991	-0.014	0.026	0.596	0.958
rs1801133	1:11856378_G/A	a	g	--	-0.012	0.018	0.509	1.00	-0.001	0.028	0.979	0.826	-0.019	0.023	0.408	1.013
rs1800566	16:69745145_G/A	a	g	++	0.010	0.018	0.575	1.00	0.014	0.027	0.600	1.000	0.007	0.024	0.774	1.044
rs2292832	2:241395503_T/C	t	c	--	-0.010	0.019	0.601	0.88	-0.016	0.026	0.527	0.922	-0.003	0.027	0.927	0.688
rs2665802	17:61995030_A/T	a	t	--	-0.009	0.018	0.624	1.00	-0.015	0.028	0.607	0.878	-0.005	0.024	0.837	0.972
rs35767	12:102875569_A/G	a	g	+-	0.009	0.018	0.645	1.00	0.031	0.028	0.275	0.970	-0.008	0.024	0.745	1.027
rs9858822	3:12411238_A/C	a	c	+-	0.059	0.128	0.647	1.00	0.278	0.234	0.235	0.916	-0.035	0.153	0.820	0.987
rs909460	18:55254310_C/T	t	c	--	-0.074	0.169	0.661	1.00	-0.104	0.361	0.773	0.837	-0.066	0.191	0.731	0.815
rs1801131	1:11854476_T/G	t	g	--	-0.007	0.017	0.680	0.99	-0.007	0.026	0.779	0.969	-0.007	0.023	0.763	1.012
rs699947	6:43736389_A/C	a	c	+-	0.008	0.020	0.687	1.00	0.022	0.029	0.454	0.960	-0.005	0.028	0.870	0.971
rs6083	15:58838010_A/G	a	g	-+	0.008	0.020	0.690	1.00	-0.015	0.028	0.601	0.886	0.030	0.028	0.281	0.855
rs184967	5:80149981_A/G	a	g	+-	-0.008	0.019	0.694	0.99	0.023	0.028	0.412	1.012	-0.035	0.026	0.188	1.015
rs1799930	8:18258103_G/A	a	g	--	-0.006	0.017	0.711	0.07	-0.012	0.025	0.633	1.005	-0.002	0.023	0.944	1.012
rs895819	19:13947292_T/C	t	c	++	0.006	0.018	0.750	1.00	0.004	0.027	0.882	0.921	0.007	0.023	0.769	0.951
rs26779	5:80059203_C/T	t	c	-+	-0.005	0.018	0.765	0.99	-0.025	0.026	0.352	1.001	0.010	0.023	0.673	0.998
rs17655	13:103528002_G/C	c	g	+-	-0.004	0.017	0.803	0.16	0.023	0.026	0.383	1.004	-0.026	0.023	0.265	1.064
rs25487	19:44055726_T/C	t	c	++	0.004	0.018	0.817	1.00	0.001	0.027	0.966	0.915	0.006	0.023	0.791	1.008
rs2228000	3:14199887_G/A	a	g	-+	-0.003	0.017	0.873	0.77	-0.008	0.026	0.765	0.997	0.001	0.023	0.959	1.011
rs1950902	14:64882380_A/G	a	g	+-	-0.002	0.018	0.907	0.05	0.004	0.027	0.887	0.985	-0.007	0.024	0.779	1.007
rs1569686	20:31367079_G/T	t	g	-+	-0.001	0.018	0.972	0.57	-0.016	0.026	0.557	0.999	0.013	0.025	0.610	1.113
rs4951291	1:204006538_T/C	t	c	+-	0.001	0.026	0.972	1.00	0.026	0.031	0.401	0.952	-0.064	0.049	0.197	0.851
rs4951039	1:204006597_G/A	a	g	-+	0.000	0.026	0.996	0.99	-0.024	0.031	0.426555	0.951	0.063	0.049	0.203	0.853

* rs34612342 was dropped due to low imputation quality.

Supplementary Table S7. Validation test for recessive model with meta-GWAS of GECCO, CORECT and CCRF.

rsid	SNP_Name	Effect allele	Other allele	Direction	Meta-beta	Meta-se	Meta-pval	Meta-power	GECCO_beta	GECCO_se	GECCO_pval	GECCO_R2 Imputation	CORECT_beta	CORECT_se	CORECT_pval	CORECT_R2 Imputation
rs6983267	8:128413305_G/T	t	g	--	-0.207	0.020	1.04E-24	1.00	-0.214	0.031	5.96E-12	0.972	-0.202	0.026	2.52E-14	1.011
rs10505477	8:128407443_A/G	a	g	++	0.200	0.020	1.49E-23	1.00	0.204	0.031	4.27E-11	0.959	0.197	0.026	5.38E-14	1.008
rs4939827	18:46453463_T/C	t	c	++	0.188	0.020	1.84E-20	0.99	0.149	0.031	1.88E-06	0.988	0.216	0.027	4.69E-16	1.047
rs12953717	18:46453929_C/T	t	c	++	0.172	0.022	3.27E-15	1.00	0.161	0.032	7.47E-07	0.970	0.181	0.029	7.78E-10	1.036
rs4464148	18:46459032_T/C	t	c	--	-0.174	0.029	1.55E-09	1.00	-0.102	0.044	1.96E-02	0.961	-0.228	0.038	2.33E-09	1.063
rs355527	20:6388068_T/C	t	c	++	0.168	0.028	3.15E-09	1.00	0.177	0.041	1.32E-05	1.003	0.158	0.039	5.63E-05	1.037
rs961253	20:6404281_C/A	a	c	++	0.144	0.026	3.13E-08	1.00	0.170	0.038	8.83E-06	0.988	0.122	0.036	6.22E-04	1.045
rs3802842	11:111171709_C/A	a	c	--	-0.152	0.031	5.85E-07	1.00	-0.181	0.048	1.62E-04	0.948	-0.133	0.039	7.54E-04	1.011
rs2736100	5:1286516_C/A	a	c	++	0.082	0.020	4.37E-05	1.00	0.089	0.032	0.005	0.922	0.078	0.026	0.003	1.008
rs2665802	17:61995030_A/T	a	t	++	0.090	0.024	1.30E-04	0.41	0.094	0.036	0.010	0.878	0.087	0.031	0.005	0.972
rs1862748	16:68832943_C/T	t	c	--	-0.103	0.029	3.67E-04	1.00	-0.129	0.044	0.003	0.992	-0.083	0.038	0.030	1.027
rs719725	9:6365683_A/C	a	c	++	0.072	0.024	0.003	1.00	0.097	0.037	0.009	0.983	0.052	0.032	0.103	1.002
rs1342387	1:202914356_T/C	t	c	--	-0.061	0.021	0.003	1.00	-0.043	0.032	0.176	0.991	-0.075	0.028	0.007	0.958
rs7259371	19:33534641_G/A	a	g	--	-0.099	0.035	0.005	1.00	-0.089	0.066	0.179	1.003	-0.103	0.041	0.013	1.097
rs16892766	8:117630683_A/C	a	c	--	-0.233	0.089	0.009	1.00	-0.265	0.157	0.091	0.983	-0.217	0.108	0.044	1.031
rs8752	4:175412477_C/T	t	c	--	-0.056	0.022	0.010	0.74	-0.057	0.034	0.092	0.983	-0.056	0.029	0.051	1.038
rs10795668	10:8701219_G/A	a	g	--	-0.072	0.028	0.011	1.00	-0.023	0.043	0.600	0.993	-0.109	0.038	0.004	1.007
rs1800469	19:41860296_A/G	a	g	--	-0.067	0.027	0.012	1.00	-0.066	0.043	0.121	0.999	-0.068	0.034	0.047	1.033
rs4803455	19:41851509_C/A	a	c	++	0.053	0.021	0.013	1.00	0.026	0.034	0.447	0.816	0.069	0.027	0.010	1.002
rs928554	14:64694195_C/T	t	c	--	-0.053	0.022	0.018	1.00	-0.046	0.033	0.162	0.995	-0.058	0.030	0.053	1.005
rs1136410	1:226555302_A/G	a	g	--	-0.096	0.045	0.034	0.69	-0.023	0.084	0.783	0.980	-0.127	0.054	0.019	1.055
rs1801155	5:112175211_T/A	a	t	-+	1.149	0.629	0.068	1.00	-0.160	1.381	0.908	0.653	1.491	0.706	0.035	1.007
rs1801133	1:11856378_G/A	a	g	--	-0.041	0.027	0.124	0.43	-0.011	0.044	0.796	0.826	-0.058	0.033	0.083	1.013
rs1569686	20:31367079_G/T	t	g	--	-0.034	0.023	0.134	1.00	-0.059	0.035	0.094	0.999	-0.016	0.030	0.594	1.113
rs25487	19:44055726_T/C	t	c	--	-0.038	0.026	0.153	1.00	-0.031	0.040	0.440	0.915	-0.042	0.035	0.221	1.008
rs36053993	1:45797228_C/T	t	c	++	20.751	15.062	0.168	1.00	84.757	84.007	0.313	0.835	18.625	15.310	0.224	0.797
rs9344	11:69462910_G/A	a	g	++	0.030	0.023	0.188	1.00	0.058	0.036	0.110	0.770	0.012	0.029	0.677	0.940
rs4444903	4:110834110_A/G	a	g	--	-0.029	0.022	0.196	1.00	-0.012	0.035	0.732	0.973	-0.040	0.028	0.165	1.042
rs34612342*	1:45798475_T/C	t	c	?-	-1236	975.53	0.205	1.00	-40.662	170.70	0.812	0.163	-1235	975.536	0.205	0.097
rs1799930	8:18258103_G/A	a	g	-+	-0.035	0.030	0.253	1.00	-0.086	0.046	0.058	1.005	0.007	0.041	0.873	1.012
rs1229984	4:100239319_T/C	t	c	+-	-0.063	0.060	0.298	0.90	0.326	0.221	0.139	0.815	-0.094	0.062	0.133	1.200
rs1045642	7:87138645_A/G	a	g	--	-0.020	0.020	0.310	0.77	-0.008	0.031	0.806	0.978	-0.029	0.026	0.266	1.026
rs4608577	2:47703984_T/G	t	g	--	-0.048	0.050	0.333	0.91	-0.106	0.075	0.155	0.993	-0.002	0.066	0.975	1.005
rs3803185	13:50205025_T/C	t	c	--	-0.019	0.020	0.340	1.00	-0.004	0.030	0.896	0.974	-0.029	0.026	0.254	1.051
rs1205	1:159682233_C/T	t	c	++	0.023	0.025	0.354	0.99	0.047	0.040	0.240	1.002	0.008	0.032	0.806	1.041
rs1800562	6:26093141_G/A	a	g	++	0.134	0.146	0.358	0.99	0.170	0.198	0.391	0.971	0.092	0.216	0.671	1.022
rs699947	6:43736389_A/C	a	c	-+	-0.018	0.020	0.358	1.00	-0.045	0.030	0.135	0.960	0.002	0.026	0.942	0.971
rs11568820	12:48302545_C/T	t	c	+-	0.035	0.038	0.359	1.00	0.223	0.065	0.001	0.967	-0.064	0.047	0.180	1.039
rs1800947	1:159683438_C/G	c	g	++	0.132	0.146	0.364	0.61	0.182	0.245	0.456	0.766	0.105	0.182	0.564	0.982
rs35767	12:102875569_A/G	a	g	++	0.042	0.048	0.375	1.00	0.068	0.082	0.406	0.970	0.029	0.059	0.619	1.027
rs1801280	8:18257854_T/C	t	c	-+	-0.019	0.022	0.389	0.93	-0.051	0.032	0.106	0.994	0.010	0.030	0.735	1.094
rs4951291	1:204006538_T/C	t	c	+-	0.074	0.091	0.417	0.99	0.117	0.104	0.258	0.952	-0.068	0.186	0.717	0.851
rs4951039	1:204006597_G/A	a	g	-+	-0.073	0.091	0.419	1.00	-0.115	0.104	0.266	0.951	0.063	0.186	0.736	0.853
rs2228000	3:14199887_G/A	a	g	-+	0.026	0.035	0.465	1.00	-0.072	0.054	0.182	0.997	0.097	0.046	0.035	1.011
rs2241766	3:186570892_T/G	t	g	++	0.051	0.070	0.470	1.00	0.061	0.114	0.592	0.961	0.045	0.089	0.618	0.861
rs2066844	16:50745926_C/T	t	c	+-	0.139	0.201	0.491	1.00	0.622	0.255	0.015	0.901	-0.660	0.328	0.044	0.920
rs2292832	2:241395503_T/C	t	c	--	-0.024	0.035	0.499	1.00	-0.027	0.047	0.561	0.922	-0.019	0.051	0.715	0.688
rs1799853	10:96702047_C/T	t	c	+-	-0.044	0.067	0.511	0.99	0.038	0.099	0.706	0.997	-0.112	0.091	0.217	0.983

rs10380	5:7897191_C/T	t	c	--	-0.051	0.077	0.512	0.92	-0.122	0.130	0.351	0.997	-0.012	0.096	0.897	0.990
rs20417	1:186650321_C/G	c	g	+-	-0.032	0.052	0.537	1.00	0.001	0.077	0.989	0.988	-0.058	0.069	0.399	1.019
rs1042838	11:100933412_C/A	a	c	--	-0.030	0.051	0.558	1.00	-0.010	0.079	0.902	1.000	-0.045	0.067	0.506	1.033
rs7849	10:102122603_T/C	t	c	+-	0.024	0.045	0.593	1.00	0.085	0.073	0.250	0.983	-0.013	0.057	0.827	1.007
rs9858822	3:12411238_A/C	a	c	+-	-3.552	6.868	0.605	1.00	9406	72463	0.897	0.916	-3.552	6.868	0.605	0.987
rs26779	5:80059203_C/T	t	c	-+	-0.012	0.023	0.620	0.99	-0.062	0.035	0.076	1.001	0.029	0.031	0.361	0.998
rs9282564	7:87229440_T/C	t	c	-+	0.022	0.047	0.646	0.99	-0.013	0.145	0.927	0.748	0.026	0.050	0.605	1.145
rs1128503	7:87179601_A/G	a	g	++	0.009	0.022	0.664	1.00	0.010	0.033	0.759	0.996	0.009	0.029	0.758	1.019
rs17655	13:103528002_G/C	c	g	+-	0.014	0.034	0.679	1.00	0.076	0.058	0.188	1.004	-0.018	0.042	0.663	1.064
rs1800566	16:69745145_G/A	a	g	+-	0.017	0.040	0.681	1.00	0.048	0.068	0.480	1.000	-0.001	0.050	0.988	1.044
rs1799750	11:102670495_TC/T	t	tc	+-	-0.009	0.021	0.682	1.00	0.016	0.034	0.630	0.885	-0.025	0.028	0.357	0.937
rs1799929	8:18257994_C/T	t	c	+-	0.009	0.022	0.696	1.00	0.045	0.033	0.167	0.989	-0.023	0.031	0.450	1.087
rs1544410	12:48239835_C/T	t	c	+-	-0.009	0.024	0.712	1.00	0.005	0.034	0.892	0.998	-0.021	0.032	0.524	1.066
rs184967	5:80149981_A/G	a	g	-+	-0.019	0.054	0.731	0.99	-0.052	0.077	0.504	1.012	0.013	0.076	0.861	1.015
rs895819	19:13947292_T/C	t	c	++	0.010	0.029	0.739	0.99	0.007	0.044	0.867	0.921	0.012	0.039	0.767	0.951
rs6083	15:58838010_A/G	a	g	+-	0.009	0.028	0.760	0.43	0.030	0.042	0.477	0.886	-0.009	0.038	0.817	0.855
rs1950902	14:64882380_A/G	a	g	+-	-0.014	0.048	0.768	0.99	0.009	0.074	0.900	0.985	-0.030	0.062	0.623	1.007
rs1202168	7:87195962_G/A	a	g	+-	0.006	0.022	0.780	1.00	0.014	0.033	0.667	0.988	0.000	0.029	0.993	0.977
rs373572	3:8955389_C/T	t	c	+-	-0.008	0.030	0.781	1.00	0.047	0.049	0.341	0.996	-0.041	0.038	0.280	1.076
rs909460	18:55254310_C/T	t	c	-+	9.673	41.557	0.816	1.00	-1743	13204	0.895	0.837	9.690	41.558	0.816	0.815
rs2240308	17:63554591_G/A	a	g	+-	0.005	0.022	0.824	1.00	0.019	0.040	0.632	0.650	-0.001	0.026	0.965	1.028
rs1801131	1:11854476_T/G	t	g	++	0.006	0.029	0.836	0.03	0.006	0.044	0.889	0.969	0.006	0.039	0.879	1.012
rs1048943	15:75012985_T/C	t	c	++	0.022	0.117	0.849	1.00	0.089	0.386	0.817	0.921	0.016	0.122	0.899	1.075

* rs34612342 was dropped due to low imputation quality.

Supplementary Table S8. Linkage disequilibrium (LD) between "positive" SNPs with r^2>0.20.

SNP1	SNP2	r^2	D'
rs12953717	rs4464148	0.346066	0.727533
rs355527	rs961253	0.900220	0.999999

Supplementary Table S9. Summary of results from meta-analyses and credibility assessment (negative and null associations).

Gene	Variant	Cases vs. controls (number of samples)	ALLELIC MODEL						RECESSIVE MODEL: var/var VS. wt/wt & wt/var						DOMINANT MODEL:wt/var & var/var VS. wt/wt						Classification
			N	Effect size		Heterogeneity		Power	N	Effect size		Heterogeneity		Power	N	Effect size		Heterogeneity		Power	
				OR (95% CI)	P value	I^2 (95% CI)	*P value*			OR (95% CI)	P value	I^2 (95% CI)	*P* value			OR (95% CI)	P value	I^2 (95% CI)	*P* value		
CYP2E1	96-bp insertion	1614 vs. 2396 (6)	6	1.21 (1.00, 1.47)	0.051	37 (0, 97)	0.158	0.65	6	1.18 (0.85, 1.63)	0.321	0 (0, 92)	0.540	0.14	6	1.26 (0.99, 1.60)	0.057	42 (0, 96)	0.122	0.95	Negative
NAT2	rs1801280	2470 vs. 2971 (5)	5	1.01 (0.82, 1.25)	0.891	80(63, 99)	4.31E-04	0.54	5	0.97 (0.75, 1.24)	0.795	60 (0, 98)	0.040	0.32	5	1.04 (0.76, 1.44)	0.796	82(71, 99)	2.12E-04	0.77	Negative
MMP3	rs3025058	1212 vs. 1631(8)	7	0.96(0.79,1.16)	0.681	42(0,93)	0.108	0.08	8	1.00(0.73,1.35)	0.979	21(0,91)	0.264	0.05	7	0.88(0.66,1.18)	0.382	56(0,92)	0.032	0.39	Negative
MMP9	rs3918242	621 vs. 904(5)	4	0.88(0.68,1.16)	0.369	13(0,94)	0.325	0.12	4	0.79(0.18,3.41)	0.752	11(0,93)	0.339	0.08	5	0.88(0.67,1.16)	0.358	18(0,90)	0.302	0.18	Negative
ADH1C	rs698	4545 vs. 18180(9**)	9	1.01(0.89,1.15)	0.855	76(61,98)	5.21E-05	0.06	9	1.12(0.92,1.37)	0.266	59(11,93)	0.012	0.70	9	0.97(0.82,1.15)	0.743	72(53,97)	3.17E-04	0.15	Negative
VEGF	rs3025039	3090 vs. 3147(11)	10	1.08(0.93,1.27)	0.309	58(7,90)	0.015	0.21	10	1.17(0.91,1.52)	0.226	0(0,83)	0.692	0.20	11	1.13(0.95,1.33)	0.161	54(6,84)	0.015	0.40	Negative
VEGF	rs2010963	2426 vs. 2387(7)	7	0.94(0.85,1.04)	0.216	31(0,87)	0.193	0.18	7	1.01(0.87,1.18)	0.870	0(0,78)	0.539	0.05	7	0.90(0.74,1.09)	0.271	58(0,91)	0.025	0.42	Negative
MGMT	rs2308321	2863 vs. 8040(6)	5	1.00(0.73,1.38)	0.988	77(43,98)	0.002	0.05	5	1.31(0.56,3.08)	0.533	29(0,96)	0.229	0.39	6	0.98(0.77,1.25)	0.880	71(37,97)	0.004	0.07	Negative
IL10	rs1800896	4510 vs. 5363(9*)	9	1.00(0.92,1.08)	0.947	20(0,84)	0.268	0.06	9	0.98(0.90,1.08)	0.738	0(0,83)	0.496	0.10	9	1.00(0.88,1.15)	0.925	23(0,83)	0.235	0.05	Negative
PTGS2/COX2	rs4648298	2359 vs. 2538(5)	na	na	na	na	na	na	na	na	na	na	na	na	5	0.88(0.52,1.51)	0.653	71(20,97)	0.008	0.18	Negative
PTGS2/COX2	rs5273	720 vs. 1163(4)	na	na	na	na	na	na	na	na	na	na	na	na	3	0.71(0.46,1.10)	0.128	0(0,63)	0.924		Negative
TNF-alpha	rs1800629	2980 vs. 4102(13)	13	1.11(0.90,1.37)	0.333	74(59,94)	7.40E-06	0.58	13	1.34(0.82,2.19)	0.238	48(0,87)	0.030	0.46	13	1.09(0.87,1.36)	0.468	69(51,93)	9.40E-05	0.68	Negative
NOD2	rs2066845	3303 vs. 3088(9)	9	1.33(0.94,1.87)	0.103	0(0,70)	0.529	0.23	na	na	na	na	na	na	9	1.34(0.95,1.88)	0.098	0(0,71)	0.495	0.42	Negative
NAT1	slow/rapid	4791 vs. 6628 (15)	7	0.92 (0.83, 1.02)	0.116	0 (0, 83)	0.470	0.53	7	1.05 (0.86, 1.27)	0.652	0 (0, 34)	0.932	0.12	14	0.99 (0.87, 1.14)	0.926	55 (14, 87)	0.007	0.06	Negative
MDM2	rs2279744	3564 vs. 2835 (10)	10	1.09 (0.92, 1.30)	0.307	78 (66, 96)	5.41E-06	0.40	10	1.03 (0.84, 1.25)	0.806	52 (0, 92)	0.029	0.08	10	1.26 (0.94, 1.68)	0.126	80 (77, 98)	1.04E-06	0.98	Negative
VDR	rs731236	2091 vs. 2257 (9)	9	1.03 (0.90, 1.18)	0.674	44 (0, 93)	0.077	0.08	8	1.13 (0.83, 1.54)	0.435	62 (16, 95)	0.010	0.06	9	0.99 (0.86, 1.14)	0.900	11 (0, 86)	0.346	0.60	Negative
CTLA4	rs231775	777 vs. 1356(5)	5	1.13(0.98,1.30)	0.087	0(0,87)	0.565	0.27	5	1.04(0.81,1.35)	0.744	0(0,96)	0.603	0.06	5	1.29(0.93,1.80)	0.126	53(0,98)	0.075	0.76	Negative
CX3CR1	rs3732379	3548 vs. 3649(3*)	3	0.96(0.81,1.14)	0.636	54(0,99)	0.112	0.12	3	0.65(0.33,1.29)	0.220	80(21,100)	0.007	1.00	3	1.02(0.90,1.16)	0.718	14(0,99)	0.312	0.07	Negative
DLG5	rs1248696	3672 vs. 3618(3*)	3	0.57(0.19,1.69)	0.308	98(94,100)	1.24E-23	1.00	3	0.44(0.12,1.69)	0.235	66(0,99)	0.054	0.89	3	0.47(0.12,1.89)	0.288	98(96,100)	5.23E-29	1.00	Negative
DNMT3B	rs2424913	2398 vs. 3740(5)	5	1.04(0.95,1.14)	0.360	0(0,98)	0.586	0.12	3	1.04(0.91,1.18)	0.569	0(0,94)	0.847	0.10	5	1.08(0.92,1.26)	0.341	0(0,95)	0.560	0.27	Negative
ERCC1	rs11615	4231 vs. 4928(7*)	7	0.99(0.93,1.05)	0.756	0(0,87)	0.429	0.06	7	0.95(0.84,1.07)	0.392	0(0,87)	0.458	0.14	7	1.01(0.93,1.1)	0.858	0(0,87)	0.456	0.06	Negative
ERCC1	rs3212986	1815 vs. 1858(4*)	3	1.11(0.93,1.31)	0.245	42(0,99)	0.178	0.28	3	1.22(0.92,1.63)	0.166	7(0,98)	0.341	0.34	4	1.11(0.96,1.27)	0.151	7(0,95)	0.357	0.35	Negative
ESR1	rs2234693	2792 vs. 3288(6)	6	0.90(0.76,1.07)	0.229	66(26,98)	0.012	0.53	6	0.87(0.70,1.09)	0.231	39(0,97)	0.149	0.59	6	0.91(0.68,1.22)	0.539	70(47,99)	0.005	0.39	Negative
ESR1	rs2077647	2594 vs. 3086(4)	4	1.03(0.89,1.19)	0.699	56(0,100)	0.079	0.09	4	1.09(0.77,1.55)	0.637	75(75,100)	0.007	0.27	4	1.01(0.90,1.14)	0.879	0(0,98)	0.501	0.05	Negative
ESR1	rs3798577	2593 vs. 3100(4)	4	1.02(0.94,1.10)	0.706	6(0,99)	0.361	0.07	4	1.08(0.95,1.23)	0.235	0(0,98)	0.639	0.23	4	0.97(0.86,1.09)	0.614	0(0,98)	0.391	0.08	Negative
FOLH1	His475Tyr	3389 vs. 4333(4)	4	0.94(0.86,1.02)	0.155	0(0,0)	0.983	0.17	na	na	na	na	na	na	4	0.94(0.85,1.04)	0.224	0(0,9)	0.968	0.23	Negative
GPX1	rs1050450	1974 vs. 2481(4)	3	1.01(0.90,1.13)	0.917	0(0,96)	0.446	0.05	3	0.90(0.67,1.20)	0.467	0(0,76)	0.912	0.16	4	0.98(0.83,1.15)	0.774	31(0,94)	0.226	0.06	Negative
GPX4	rs713041	1733 vs. 1519(3)	3	0.95(0.76,1.19)	0.685	78(18,100)	0.011	0.11	3	0.98(0.81,1.18)	0.812	0(0,97)	0.466	0.06	3	0.91(0.62,1.35)	0.649	84(45,100)	0.002	0.25	Negative
HPGD	rs9312555	3976 vs. 4831(3*)	3	0.95(0.88,1.04)	0.252	0(0,94)	0.629	0.14	3	1.06(0.81,1.40)	0.668	0(0,97)	0.415	0.71	3	0.93(0.85,1.03)	0.150	0(0,87)	0.817	0.33	Negative
HPGD	rs17360144	3962 vs. 4826(3*)	3	1.01(0.88,1.15)	0.917	20(0,98)	0.289	0.05	3	1.07(0.34,3.33)	0.913	70(0,99)	0.034	0.06	3	1.01(0.87,1.17)	0.923	31(0,98)	0.236	0.05	Negative
ICAM1	rs1799969	3275 vs. 3368(3*)	2	1.34(0.78,2.31)	0.293	86(30,100)	0.007	0.98	2	1.39(0.92,2.10)	0.118	0(0,99)	0.751	0.38	2	1.40(0.72,2.72)	0.321	88(42,100)	0.003	1.00	Negative
IRS2	rs1805097	3890 vs. 4778(3)	3	0.95(0.9,1.02)	0.138	0(0,96)	0.693	0.21	3	1.02(0.91,1.14)	0.732	0(0,97)	0.624	0.06	3	0.91(0.82,1.02)	0.116	37(0,99)	0.203	0.57	Negative
LCT	rs4988235	1927 vs. 2182(6)	6	1.00(0.85,1.18)	0.991	59(0,93)	0.033	0.05	6	1.03(0.77,1.38)	0.841	58(0,97)	0.036	0.07	6	0.95(0.77,1.16)	0.594	42(0,89)	0.128	0.12	Negative
LEP	rs2167270	1839 vs. 2429(3)	3	0.94(0.86,1.03)	0.205	0(0,98)	0.478	0.16	3	0.74(0.52,1.04)	0.085	46(0,100)	0.159	0.90	3	1.03(0.91,1.17)	0.621	0(0,92)	0.593	0.08	Negative
LEP	rs7799039	2324 vs. 2776(3)	3	0.95(0.88,1.03)	0.223	0(0,98)	0.461	0.15	3	0.89(0.77,1.02)	0.088	0(0,99)	0.528	0.36	3	0.98(0.87,1.10)	0.702	0(0,95)	0.524	0.06	Negative
LEPR	rs1137101	4059 vs. 4225(4*)	4	1.02(0.96,1.08)	0.611	0(0,94)	0.518	0.07	4	1.03(0.93,1.14)	0.598	0(0,48)	0.905	0.08	4	1.04(0.91,1.19)	0.534	25(0,98)	0.262	0.13	Negative
MBL2	rs5030737	1185 vs. 1199(4)	4	1.03(0.81,1.30)	0.805	0(0,77)	0.806	0.05	2	0.95(0.25,3.57)	0.939	0(0,77)	0.948	0.05	4	1.04(0.81,1.33)	0.784	0(0,78)	0.793	0.06	Negative
MBL2	rs1800451	819 vs. 843(3)	3	1.29(0.92,1.81)	0.134	0(0,98)	0.442	0.26	na	na	na	na	na	na	3	1.26(0.85,1.87)	0.242	0(0,98)	0.451	0.36	Negative
MBL2	rs7096206	826 vs. 871(3)	3	0.97(0.81,1.16)	0.761	0(0,92)	0.805	0.06	3	0.95(0.53,1.71)	0.866	15(0,99)	0.310	0.05	3	0.97(0.79,1.20)	0.809	0(0,53)	0.943	0.06	Negative
MBL2	rs11003125	822 vs. 870(3)	3	0.91(0.70,1.17)	0.462	53(0,99)	0.120	0.15	3	0.89(0.64,1.23)	0.483	0(0,100)	0.372	0.11	3	0.91(0.68,1.22)	0.540	41(0,99)	0.186	0.16	Negative
MBL2	rs7095891	822 vs. 870(3)	3	1.02(0.86,1.20)	0.847	0(0,90)	0.813	0.05	3	0.99(0.68,1.45)	0.952	0(0,0)	0.978	0.05	3	1.03(0.84,1.27)	0.783	0(0,96)	0.700	0.06	Negative
miR	rs11614913	2101 vs. 2961(7)	7	0.95(0.72,1.26)	0.740	91(74,98)	4.60E-12	0.15	7	0.84(0.54,1.31)	0.450	89(86,99)	4.51E-10	0.73	7	0.91(0.62,1.34)	0.647	86(59,96)	1.81E-07	0.31	Negative
miR	rs3746444	717 vs. 1055(3)	2	1.20(0.91,1.59)	0.199	27(0,100)	0.243	0.31	2	1.68(0.58,4.86)	0.340	66(0,100)	0.085	0.55	3	0.98(0.79,1.21)	0.860	0(0,88)	0.780	0.05	Negative
MSH2	rs2303428	4371 vs. 4590(5)	5	0.96(0.87,1.06)	0.402	0(0,87)	0.519	0.09	5	1.02(0.70,1.48)	0.929	0(0,64)	0.863	0.05	5	0.95(0.84,1.07)	0.377	9(0,91)	0.353	0.17	Negative

MSH2	rs2303425	1775 vs. 2206(3)	3	1.01(0.87,1.17)	0.947	14(0,98)	0.312	0.05	3	2.34(0.56,9.74)	0.243	74(4,99)	0.021	0.98	3	0.97(0.78,1.20)	0.764	47(0,99)	0.150	0.07	Negative
MSH2	rs4987188	3719 vs. 3852(6)	3	1.10(0.79,1.54)	0.558	0(0,97)	0.521	0.09	na	na	na	na	na	na	6	1.18(0.92,1.52)	0.202	0(0,57)	0.851	0.25	Negative
MSH2	rs1981929	4319 vs. 4009(3*)	3	1.02(0.95,1.09)	0.608	13(0,97)	0.319	0.07	3	1.04(0.86,1.26)	0.659	56(0,99)	0.104	0.10	3	1.03(0.94,1.12)	0.562	0(0,84)	0.829	0.10	Negative
MSH2	rs4952887	4321 vs. 4011(3*)	3	1.03(0.93,1.15)	0.537	0(0,94)	0.610	0.07	3	0.70(0.32,1.51)	0.360	52(0,99)	0.126	0.24	3	1.05(0.94,1.18)	0.378	0(0,85)	0.839	0.13	Negative
MSH6	rs1042821	4936 vs. 8223(6)	6	1.05(0.93,1.20)	0.408	65(16,97)	0.014	0.07	6	0.98(0.80,1.21)	0.875	0(0,88)	0.576	0.06	6	1.07(0.92,1.25)	0.348	68(23,98)	0.009	0.12	Negative
MSH6	rs1800936	3867 vs. 4019(3*)	3	1.31(0.86,2.00)	0.203	95(82,100)	8.01E-09	0.99	3	1.45(0.76,2.76)	0.255	70(0,99)	0.034	0.64	3	1.38(0.85,2.24)	0.196	95(84,100)	3.54E-09	1.00	Negative
MSH6	rs3136245	4314 vs. 4013(3*)	3	0.96(0.89,1.04)	0.301	7(0,98)	0.340	0.12	3	0.93(0.71,1.21)	0.575	31(0,99)	0.233	0.10	3	0.96(0.87,1.05)	0.325	0(0,96)	0.479	0.15	Negative
MSH6	rs3136329	4305 vs. 3996(3*)	3	1.02(0.96,1.09)	0.447	0(0,96)	0.554	0.07	3	1.04(0.91,1.18)	0.561	18(0,98)	0.294	0.10	3	1.03(0.93,1.13)	0.590	7(0,97)	0.343	0.10	Negative
MSH6	rs6713506	4306 vs. 3995(3*)	3	0.96(0.88,1.05)	0.355	0(0,92)	0.740	0.10	3	0.97(0.73,1.28)	0.810	0(0,90)	0.799	0.06	3	0.95(0.87,1.05)	0.333	0(0,93)	0.692	0.19	Negative
NBS1	rs1805794	3948 vs. 4316(5)	5	0.90(0.74,1.10)	0.311	83(72,99)	1.03E-04	0.62	5	0.80(0.58,1.11)	0.184	75(48,99)	0.003	0.90	5	0.95(0.77,1.18)	0.660	70(29,99)	0.010	0.21	Negative
NFKB1	rs28362491	2897 vs. 4961(11)	11	1.19(0.99,1.43)	0.064	85(65,95)	5.22E-10	0.62	11	1.14(0.89,1.46)	0.313	71(47,96)	1.30E-04	0.15	11	1.44(1.02,2.05)	0.040	88(85,98)	4.77E-13	1.00	Negative
NFKB1A	rs696	2310 vs. 3018(4*)	4	1.01(0.93,1.09)	0.871	0(0,94)	0.436	0.05	4	1.10(0.95,1.28)	0.188	0(0,78)	0.887	0.26	4	0.99(0.79,1.25)	0.940	70(6,98)	0.019	0.05	Negative
NOS3	rs1799983	2301 vs. 2478(5)	5	0.98(0.79,1.22)	0.845	75(46,99)	0.003	0.06	5	0.92(0.59,1.45)	0.730	59(0,99)	0.043	0.10	5	1.00(0.80,1.25)	0.994	64(0,98)	0.026	0.05	Negative
NOS3	rs2070744	1576 vs. 1782(3)	3	1.10(0.87,1.40)	0.423	66(0,99)	0.052	0.21	3	0.96(0.71,1.29)	0.771	0(0,82)	0.821	0.06	3	1.13(0.85,1.50)	0.398	64(0,99)	0.060	0.41	Negative
PMS2	rs6463524	3713 vs. 3374(3)	3	1.06(0.85,1.33)	0.596	80(35,100)	0.006	0.16	3	1.09(0.83,1.43)	0.545	10(0,99)	0.331	0.10	3	1.05(0.81,1.36)	0.694	80(31,100)	0.007	0.16	Negative
PMS2	rs2286680	4320 vs. 4010(3*)	3	1.01(0.93,1.11)	0.757	0(0,87)	0.826	0.05	3	1.06(0.78,1.45)	0.706	0(0,94)	0.668	0.07	3	1.01(0.92,1.12)	0.822	0(0,91)	0.802	0.05	Negative
PMS2	rs235060	4315 vs. 4006(3*)	3	0.97(0.90,1.04)	0.335	0(0,63)	0.925	0.09	3	1.00(0.83,1.20)	0.981	0(0,95)	0.647	0.05	3	0.95(0.87,1.03)	0.231	0(0,88)	0.788	0.21	Negative
RAD51	rs1801320	853 vs. 840(5)	5	1.07(0.44,2.62)	0.876	96(87,99)	9.64E-23	0.10	5	1.05(0.17,6.58)	0.960	96(84,99)	3.23E-19	0.06	5	1.13(0.56,2.27)	0.728	89(71,99)	1.71E-07	0.24	Negative
RNASEL	rs486907	3524 vs. 3789(3*)	3	1.01(0.94,1.08)	0.777	0(0,95)	0.722	0.06	3	1.05(0.91,1.21)	0.491	0(0,96)	0.684	0.10	3	1.00(0.91,1.09)	0.948	0(0,86)	0.867	0.05	Negative
SERPINE1	rs1799889	2241 vs. 2735(3)	3	0.97(0.89,1.05)	0.430	0(0,95)	0.644	0.08	3	1.08(0.94,1.25)	0.266	0(0,2)	0.974	0.20	3	0.87(0.77,0.99)	0.030	0(0,98)	0.372	0.62	Negative
SHMT1	rs1979277	1697 vs. 1994(5)	4	0.93(0.83,1.04)	0.222	19(0,95)	0.296	0.17	4	0.89(0.59,1.33)	0.566	64(0,99)	0.041	0.18	4	0.94(0.83,1.07)	0.379	0(0,87)	0.636	0.16	Negative
SLC19A1	rs1051266	4289 vs. 5817(6)	6	1.01(0.92,1.11)	0.809	52(0,86)	0.065	0.06	6	0.99(0.90,1.10)	0.868	1(0,74)	0.410	0.05	6	1.04(0.90,1.20)	0.590	54(0,90)	0.054	0.15	Negative
SLC22A4	rs1050152	3820 vs. 4749(5*)	5	1.01(0.96,1.06)	0.634	0(0,96)	0.414	0.06	5	0.99(0.91,1.07)	0.788	0(0,97)	0.510	0.05	5	1.06(0.96,1.16)	0.256	19(0,95)	0.295	0.22	Negative
SOD2	rs4880	2496 vs. 2283(4)	4	0.99(0.90,1.08)	0.767	0(0,62)	0.916	0.05	4	0.94(0.80,1.11)	0.461	0(0,81)	0.778	0.11	4	1.01(0.89,1.16)	0.862	0(0,0)	0.993	0.05	Negative
SULT1A1	rs9282861	4636 vs. 6291(12)	12	1.00(0.92,1.10)	0.914	48(0,90)	0.030	0.05	12	0.98(0.76,1.26)	0.875	72(48,92)	0.000	0.15	12	1.00(0.93,1.08)	0.942	0(0,66)	0.782	0.05	Negative
SULT1A2	rs1059491	1806 vs. 1986(3)	3	1.05(0.95,1.17)	0.308	5(0,98)	0.347	0.11	3	1.03(0.82,1.30)	0.783	15(0,98)	0.309	0.06	3	1.08(0.95,1.23)	0.219	0(0,94)	0.625	0.22	Negative
Survivin31	rs9904341	1452 vs. 1475(4)	4	1.20(0.84,1.72)	0.317	90(71,99)	8.51E-07	0.69	4	1.24(0.77,2.02)	0.375	84(55,99)	3.22E-04	0.69	4	1.23(0.77,1.96)	0.391	87(60,99)	5.63E-05	0.70	Negative
TLR4	rs4986790	4550 vs. 5432(10*)	8	1.26(0.95,1.69)	0.112	58(8,96)	0.018	0.82	6	1.77(0.94,3.31)	0.075	10(0,93)	0.353	0.55	9	1.21(0.97,1.52)	0.088	37(0,94)	0.121	0.89	Negative
TLR4	rs4986791	3387 vs. 4072(6)	6	1.05(0.84,1.31)	0.687	22(0,98)	0.267	0.08	2	1.45(0.59,3.54)	0.413	3(0,99)	0.310	0.17	6	1.03(0.81,1.31)	0.782	24(0,98)	0.256	0.07	Negative
UGT1A1	rs10929302	1560 vs. 1952(3)	3	1.06(0.96,1.18)	0.248	0(0,98)	0.389	0.13	3	1.19(0.92,1.53)	0.181	25(0,98)	0.262	0.41	3	1.04(0.90,1.19)	0.612	0(0,59)	0.918	0.09	Negative
UGT1A1	rs4124874	1560 vs. 1987(3)	3	0.99(0.83,1.18)	0.930	58(0,99)	0.093	0.05	3	1.01(0.65,1.57)	0.962	74(6,100)	0.021	0.05	3	1.04(0.90,1.21)	0.563	0(0,97)	0.494	0.08	Negative
UGT1A1	UGT1A1*28	3344 vs. 4159(8)	8	1.03(0.93,1.14)	0.546	45(0,90)	0.081	0.09	8	1.03(0.89,1.19)	0.716	0(0,92)	0.479	0.07	8	1.05(0.90,1.22)	0.520	52(0,93)	0.040	0.18	Negative
UGT1A6	rs2070959	4773 vs. 5070(4*)	4	1.02(0.96,1.08)	0.568	0(0,85)	0.737	0.07	4	1.03(0.90,1.18)	0.672	0(0,95)	0.493	0.07	4	1.00(0.90,1.12)	0.940	29(0,97)	0.236	0.05	Negative
UGT1A7	x	2316 vs. 2918(6)	6	1.14(0.98,1.33)	0.096	65(9,95)	0.014	0.65	6	1.21(0.97,1.51)	0.092	45(0,94)	0.103	0.76	6	1.19(0.91,1.55)	0.201	73(22,94)	0.003	0.83	Negative
WRN	rs2725362	4681 vs. 4833(3)	3	0.95(0.89,1.02)	0.128	18(0,98)	0.294	0.24	3	0.91(0.81,1.02)	0.116	16(0,99)	0.303	0.44	3	0.95(0.87,1.03)	0.232	0(0,96)	0.405	0.21	Negative
XPA	rs1800975	2308 vs. 2871(6**)	5	0.92(0.76,1.10)	0.341	69(12,97)	0.011	0.29	5	0.81(0.58,1.15)	0.244	58(0,96)	0.048	0.63	6	0.95(0.79,1.13)	0.537	53(0,95)	0.061	0.15	Negative
MMP2	rs243865	1215 vs. 1347 (8)	7	1.11 (0.80, 1.55)	0.541	72 (47, 97)	0.001	0.46	7	1.80 (0.90, 3.60)	0.099	38 (0, 93)	0.155	0.80	8	0.98 (0.72, 1.34)	0.913	58 (5, 95)	0.020	0.10	Negative
MMP7	rs11568818	2139 vs. 2355 (9*)	9	0.96 (0.78, 1.16)	0.649	61 (17, 96)	0.008	0.06	6	0.91 (0.57, 1.45)	0.683	75 (52, 98)	0.001	0.15	9	0.97 (0.77, 1.23)	0.811	48 (0, 94)	0.053	0.08	Negative
MMP9	rs17576	1520 vs. 1753 (4*)	4	0.83 (0.66, 1.04)	0.105	71 (9, 98)	0.017	0.71	4	0.84 (0.68, 1.04)	0.108	0 (0, 91)	0.639	0.36	4	0.77 (0.56, 1.06)	0.108	74 (21, 98)	0.008	0.96	Negative
ALDH2	rs886205	2595 vs. 2993 (4*)	4	1.01 (0.91, 1.12)	0.898	0 (0, 88)	0.521	0.05	4	0.93 (0.68, 1.28)	0.656	0 (0, 72)	0.880	0.07	4	1.02 (0.89, 1.16)	0.780	15 (0, 92)	0.316	0.06	Negative
VEGF	rs833061	989 vs. 897 (4)	4	1.16 (0.77, 1.74)	0.476	88 (66, 99)	1.00E-05	0.36	4	1.27 (0.69, 2.34)	0.444	83 (50, 99)	0.001	0.52	4	1.18 (0.71, 1.94)	0.525	82 (49, 99)	0.001	0.39	Negative
VEGF	rs1570360	1150 vs. 1342 (4)	4	0.98 (0.87, 1.12)	0.798	0 (0, 86)	0.741	0.06	4	0.98 (0.74, 1.29)	0.869	0 (0, 82)	0.822	0.05	4	0.98 (0.83, 1.16)	0.816	0 (0, 90)	0.700	0.06	Negative
IL1B	rs1143627	1251 vs. 2124 (5)	5	1.03 (0.93, 1.14)	0.611	0 (0, 78)	0.782	0.07	5	1.02 (0.75, 1.40)	0.881	50 (0, 96)	0.094	0.05	5	1.06 (0.92, 1.22)	0.439	0 (0, 89)	0.690	0.13	Negative
IL1B	rs16944	577 vs. 828 (3)	3	0.96 (0.82, 1.13)	0.626	0 (0, 96)	0.667	0.07	3	1.08 (0.78, 1.48)	0.649	0 (0, 91)	0.773	0.08	3	0.82 (0.55, 1.24)	0.352	63 (0, 100)	0.067	0.44	Negative
IL4	rs2057768	1123 vs. 1483 (5)	5	0.94 (0.78 1.13)	0.529	32 (0, 96)	0.208	0.09	5	0.88 (0.59, 1.31)	0.529	0 (0, 88)	0.577	0.09	5	0.94 (0.77, 1.15)	0.556	25 (0, 95)	0.257	0.11	Negative
IL4R	rs1805012	3801 vs. 3962 (3*)	3	1.05 (0.89, 1.24)	0.566	49 (0, 99)	0.143	0.10	3	1.49 (0.99, 2.24)	0.058	0 (0, 97)	0.494	0.56	3	1.02 (0.83, 1.25)	0.874	57 (0, 99)	0.095	0.07	Negative
IL4R	rs1805015	3607 vs. 3692 (3*)	3	1.02 (0.94, 1.12)	0.599	0 (0, 96)	0.673	0.06	3	1.23 (0.94, 1.62)	0.137	0 (0, 96)	0.643	0.34	3	1.00 (0.91, 1.11)	0.962	0 (0, 98)	0.537	0.05	Negative
IL4R	rs1801275	1764 vs. 1747 (4*)	4	0.94 (0.74, 1.19)	0.589	67 (0, 99)	0.027	0.11	4	1.33 (0.93, 1.90)	0.113	0 (0, 54)	0.931	0.39	4	0.88 (0.65, 1.18)	0.394	71 (20, 99)	0.015	0.43	Negative
IL6	rs1800797	2619 vs. 3396 (3)	2	1.00 (0.81, 1.23)	0.981	0 (0, 100)	0.544	0.05	2	1.04 (0.72, 1.51)	0.835	0 (0, 100)	0.631	0.09	3	0.96 (0.87, 1.07)	0.472	0 (0, 90)	0.889	0.11	Negative

IL6R	rs8192284	3070 vs. 20173 (3)	3	0.97 (0.91, 1.04)	0.437	0 (0, 96)	0.469	0.11	3	0.94 (0.82, 1.07)	0.363	0 (0, 92)	0.862	0.15	3	1.02 (0.87, 1.20)	0.796	36 (0, 98)	0.208	0.08	Negative
IL10	rs1800872	1598 vs. 2760 (7)	7	1.23 (0.92, 1.64)	0.163	86 (68, 97)	1.17E-07	0.08	7	1.57 (0.74, 3.33)	0.240	85 (66, 97)	5.44E-07	0.08	7	1.15 (0.92, 1.44)	0.209	64 (14, 94)	0.011	0.09	Negative
PTGS1/COX1	rs5788	4087 vs. 4808 (3)	3	1.02 (0.86, 1.21)	0.856	75 (7, 99)	0.019	0.06	3	0.93 (0.68, 1.27)	0.640	0 (0, 95)	0.575	0.08	3	1.03 (0.84, 1.26)	0.778	76 (13, 99)	0.015	0.09	Negative
PTGS1/COX1	rs10306135	4644 vs. 6893 (3)	3	1.01 (0.94, 1.09)	0.705	0 (0, 93)	0.661	0.05	3	0.96 (0.74, 1.24)	0.742	0 (0, 70)	0.919	0.06	3	1.02 (0.94, 1.11)	0.595	0 (0, 93)	0.678	0.08	Negative
PTGS1/COX1	rs6478565	4418 vs. 5909 (3*)	3	0.97 (0.89, 1.05)	0.415	20 (0, 98)	0.288	0.09	3	0.94 (0.71, 1.26)	0.697	25 (0, 99)	0.264	0.08	3	0.95 (0.84, 1.09)	0.478	55 (0, 99)	0.109	0.22	Negative
PTGS2/COX2	rs4648261	4687 vs. 6951 (3)	2	1.05 (0.87, 1.27)	0.640	0 (0, 93)	0.907	0.07	2	3.04 (0.76, 12.18)	0.116	0 (0, 97)	0.859	0.70	3	0.92 (0.75, 1.13)	0.434	37 (0, 98)	0.202	0.17	Negative
PTGS2/COX2	rs2745557	4300 vs. 5825 (5)	5	1.05 (0.94, 1.19)	0.373	48 (0, 96)	0.102	0.16	5	1.01 (0.84, 1.23)	0.885	0 (0, 83)	0.608	0.05	5	1.09 (0.92, 1.29)	0.304	60 (0, 98)	0.039	0.53	Negative
PTGS2/COX2	rs20424	3590 vs. 4927 (3)	na	na	na	na	na	na	na	na	na	na	na	na	3	1.16 (0.90, 1.49)	0.243	0 (0, 95)	0.839	0.28	Negative
TNF-alpha	rs361525	292 vs. 351 (4)	3	1.06 (0.11, 9.86)	0.961	86 (44,99)	0.001	0.24	4	1.01(0.15, 6.67)	0.988	76(11, 99)	0.016	na	2	0.47 (0.11, 2.03)	0.311	22 (0, 99)	0.257	0.42	Negative
TNF-alpha	rs1799724	845 vs. 832 (4)	4	0.97 (0.78, 1.20)	0.775	4 (0, 93)	0.373	0.05	3	0.99 (0.45, 2.15)	0.972	0 (0, 99)	0.696	0.05	4	0.96 (0.75, 1.24)	0.771	10 (0, 93)	0.344	0.06	Negative
TNF-alpha	rs1799964	1959 vs. 1948 (3§)	3	0.92 (0.83, 1.03)	0.152	0 (0, 93)	0.544	0.18	3	0.83 (0.59, 1.17)	0.290	4 (0, 98)	0.353	0.21	3	0.92 (0.81, 1.05)	0.213	0 (0, 89)	0.734	0.24	Negative
TGFB1	rs1800468	834 vs. 1579 (3)	3	0.90 (0.75, 1.09)	0.276	0 (0, 91)	0.817	0.19	3	0.92 (0.65, 1.31)	0.643	0 (0, 92)	0.813	0.08	3	0.86 (0.66, 1.11)	0.234	0 (0, 95)	0.629	0.42	Negative
TGFB1	rs1800470	998 vs. 1383 (4)	4	1.03 (0.70, 1.51)	0.889	87 (76, 100)	4.44E-05	0.06	3	1.29 (0.75, 2.21)	0.351	78 (29, 100)	0.010	0.70	4	0.95 (0.57, 1.58)	0.847	83 (60, 100)	0.001	0.09	Negative
TGFBR1	rs334354	1012 vs. 1923 (3)	3	1.06 (0.79, 1.40)	0.711	78 (20, 99)	0.010	0.11	3	1.36 (0.94, 1.97)	0.103	29 (0, 98)	0.245	0.68	3	1.00 (0.68, 1.48)	0.994	80 (25, 99)	0.006	0.05	Negative
IGF1	rs6214	1248 vs. 3005 (3*)	3	1.10 (0.95, 1.28)	0.219	34 (0, 99)	0.217	0.29	3	1.08 (0.85, 1.38)	0.517	22 (0, 98)	0.278	0.14	3	1.16 (0.93, 1.44)	0.181	33 (0, 99)	0.223	0.56	Negative
IGFBP3	rs2854746	4484 vs. 7577 (6)	6	0.99 (0.89, 1.09)	0.798	61 (1, 97)	0.024	0.06	6	0.94 (0.83, 1.07)	0.337	38 (0, 95)	0.152	0.28	6	1.01 (0.93, 1.10)	0.633	50 (0, 96)	0.075	0.06	Negative
IGFBP3	rs3110697	3079 vs. 3860 (3*)	3	0.96 (0.83, 1.11)	0.603	67 (0, 100)	0.049	0.13	3	0.95 (0.78, 1.17)	0.644	52 (0, 99)	0.122	0.12	3	1.00 (0.88, 1.14)	0.954	27 (0, 99)	0.255	0.05	Negative
HFE	rs1799945	1495 vs. 2038 (5)	5	1.12 (0.94, 1.34)	0.199	32 (0, 93)	0.211	0.20	5	1.41 (0.84, 2.36)	0.191	14 (0, 91)	0.326	0.26	5	1.11 (0.93, 1.32)	0.257	15 (0, 91)	0.318	0.26	Negative
MTHFR	rs2274976	2860 vs. 3414 (5*§)	3	0.80 (0.53, 1.19)	0.266	79 (73, 100)	0.009	0.51	2	1.50 (0.27, 8.34)	0.641	59 (0, 99)	0.118	0.21	5	0.94 (0.84, 1.05)	0.415	64 (0, 100)	0.027	0.12	Negative
MTRR	rs1532268	4457 vs. 5807 (4*)	4	0.98 (0.90, 1.06)	0.587	38 (0, 96)	0.184	0.08	4	0.90 (0.77, 1.05)	0.192	34 (0, 96)	0.209	0.43	4	1.01 (0.91, 1.12)	0.856	27 (0, 95)	0.251	0.06	Negative
CYP1A1	rs1799814	4389 vs. 4621 (4§)	4	0.89 (0.63, 1.26)	0.513	76 (31, 99)	0.006	0.18	3	1.05 (0.25, 4.43)	0.944	41 (0, 99)	0.184	0.05	4	0.87 (0.60, 1.26)	0.463	76 (34, 99)	0.006	0.41	Negative
CYP1A2	rs2470890	3638 vs. 3885 (4)	4	0.95 (0.84, 1.08)	0.459	68 (3, 99)	0.023	0.19	4	0.89 (0.75, 1.05)	0.162	34 (0, 97)	0.209	0.46	4	0.96 (0.82, 1.13)	0.637	61 (0, 99)	0.053	0.14	Negative
CYP1A2	rs2069514	1283 vs. 1305 (5)	5	1.00 (0.76, 1.32)	0.989	39 (0, 99)	0.163	0.05	4	0.98 (0.69, 1.39)	0.913	0 (0, 91)	0.536	0.05	5	0.96 (0.65, 1.43)	0.854	53 (0, 99)	0.075	0.08	Negative
CYP1B1	rs1056827	3153 vs. 3199 (4)	4	1.03 (0.90, 1.18)	0.657	53 (0, 100)	0.093	0.11	4	1.12 (0.94, 1.33)	0.209	0 (0, 97)	0.536	0.27	4	1.02 (0.86, 1.20)	0.857	50 (0, 100)	0.109	0.09	Negative
CYP2C19	rs4244285	3385 vs. 3498 (5)	5	0.91 (0.80, 1.04)	0.160	34 (0, 97)	0.194	0.28	4	0.84 (0.60, 1.18)	0.304	0 (0, 84)	0.769	0.18	5	0.90 (0.77, 1.07)	0.233	46 (0, 97)	0.119	0.49	Negative
CYP2D6	rs3892097	1153 vs. 1806 (4)	4	1.00 (0.87, 1.15)	0.961	0 (0, 88)	0.694	0.05	4	1.05 (0.74, 1.48)	0.788	0 (0, 92)	0.492	0.06	4	0.99 (0.84, 1.16)	0.869	0 (0, 66)	0.914	0.05	Negative
CYP2E1	rs6413432	3893 vs. 4643 (8)	8	0.88 (0.67, 1.16)	0.353	84(74,98)	1.67E-07	0.05	7	0.83 (0.52, 1.30)	0.411	28 (0, 96)	0.218	0.12	7	0.85 (0.62, 1.16)	0.303	85(75, 98)	0.000	0.05	Negative
GSTA1	rs3957357	806 vs. 1103 (4)	4	1.10 (0.92, 1.32)	0.306	43 (0, 96)	0.153	0.17	4	1.26 (0.90, 1.78)	0.178	45 (0, 96)	0.143	0.45	4	1.05 (0.84, 1.31)	0.687	25 (0, 95)	0.262	0.08	Negative
GSTM1	rs1065411	828 vs.873 (3)	3	1.04 (0.90, 1.21)	0.594	0 (0, 96)	0.692	0.07	2	0.96 (0.78, 1.19)	0.721	0 (0, 99)	0.659	0.06	3	1.12 (0.91, 1.37)	0.277	0 (0, 96)	0.706	0.20	Negative
GSTM3	rs1799735	3505 vs. 4180 (5)	5	0.98 (0.83, 1.15)	0.782	66 (7, 97)	0.019	0.06	5	1.19 (0.79, 1.80)	0.406	43 (0, 97)	0.137	0.24	5	0.96 (0.80, 1.16)	0.660	66 (6, 97)	0.019	0.13	Negative
NAT1	rs4986990	2253 vs. 2768 (3)	3	1.05 (0.81, 1.37)	0.698	0 (0, 79)	0.923	0.06	na	na	na	na	na	na	3	1.05 (0.81, 1.37)	0.694	0 (0, 80)	0.922	0.06	Negative
NAT1	rs1057126	2251 vs. 2747 (3)	3	1.00 (0.90, 1.10)	0.937	0 (0, 95)	0.814	0.05	3	0.95 (0.70, 1.28)	0.734	22 (0, 99)	0.278	0.07	3	1.01 (0.90, 1.14)	0.886	0 (0, 0)	0.998	0.05	Negative
NAT2	rs1799931	2510 vs. 3130 (5)	5	1.01 (0.83, 1.22)	0.955	0 (0, 83)	0.609	0.05	2	0.70 (0.27, 1.81)	0.459	9 (0, 99)	0.295	0.54	5	1.04 (0.83, 1.29)	0.756	0 (0, 70)	0.791	0.07	Negative
NQ01	rs1131341	4436 vs. 4725 (4)	4	0.99 (0.86, 1.15)	0.914	0 (0, 92)	0.509	0.05	2	5.44 (0.94, 31.43)	0.058	0 (0, 98)	0.604	1.00	4	0.97 (0.83, 1.13)	0.699	0 (0, 93)	0.501	0.07	Negative
VDR	rs2238136	4584 vs. 4621 (5*)	5	1.04 (0.96, 1.13)	0.342	20 (0, 91)	0.287	0.13	5	1.16 (0.90, 1.50)	0.258	50 (0, 95)	0.092	0.48	5	1.02 (0.94, 1.11)	0.622	0 (0, 73)	0.767	0.08	Negative
VDR	rs7975232	3301 vs. 3402 (5)	5	1.11 (0.95, 1.30)	0.193	70 (23, 98)	0.010	0.57	5	1.15 (0.92, 1.43)	0.225	53 (0, 98)	0.076	0.64	5	1.10 (0.90, 1.35)	0.329	59 (0, 96)	0.047	0.43	Negative
ABCB1 (MDR1)	rs2032582	1008 vs. 1100 (5)	5	1.07 (0.94, 1.21)	0.314	0 (0, 90)	0.420	0.12	5	1.18 (0.98, 1.42)	0.075	1 (0, 89)	0.403	0.44	5	0.94 (0.74, 1.21)	0.654	6 (0, 90)	0.370	0.07	Negative
ABCB1 (MDR1)	rs868755	2958 vs. 3255 (3)	3	1.06 (0.99, 1.14)	0.109	0 (0, 97)	0.393	0.21	3	1.04 (0.90, 1.20)	0.573	14 (0, 98)	0.312	0.09	3	1.09 (0.96, 1.24)	0.166	25 (0, 98)	0.263	0.36	Negative
ABCB1 (MDR1)	rs3789243	2724 vs. 3385 (4*§)	4	1.03 (0.92, 1.14)	0.625	46 (0, 97)	0.135	0.09	4	0.95 (0.84, 1.07)	0.398	0 (0, 87)	0.627	0.13	4	1.12 (0.93, 1.35)	0.243	56 (0, 98)	0.080	0.48	Negative
ACE	I/D	649 vs. 6556 (4)	4	1.04 (0.91, 1.19)	0.588	0 (0, 84)	0.758	0.05	4	1.17 (0.76, 1.80)	0.470	63 (0, 99)	0.043	0.11	4	1.08 (0.86, 1.36)	0.490	0 (0, 76)	0.860	0.13	Negative
ADIPOQ	rs1501299	2221 vs. 3035 (8)	8	0.96 (0.87, 1.05)	0.343	13 (0, 81)	0.325	0.10	8	1.03 (0.84, 1.26)	0.800	8 (0, 78)	0.366	0.06	8	0.92 (0.81, 1.05)	0.227	22 (0, 82)	0.257	0.32	Negative
ADIPOQ	rs266729	2173 vs. 2582 (5)	5	0.92 (0.79, 1.07)	0.269	64 (1, 97)	0.025	0.25	5	0.97 (0.79, 1.20)	0.781	0 (0, 93)	0.530	0.06	5	0.88 (0.72, 1.08)	0.224	65 (5, 97)	0.021	0.59	Negative
ADIPOR1	rs7539542	1098 vs. 1301 (3)	3	1.01 (0.84, 1.21)	0.950	53 (0, 98)	0.122	0.05	3	1.03 (0.74, 1.43)	0.861	66 (0, 99)	0.053	0.06	3	1.05 (0.85, 1.29)	0.669	0 (0, 90)	0.733	0.07	Negative
ALOX12	rs1126667	3736 vs. 4610 (7*)	7	1.08 (0.93, 1.25)	0.316	78 (61, 98)	1.15E-04	0.41	7	1.11 (0.92, 1.35)	0.268	53 (0, 93)	0.045	0.47	7	1.07 (0.86, 1.34)	0.530	78 (66, 98)	1.10E-04	0.30	Negative

APE1	rs1048945	3224 vs. 3369 (3)	3	0.89 (0.63, 1.25)	0.495	41 (0, 99)	0.185	0.15	3	0.40 (0.08, 1.87)	0.243	0 (0, 97)	0.380	0.25	3	0.90 (0.64, 1.26)	0.533	39 (0, 99)	0.196	0.21	Negative
ARLTS1	rs3430134	1010 vs. 1862 (3)	3	0.98 (0.56, 1.71)	0.947	0 (0, 87)	0.810	0.05	na	na	na	na	na	na	3	0.98 (0.56, 1.71)	0.947	0 (0, 87)	0.808	0.05	Negative
ATM	rs1801516	3477 vs. 6951 (3*)	3	0.99 (0.91, 1.09)	0.885	0 (0, 98)	0.437	0.05	3	0.95 (0.70, 1.30)	0.752	0 (0, 98)	0.418	0.07	3	1.00 (0.90, 1.11)	0.989	0 (0, 98)	0.380	0.05	Negative
BLM	ASH	1741 vs. 11043 (3)	3	1.89 (0.75, 4.75)	0.178	59 (0, 99)	0.087	1.00	na	na	na	na	na	na	3	1.89 (0.75, 4.80)	0.179	59 (0, 99)	0.085	0.84	Negative
BMP4	rs17563	3505 vs. 2518 (4)	4	0.95 (0.86, 1.06)	0.368	40 (0, 96)	0.174	0.17	4	0.93 (0.83, 1.05)	0.270	0 (0, 93)	0.412	0.25	4	0.94 (0.81, 1.10)	0.465	25 (0, 95)	0.261	0.16	Negative
CBS	rs5742905	2197 vs. 2579 (7)	6	1.04 (0.87, 1.25)	0.632	0 (0, 98)	0.530	0.06	4	0.88 (0.39, 2.01)	0.770	0 (0, 60)	0.878	0.06	7	1.05 (0.87, 1.26)	0.618	0 (0, 95)	0.620	0.09	Negative
CDKN1A	rs1059234	1036 vs. 1512 (3)	3	0.93 (0.81, 1.08)	0.347	0 (0, 95)	0.545	0.13	3	0.97 (0.73, 1.30)	0.843	0 (0, 99)	0.424	0.06	3	0.88 (0.72, 1.08)	0.225	0 (0, 97)	0.444	0.35	Negative
CDKN1A	rs1801270	1038 vs. 1513 (3)	3	0.81 (0.57, 1.13)	0.209	65 (0, 100)	0.057	0.65	3	0.97 (0.40, 2.34)	0.948	28 (0, 99)	0.250	0.06	3	0.76 (0.52, 1.12)	0.167	61 (0, 100)	0.075	0.93	Negative
SMAD7	rs4939827	43924 vs. 44264 (21*)	21	0.96 (0.90, 1.02)	0.213	85 (81, 96)	2.39E-19	0.96	21	0.95 (0.86, 1.05)	0.306	81 (67, 95)	1.36E-13	0.99	21	0.93 (0.86, 1.00)	0.084	79 (73, 95)	1.35E-11	1.00	Null
WRN	rs2230009	7662 vs. 7908(4*)	4	0.94(0.85,1.03)	0.170	0(0,0)	0.984	0.15	4	0.76(0.48,1.23)	0.266	0(0,88)	0.483	0.17	4	0.94(0.86,1.04)	0.231	0(0,53)	0.926	0.24	Null
MTRR	rs162036	6473 vs. 7859 (4*)	4	1.05 (0.93, 1.17)	0.439	49 (0, 98)	0.116	0.15	4	1.18 (0.89, 1.58)	0.248	0 (0, 95)	0.489	0.20	4	1.05 (0.94, 1.18)	0.410	41 (0, 98)	0.168	0.22	Null
CDH1	rs16260	7261 vs. 7230(10)	10	1.13(0.97,1.31)	0.105	78(63,95)	7.07E-06	0.12	10	1.03(0.81,1.32)	0.792	51(0,97)	0.030	0.13	10	1.20(0.99,1.44)	0.059	76(55,96)	1.94E-05	0.30	Null
ALDH2	rs671	5561 vs. 8415(14)	14	1.03(0.87,1.23)	0.718	77(56,94)	2.92E-07	0.05	14	1.12(0.75,1.66)	0.584	72(32,88)	6.16E-05	0.05	14	1.01(0.84,1.21)	0.916	67(43,93)	1.78E-04	0.08	Null
MGMT	rs12917	8296 vs. 11815(9*)	8	1.00(0.93,1.07)	0.988	2(0,85)	0.413	0.05	7	1.34(1,10.81)	0.051	23(0,93)	0.253	0.81	9	0.96(0.87,1.05)	0.350	33(0,94)	0.154	0.23	Null
OGG1	rs1052133	8090 vs. 10486 (21)	21	1.09(0.99,1.19)	0.063	65(60,93)	2.00E-05	0.82	21	1.09(0.94,1.25)	0.257	36(0,93)	0.053	0.60	21	1.12(0.99,1.26)	0.072	65(52,91)	2.54E-05	0.87	Null
XRCC1	rs1799782	7643 vs. 11883(25)	24	1.07(0.95,1.21)	0.265	64(48,89)	1.02E-05	0.53	22	1.13(0.91,1.40)	0.277	27(0,84)	0.124	0.36	25	1.07(0.94,1.21)	0.290	57(33,84)	2.33E-04	0.57	Null
XRCC1	rs25489	6510 vs. 7889(10)	9	1.02(0.91,1.14)	0.723	0(0,71)	0.789	0.06	7	1.05(0.59,1.87)	0.869	0(0,73)	0.597	0.06	10	1.00(0.90,1.11)	0.963	0(0,75)	0.625	0.05	Null
XRCC3	rs861539	7995 vs. 9886(21)	21	1.10(0.96,1.26)	0.169	85(79,97)	2.95E-19	0.43	21	1.1(0.92,1.31)	0.280	55(36,91)	0.001	0.12	21	1.10(0.92,1.31)	0.310	84(84,97)	3.19E-17	0.36	Null
IL6	rs1800795	7858 vs. 9345(13§**)	13	1.03(0.93,1.14)	0.572	74(70,96)	4.62E-06	0.05	13	1.01(0.88,1.17)	0.866	57(28,96)	0.006	0.08	13	1.04(0.92,1.17)	0.580	66(46,94)	4.33E-04	0.06	Null
IL8	rs4073	6060 vs. 7435(12**)	11	1.04(0.96,1.14)	0.303	57(14,92)	0.010	0.20	11	1.05(0.95,1.17)	0.340	24(0,75)	0.215	0.21	12	1.07(0.93,1.22)	0.362	65(40,93)	0.001	0.43	Null
PPAR-gamma	rs3856806	8098 vs. 11100(11*)	9	1.05(0.97,1.14)	0.193	20(0,84)	0.263	0.20	9	0.87(0.55,1.37)	0.549	60(13,92)	0.010	0.19	11	1.06(0.99,1.14)	0.073	0(0,68)	0.551	0.39	Null
PPAR-gamma	rs1801282	15855 vs. 24348(22§**)	19	1.00(0.93,1.08)	0.978	48(5,90)	0.019	0.05	19	0.84(0.68,1.04)	0.102	0(0,57)	0.796	0.35	22	0.99(0.91,1.08)	0.870	49(18,88)	0.006	0.07	Null
PTGS2/COX2	rs689466	7790 vs. 11652(12)	12	0.95(0.84,1.08)	0.437	80(80,98)	8.22E-08	0.31	12	0.95(0.79,1.13)	0.549	28(0,85)	0.168	0.12	12	0.93(0.80,1.09)	0.380	81(81,98)	1.39E-08	0.68	Null
PTGS2/COX2	rs5275	12396 vs. 15884(13*)	13	1.01(0.97,1.04)	0.672	0(0,71)	0.482	0.07	13	1.02(0.94,1.11)	0.616	14(0,81)	0.301	0.08	13	1.01(0.96,1.06)	0.790	0(0,45)	0.791	0.07	Null
PTGS2/COX2	rs5277	5740 vs. 7509(7)	7	0.98(0.92,1.05)	0.620	0(0,75)	0.786	0.07	7	0.83(0.66,1.04)	0.106	0(0,82)	0.594	0.36	7	1.00(0.93,1.08)	0.974	0(0,65)	0.881	0.05	Null
TGFBR1	rs11466445	6705 vs. 8162(12**)	12	1.13(0.99,1.30)	0.080	62(33,93)	0.003	0.59	12	1.12(0.74,1.71)	0.589	27(0,93)	0.184	0.09	12	1.12(0.99,1.28)	0.079	49(0,90)	0.029	0.73	Null
IGF1	CA-repeat	9335 vs. 11089(9)	9	0.99(0.92,1.07)	0.866	62(32,96)	0.007	0.06	9	1.02(0.94,1.11)	0.642	8(0,82)	0.369	0.08	9	0.95(0.79,1.13)	0.550	86(88,99)	1.69E-09	0.43	Null
IGFBP3	rs2854744	10880 vs. 15046(12)	12	1.01(0.97,1.05)	0.655	0(0,2)	0.973	0.07	12	1.02(0.96,1.08)	0.561	0(0,64)	0.651	0.10	12	1.00(0.95,1.07)	0.878	0(0,0)	0.995	0.05	Null
MTR	rs1805087	18831 vs. 24357 (28*§)	28	0.95 (0.88, 1.04)	0.279	78 (75, 93)	1.55E-14	0.07	28	0.98 (0.85, 1.14)	0.843	38 (0, 71)	0.024	0.07	27	0.98 (0.94, 1.02)	0.424	0 (0, 46)	0.490	0.16	Null
MTRR	rs1801394	10175 vs. 15506 (21)	21	1.04 (1.00, 1.09)	0.077	28 (0, 73)	0.114	0.47	21	1.06 (0.99, 1.14)	0.091	18 (0, 80)	0.230	0.50	21	1.05 (0.97, 1.14)	0.193	37(0, 74)	0.048	0.49	Null
TS	rs34743033	5860 vs. 7540 (11)	11	0.96 (0.87, 1.05)	0.378	57 (16, 92)	0.010	0.20	11	0.93 (0.83, 1.04)	0.203	10 (0, 87)	0.351	0.29	11	0.96 (0.84, 1.10)	0.540	60 (19, 92)	0.005	0.21	Null
TS	rs34489327	5786 vs. 6856 (8)	8	0.99 (0.94, 1.05)	0.839	12 (0, 86)	0.334	0.06	8	0.95 (0.82, 1.10)	0.499	32 (0, 87)	0.172	0.14	8	1.00 (0.93, 1.07)	0.985	0 (0, 83)	0.442	0.05	Null
APC	rs1801166	7161 vs. 8140 (7)	7	1.27 (0.89, 1.82)	0.191	31 (0, 95)	0.195	0.35	na	na	na	na	na	na	7	1.27 (0.88, 1.83)	0.200	31 (0, 95)	0.188	0.59	Null
APC	rs459552	21929 vs. 20943 (22**)	22	1.00 (0.94, 1.07)	0.943	69 (65, 93)	8.56E-07	0.05	22	1.02 (0.86, 1.21)	0.835	69 (56, 90)	9.74E-07	0.07	22	0.99 (0.94, 1.05)	0.701	42 (3, 85)	0.022	0.08	Null
MLH1	rs1799977	15398 vs. 16730 (20*)	19	0.99 (0.94, 1.05)	0.846	50 (17, 94)	0.008	0.07	19	0.96 (0.89, 1.04)	0.332	4 (0, 80)	0.410	0.19	20	1.01 (0.94, 1.08)	0.811	43 (1, 90)	0.023	0.07	Null
MLH1	rs1800734	21837 vs. 18831 (15*§)	15	1.04 (0.99, 1.09)	0.082	30 (0, 92)	0.131	0.38	15	1.06 (0.94, 1.19)	0.381	26 (0, 91)	0.166	0.24	15	1.05 (0.99, 1.12)	0.126	38 (0, 96)	0.065	0.67	Null
CYP1A1	rs4646903	9725 vs. 12104 (20)	20	0.95(0.87, 1.04)	0.282	49 (27, 94)	0.007	0.08	19	0.79 (0.61, 1.03)	0.078	50 (17, 90)	0.007	0.41	20	0.97 (0.88, 1.06)	0.448	37 (0, 92)	0.049	0.10	Null
CYP1A2	rs762551	9669 vs. 10852 (16)	16	1.01 (0.95, 1.07)	0.835	40 (0, 90)	0.052	0.06	16	0.99 (0.90, 1.09)	0.849	0 (0, 62)	0.599	0.06	16	1.01(0.93, 1.10)	0.819	46 (4, 94)	0.023	0.06	Null
CYP1B1	rs1056836	10318 vs. 11251 (12)	12	1.01 (0.98, 1.05)	0.456	0 (0, 59)	0.721	0.07	12	1.06 (0.98, 1.14)	0.140	13(0, 84)	0.319	0.41	12	0.99 (0.93, 1.05)	0.740	0 (0, 59)	0.790	0.06	Null
CYP2C9	rs1057910	15444 vs. 17426 (13*)	13	0.99 (0.91, 1.08)	0.803	48 (0, 95)	0.028	0.06	11	0.75 (0.54, 1.06)	0.107	28 (0, 89)	0.176	0.58	13	1.01 (0.92, 1.10)	0.902	47 (0, 95)	0.031	0.06	Null
CYP2E1	rs2031920	5216 vs. 6026 (11§)	11	1.10 (0.95, 1.28)	0.210	53 (3, 88)	0.020	0.36	10	1.42 (1.00, 2.03)	0.052	32 (0, 92)	0.149	0.60	11	1.05 (0.91, 1.22)	0.500	39 (0, 86)	0.091	0.19	Null
CYP2E1	rs3813867	5035 vs. 6688 (10)	10	0.99 (0.75, 1.31)	0.947	82 (65, 95)	1.45E-07	0.45	8	1.38 (0.94, 2.03)	0.100	0 (0, 90)	0.527	0.38	10	0.98 (0.72, 1.33)	0.874	81 (62, 95)	2.43E-07	0.70	Null
GSTP1	rs1695	16373 vs. 20872 (38*)	38	0.99 (0.94, 1.05)	0.806	50 (39, 84)	2.49E-04	0.05	38	0.95 (0.85, 1.06)	0.371	38 (10, 90)	0.012	0.36	38	1.02 (0.94, 1.09)	0.673	54 (50, 89)	3.69E-05	0.16	Null
GSTP1	rs1138272	12144 vs. 12586	13	0.98 (0.90, 1.07)	0.616	37 (0, 93)	0.084	0.07	13	0.82 (0.62, 1.09)	0.182	12 (0, 85)	0.327	0.26	13	0.99 (0.91, 1.08)	0.814	22 (0, 90)	0.223	0.06	Null

		(13*§)																			
NAT2	slow/rapid	8057 vs. 10431 (45)	19	1.02 (0.91, 1.14)	0.789	79 (59, 92)	3.91E-11	0.10	19	1.01 (0.88, 1.15)	0.912	47 (10, 88)	0.012	0.06	19	1.00 (0.85, 1.17)	0.988	79 (42, 87)	7.53E-11	0.05	Null
CX3CR1	rs3732378	6110 vs. 6335(4*)	4	1.01(0.93,1.09)	0.841	16(0,99)	0.310	0.06	4	1.06(0.87,1.30)	0.569	0(0,53)	0.850	0.09	4	1.00(0.90,1.12)	0.977	36(0,99)	0.195	0.05	Null
EPHX1	rs1051740	11718 vs. 13560(19*)	19	1.03(0.98,1.09)	0.250	43(0,86)	0.026	0.19	19	1.09(0.95,1.25)	0.224	53(23,88)	0.004	0.53	19	1.03(0.96,1.10)	0.385	39(0,88)	0.041	0.21	Null
EPHX1	rs2234922	6570 vs. 7960(14*)	14	0.97(0.90,1.05)	0.464	31(0,86)	0.124	0.11	14	1.12(0.89,1.42)	0.325	35(0,81)	0.093	0.33	14	0.95(0.88,1.02)	0.146	7(0,79)	0.358	0.40	Null
ERCC2	rs1799793	6722 vs. 9585(11)	9	1.14(0.98,1.33)	0.092	85(61,95)	6.91E-09	0.98	9	1.44(0.95,2.18)	0.083	92(76,97)	6.36E-19	1.00	11	1.02(0.96,1.09)	0.506	0(0,52)	0.781	0.10	Null
ERCC2	rs13181	10239 vs. 13833(24*)	24	1.00(0.93,1.08)	0.948	57(54,92)	4.55E-04	0.07	24	0.97(0.83,1.14)	0.730	59(60,94)	1.99E-04	0.06	24	1.01(0.95,1.08)	0.705	15(0,76)	0.250	0.12	Null
ERCC4	rs1800067	6896 vs. 7431(8*)	8	0.98(0.89,1.07)	0.617	0(0,95)	0.609	0.06	7	0.71(0.44,1.12)	0.139	0(0,91)	0.560	0.30	8	0.99(0.90,1.09)	0.869	0(0,94)	0.618	0.06	Null
ESR1	rs9340799	5043 vs. 6090(7)	7	0.98(0.93,1.04)	0.525	0(0,90)	0.540	0.08	7	0.97(0.85,1.11)	0.673	10(0,94)	0.356	0.08	7	0.99(0.89,1.10)	0.843	23(0,97)	0.252	0.06	Null
EXO1	rs4149963	6597 vs. 6842(5*)	5	1.07(0.89,1.29)	0.485	69(34,99)	0.012	0.19	5	0.90(0.49,1.63)	0.719	34(0,98)	0.196	0.08	5	1.06(0.89,1.26)	0.505	59(0,99)	0.047	0.24	Null
EXO1	rs9350	5459 vs. 5926(4*)	4	0.94(0.80,1.09)	0.409	65(0,99)	0.035	0.23	4	0.81(0.52,1.26)	0.348	64(0,99)	0.039	0.42	4	0.99(0.88,1.12)	0.911	32(0,99)	0.220	0.06	Null
HIF1A	rs11549465	6036 vs. 8066(5*)	5	0.99(0.83,1.18)	0.875	59(0,100)	0.089	0.05	2	1.22(0.82,1.81)	0.337	18(0,100)	0.271	0.22	3	1.06(0.85,1.31)	0.605	66(18,100)	0.020	0.27	Null
ICAM1	rs5498	6391 vs. 6611(6*)	6	0.94(0.83,1.07)	0.354	74(57,99)	0.002	0.09	6	1.00(0.86,1.17)	0.960	41(0,98)	0.132	0.07	6	0.89(0.75,1.06)	0.184	67(25,98)	0.009	0.28	Null
IRS	rs1801278	9726 vs. 10756(9**)	8	1.04(0.93,1.17)	0.476	38(0,89)	0.126	0.23	8	1.04(0.69,1.58)	0.840	0(0,0)	0.985	0.05	9	1.07(0.96,1.20)	0.227	43(0,89)	0.079	0.66	Null
MBL2	rs1800450	6362 vs. 6511(5*)	5	1.04(0.95,1.15)	0.373	23(0,99)	0.266	0.12	4	1.27(0.78,2.06)	0.333	57(0,95)	0.070	0.48	5	1.02(0.90,1.16)	0.745	36(0,99)	0.181	0.08	Null
miR	rs2910164	5127 vs. 4110(8)	8	0.80(0.47,1.36)	0.413	98(95,99)	3.01E-89	1.00	8	0.81(0.49,1.35)	0.427	94(87,99)	1.45E-23	0.83	8	0.75(0.36,1.57)	0.451	98(92,99)	1.34E-67	1.00	Null
MSH6	rs1800932	12214 vs. 10598(9)	9	0.96(0.90,1.03)	0.277	46(0,92)	0.064	0.22	9	0.88(0.75,1.02)	0.080	3(0,86)	0.411	0.40	9	0.97(0.89,1.05)	0.439	47(0,91)	0.056	0.19	Null
MSH6	rs1800935	14234 vs. 12089(11*)	11	0.97(0.94,1.01)	0.139	1(0,76)	0.435	0.20	11	0.97(0.89,1.06)	0.556	4(0,82)	0.405	0.11	11	0.96(0.91,1.02)	0.209	24(0,77)	0.216	0.38	Null
MTHFD1	rs2236225	11673 vs. 15250(10*)	10	1.00(0.96,1.03)	0.847	0(0,75)	0.559	0.05	10	0.98(0.93,1.04)	0.571	0(0,74)	0.531	0.10	10	1.01(0.95,1.06)	0.833	0(0,65)	0.609	0.07	Null
PLA2G2A	rs11677	5887 vs. 6774(4*)	4	0.96(0.86,1.07)	0.460	48(0,96)	0.125	0.11	4	0.85(0.63,1.15)	0.291	0(0,92)	0.602	0.18	4	0.96(0.85,1.08)	0.519	45(0,96)	0.144	0.16	Null
15q13.3	rs4779584	6994 vs. 7280(7*)	7	1.05(0.94,1.17)	0.398	69(30,96)	0.003	0.30	7	1.07(0.98,1.17)	0.146	0(0,98)	0.461	0.36	7	1.02(0.86,1.21)	0.804	72(30,94)	0.001	0.09	Null
SHBG	rs6259	6312 vs. 6820(5*)	5	0.87(0.61,1.25)	0.454	95(78,99)	2.08E-17	0.81	5	0.84(0.20,3.51)	0.816	95(78,99)	2.58E-18	0.32	5	0.90(0.72,1.12)	0.348	84(43,97)	6.56E-05	0.77	Null
TCF7L2	rs7903146	5059 vs. 21289(6*)	6	1.07(0.96,1.18)	0.210	59(0,95)	0.032	0.37	6	1.11(0.93,1.33)	0.259	33(0,95)	0.192	0.30	6	1.06(0.94,1.20)	0.319	54(0,92)	0.056	0.33	Null
UGT1A6	rs1105879	7334 vs. 7774(5*)	5	1.01(0.97,1.06)	0.598	2(0,79)	0.397	0.06	5	1.03(0.93,1.14)	0.565	0(0,92)	0.459	0.09	5	1.01(0.93,1.09)	0.874	23(0,90)	0.269	0.06	Null
WRN	rs1346044	8548 vs. 8816(5*)	5	1.02(0.95,1.08)	0.644	35(0,96)	0.185	0.09	5	0.91(0.70,1.18)	0.478	72(48,99)	0.006	0.31	5	1.04(0.98,1.10)	0.212	0(0,89)	0.467	0.25	Null
XPC	rs2228001	7650 vs. 9769(13*)	13	1.03(0.96,1.11)	0.361	46(0,89)	0.036	0.16	13	1.01(0.90,1.14)	0.861	32(0,89)	0.125	0.06	13	1.06(0.95,1.18)	0.300	58(19,88)	0.005	0.45	Null
MUTYH	rs3219484	16671 vs. 14985 (13*)	13	1.03 (0.97, 1.09)	0.419	0 (0, 52)	0.611	0.10	12	0.87 (0.63, 1.20)	0.394	0 (0, 63)	0.771	0.14	13	1.03 (0.97, 1.10)	0.294	0 (0, 47)	0.682	0.15	Null
MUTYH	rs3219489	17936 vs. 19154 (20)	20	1.04 (0.98, 1.10)	0.186	60 (53, 95)	3.28E-04	0.38	20	1.09 (0.97, 1.22)	0.164	41 (0, 84)	0.028	0.54	20	1.03 (0.97, 1.11)	0.331	53 (42, 96)	0.003	0.29	Null
XRCC2	rs3218536	5977 vs. 6174 (4*)	4	1.07 (0.96, 1.20)	0.228	22 (0, 99)	0.280	0.18	4	0.81 (0.45, 1.47)	0.485	40 (0, 98)	0.169	0.14	4	1.09 (0.99, 1.20)	0.080	0 (0, 98)	0.545	0.41	Null
IL16	rs4072111	6160 vs. 6643 (4*)	4	1.04 (0.95, 1.14)	0.369	15 (0, 98)	0.315	0.11	4	1.15 (0.82, 1.61)	0.423	16 (0, 97)	0.313	0.16	4	1.04 (0.95, 1.13)	0.422	7 (0, 98)	0.360	0.16	Null
PTGS1/COX1	rs5789	8164 vs. 10969 (6*)	3	1.02 (0.86, 1.21)	0.856	75 (7, 99)	0.019	0.07	3	0.93 (0.68, 1.27)	0.640	0 (0, 95)	0.575	0.07	3	1.03 (0.84, 1.26)	0.778	76 (13, 99)	0.015	0.12	Null
PTGS1/COX1	rs1236913	9511 vs. 11189 (6*)	4	0.99 (0.90, 1.08)	0.790	0 (0, 88)	0.582	0.05	4	0.84 (0.46, 1.54)	0.582	25 (0, 98)	0.259	0.13	6	1.01 (0.93, 1.09)	0.884	0 (0, 79)	0.649	0.06	Null
PTGS2/COX2	rs2206593	5391 vs. 6096 (3*)	3	0.94 (0.81, 1.10)	0.458	51 (0, 100)	0.131	0.19	3	1.01 (0.77, 1.31)	0.963	0 (0, 99)	0.577	0.05	3	0.95 (0.82, 1.10)	0.470	44 (0, 100)	0.170	0.21	Null
IGFBP1	rs4619	6537 vs. 6826 (4*)	4	1.03 (0.98, 1.08)	0.299	0 (0, 88)	0.768	0.13	4	1.06 (0.96, 1.17)	0.265	0 (0, 0)	0.977	0.21	4	1.02 (0.96, 1.10)	0.503	0 (0, 92)	0.662	0.09	Null
MTRR	rs10380	9022 vs. 10910 (7*)	6	1.05 (0.96, 1.14)	0.327	30 (0, 95)	0.207	0.18	6	1.38 (1.04, 1.82)	0.025	0 (0, 90)	0.560	0.69	7	1.05 (0.96, 1.15)	0.327	30 (0, 92)	0.199	0.27	Null
MLH1	rs3774332	7556 vs. 7299 (4*)	4	1.00 (0.88, 1.14)	0.967	50 (0, 97)	0.110	0.05	4	0.99 (0.66, 1.49)	0.969	14 (0, 93)	0.322	0.05	4	1.01 (0.89, 1.13)	0.930	38 (0, 96)	0.187	0.06	Null
CYP1B1	rs10012	5336 vs. 5850 (6)	6	1.00 (0.94, 1.07)	0.917	20 (0, 95)	0.285	0.05	6	1.04 (0.86, 1.25)	0.698	43 (0, 97)	0.115	0.09	6	0.99 (0.92, 1.07)	0.855	0 (0, 83)	0.787	0.06	Null
CYP1B1	rs1800440	11152 vs. 11918 (9*)	9	0.99 (0.92, 1.07)	0.860	49 (0, 91)	0.047	0.06	9	0.95 (0.82, 1.10)	0.490	0 (0, 53)	0.723	0.10	9	1.01 (0.91, 1.11)	0.893	62 (20, 94)	0.007	0.07	Null
CYP17A1	rs743572	5009 vs. 5163 (3)	3	1.04 (0.95, 1.13)	0.407	50 (0, 99)	0.133	0.16	3	0.96 (0.87, 1.07)	0.509	0 (0, 98)	0.492	0.12	3	1.10 (0.96, 1.25)	0.170	57 (0, 99)	0.100	0.63	Null
CYP3A4	rs11773597	5005 vs. 5155 (3)	3	0.99 (0.89, 1.10)	0.843	0 (0, 41)	0.955	0.05	3	0.89 (0.47, 1.68)	0.715	0 (0, 97)	0.619	0.07	3	0.99 (0.88, 1.11)	0.889	0 (0, 77)	0.899	0.05	Null
CYP3A4	rs2740574	5138 vs. 5295 (4)	4	0.92 (0.80, 1.06)	0.236	0 (0, 86)	0.767	0.13	4	0.47 (0.19, 1.14)	0.095	5 (0, 97)	0.367	0.29	4	0.94 (0.82, 1.09)	0.451	0 (0, 21)	0.961	0.14	Null
VDR	rs2228570	16386 vs. 18814 (17**)	17	0.99 (0.94, 1.05)	0.702	59 (35, 93)	0.001	0.08	17	0.98 (0.90, 1.08)	0.706	45 (0, 94)	0.025	0.11	17	0.98 (0.90, 1.07)	0.685	61 (47, 96)	0.001	0.15	Null
ABCB1 (MDR1)	rs2229109	6143 vs. 6104 (5§)	5	0.92 (0.82, 1.03)	0.156	0 (0, 84)	0.673	0.17	5	1.39 (0.56, 3.43)	0.474	0 (0, 87)	0.657	0.19	5	0.91 (0.80, 1.03)	0.119	0 (0, 82)	0.713	0.34	Null
ABCB1 (MDR1)	rs2214102	7865 vs. 7919 (5*)	5	1.07 (0.98, 1.17)	0.116	9 (0, 84)	0.358	0.22	5	1.07 (0.66, 1.73)	0.794	36 (0, 90)	0.181	0.06	5	1.08 (0.99, 1.18)	0.092	0 (0, 80)	0.548	0.42	Null
APE1	rs1130409	5786 vs. 6550 (11)	11	1.08 (0.94, 1.22)	0.277	80 (79, 98)	3.27E-07	0.52	11	1.07 (0.87, 1.32)	0.522	74 (72, 98)	4.07E-	0.18	11	1.11 (0.92, 1.34)	0.288	78 (77, 97)	2.43E-06	0.76	Null

													05								
AURKA	rs2273535	5221 vs. 4806 (5)	5	1.07 (0.94, 1.23)	0.309	68 (19, 98)	0.014	0.31	5	1.19 (0.87, 1.64)	0.274	67 (6, 97)	0.018	0.56	5	1.03 (0.92, 1.15)	0.617	25 (0, 97)	0.254	0.11	Null
CASP8	rs3834129	7023 vs. 10751 (12)	12	0.98 (0.91, 1.06)	0.654	45 (0, 83)	0.046	0.06	12	1.04 (0.91, 1.20)	0.570	41 (0, 87)	0.066	0.30	12	0.93 (0.86, 1.01)	0.083	10 (0, 66)	0.352	0.49	Null
CASR	rs1801725	6000 vs. 6425 (4*)	4	1.05 (0.95, 1.17)	0.325	41 (0, 98)	0.163	0.15	4	1.27 (0.82, 1.96)	0.292	56 (0, 99)	0.079	0.45	4	1.03 (0.95, 1.11)	0.527	0 (0, 96)	0.483	0.11	Null
CASR	rs1042636	9265 vs. 10905 (5*)	5	1.01 (0.94, 1.08)	0.887	0 (0, 65)	0.810	0.06	5	1.04 (0.78, 1.40)	0.776	0 (0, 97)	0.413	0.06	5	1.00 (0.93, 1.08)	0.959	0 (0, 61)	0.834	0.05	Null
COMT	rs4680	9830 vs. 10176 (7*)	7	0.99 (0.94, 1.05)	0.845	42 (0, 94)	0.114	0.07	7	0.99 (0.91, 1.07)	0.724	25 (0, 92)	0.240	0.06	7	0.98 (0.91, 1.06)	0.579	19 (0, 91)	0.282	0.10	Null
PTGS1/COX1	rs3842787	5754 vs. 7916 (4)	3	1.01 (0.88, 1.16)	0.838	4 (0, 99)	0.352	0.05	2	0.85 (0.42, 1.70)	0.638	0 (0, 100)	0.347	0.08	4	1.09 (0.95, 1.24)	0.240	23 (0, 97)	0.274	0.33	Null

* Includes GWAS data from SOCCS
§ Includes GWAS data from Ontario
** Tomlinson 2008 was based on 10 samples
