# Supplementary material for: Systematic meta-analyses, field synopsis and global assessment of the evidence of genetic association studies in colorectal cancer
Source: Gut. 2019 Dec 9;69(8):1460–71. doi: 10.1136/gutjnl-2019-319313 (PMC7398467; doi:10.1136/gutjnl-2019-319313)
Supplement: Supplementary data [file gutjnl-2019-319313supp003.pdf]

Supplementary Figures

Supplementary Figures 1-14: Forest and Egger's plots for 14 positive polymorphisms that had the most credible associations with CRC risk.

Supplementary Figures 15-77: Forest and Egger's plots for 63 polymorphisms that had the less credible associations with CRC risk.

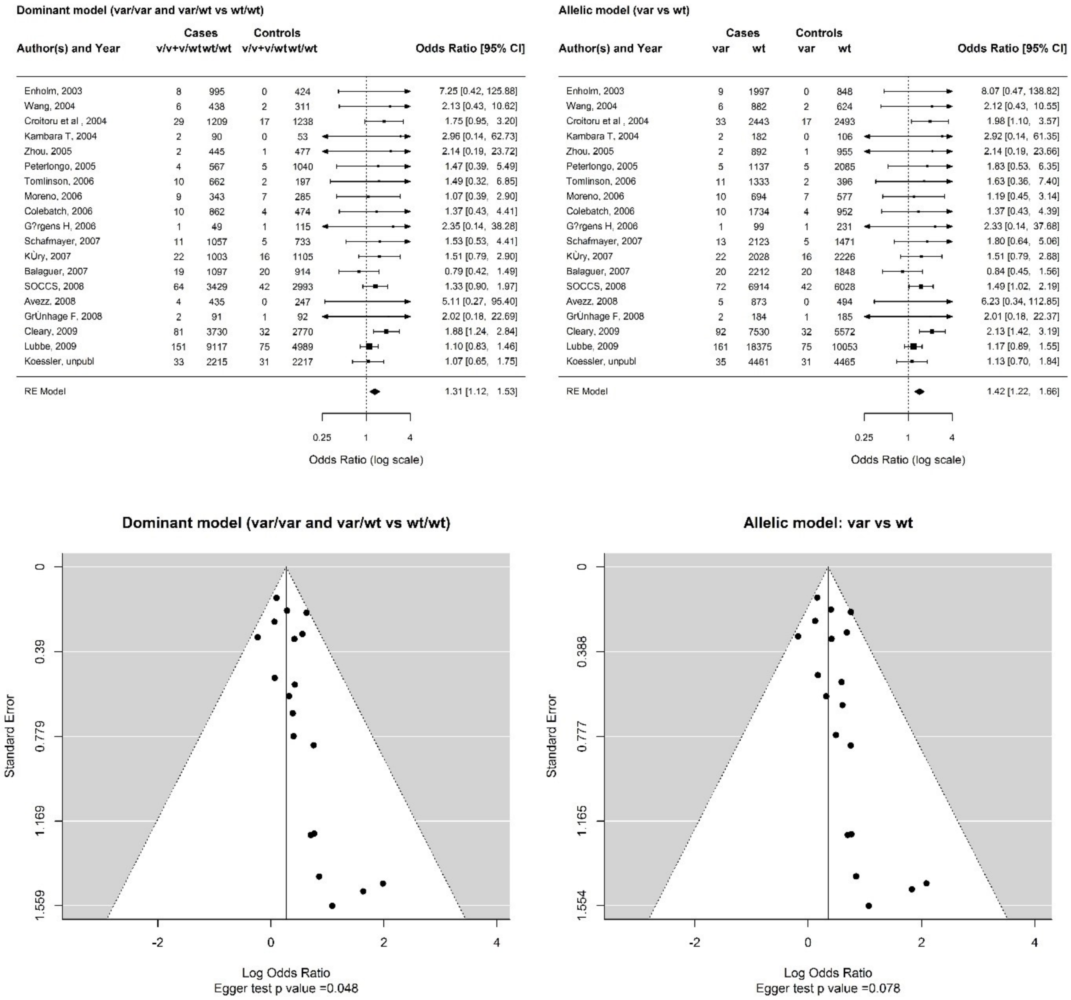

Supplementary Figure 1 Forest and Egger's plots for *MUTYH* (G396D, rs36053993).

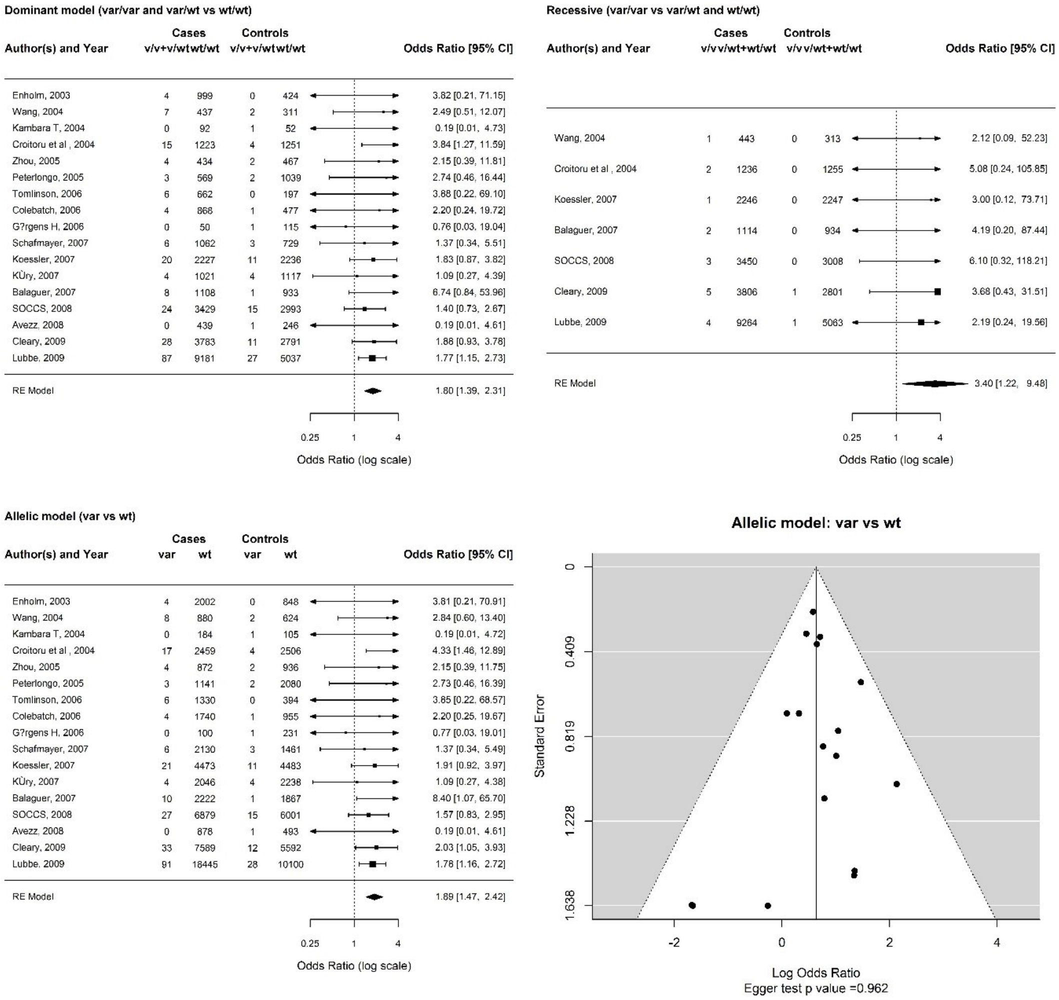

Supplementary Figure 2 Forest and Egger's plots for *MUTYH* (Y179C, rs34612342).

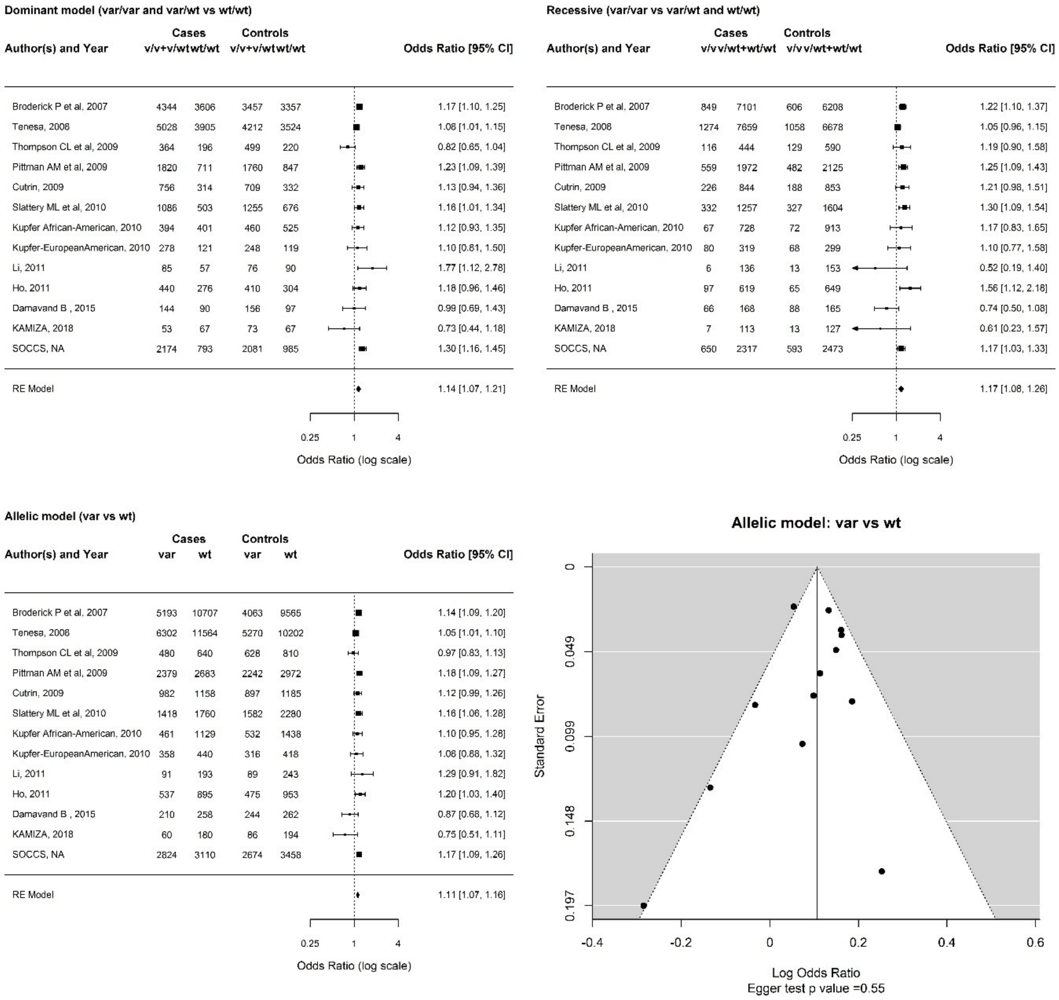

Supplementary Figure 3 Forest and Egger's plots for *SMAD7* (rs12953717).

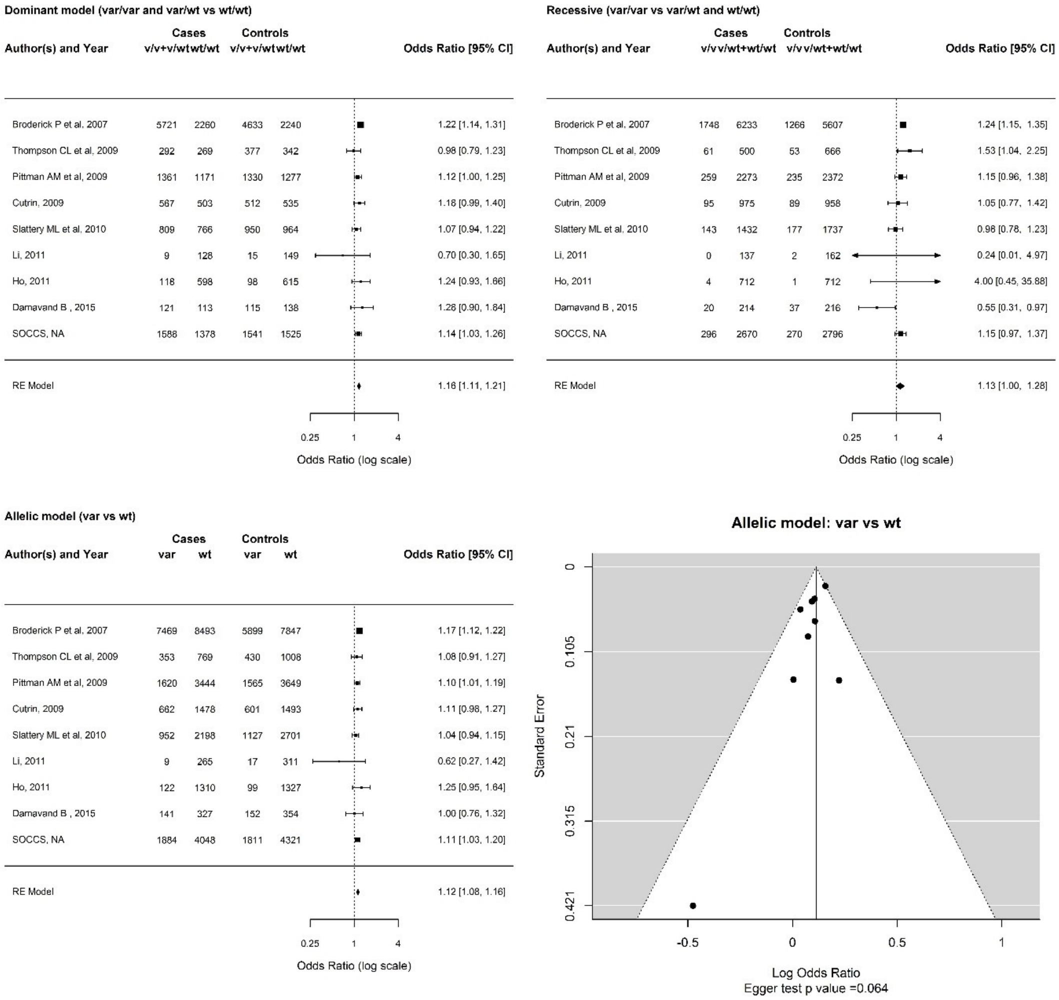

Supplementary Figure 4 Forest and Egger's plots for *SMAD7* (rs4464148).

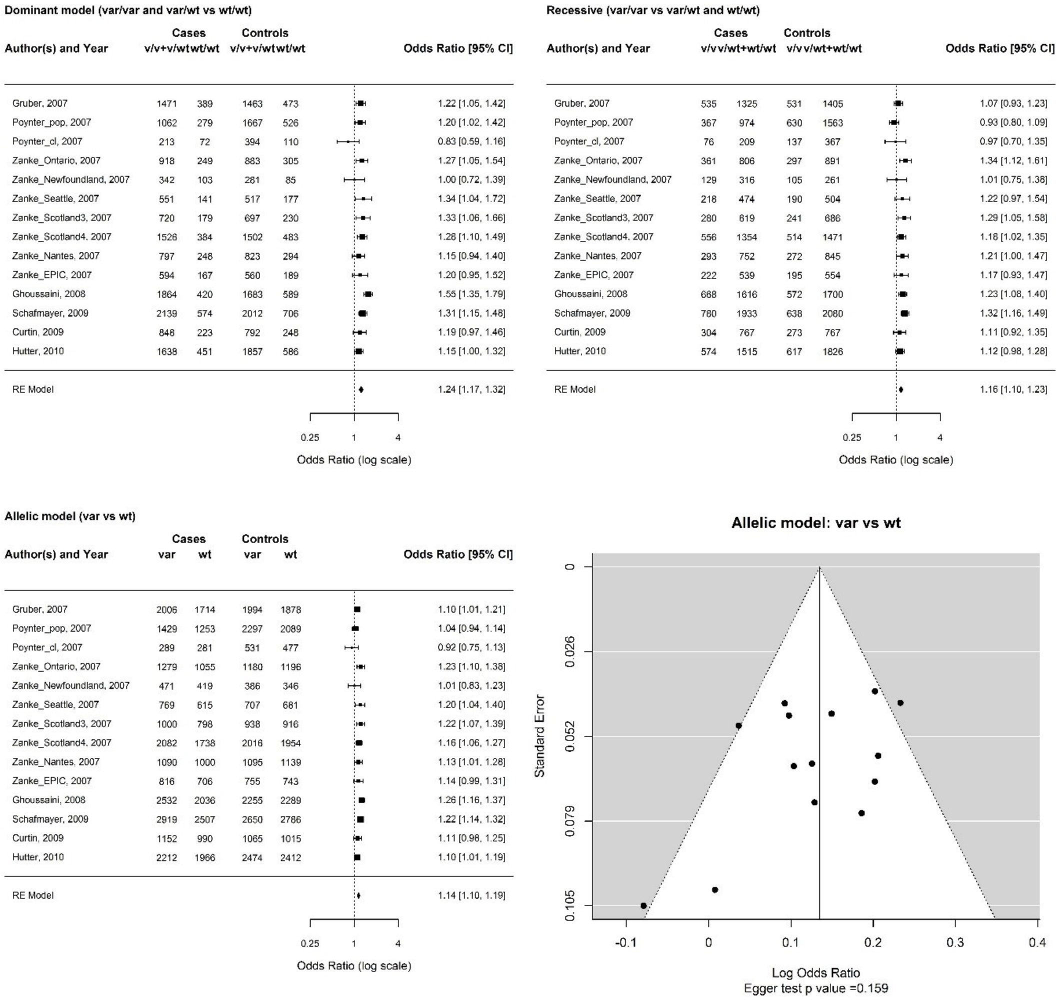

Supplementary Figure 5 Forest and Egger's plots for 8q24 (rs10505477).

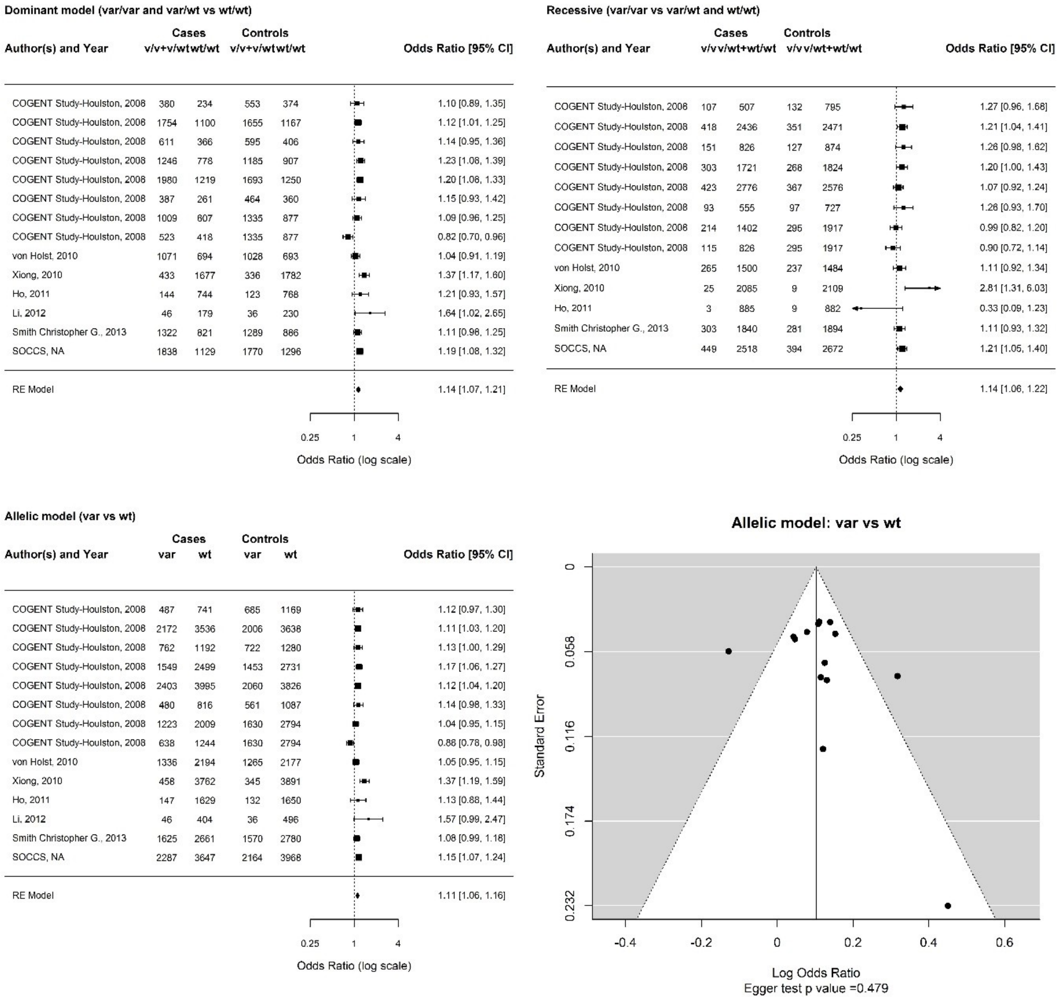

Supplementary Figure 6 Forest and Egger’s plots for 20p12.3 (rs961253).

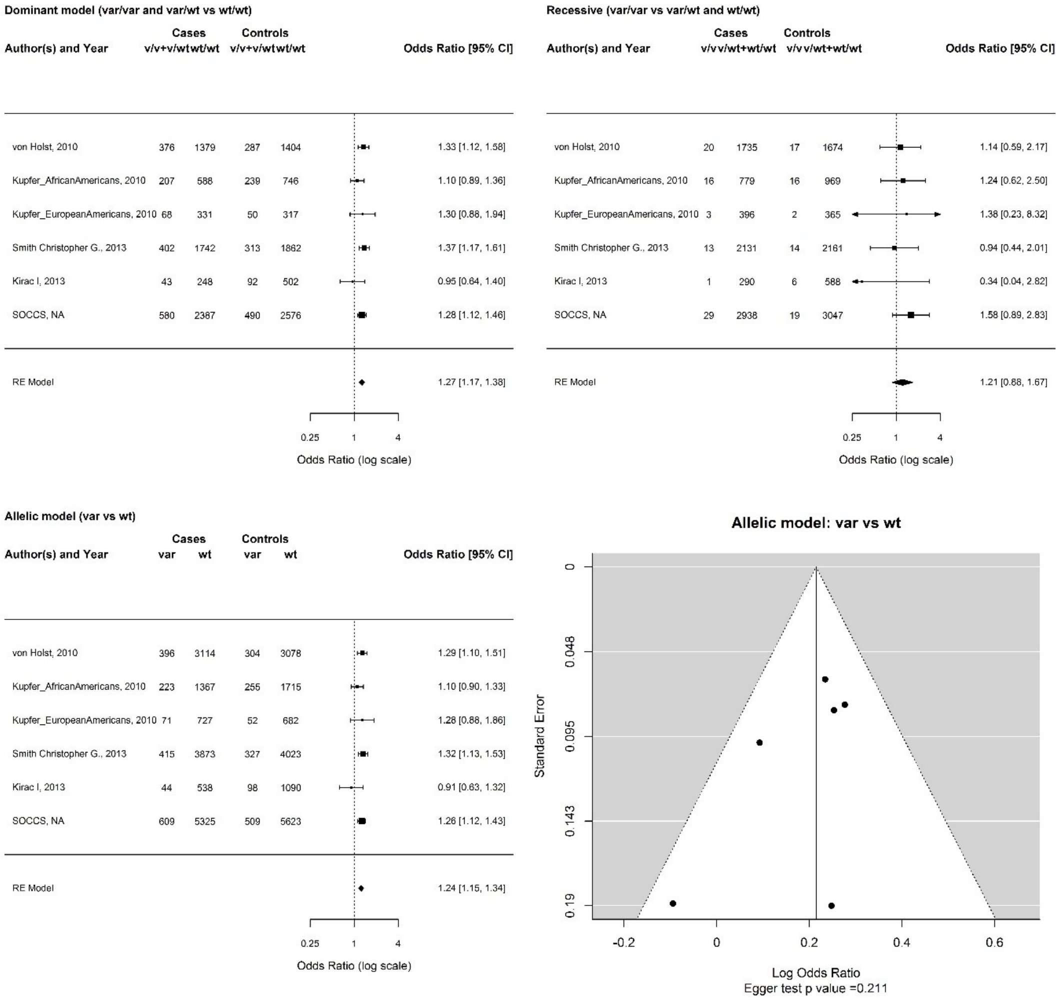

Supplementary Figure 7 Forest and Egger's plots for 8q23.3 (rs16892766).

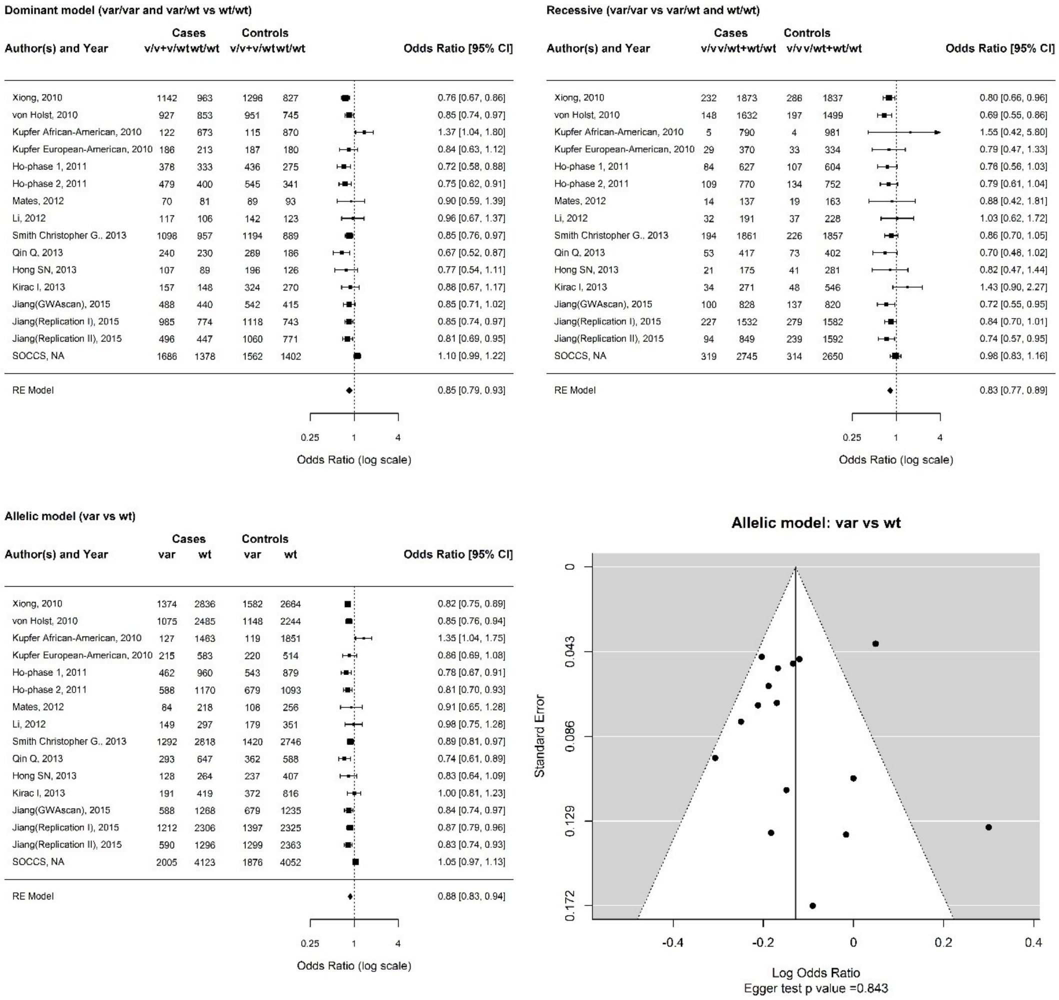

Supplementary Figure 8 Forest and Egger’s plots for 10p14 (rs10795668).

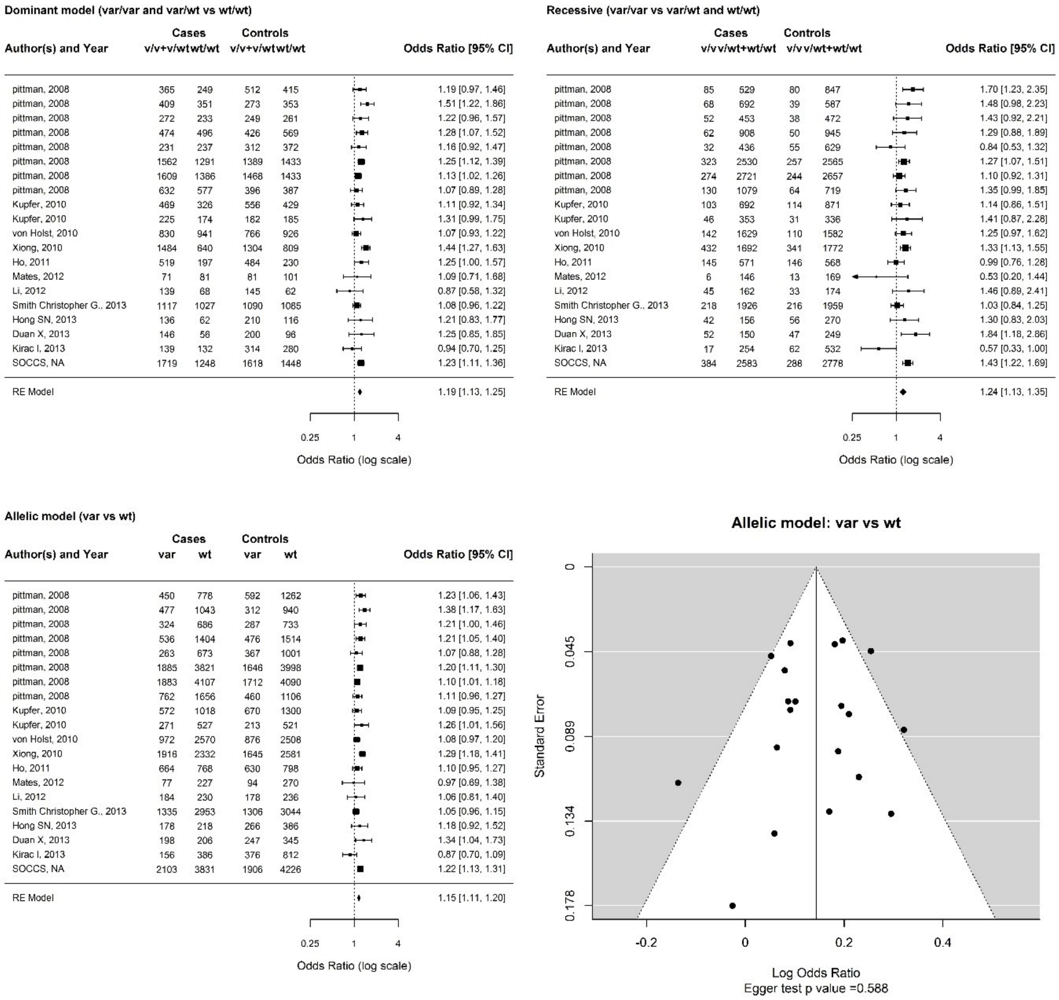

Supplementary Figure 9 Forest and Egger's plots for 11q23.1 (rs3802842).

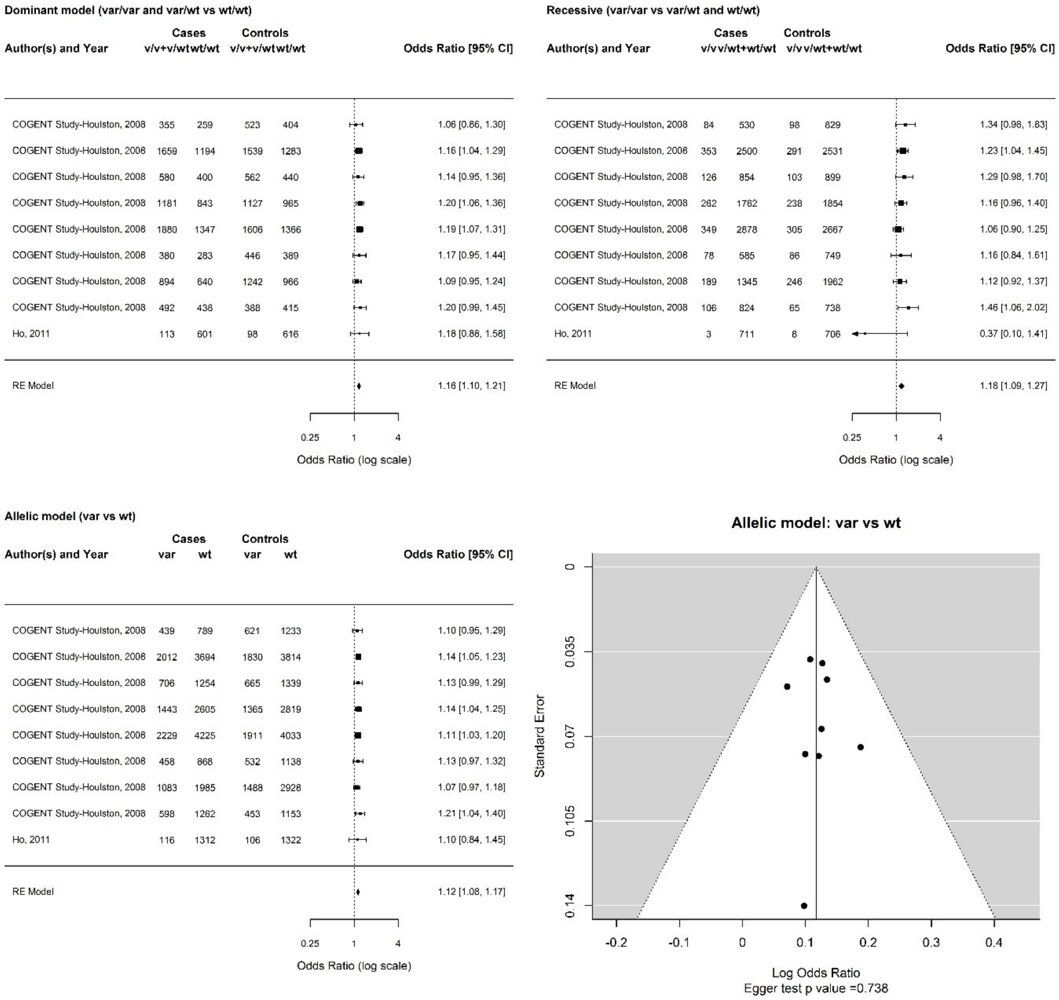

Supplementary Figure 10 Forest and Egger’s plots for *BMP2* (rs355527).

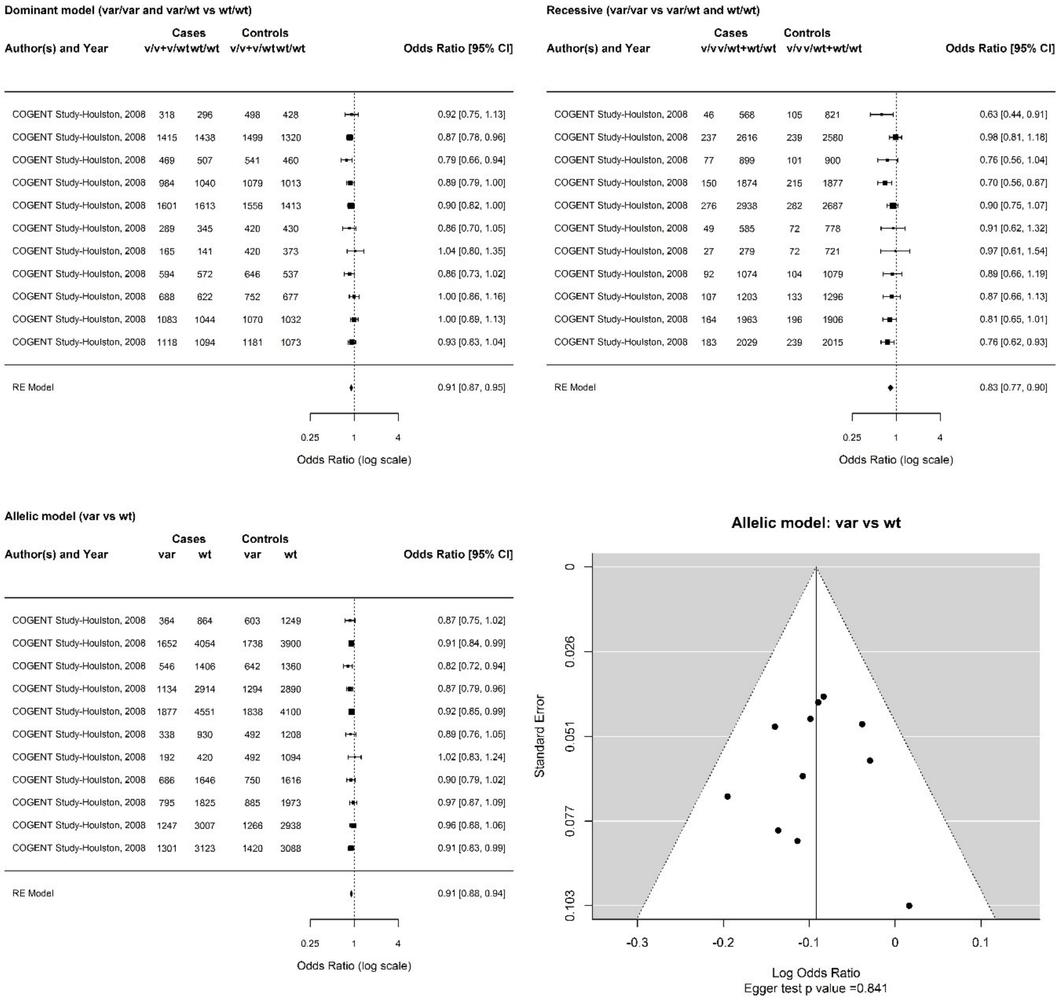

Supplementary Figure 11 Forest and Egger's plots for *CDH1* (rs1862748).

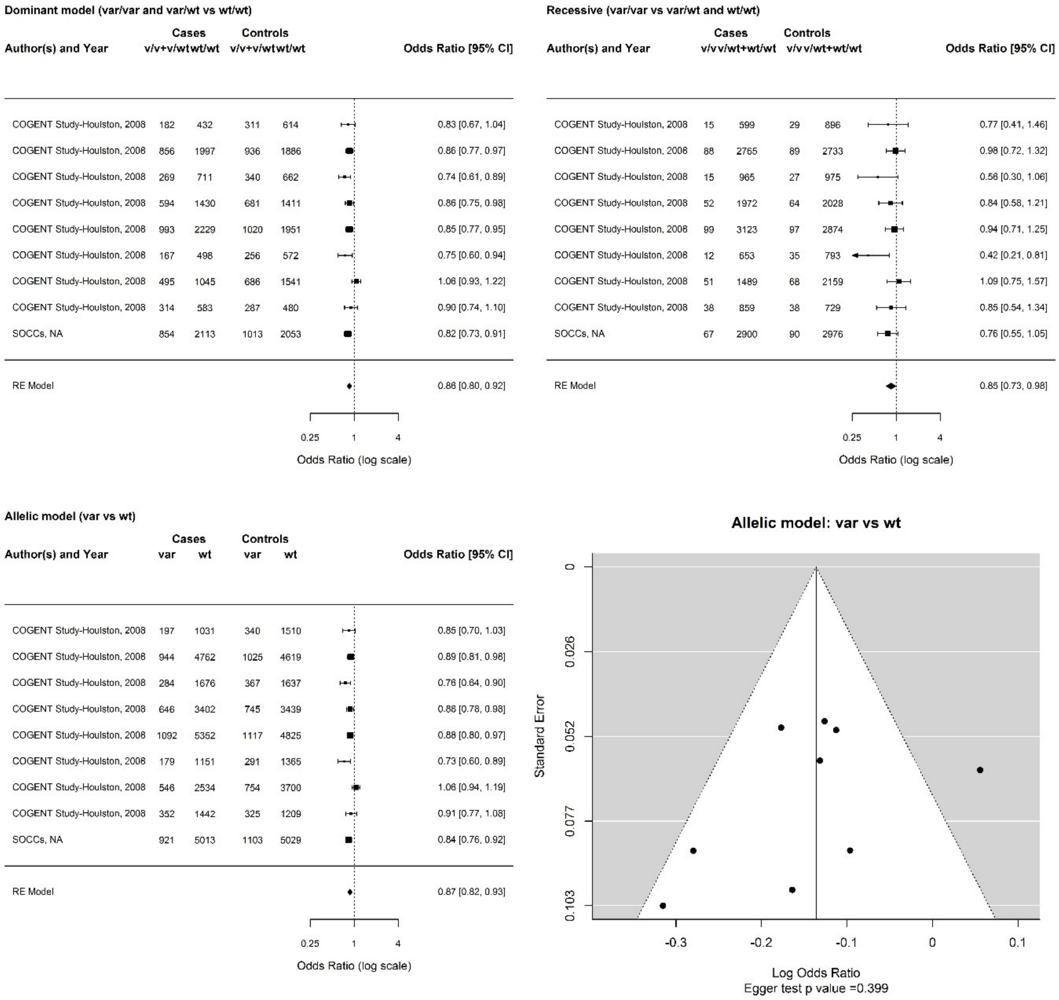

Supplementary Figure 12 Forest and Egger's plots for *RHPN2* (rs7259371).

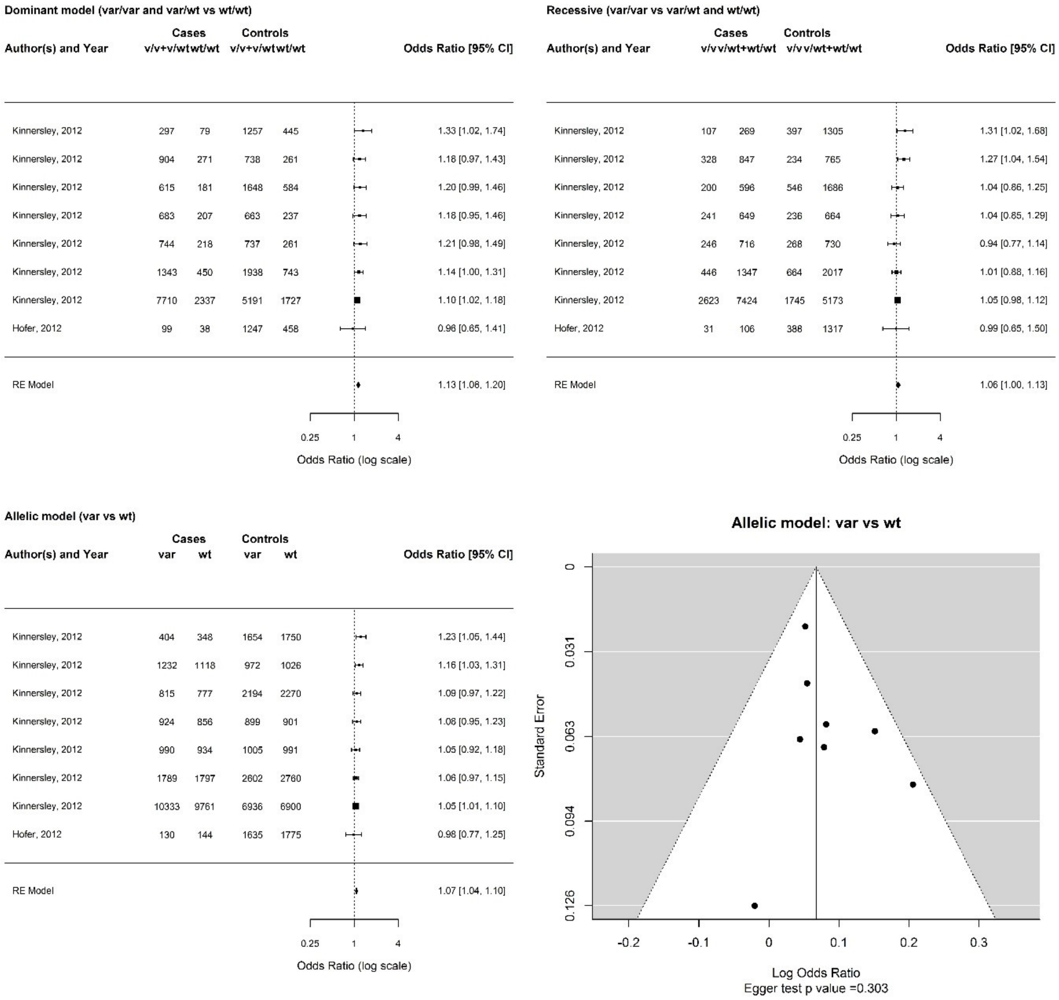

Supplementary Figure 13 Forest and Egger's plots for *TERT* (rs2736100).

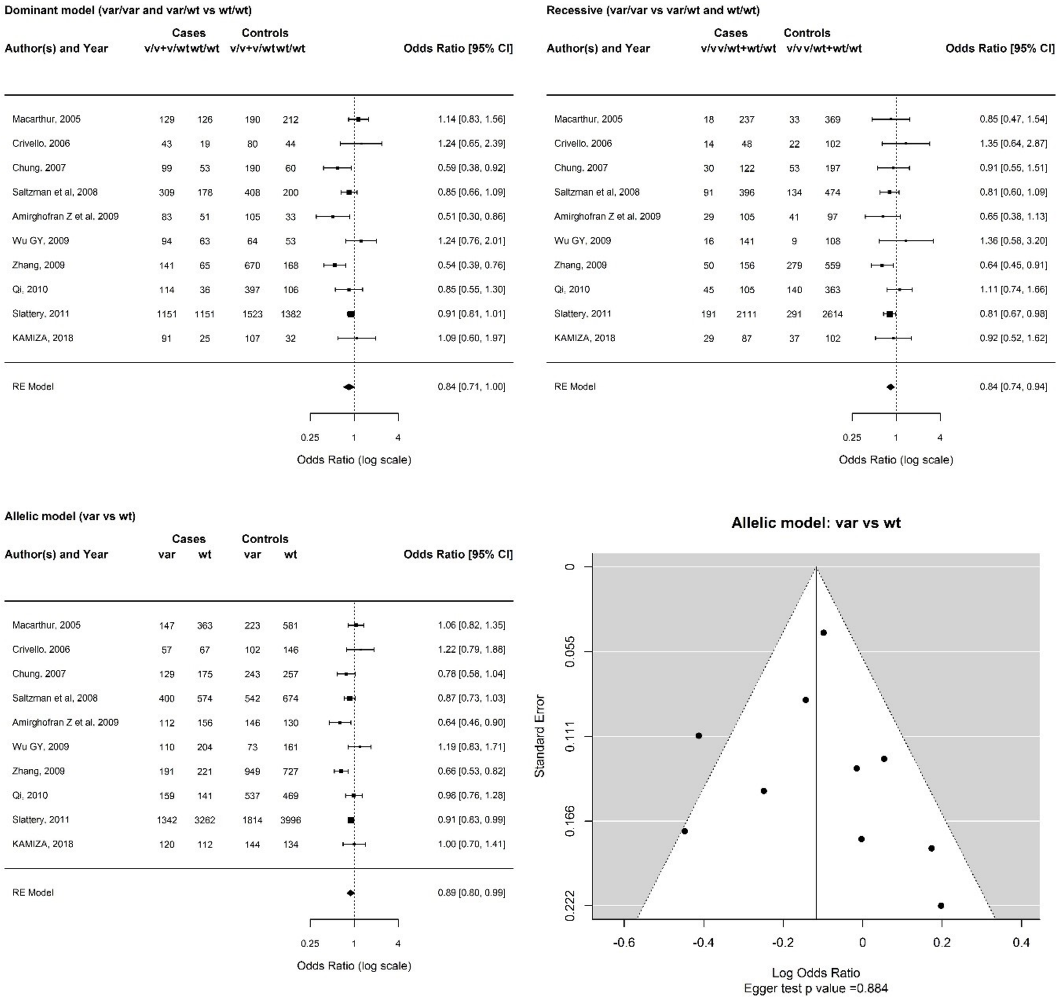

Supplementary Figure 14 Forest and Egger's plots for *TGFB1* (C509T, rs1800469).

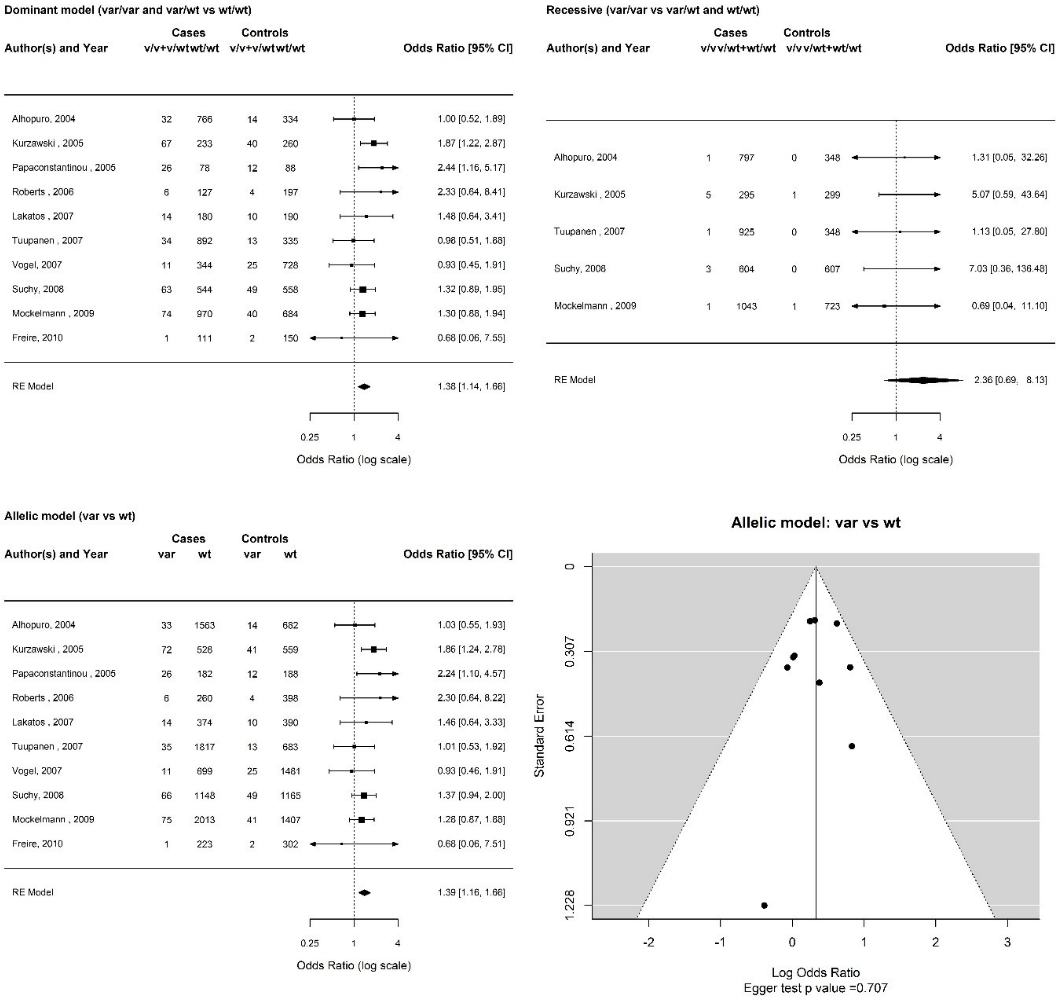

Supplementary Figure 15 Forest and Egger's plots for *NOD2* (3020insC, rs2066847).

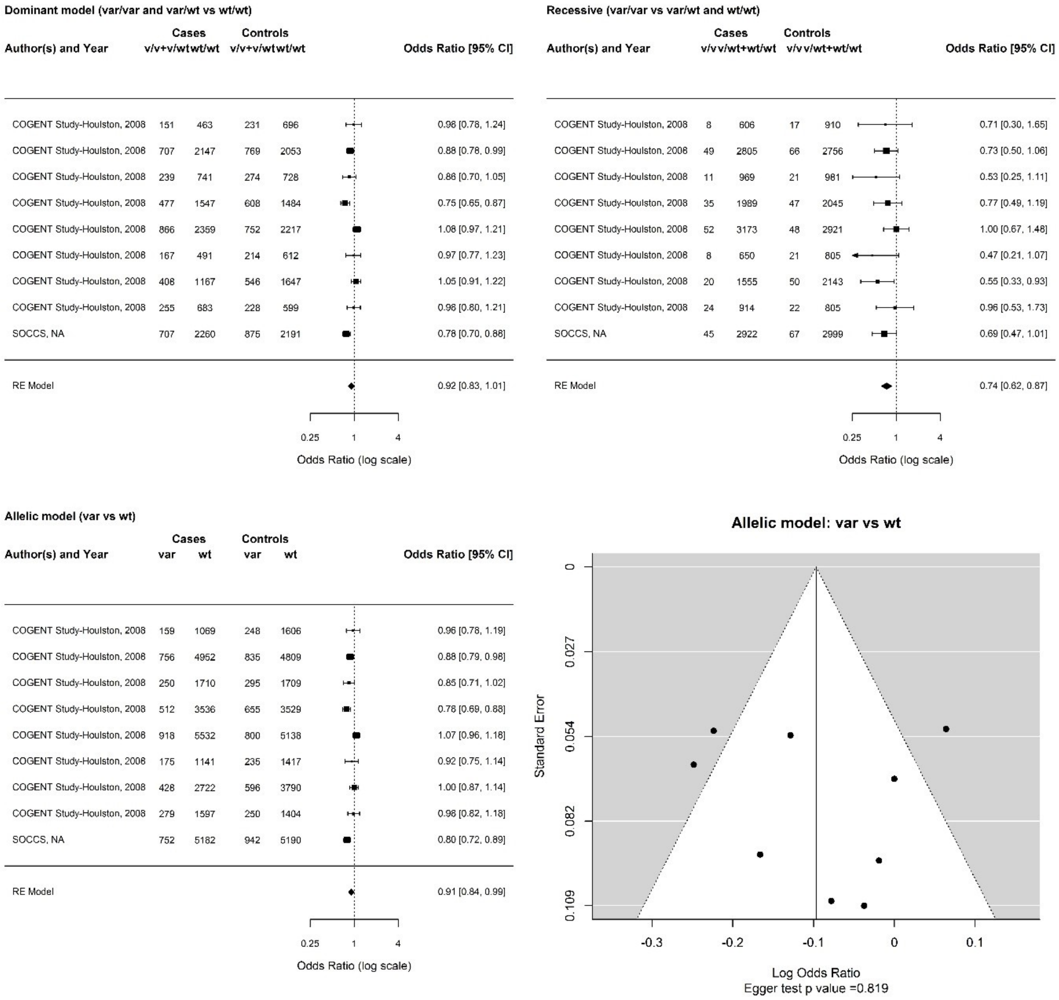

Supplementary Figure 16 Forest and Egger's plots for 1q32.1 (rs4951291).

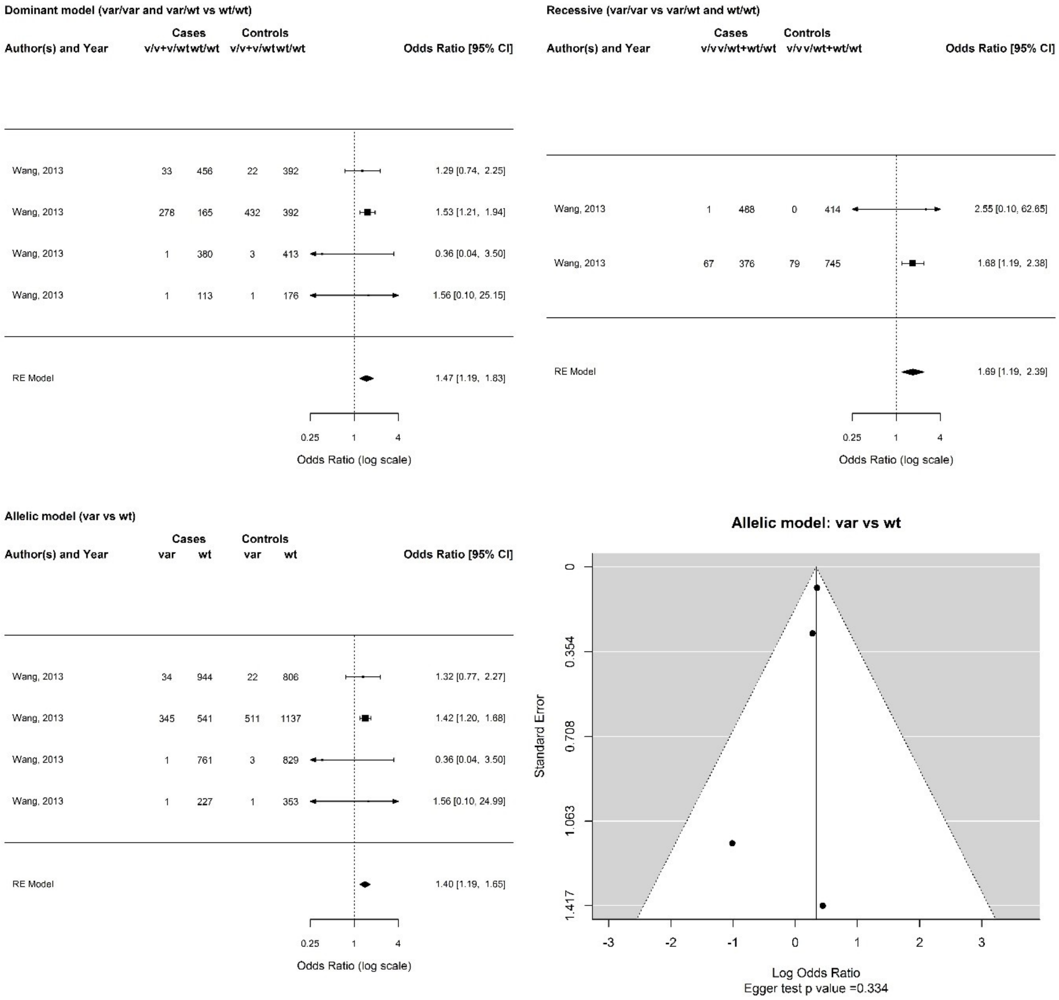

Supplementary Figure 17 Forest and Egger's plots for *PPAR-gamma* (rs9858822).

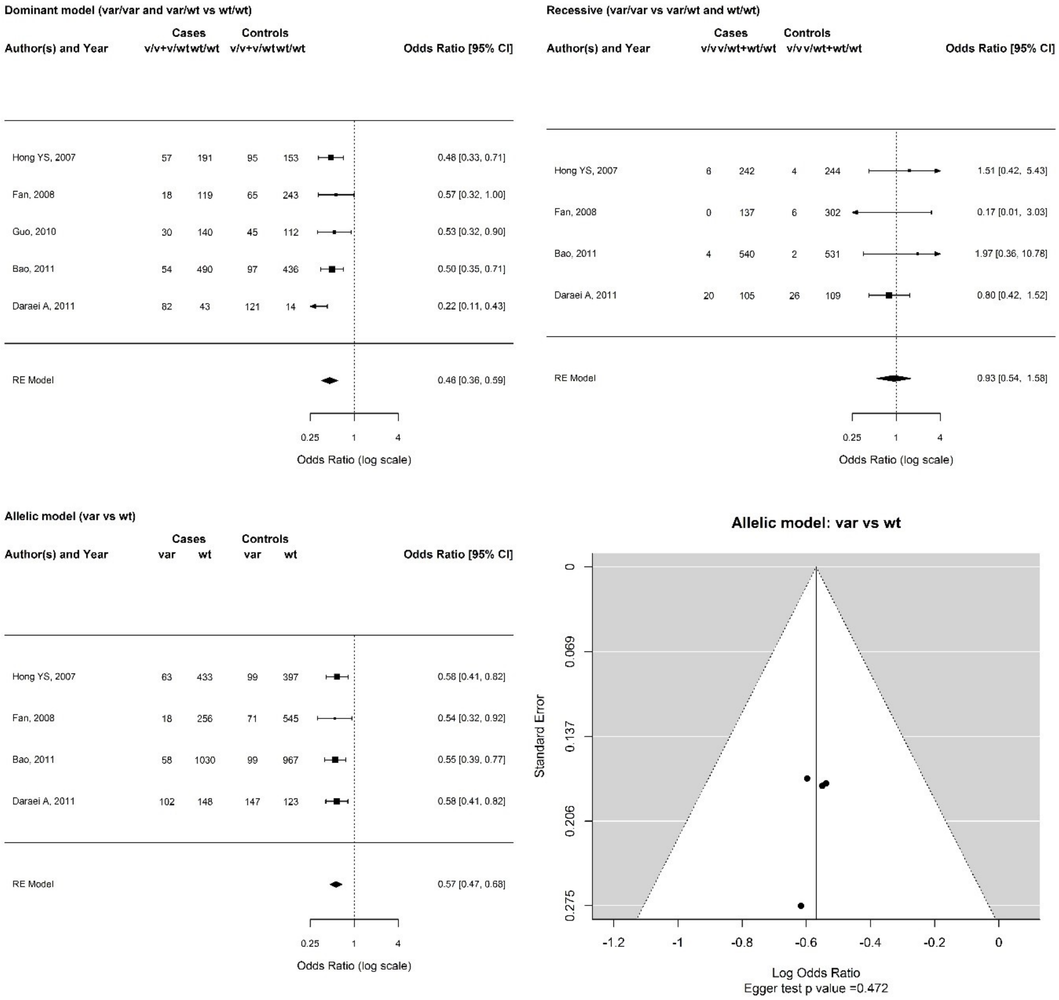

Supplementary Figure 18 Forest and Egger's plots for *DNMT3B* (G579T, rs1569686).

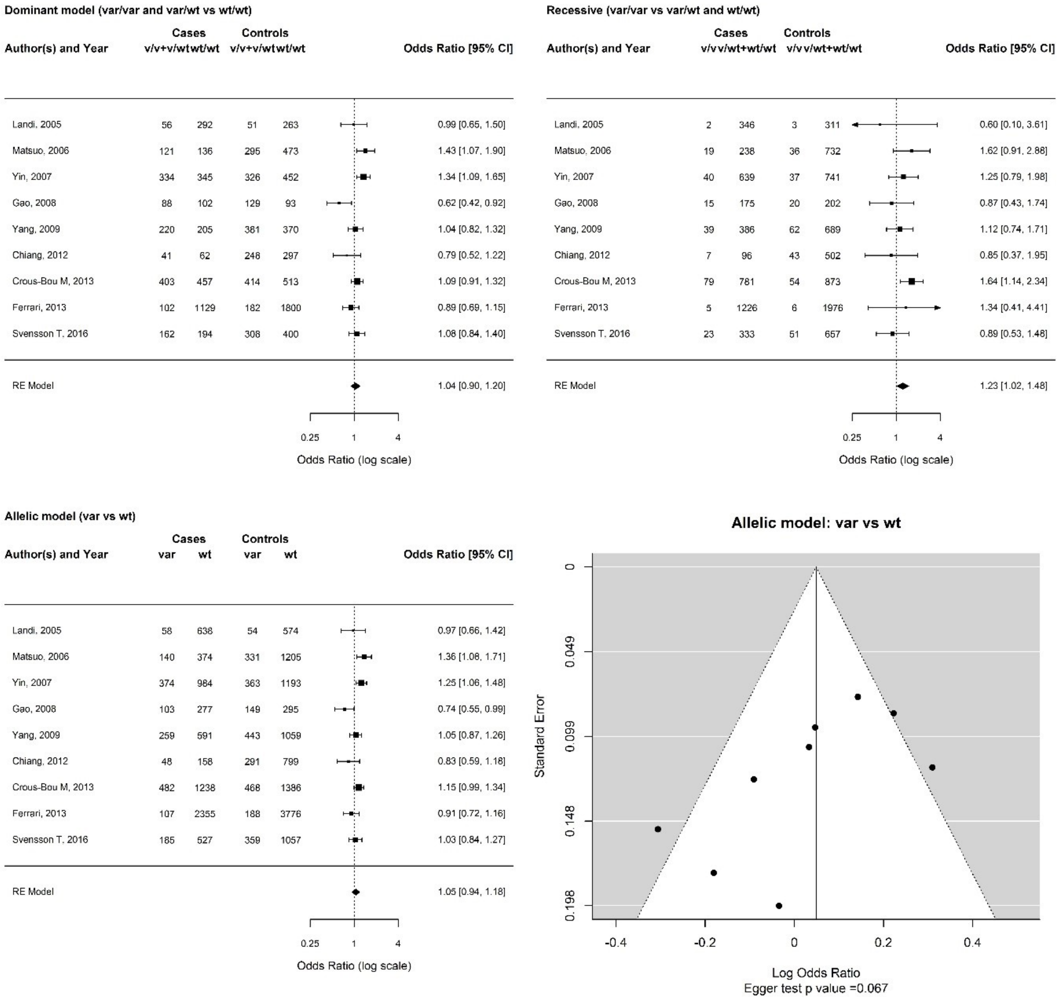

Supplementary Figure 19 Forest and Egger's plots for *ADH1B* (Arg47His, rs1229984).

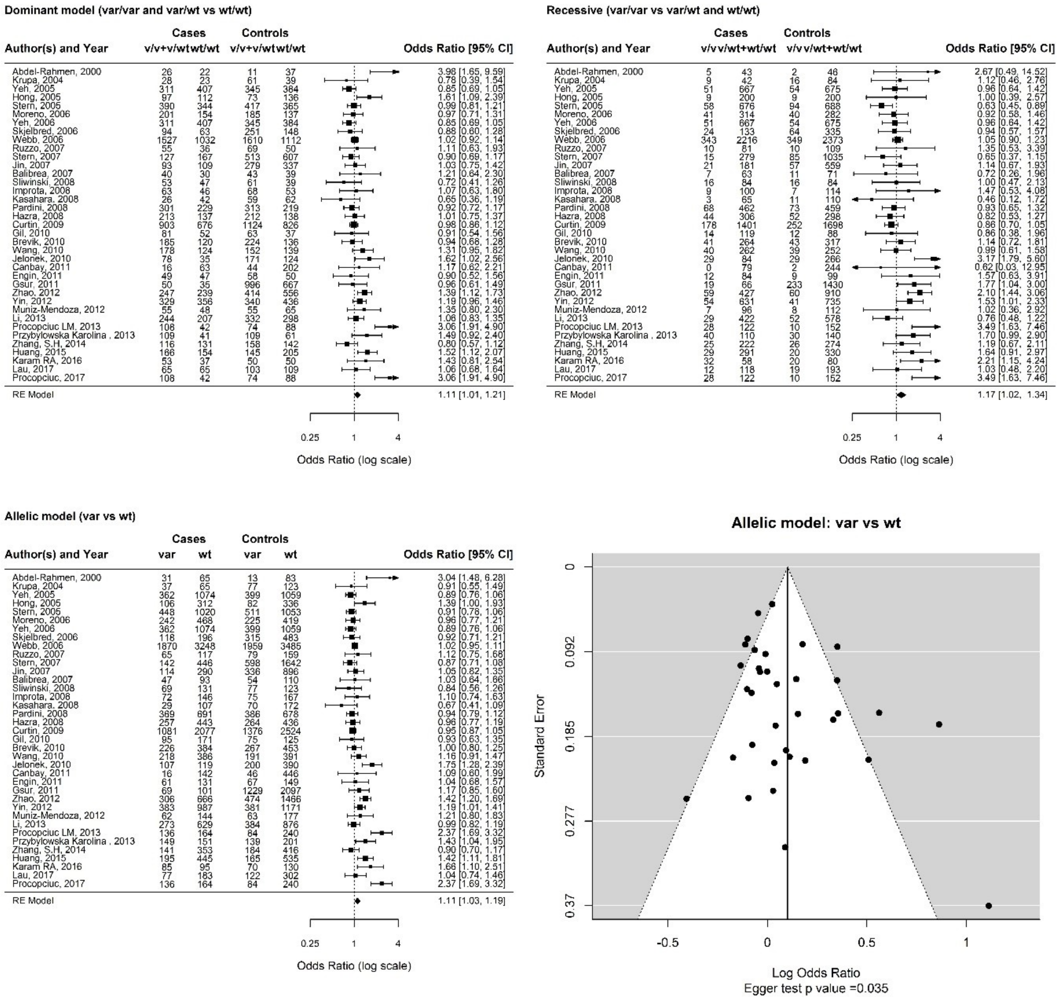

Supplementary Figure 20 Forest and Egger's plots for *XRCC1 (Arg399Gln, rs25487)*.

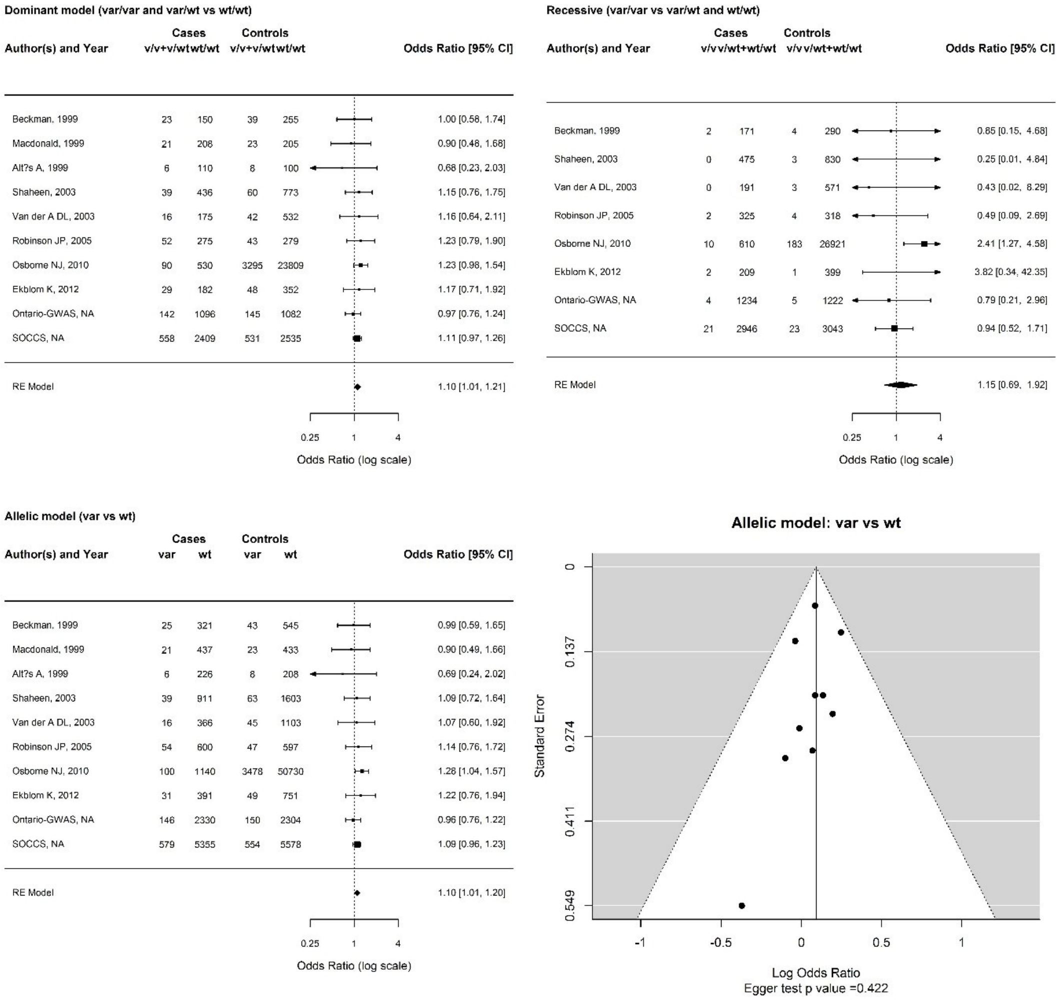

Supplementary Figure 21 Forest and Egger's plots for *HFE (C282T, rs1800562*).

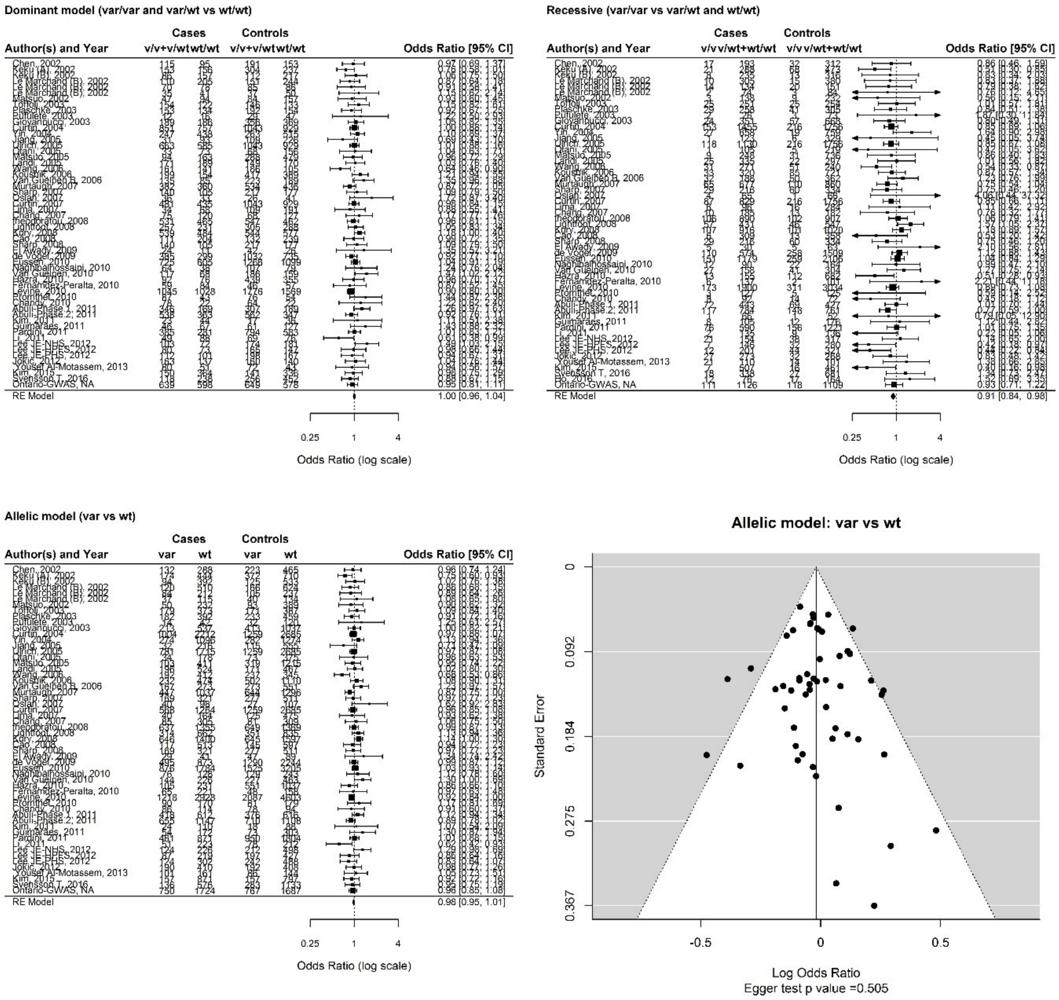

Supplementary Figure 22 Forest and Egger's plots for *MTHFR* (A1298C, rs1801131).

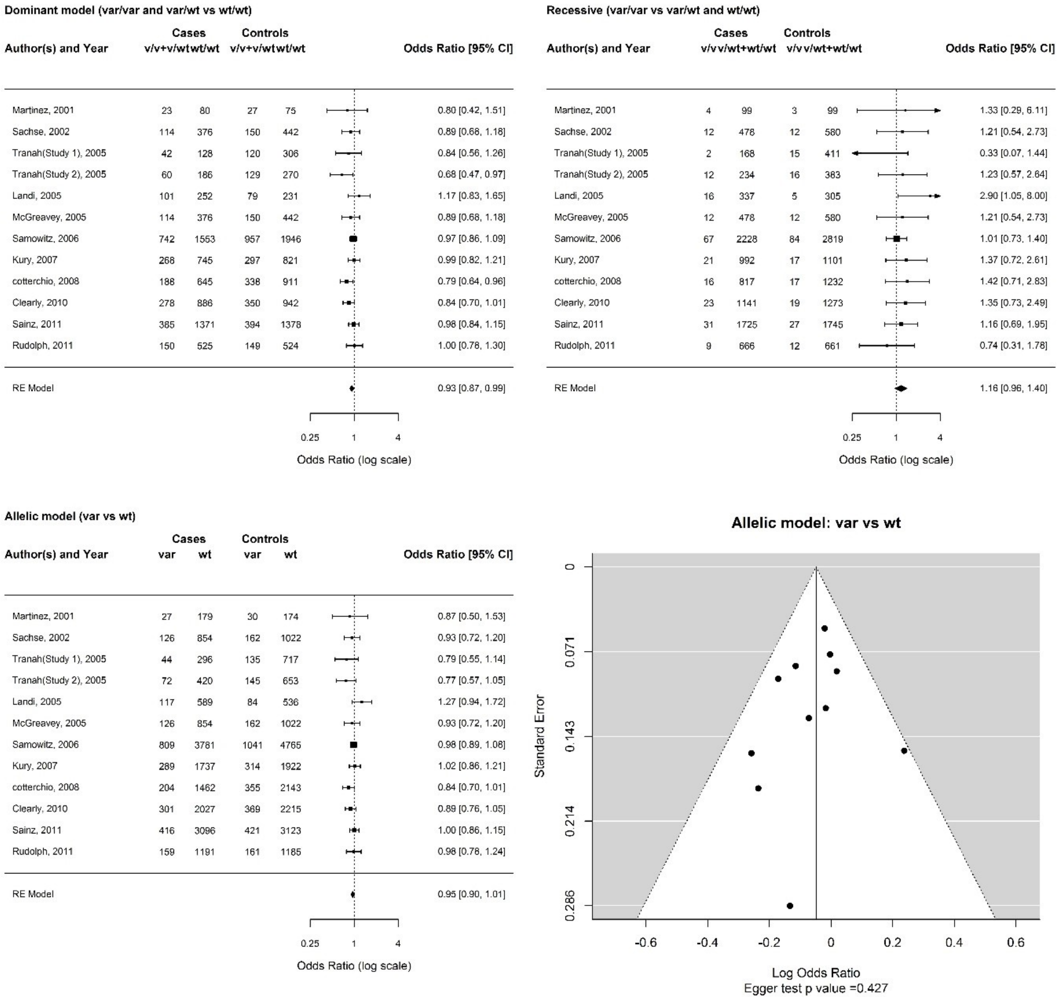

Supplementary Figure 23 Forest and Egger's plots for *CYP2C9 (430C>T, rs1799853*).

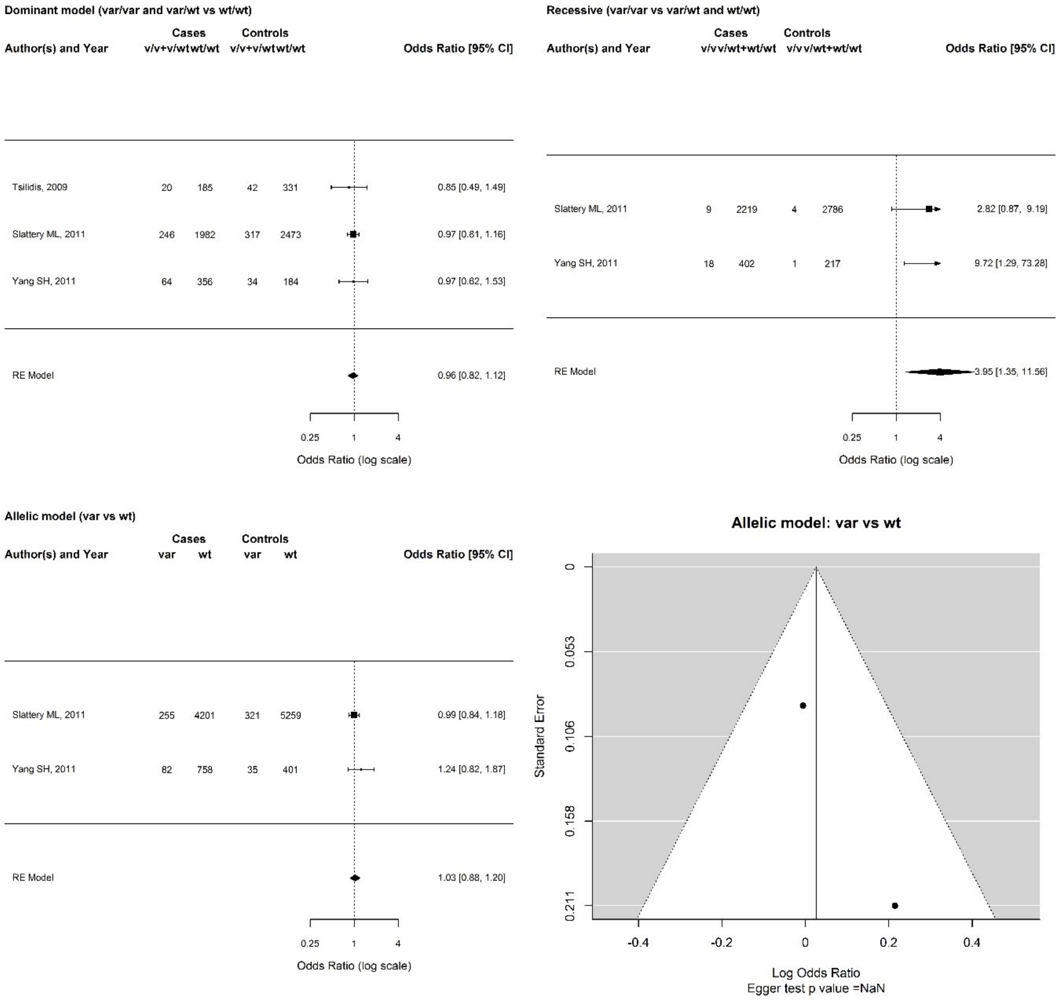

Supplementary Figure 24 Forest and Egger's plots for *CRP* (L184L, rs1800947).

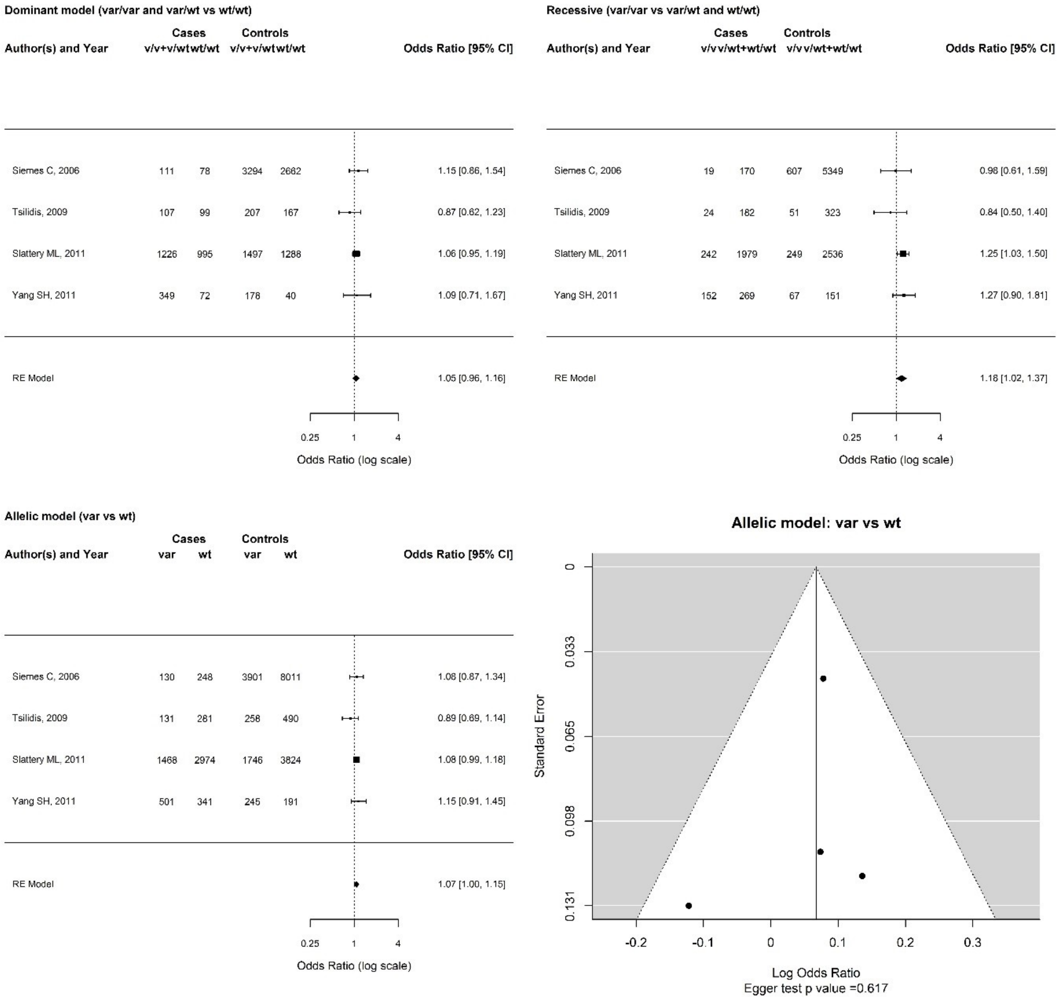

Supplementary Figure 25 Forest and Egger's plots for *CRP* (2042C>T, rs1205).

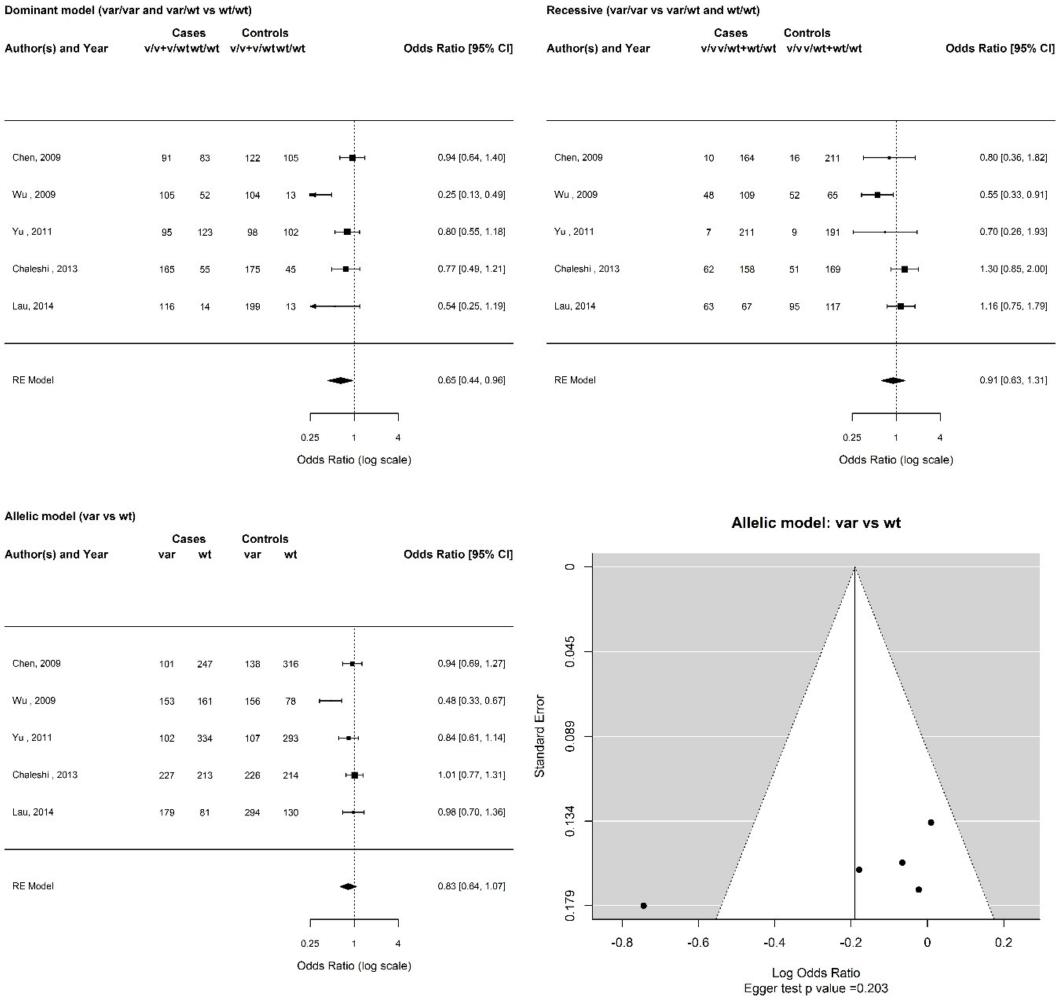

Dominant model: Author(s) and Year	Cases v/v+v/wt	Cases wt/wt	Controls v/v+v/wt	Controls wt/wt	Odds Ratio [95% CI]
Chen, 2009	91	83	122	105	0.94 [0.64, 1.40]
Wu , 2009	105	52	104	13	0.25 [0.13, 0.49]
Yu , 2011	95	123	98	102	0.80 [0.55, 1.18]
Chaleshi , 2013	165	55	175	45	0.77 [0.49, 1.21]
Lau, 2014	116	14	199	13	0.54 [0.25, 1.19]
RE Model					0.65 [0.44, 0.96]

Recessive: Author(s) and Year	Cases v/v	Cases v/wt+wt/wt	Controls v/v	Controls v/wt+wt/wt	Odds Ratio [95% CI]
Chen, 2009	10	164	16	211	0.80 [0.36, 1.82]
Wu , 2009	48	109	52	65	0.55 [0.33, 0.91]
Yu , 2011	7	211	9	191	0.70 [0.26, 1.93]
Chaleshi , 2013	62	158	51	169	1.30 [0.85, 2.00]
Lau, 2014	63	67	95	117	1.16 [0.75, 1.79]
RE Model					0.91 [0.63, 1.31]

Allelic model: Author(s) and Year	Cases var	Cases wt	Controls var	Controls wt	Odds Ratio [95% CI]
Chen, 2009	101	247	138	316	0.94 [0.69, 1.27]
Wu , 2009	153	161	156	78	0.48 [0.33, 0.67]
Yu , 2011	102	334	107	293	0.84 [0.61, 1.14]
Chaleshi , 2013	227	213	226	214	1.01 [0.77, 1.31]
Lau, 2014	179	81	294	130	0.98 [0.70, 1.36]
RE Model					0.83 [0.64, 1.07]

Supplementary Figure 26 Forest and Egger's plots for *EGF* (A61G, rs4444903).

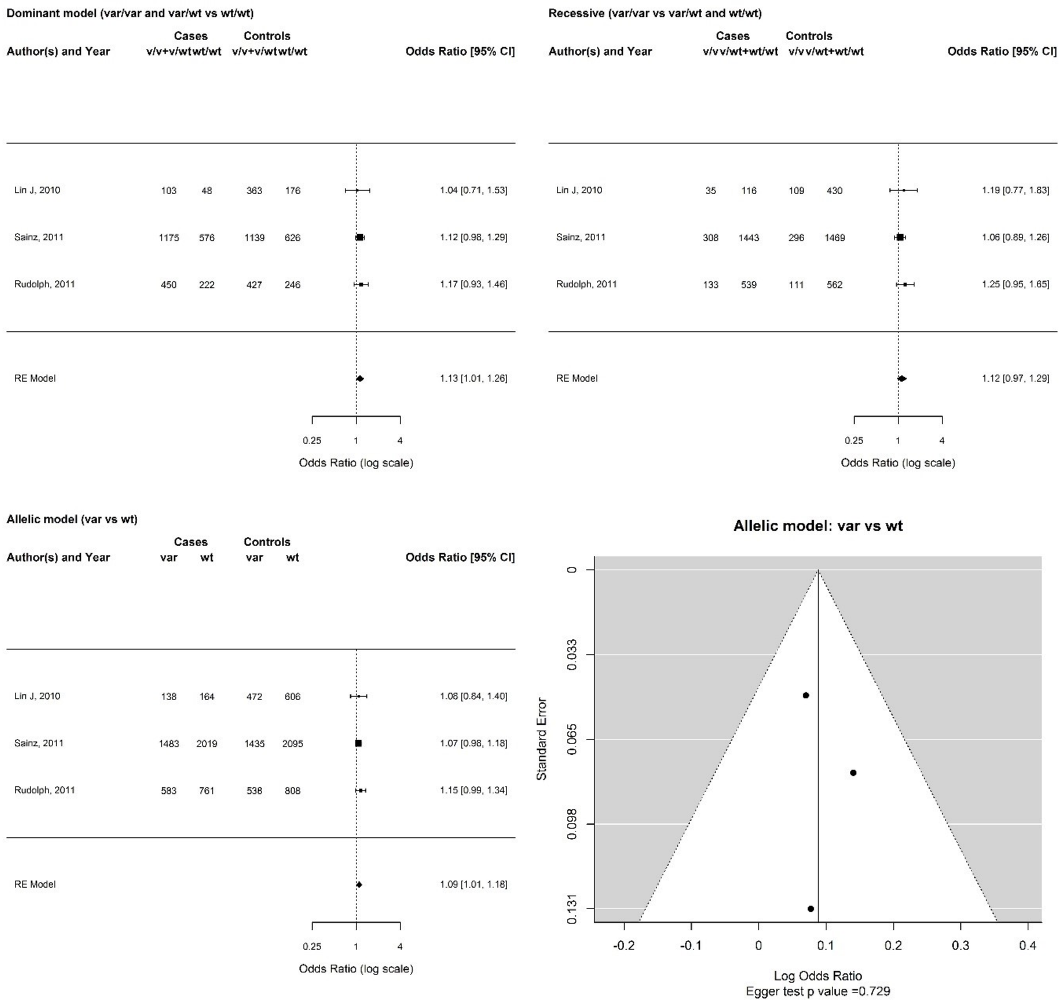

Supplementary Figure 27 Forest and Egger's plots for *ESR2* (A61G, rs928554).

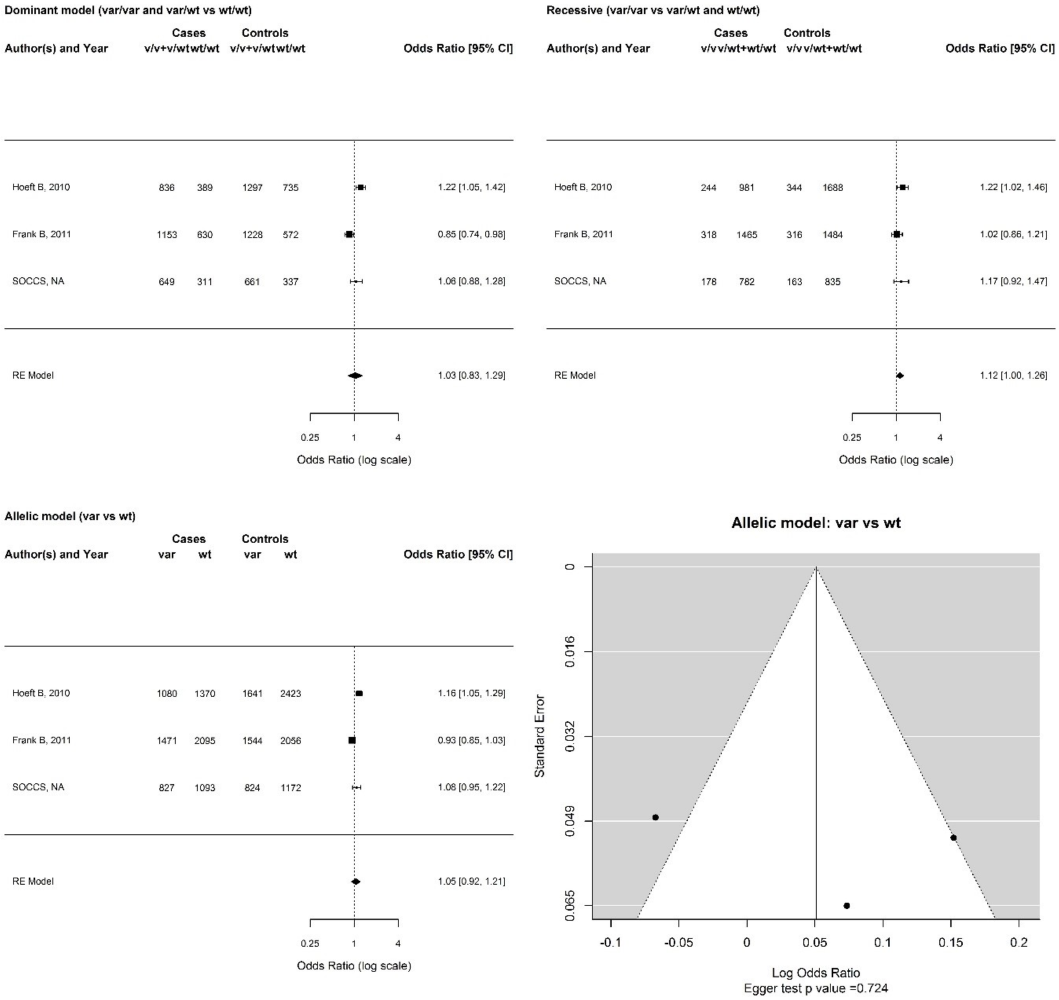

Supplementary Figure 28 Forest and Egger's plots for *HPGD* (rs8752).

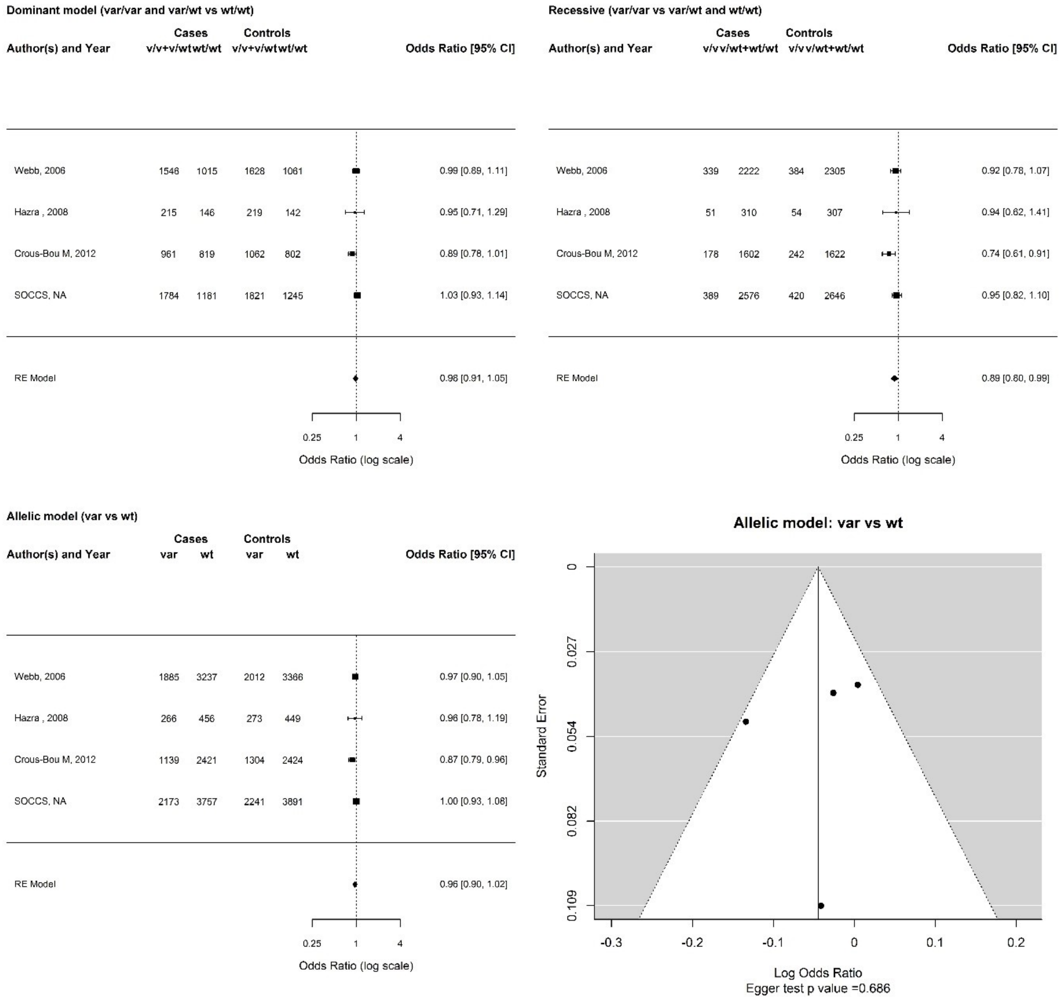

Supplementary Figure 29 Forest and Egger's plots for *LIPC* (N215S, rs6083).

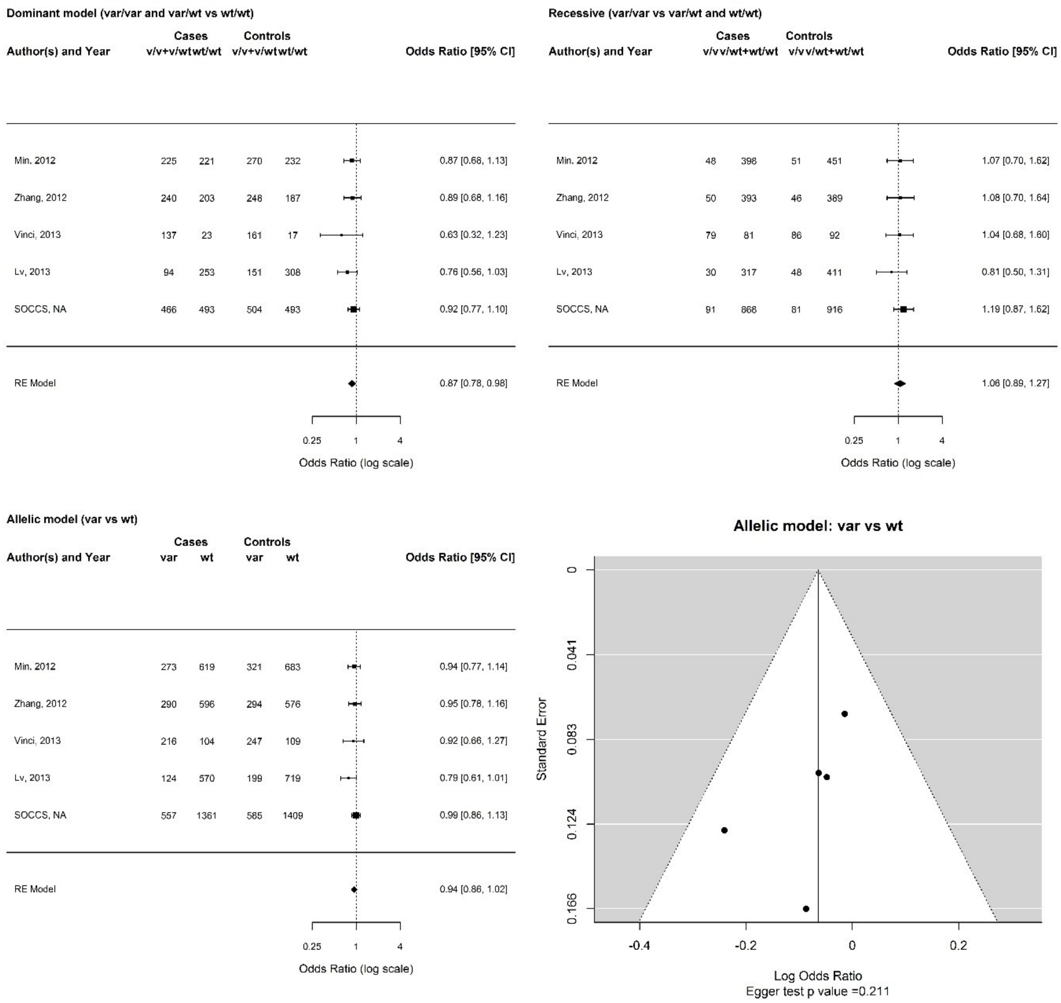

Supplementary Figure 30 Forest and Egger's plots for miR-149 (rs2292832).

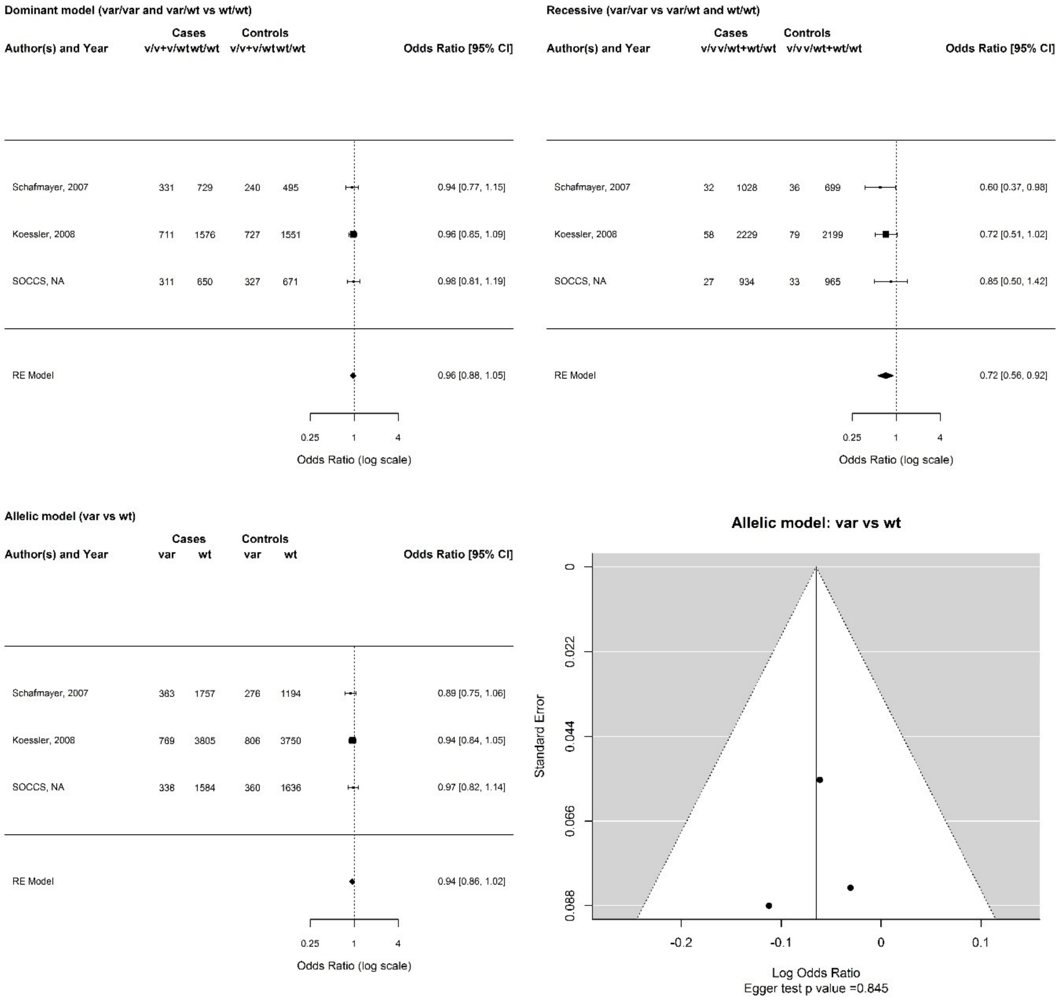

Supplementary Figure 31 Forest and Egger's plots for *MSH2* (rs4608577).

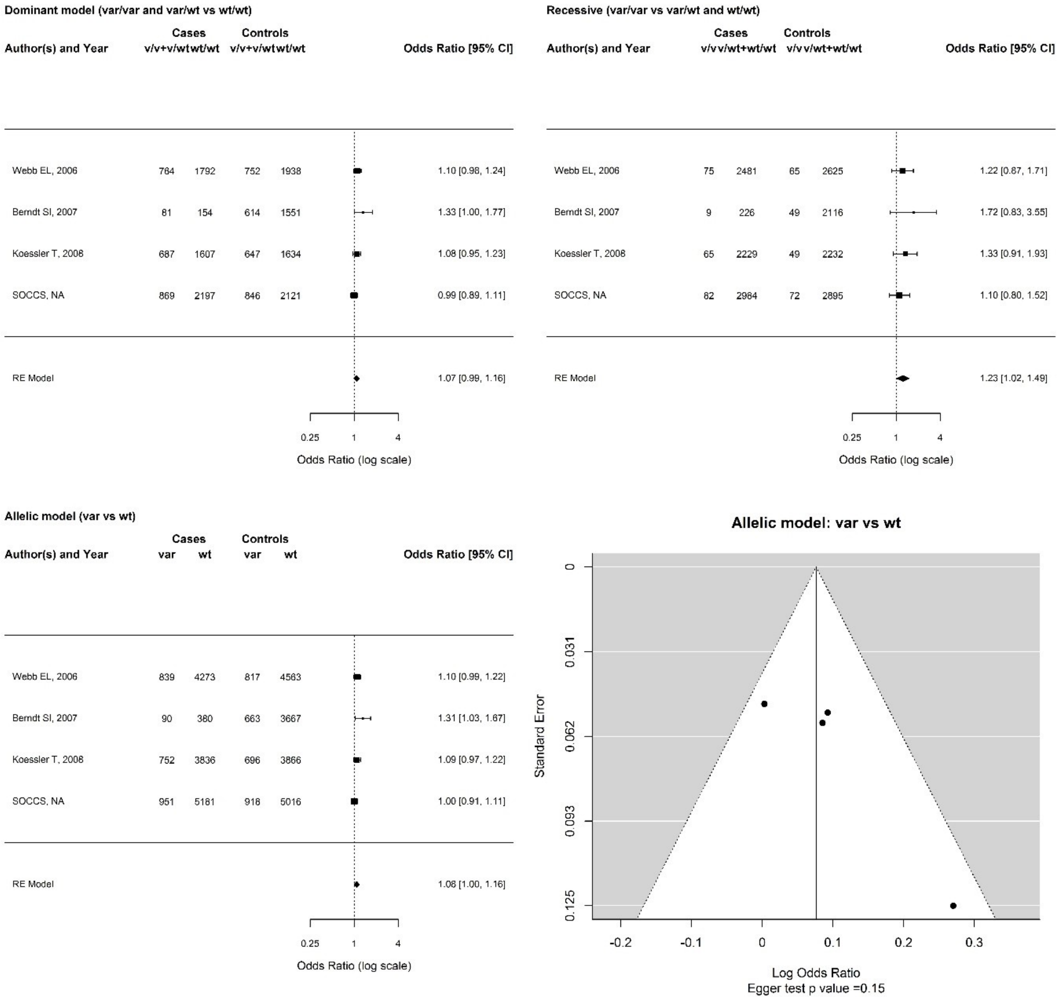

Supplementary Figure 32 Forest and Egger's plots for *MSH3 (Q949R, rs184967*).

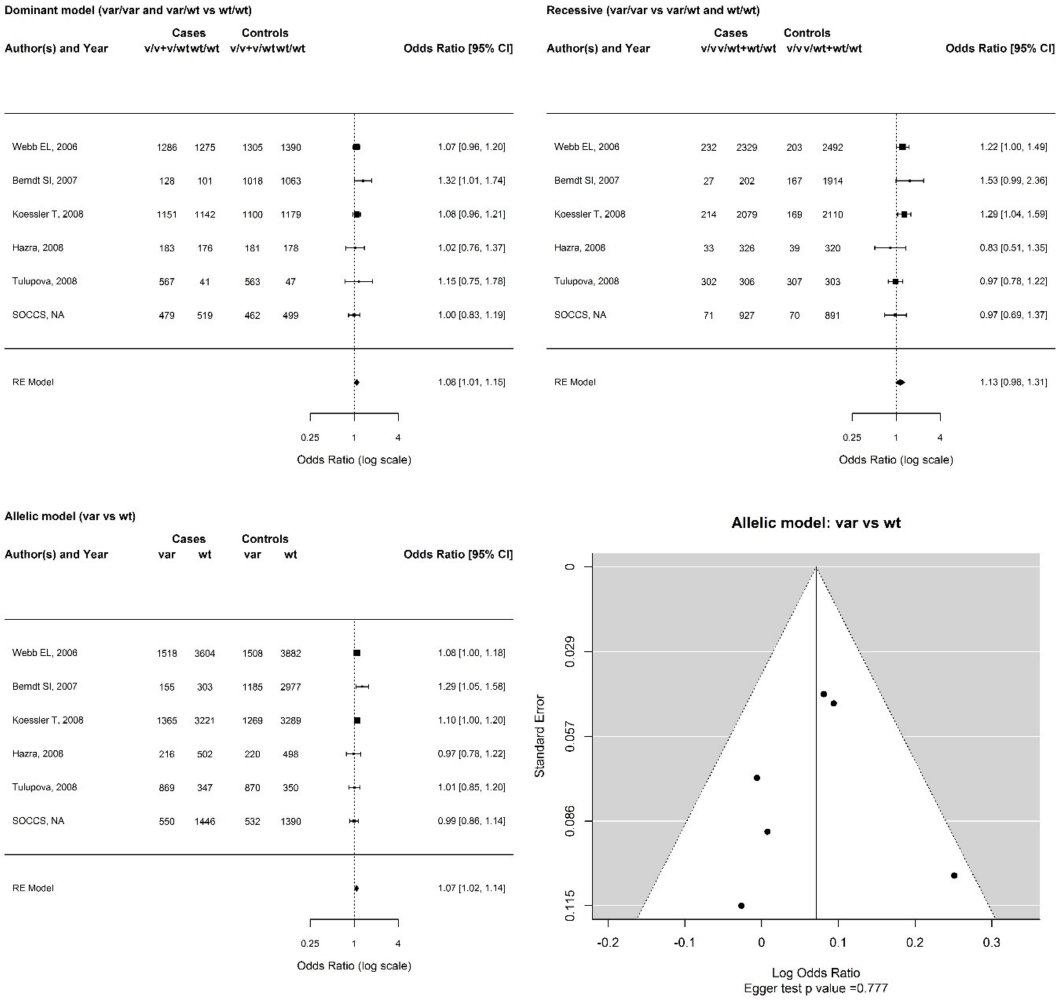

Supplementary Figure 33 Forest and Egger's plots for *MSH3 (A1036T, rs26779)*.

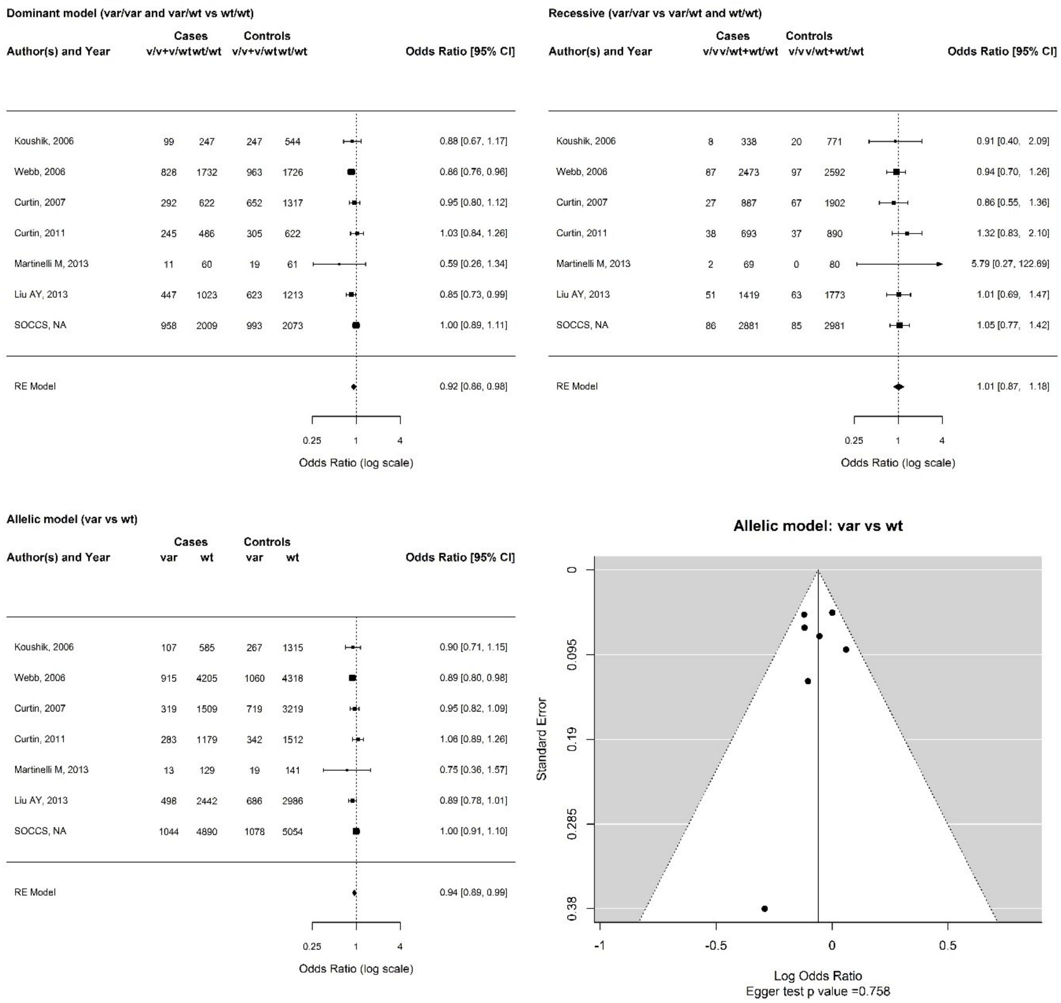

Supplementary Figure 34 Forest and Egger's plots for *MTHFD1 (Arg134Lys, rs1950902*).

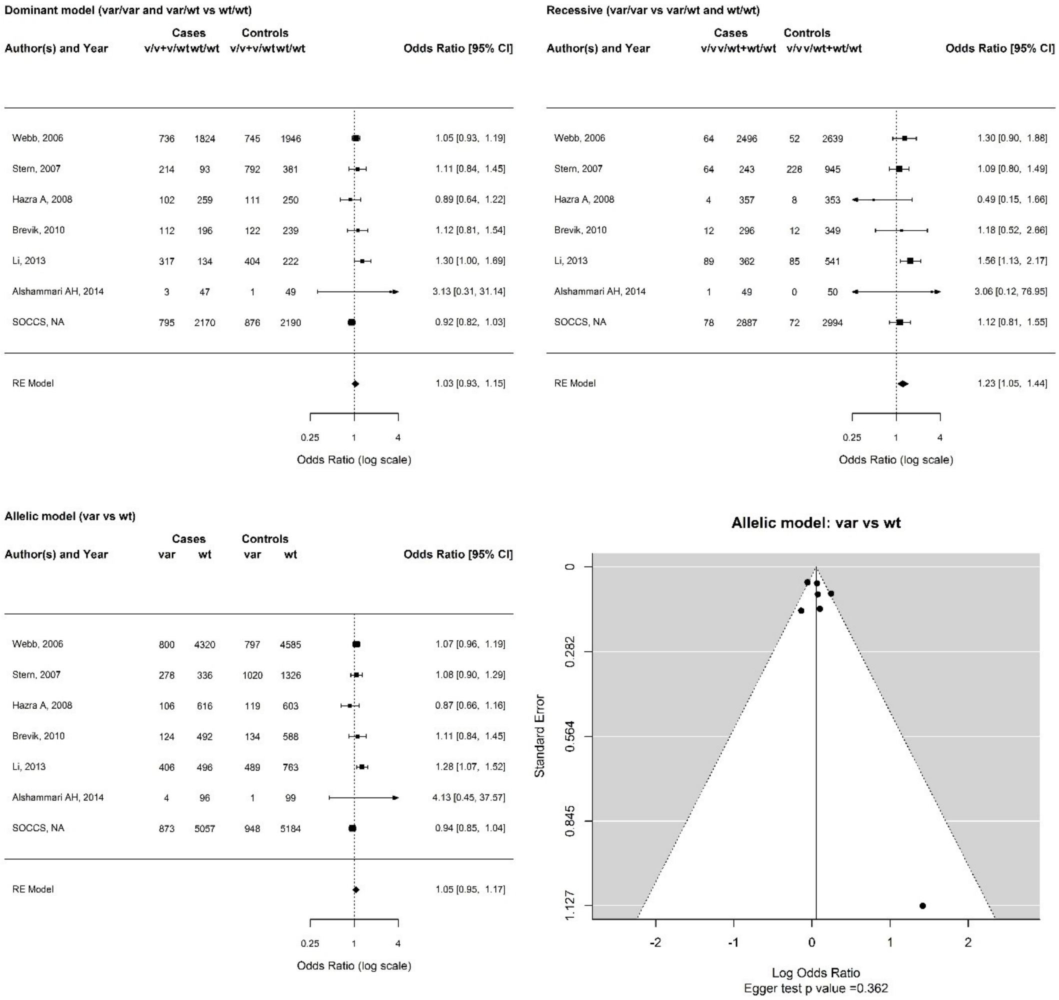

Supplementary Figure 35 Forest and Egger’s plots for *PARP1* (Val762Ala, rs1136410).

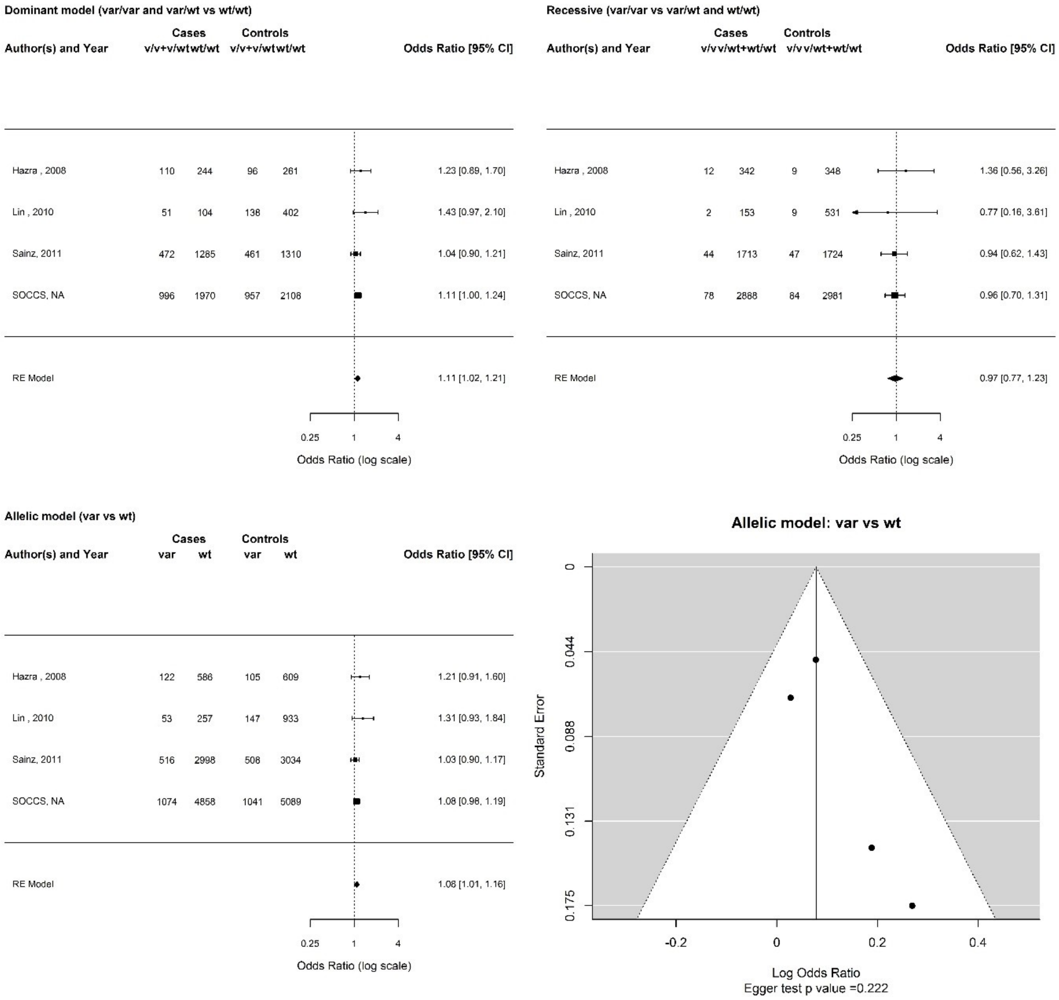

Supplementary Figure 36 Forest and Egger's plots for *PGR* (V660L, rs1042838).

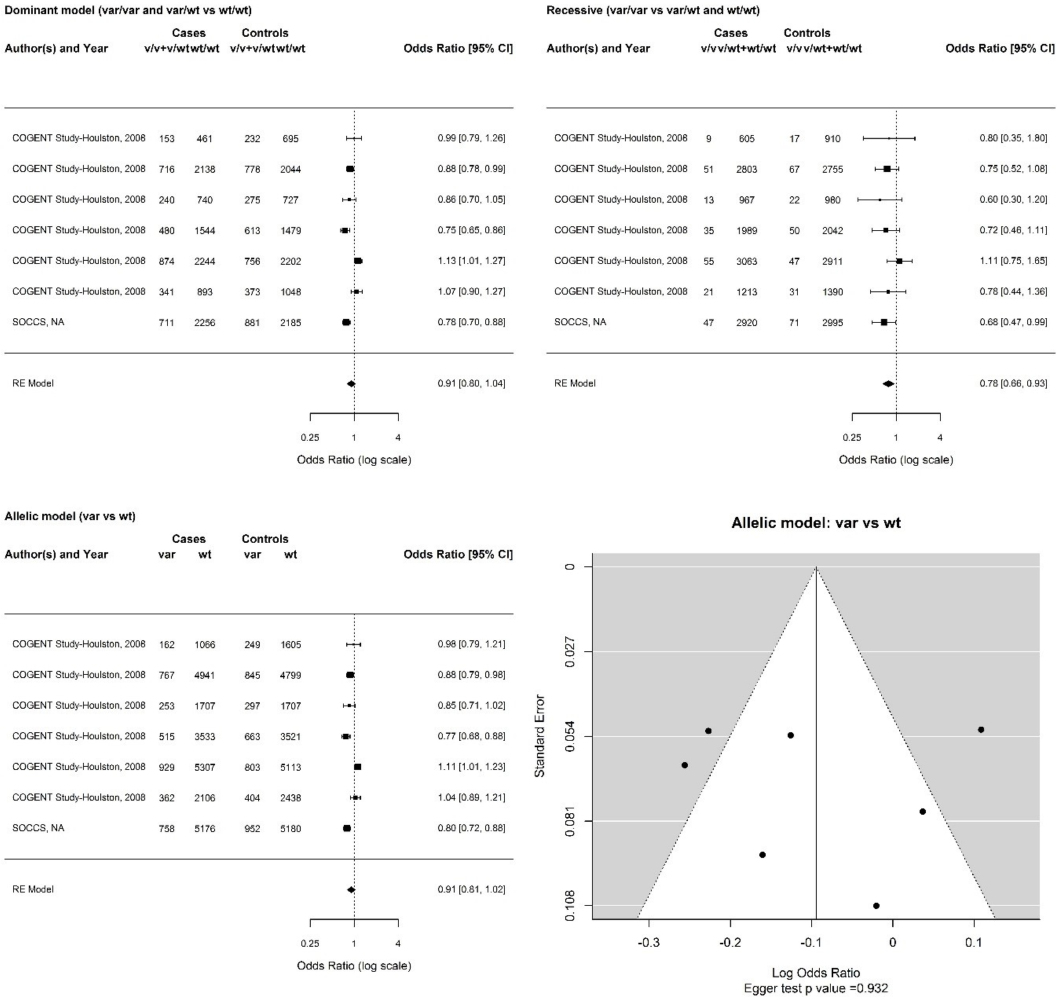

Supplementary Figure 37 Forest and Egger's plots for polymorphism (rs4951039).

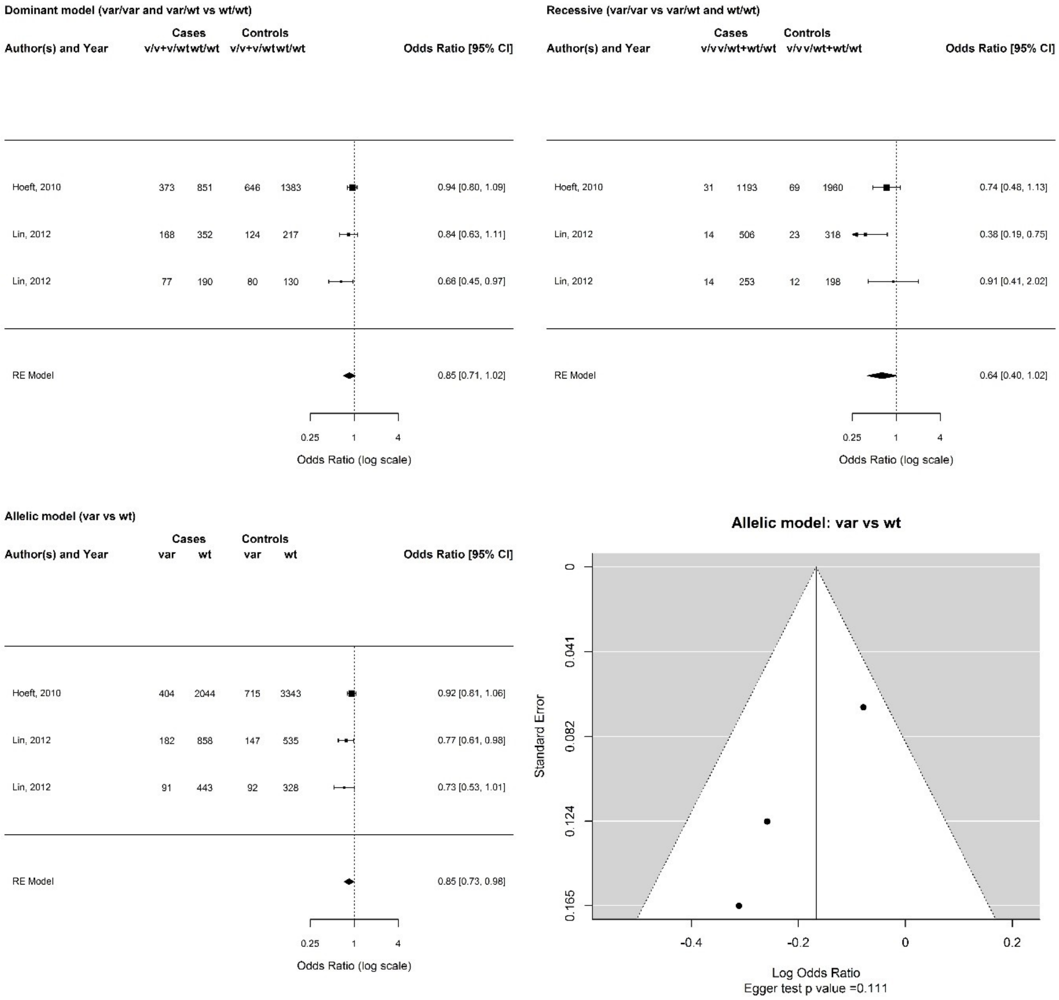

Supplementary Figure 38 Forest and Egger's plots for *SCD* (rs7849).

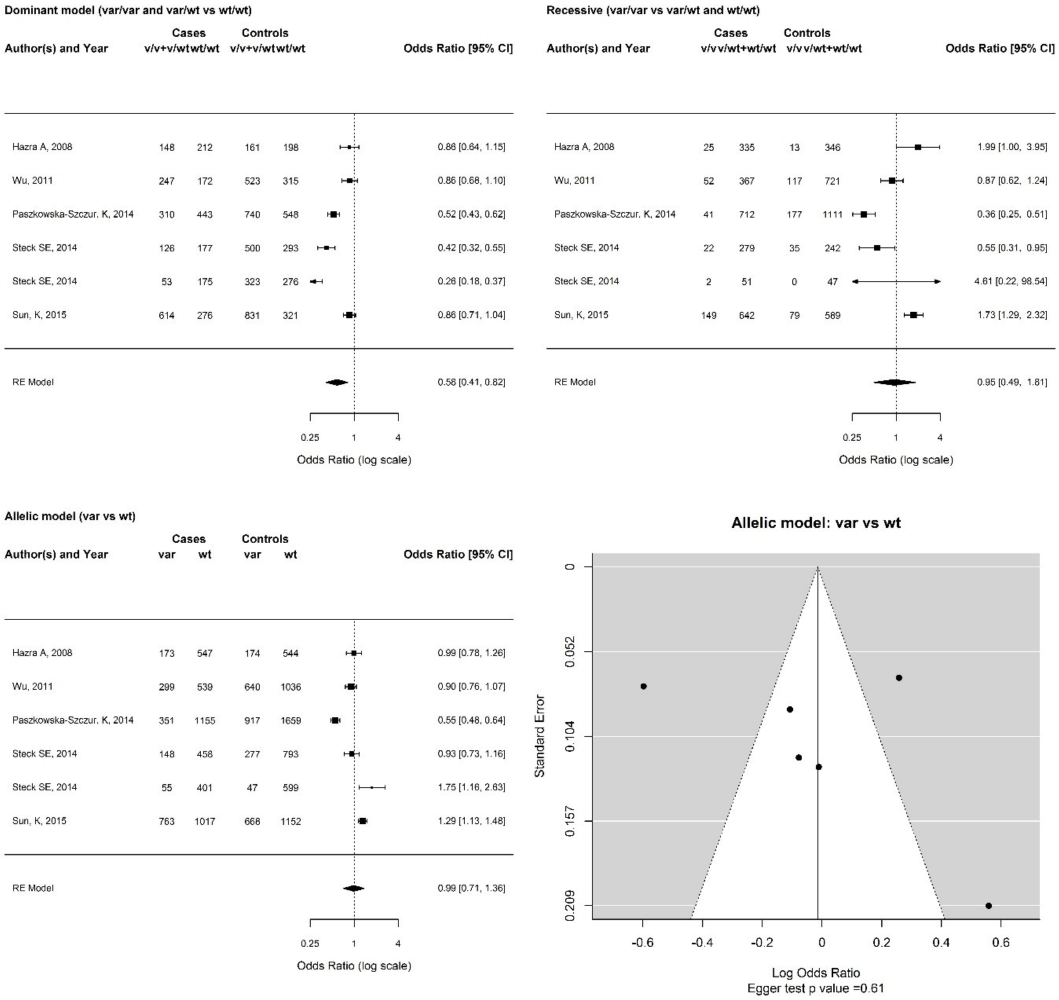

Supplementary Figure 39 Forest and Egger's plots for *XPC* (Ala499Val, rs2228000).

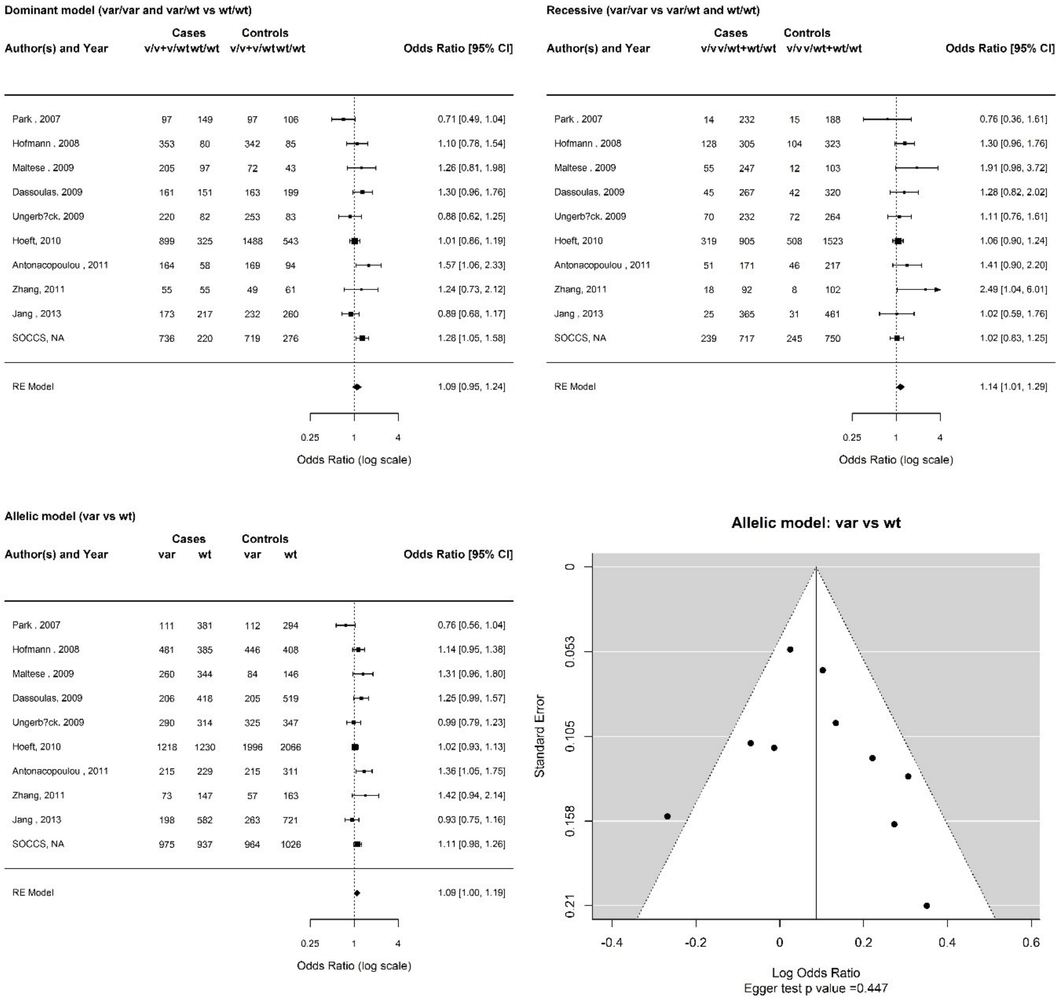

Supplementary Figure 40 Forest and Egger's plots for *VEGF* (2578C>A, rs699947).

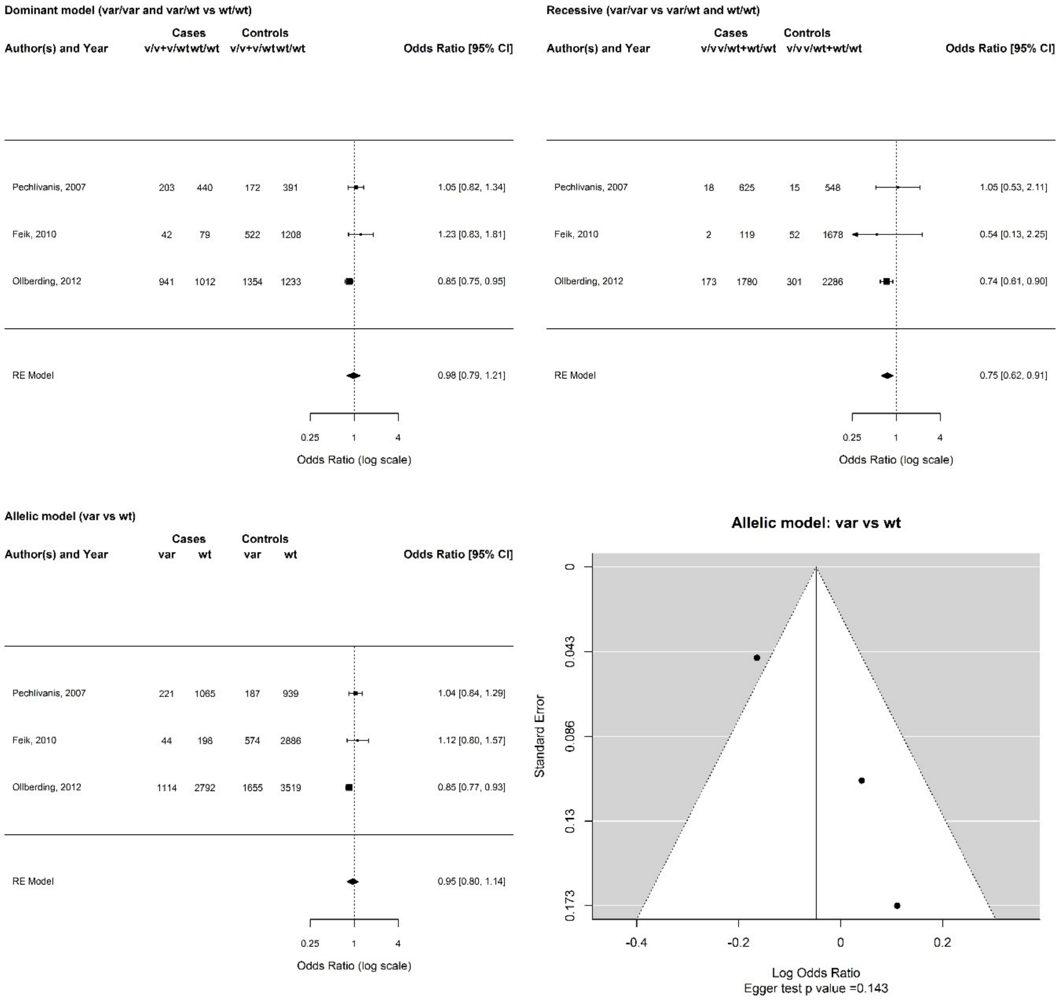

Supplementary Figure 41 Forest and Egger's plots for *IGF1(C1410T, rs35767)*.

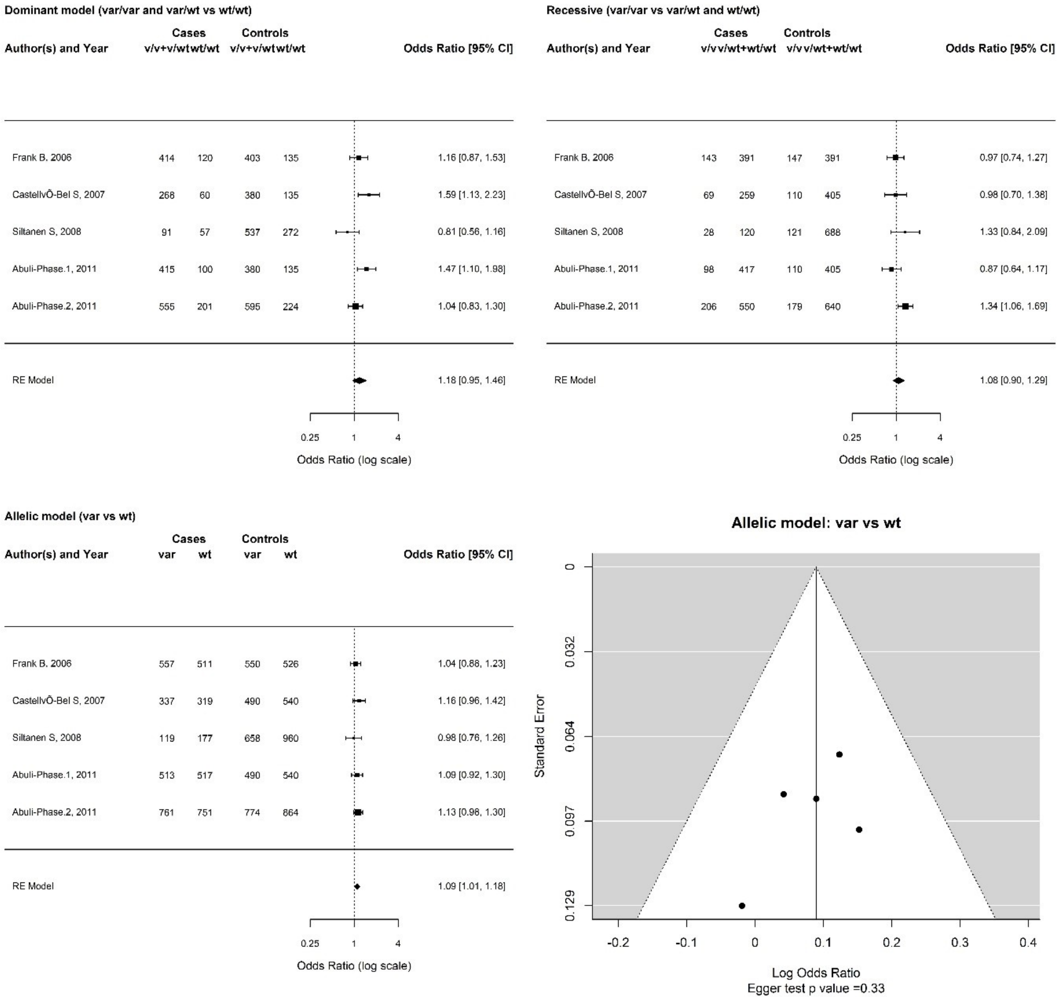

Supplementary Figure 42 Forest and Egger's plots for *ARLTS1 (T442C, rs3803185*).

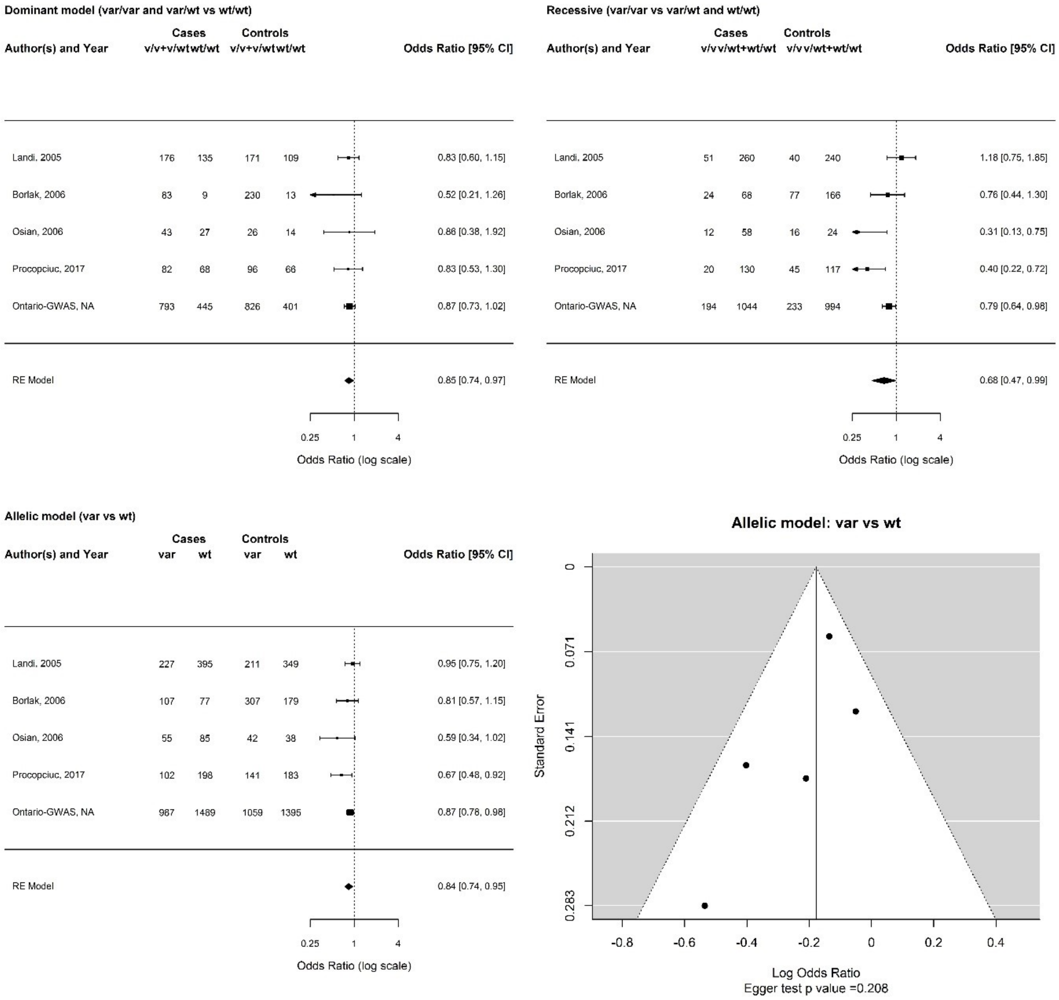

Supplementary Figure 43 Forest and Egger's plots for *NAT2* (481C>T, rs1799929).

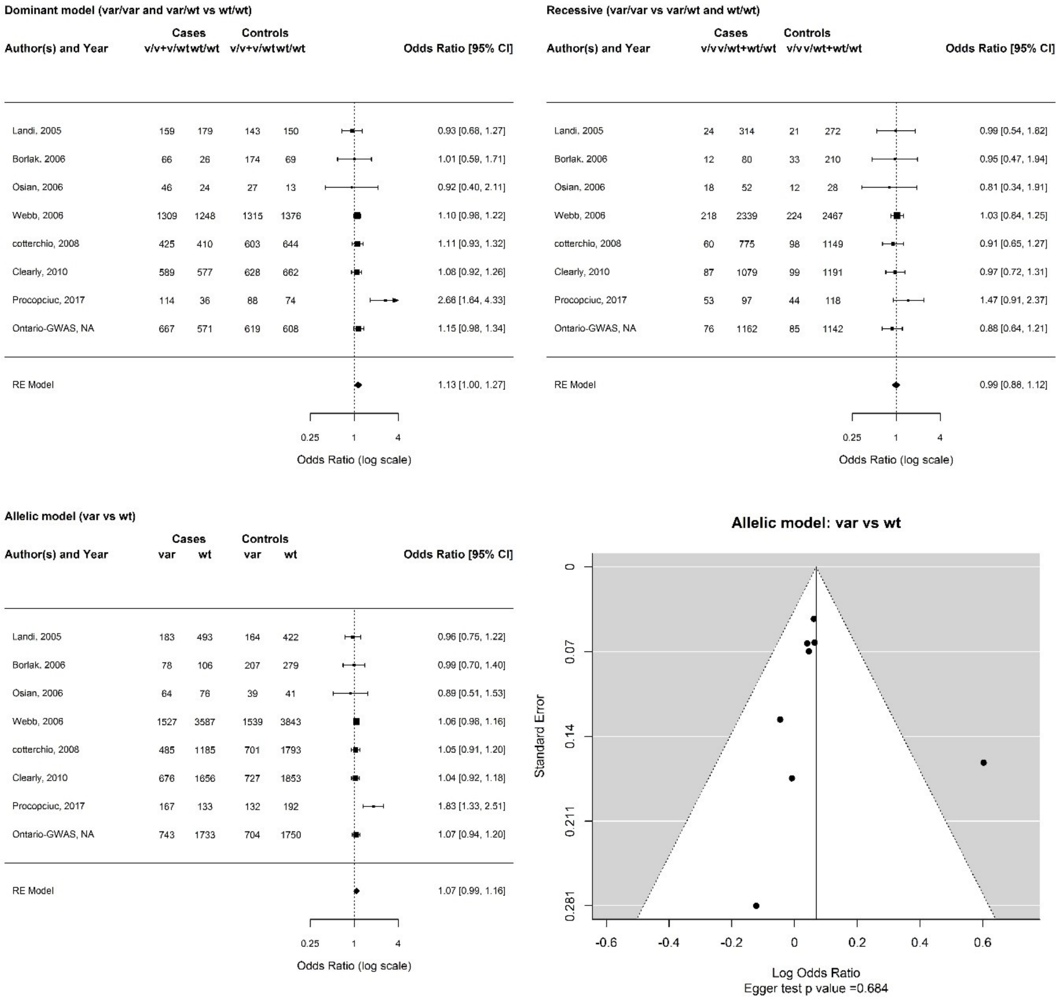

Supplementary Figure 44 Forest and Egger's plots for *NAT2* (590G>A, rs1799930).

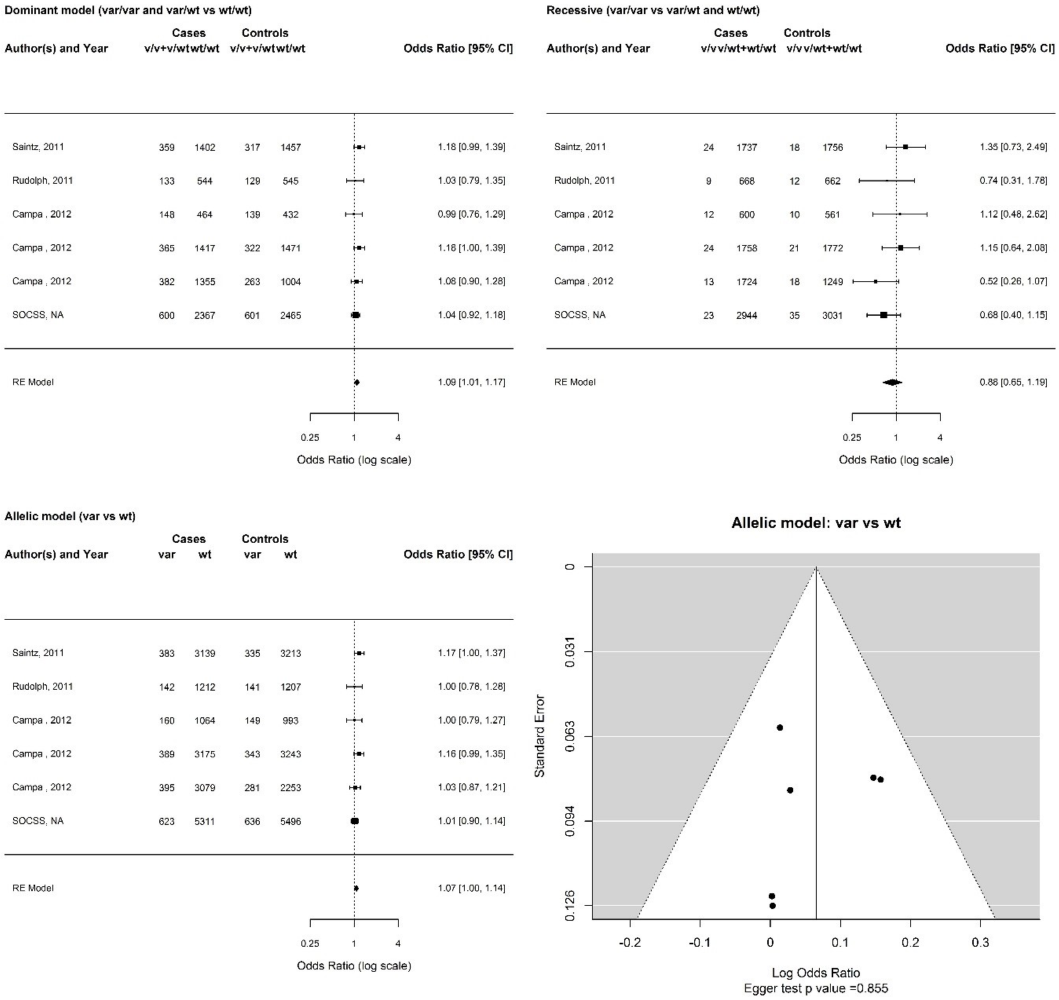

Supplementary Figure 45 Forest and Egger's plots for *ABCB1 (MDR1)* (61A>G, rs9282564).

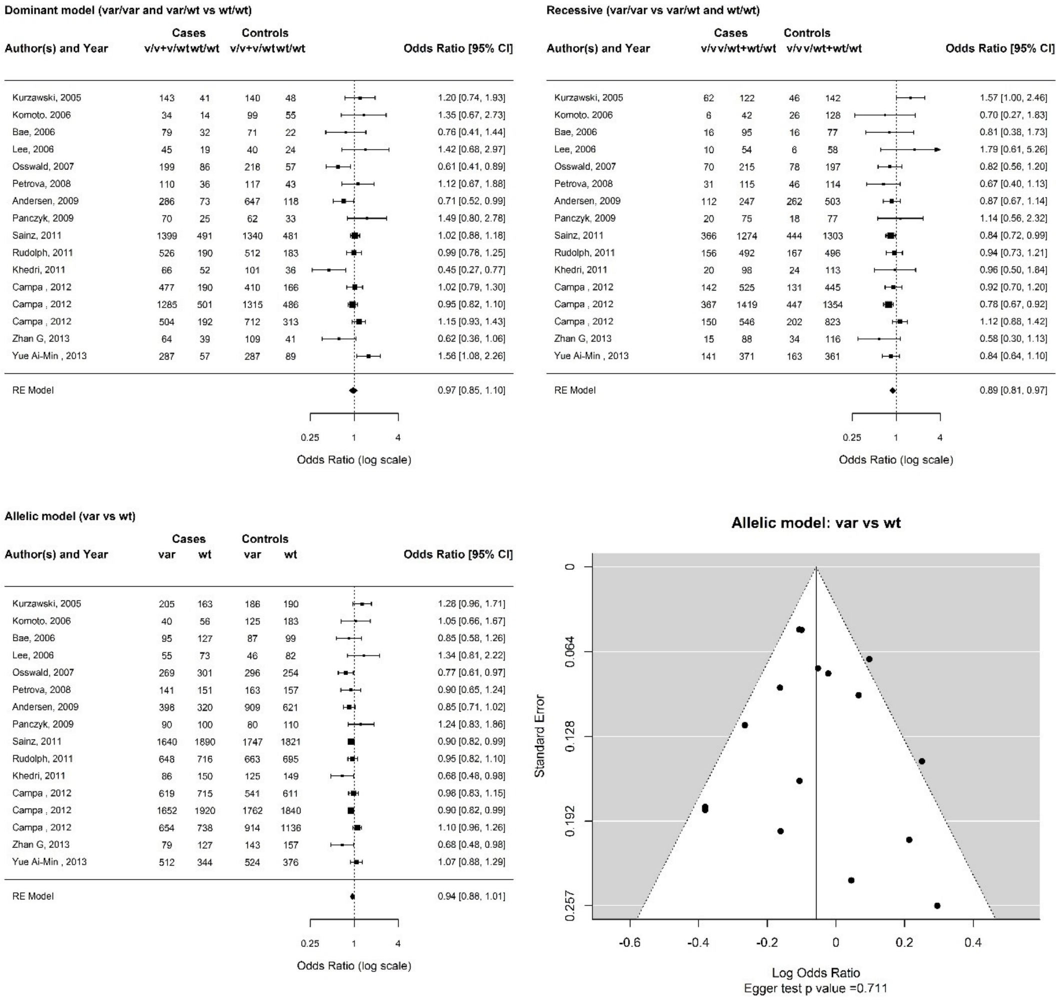

Supplementary Figure 46 Forest and Egger's plots for *ABCB1 (MDR1) (3435T>C, rs1045642)*.

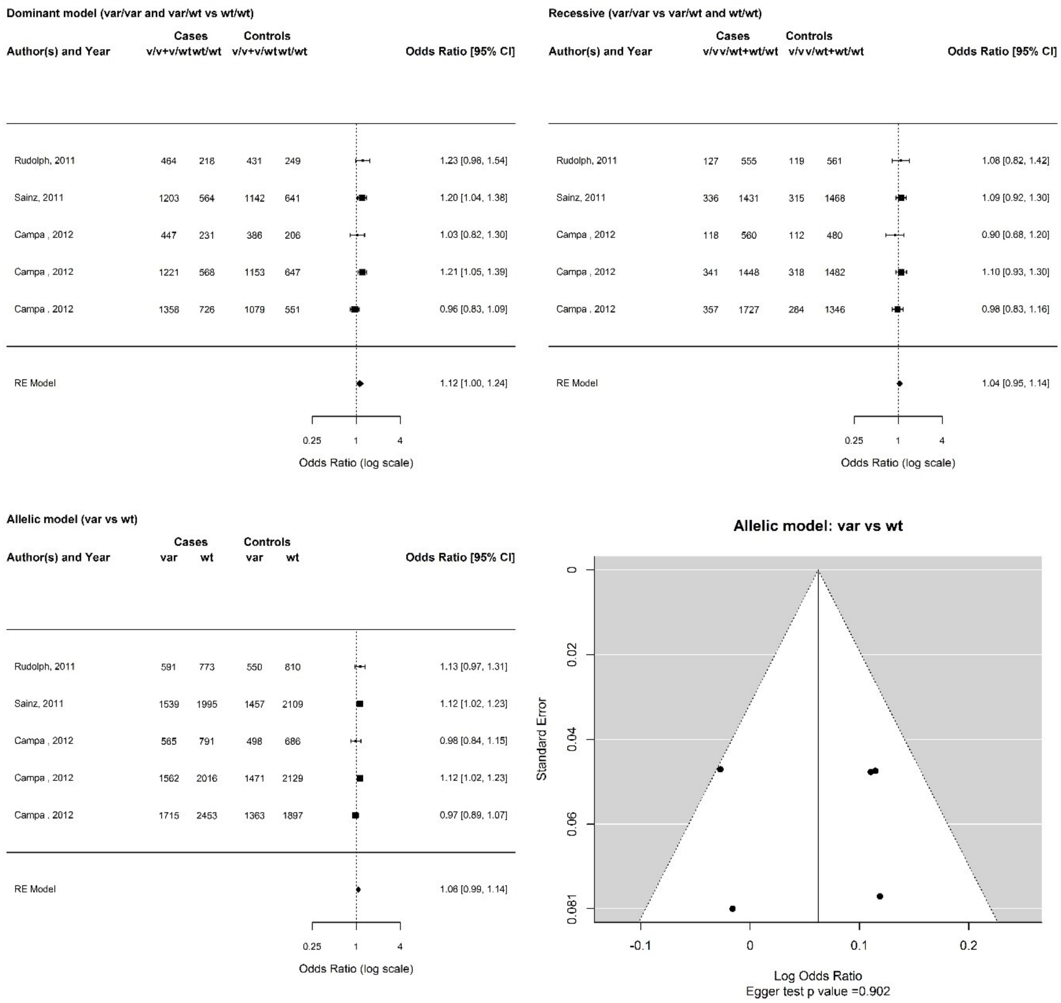

Supplementary Figure 47 Forest and Egger's plots for *ABCB1 (MDR1) (rs1202168*).

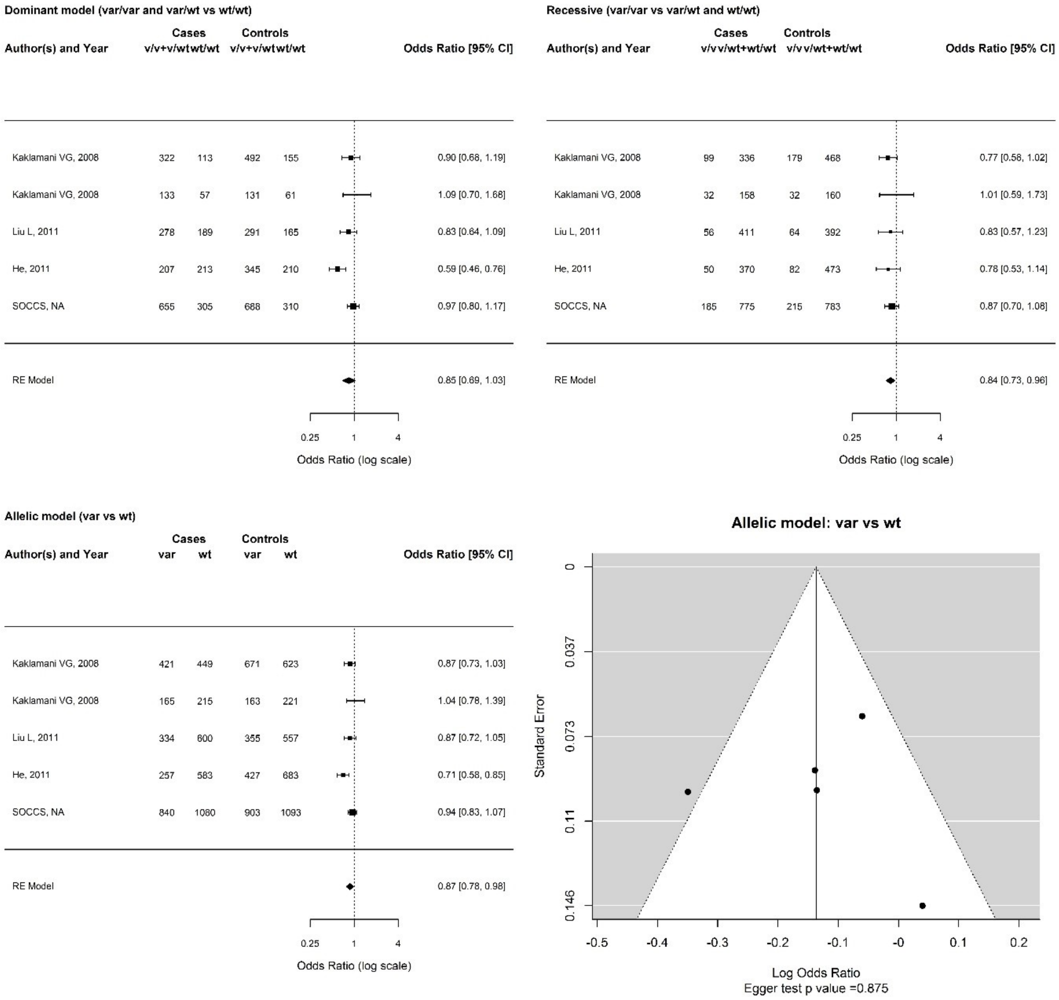

Supplementary Figure 48 Forest and Egger's plots for *ADIPOR1* (+5843G>A, rs1342387).

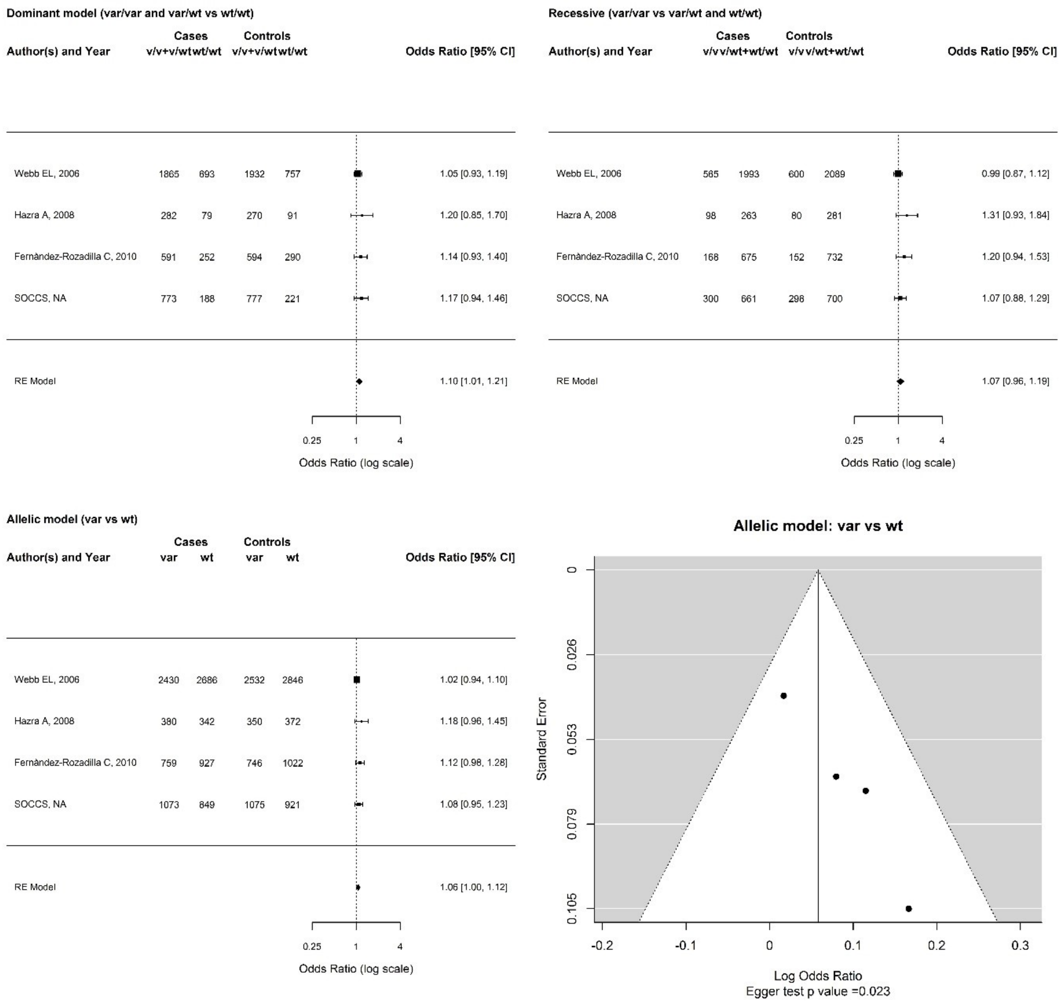

Supplementary Figure 49 Forest and Egger's plots for *AXIN2* (P50S, rs2240308).

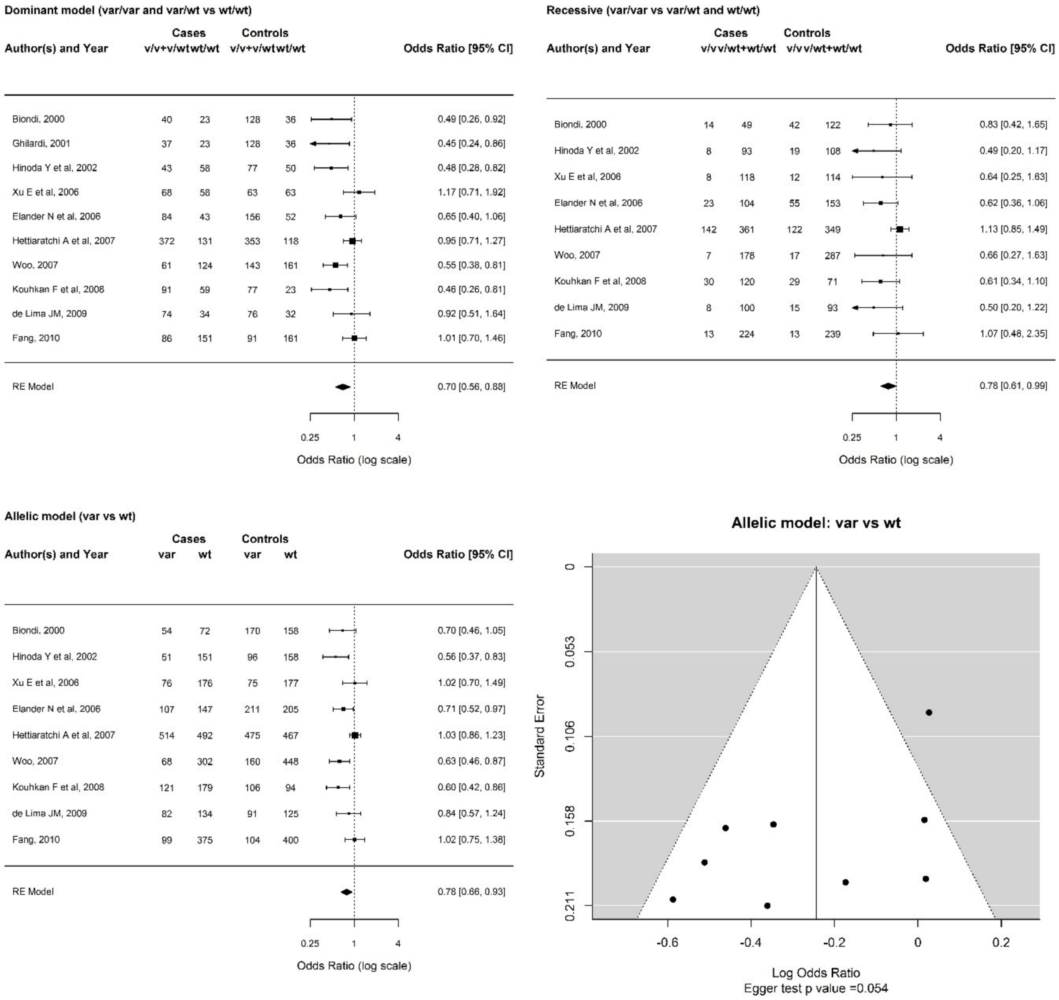

Supplementary Figure 50 Forest and Egger's plots for *MMP1* (G-1607GG, rs1799750).

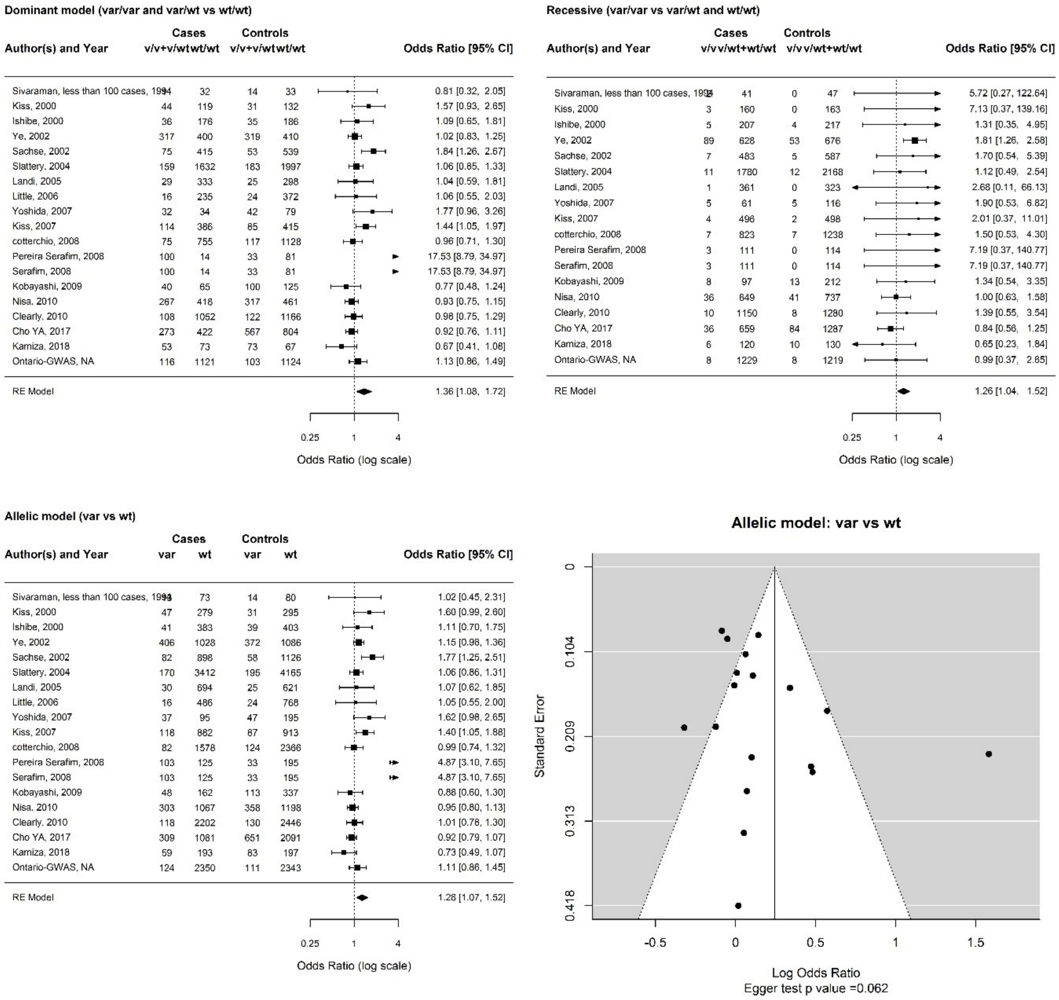

Supplementary Figure 51 Forest and Egger's plots for *CYP1A1* (2454A>G, rs1048943).

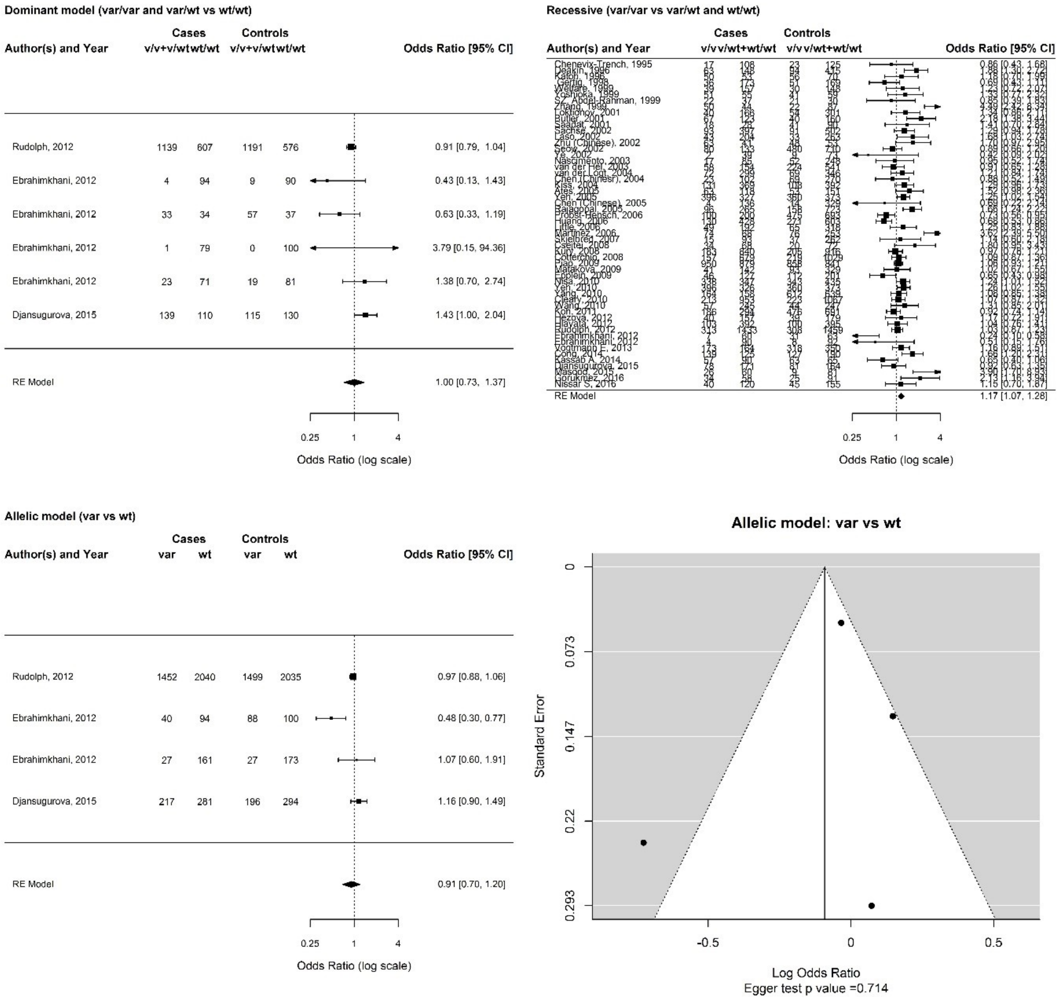

Supplementary Figure 52 Forest and Egger's plots for *GSTT1* (Null variant).

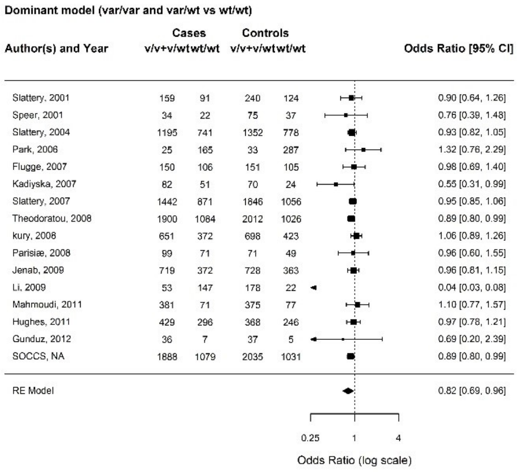

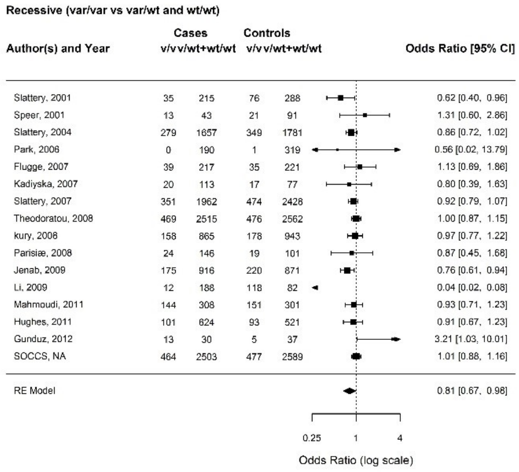

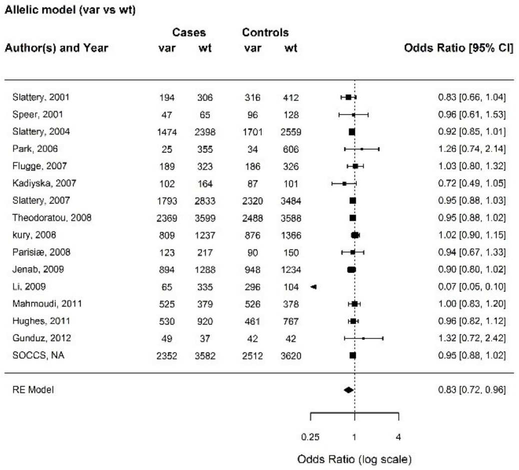

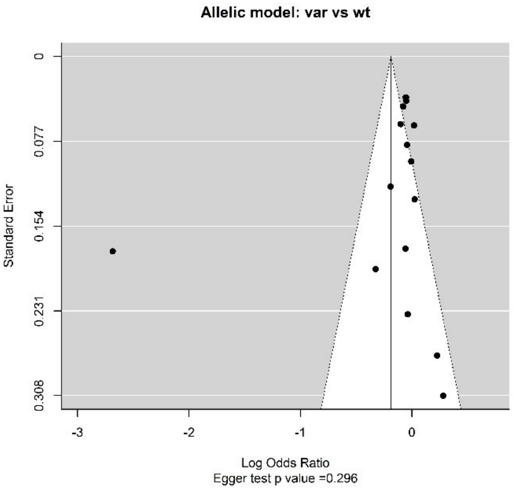

Supplementary Figure 53 Forest and Egger's plots for *VDR BsmI* (60890GA, rs1544410).

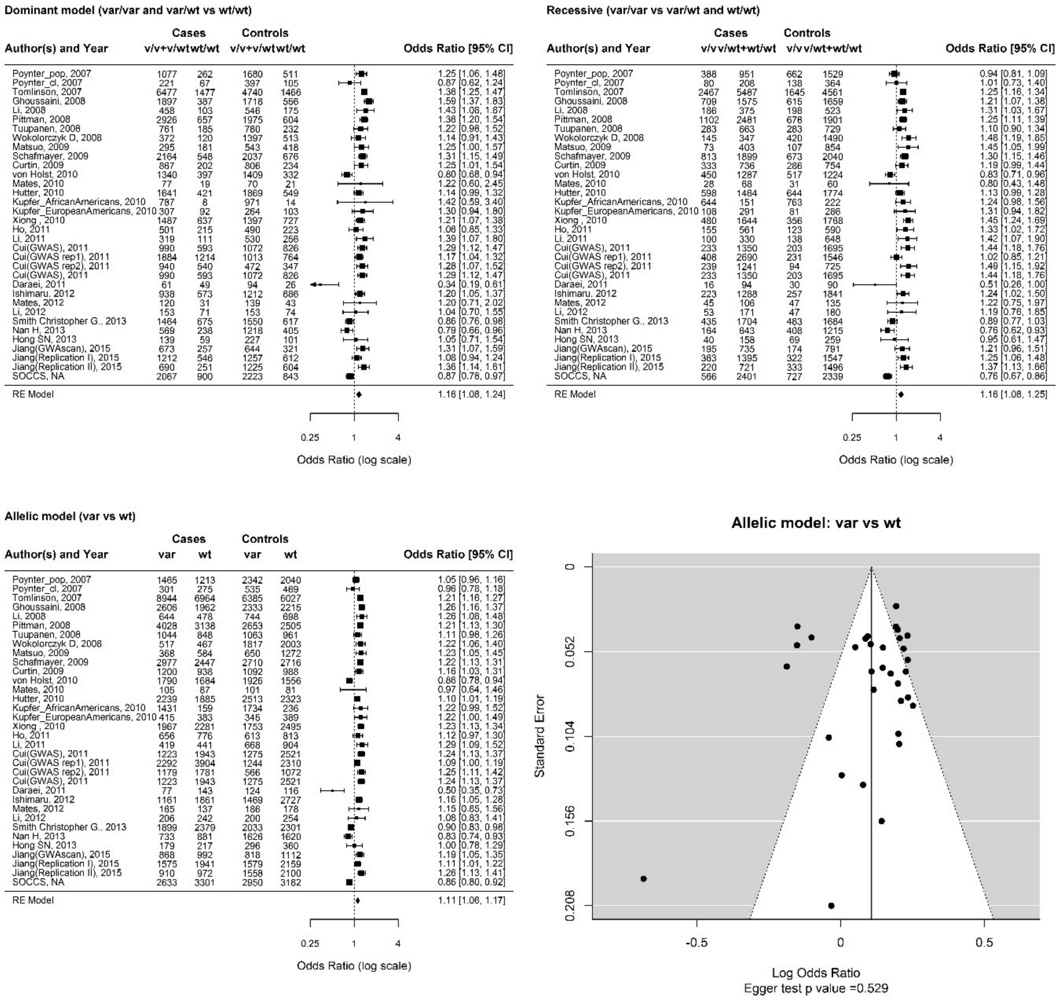

Supplementary Figure 54 Forest and Egger's plots for 8q24 (rs6983267).

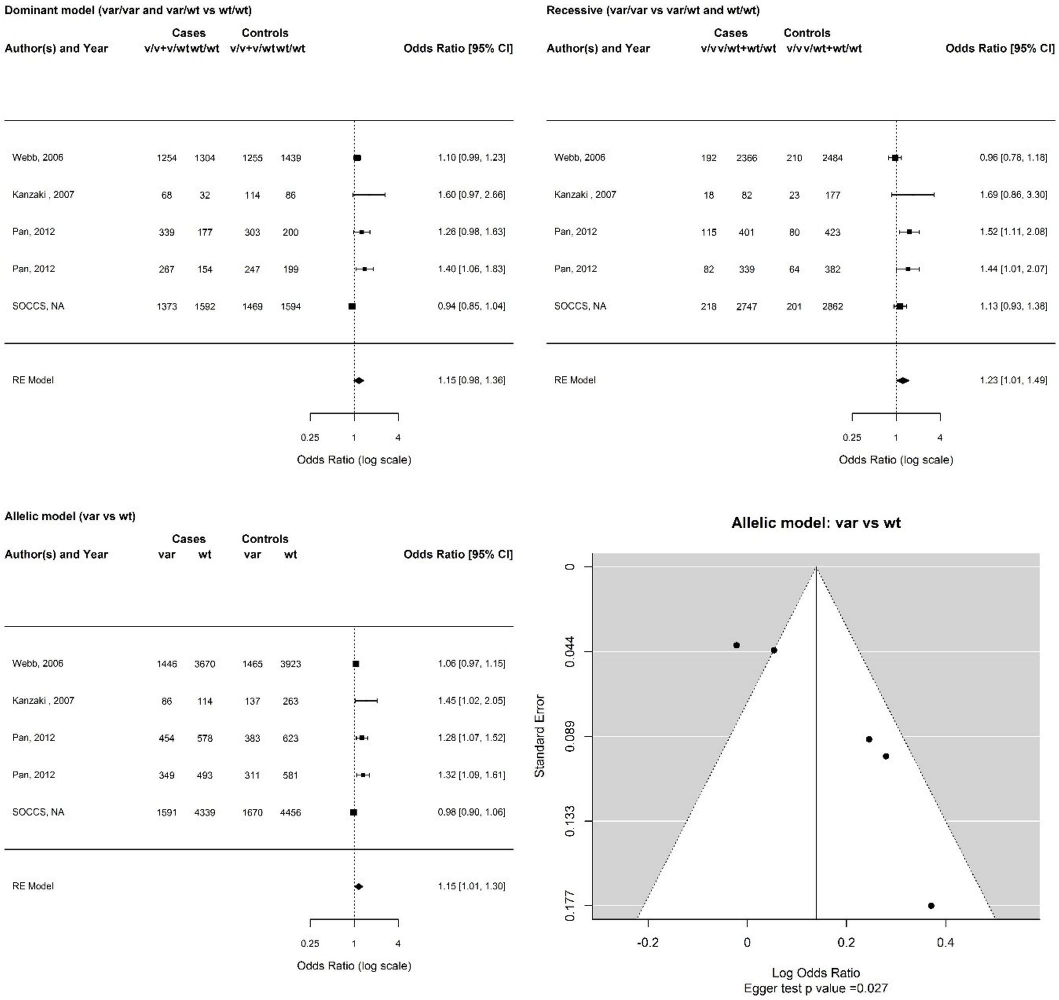

Supplementary Figure 55 Forest and Egger’s plots for *RAD18* (905G>A, rs373572).

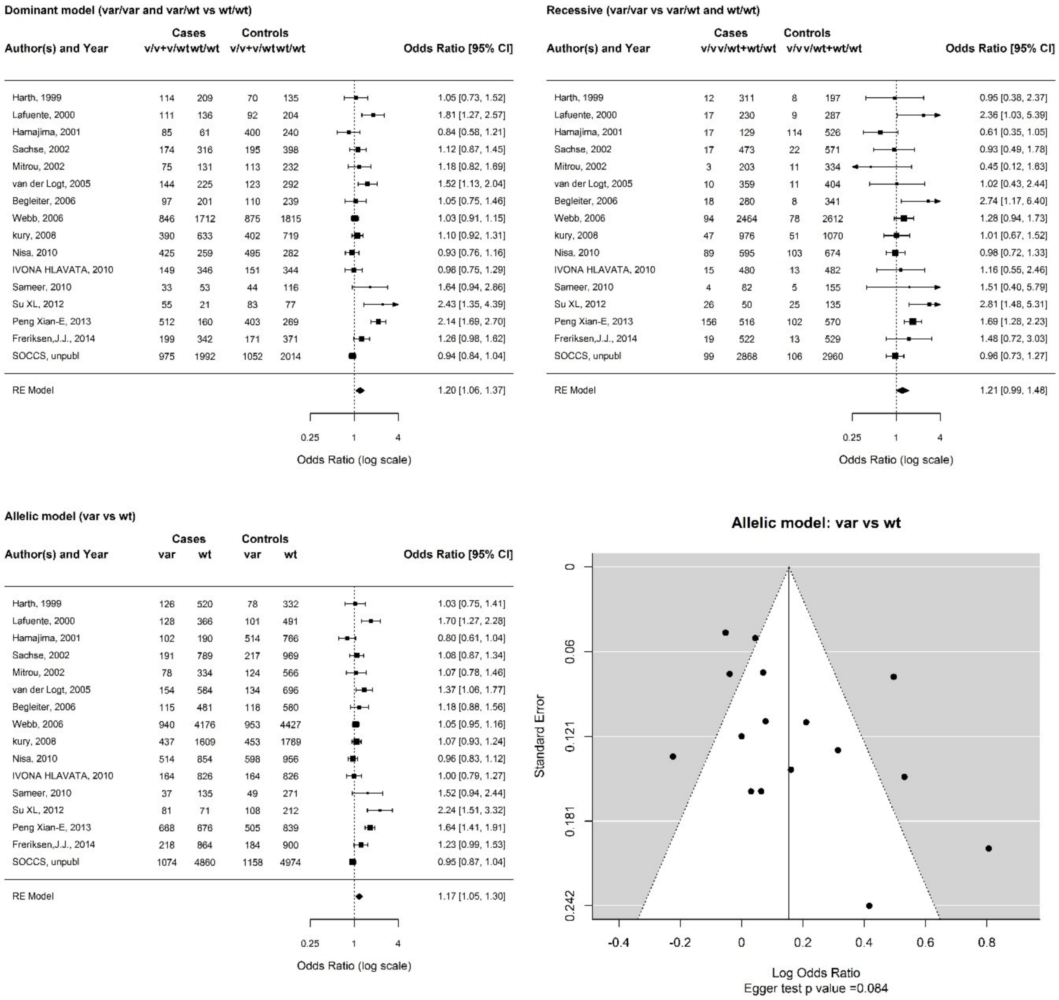

Supplementary Figure 56 Forest and Egger's plots for *NQ01* (Pro187Ser [C609T], rs1800566).

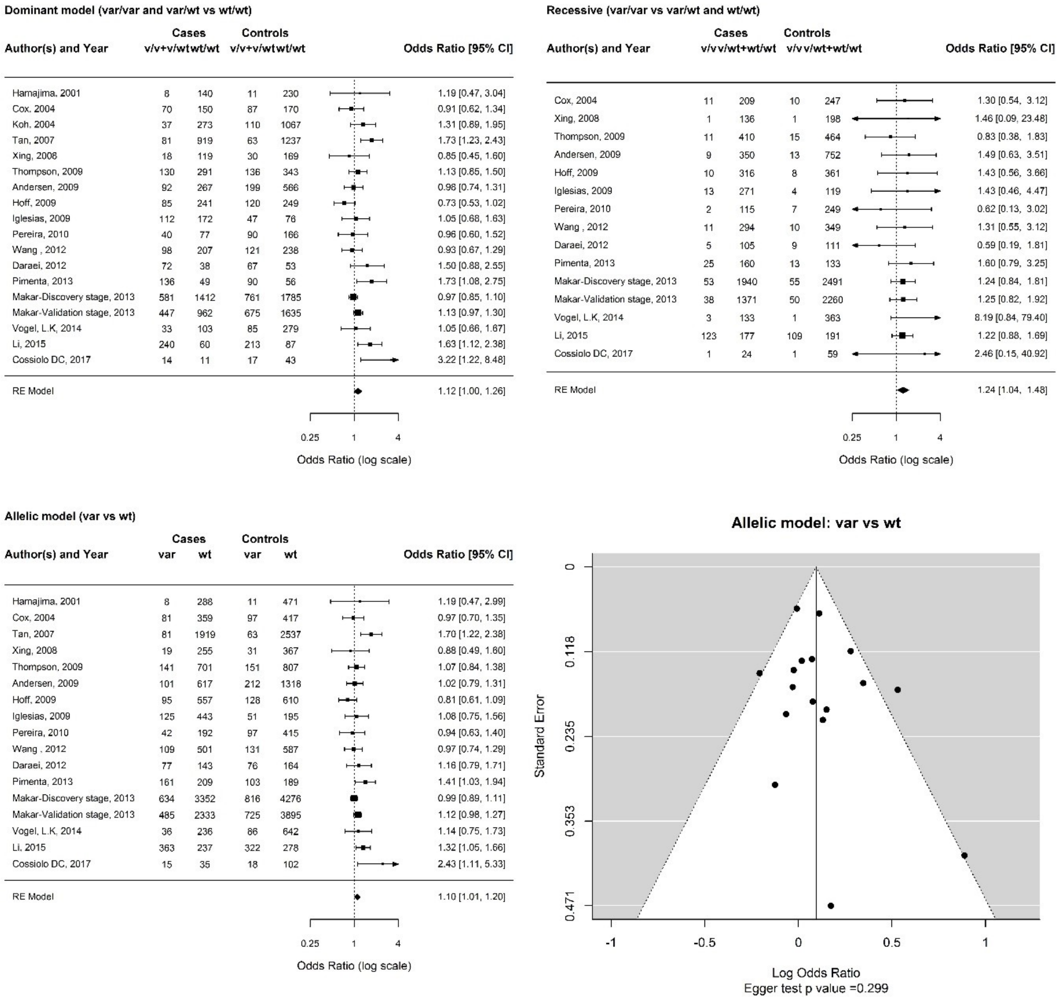

Supplementary Figure 57 Forest and Egger's plots for *PTGS2/COX2* (G765C, rs20417).

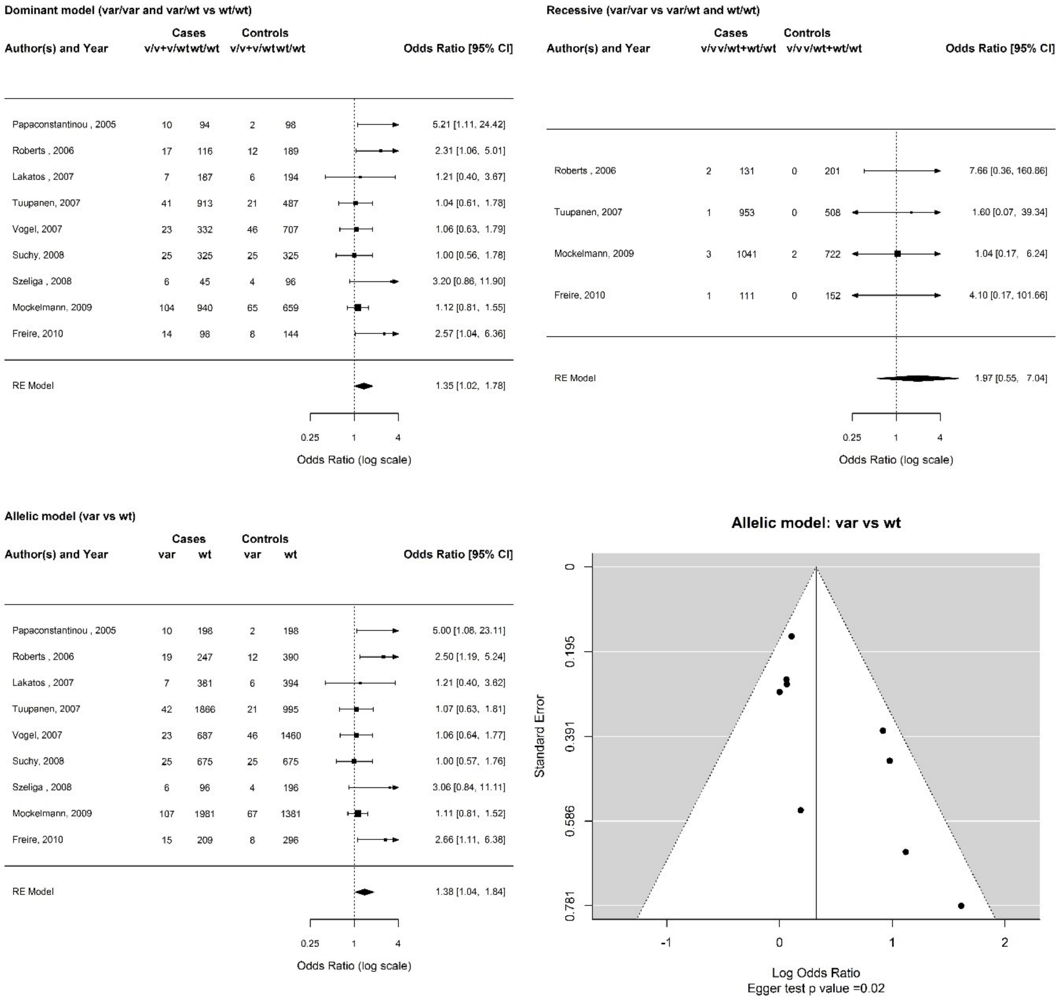

Supplementary Figure 58 Forest and Egger's plots for *NOD2* (R702W, rs2066844).

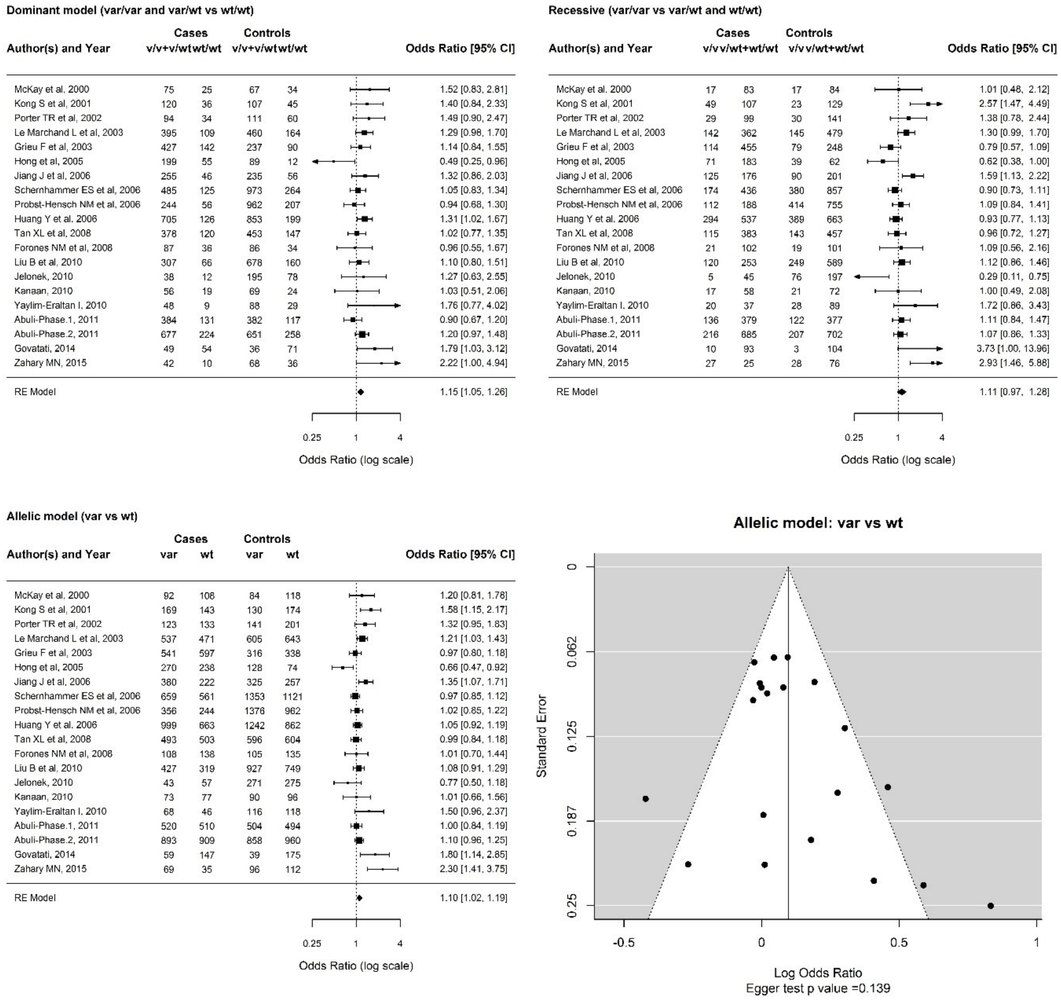

Supplementary Figure 59 Forest and Egger’s plots for *CCND1* (870A, rs17852153).

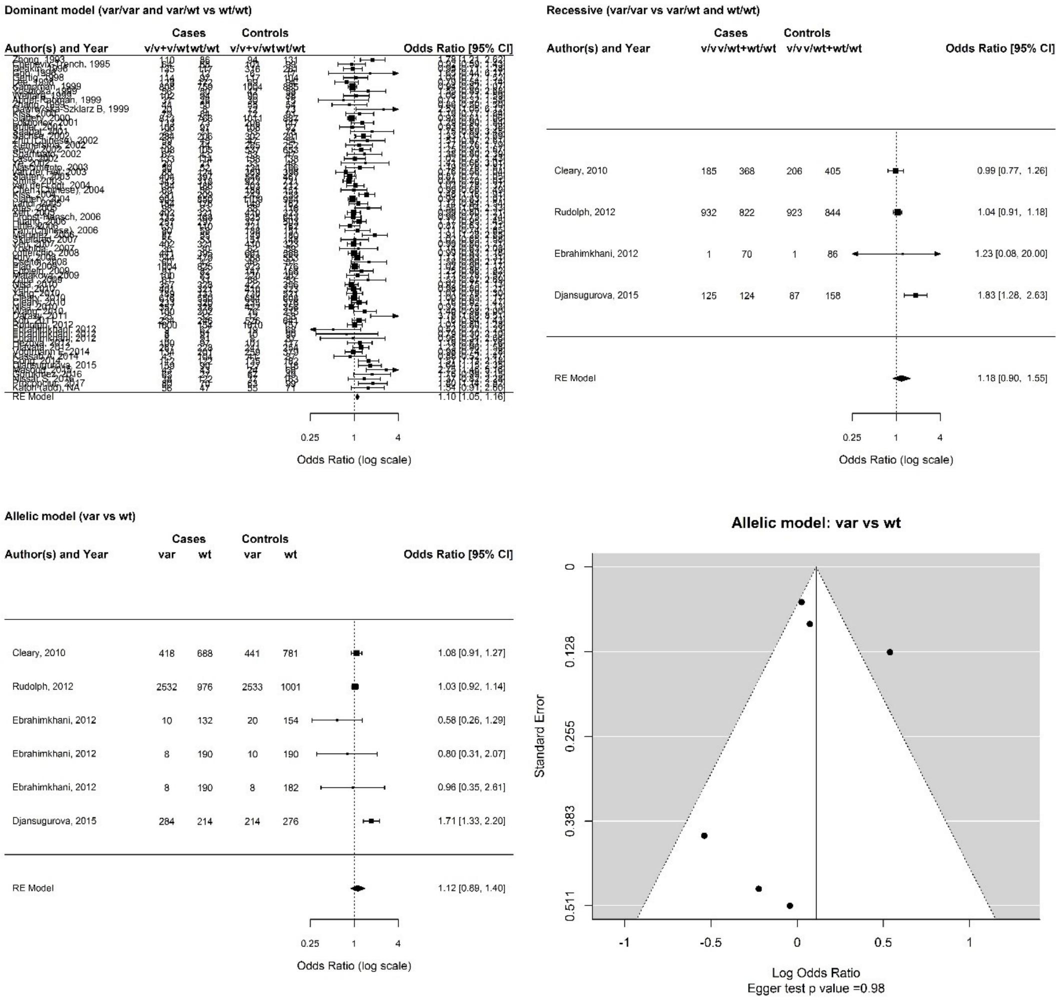

Supplementary Figure 60 Forest and Egger's plots for *GSTM1* (Null variant).

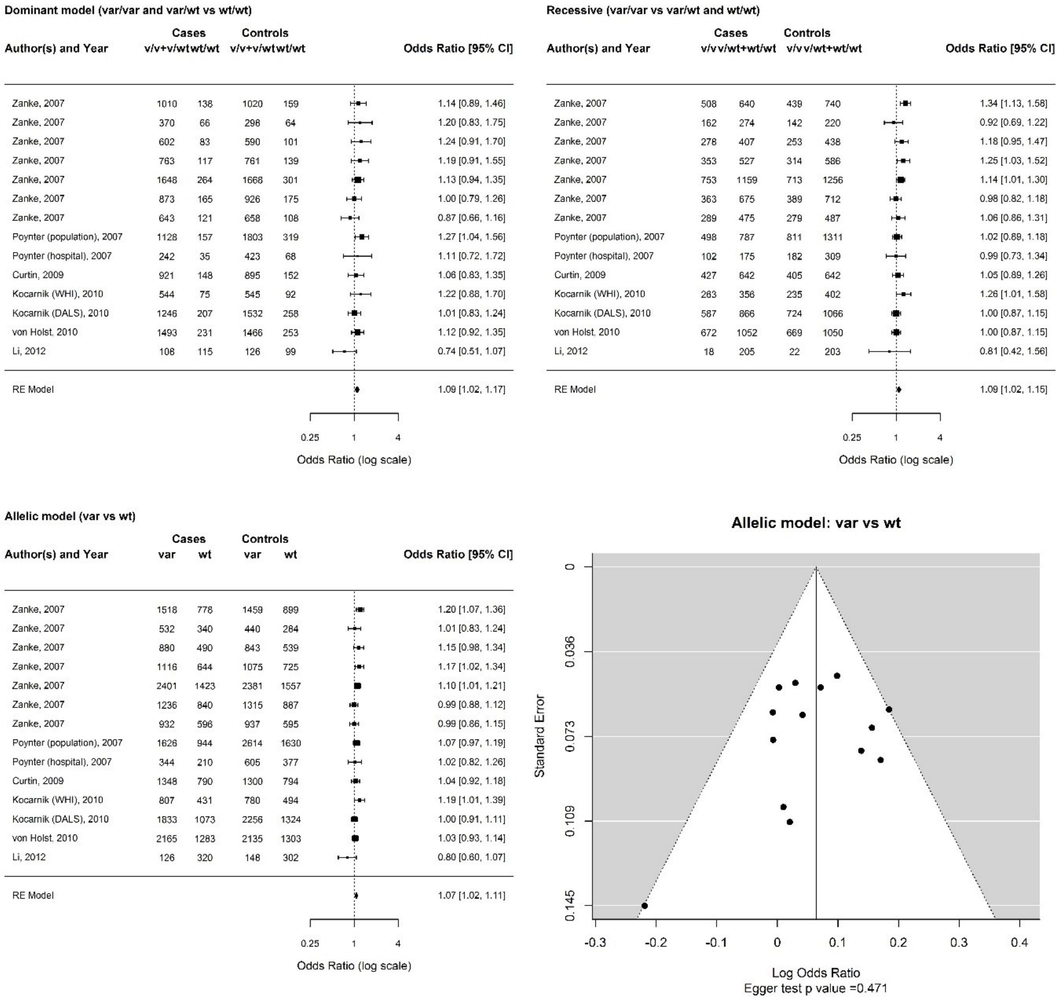

Supplementary Figure 61 Forest and Egger’s plots for 9p24 (rs719725).

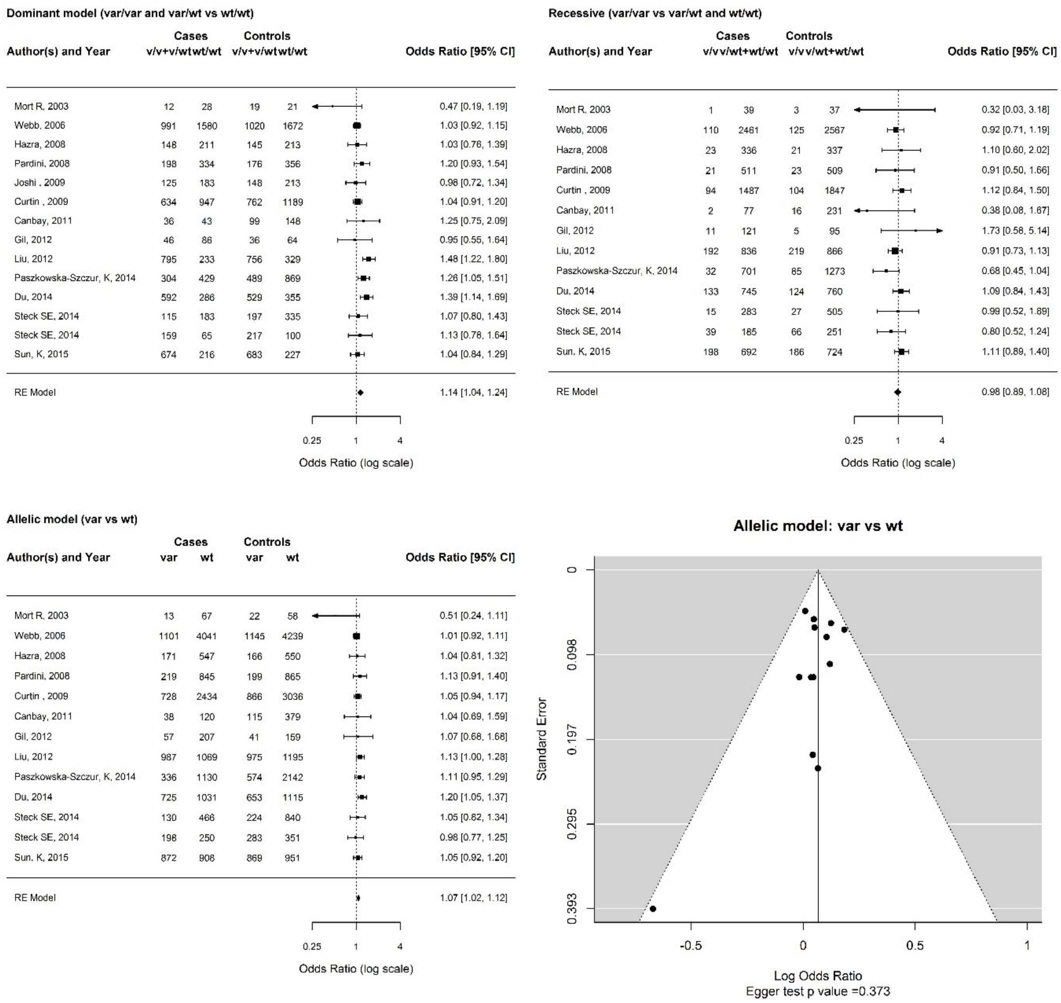

Supplementary Figure 62 Forest and Egger’s plots for *ERCC5* (D1104H, rs17655).

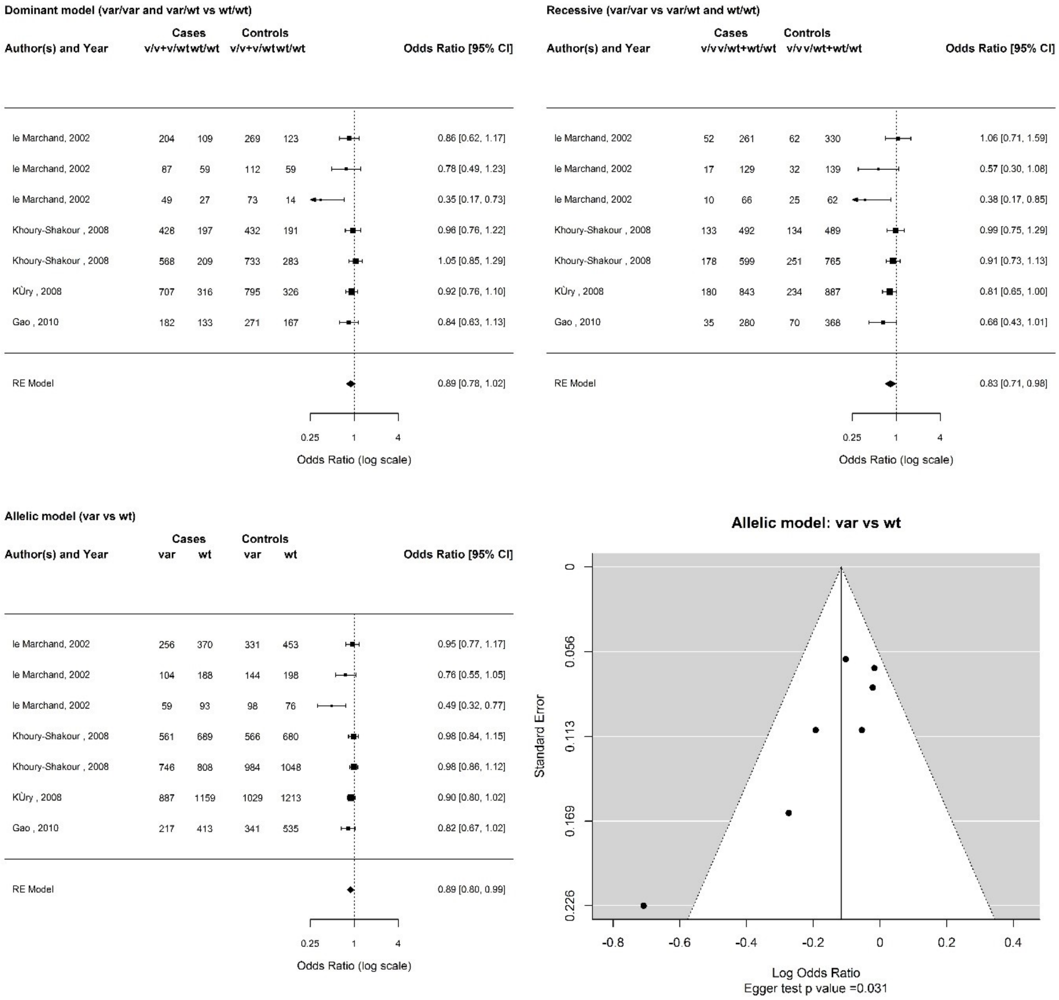

Supplementary Figure 63 Forest and Egger's plots for *GH1* (1663T>A, rs2665802).

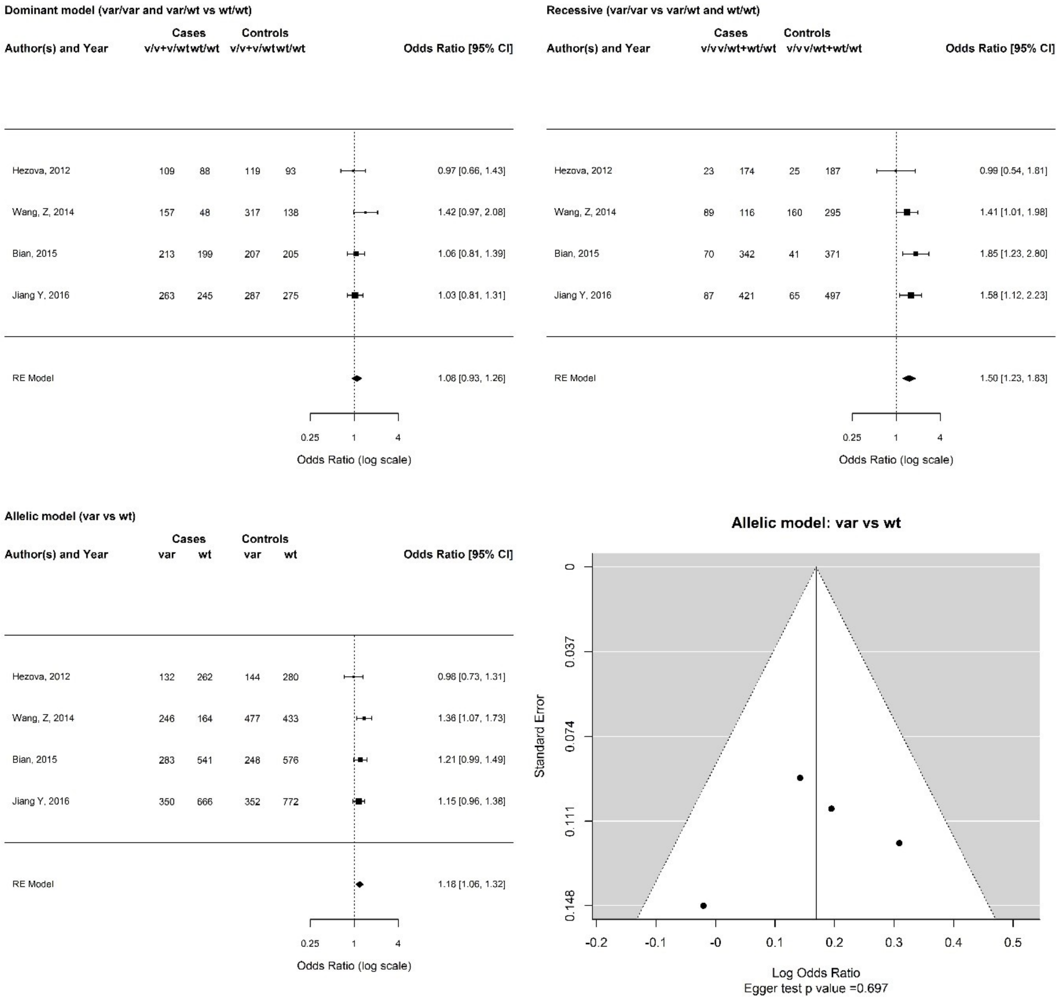

Supplementary Figure 64 Forest and Egger's plots for *miR* (pre-miR-27a, rs895819).

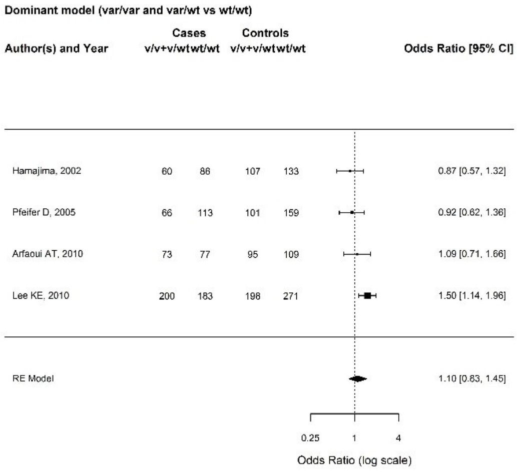

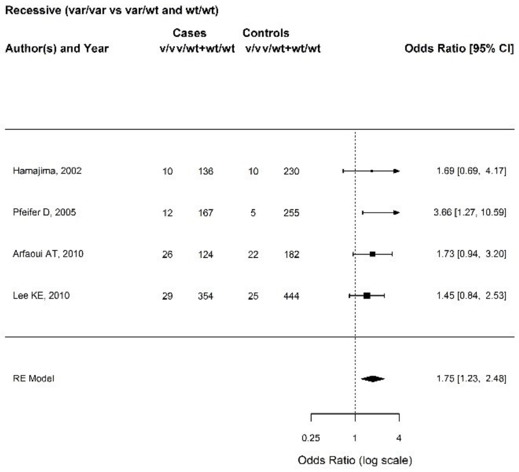

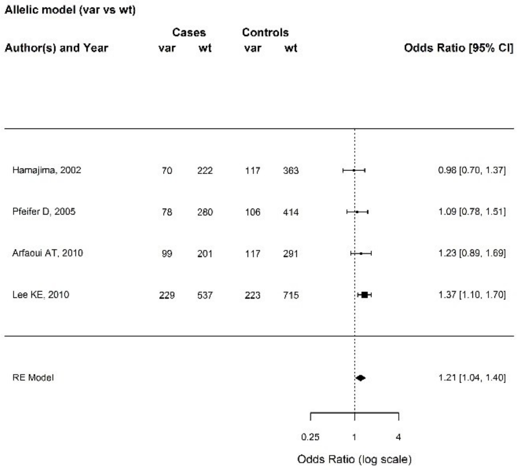

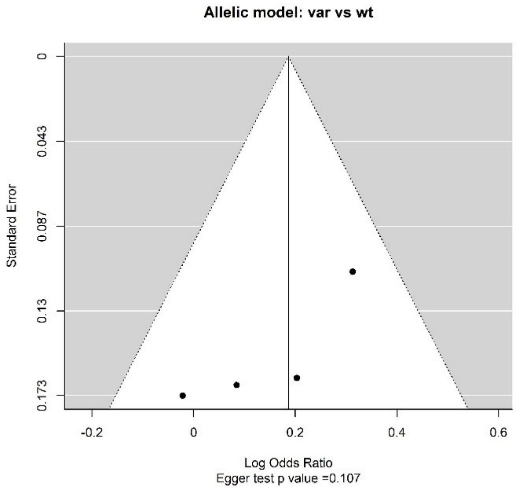

Supplementary Figure 65 Forest and Egger's plots for *TP73* (G4C14).

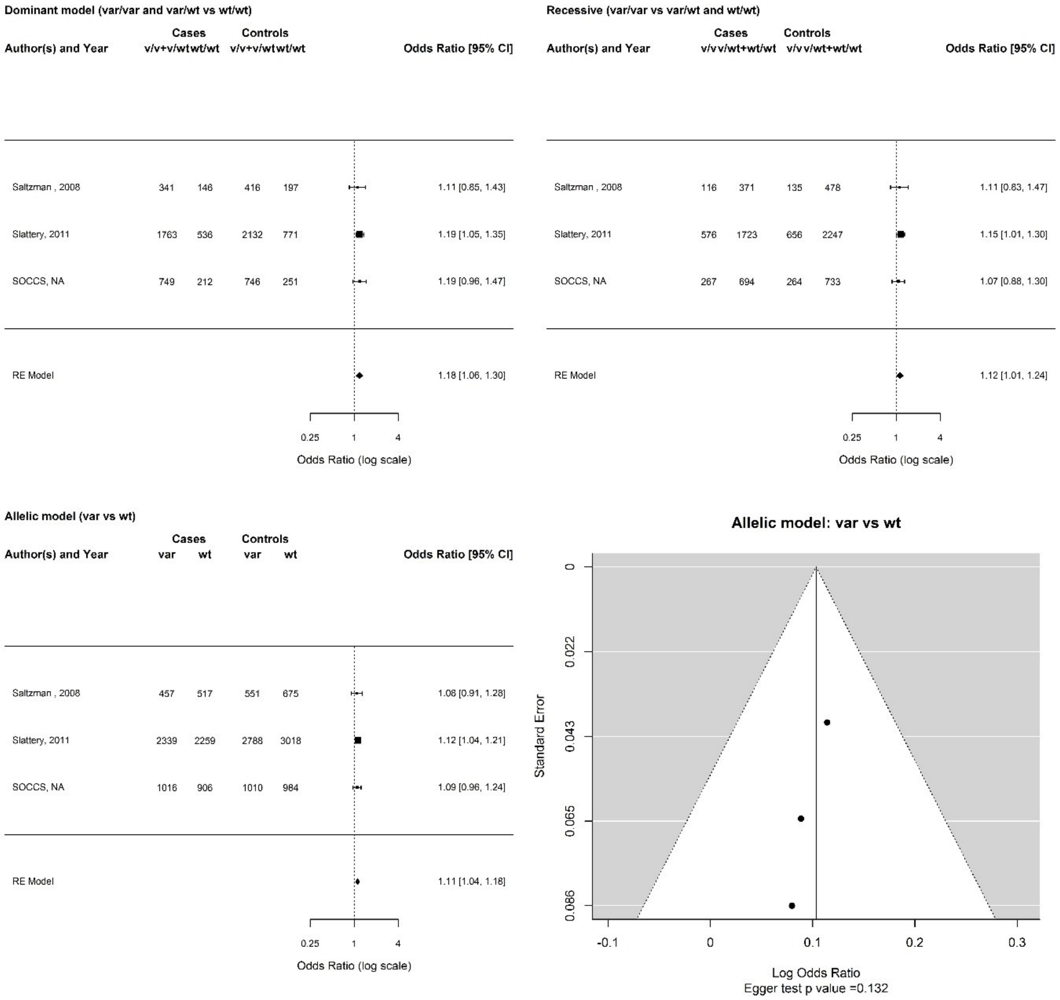

Supplementary Figure 66 Forest and Egger's plots for *TGFB1* (rs4803455).

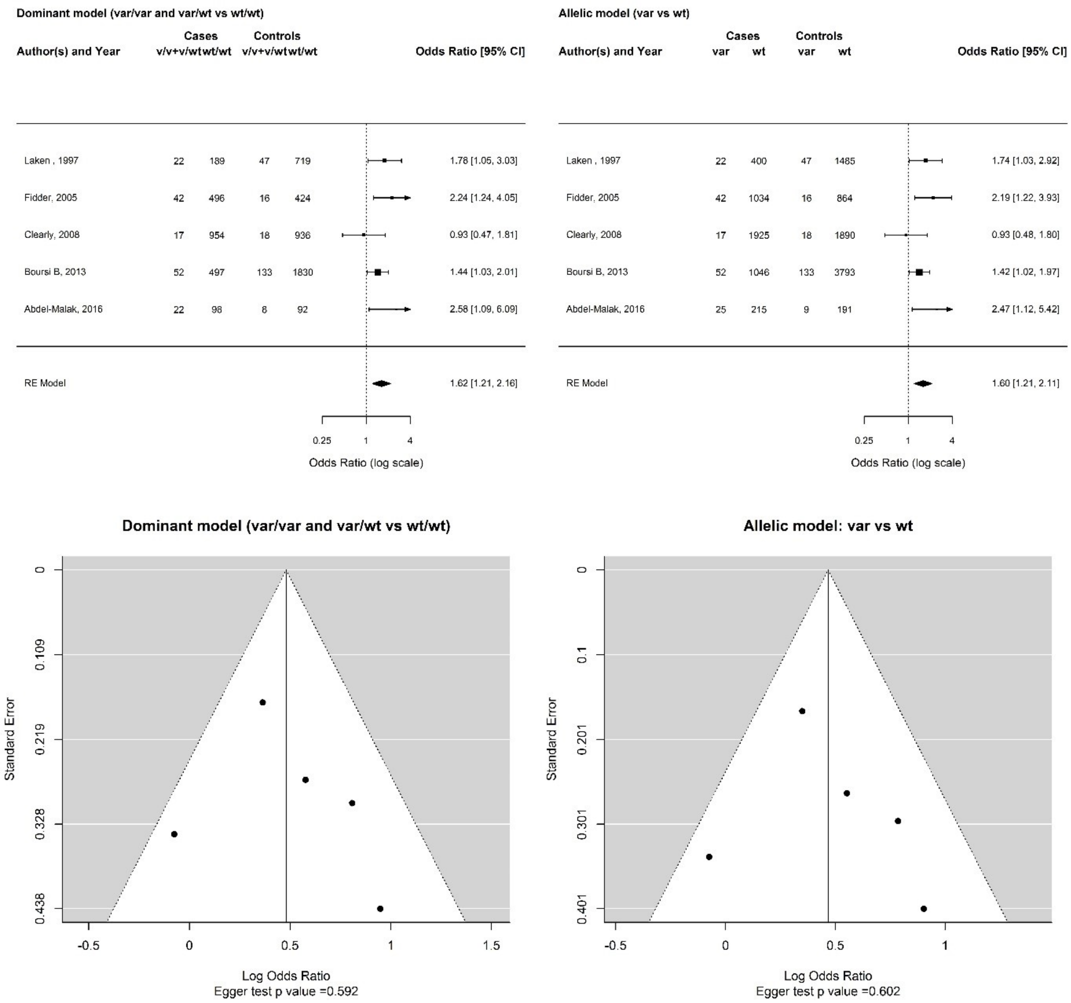

Supplementary Figure 67 Forest and Egger's plots for *APC* (I1307K, rs1801155).

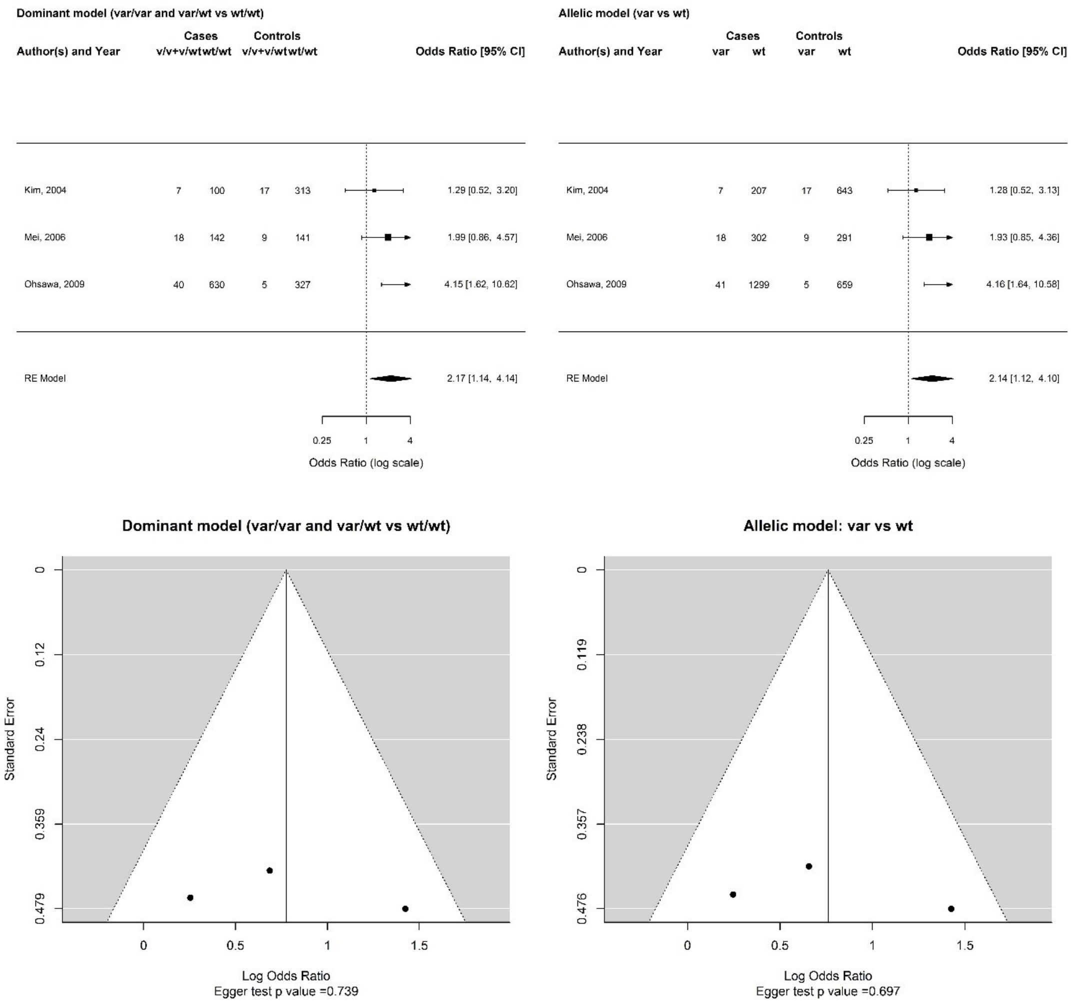

Supplementary Figure 68 Forest and Egger's plots for *MLH1* (V384D, rs63750447).

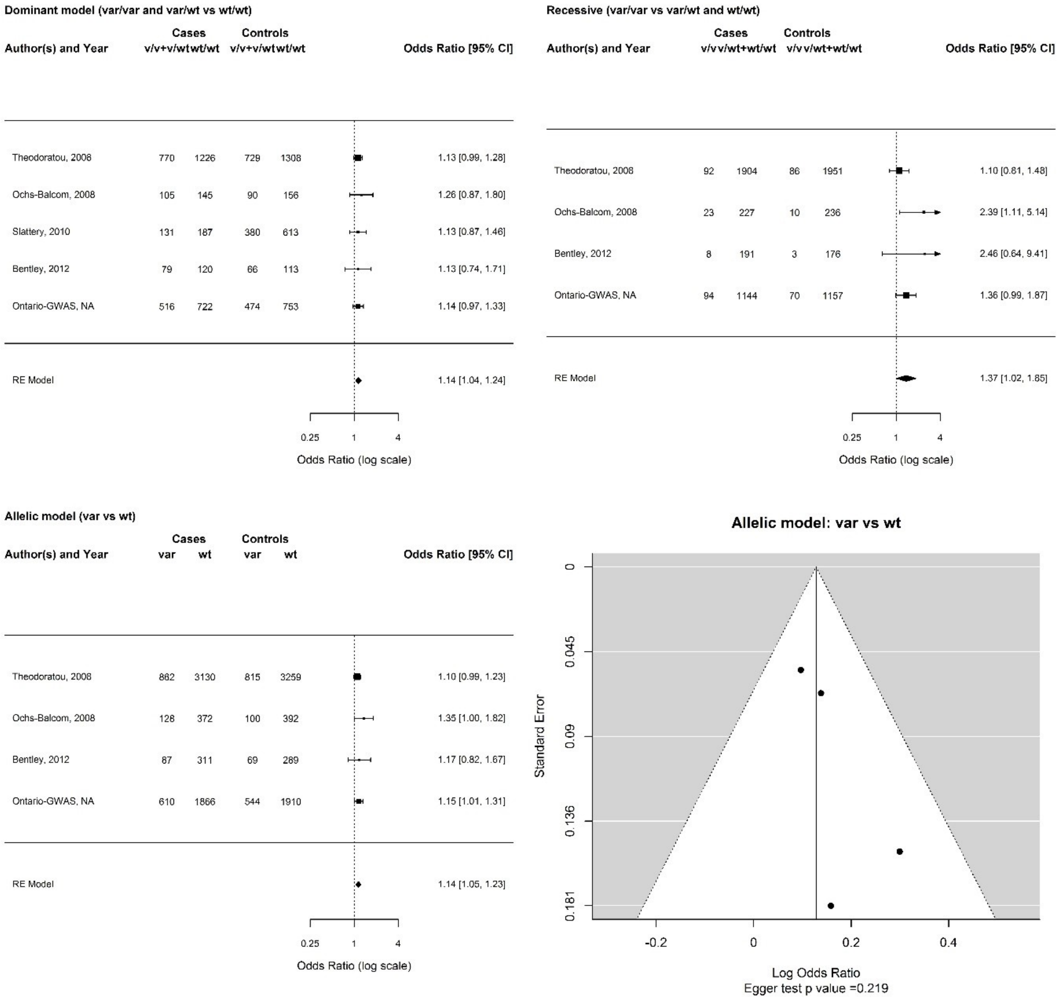

Supplementary Figure 69 Forest and Egger's plots for *VDR* (29648A>G, rs11568820).

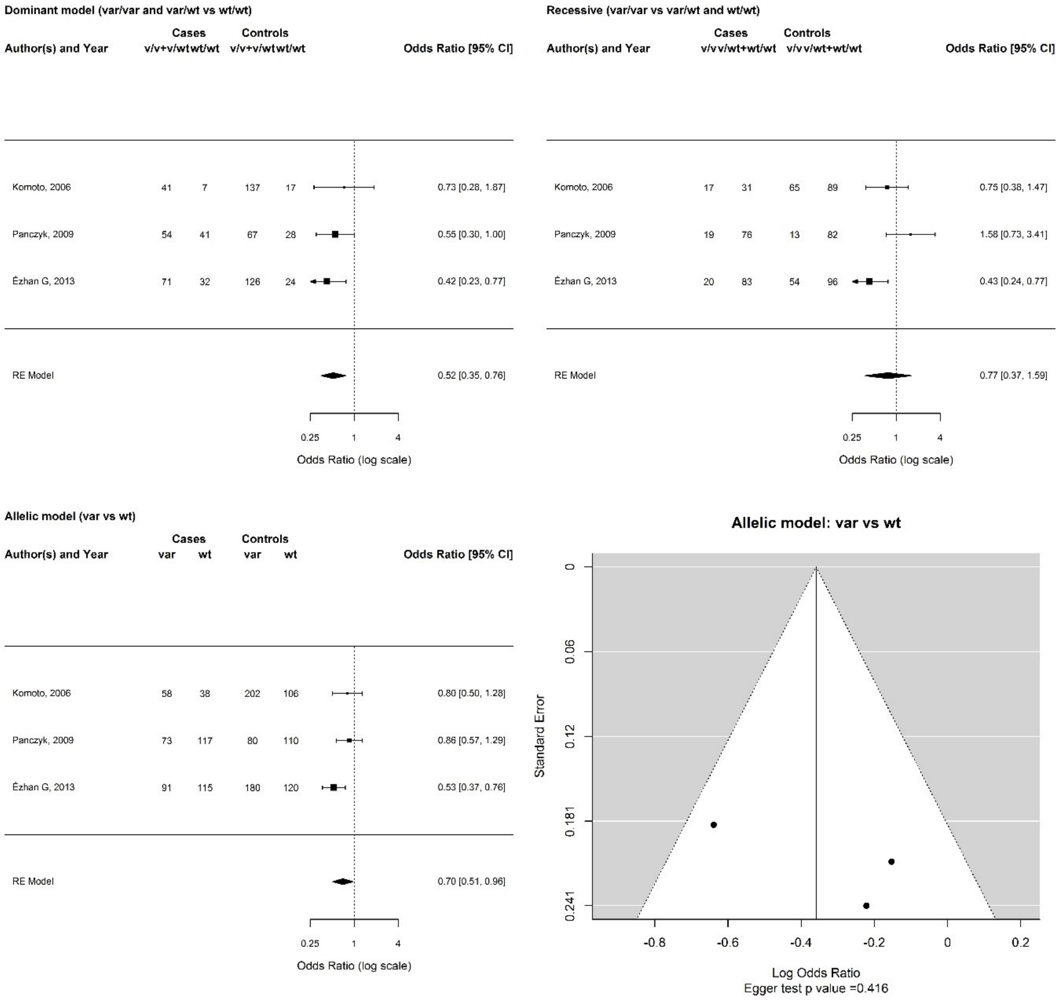

Supplementary Figure 70 Forest and Egger's plots for *ABCB1 (MDR1)* (C1236T, rs1128503).

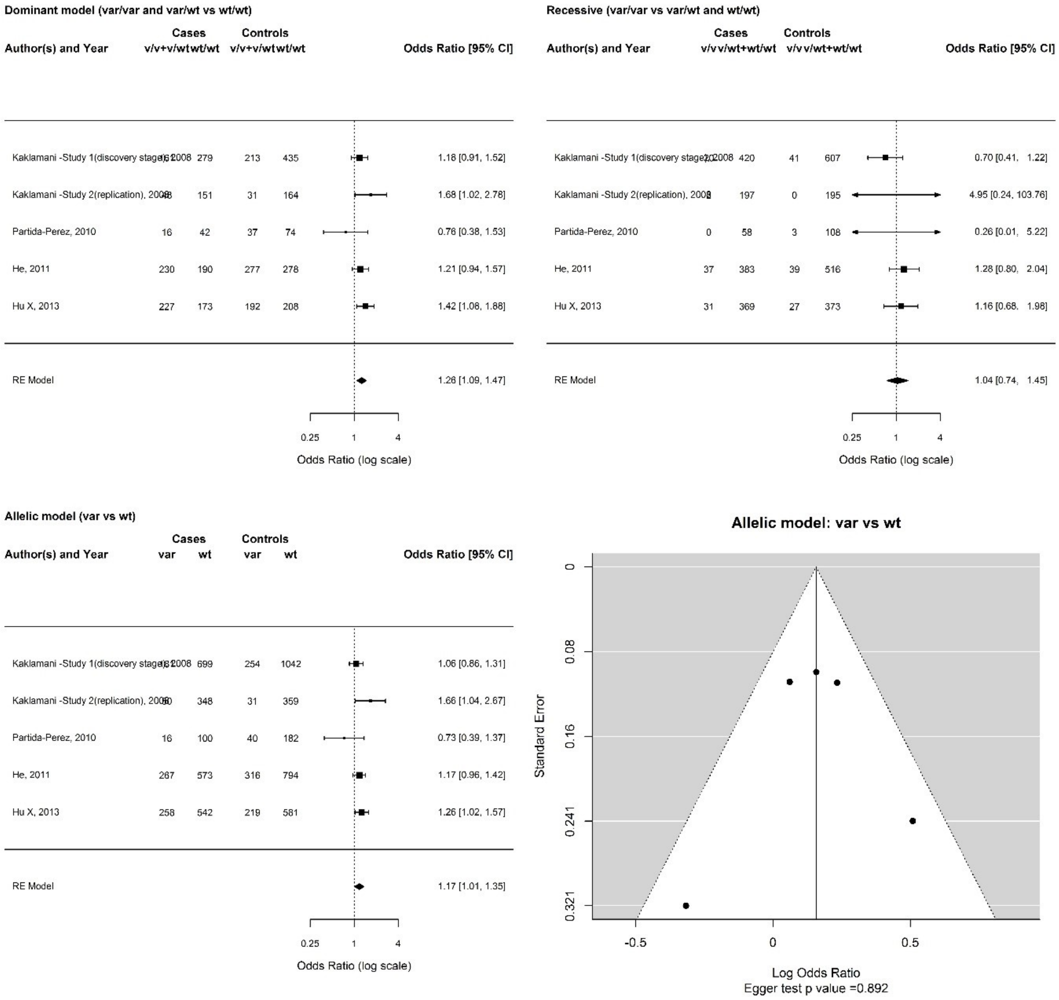

Supplementary Figure 71 Forest and Egger's plots for *ADIPOQ* (45 T>G, rs2241766).

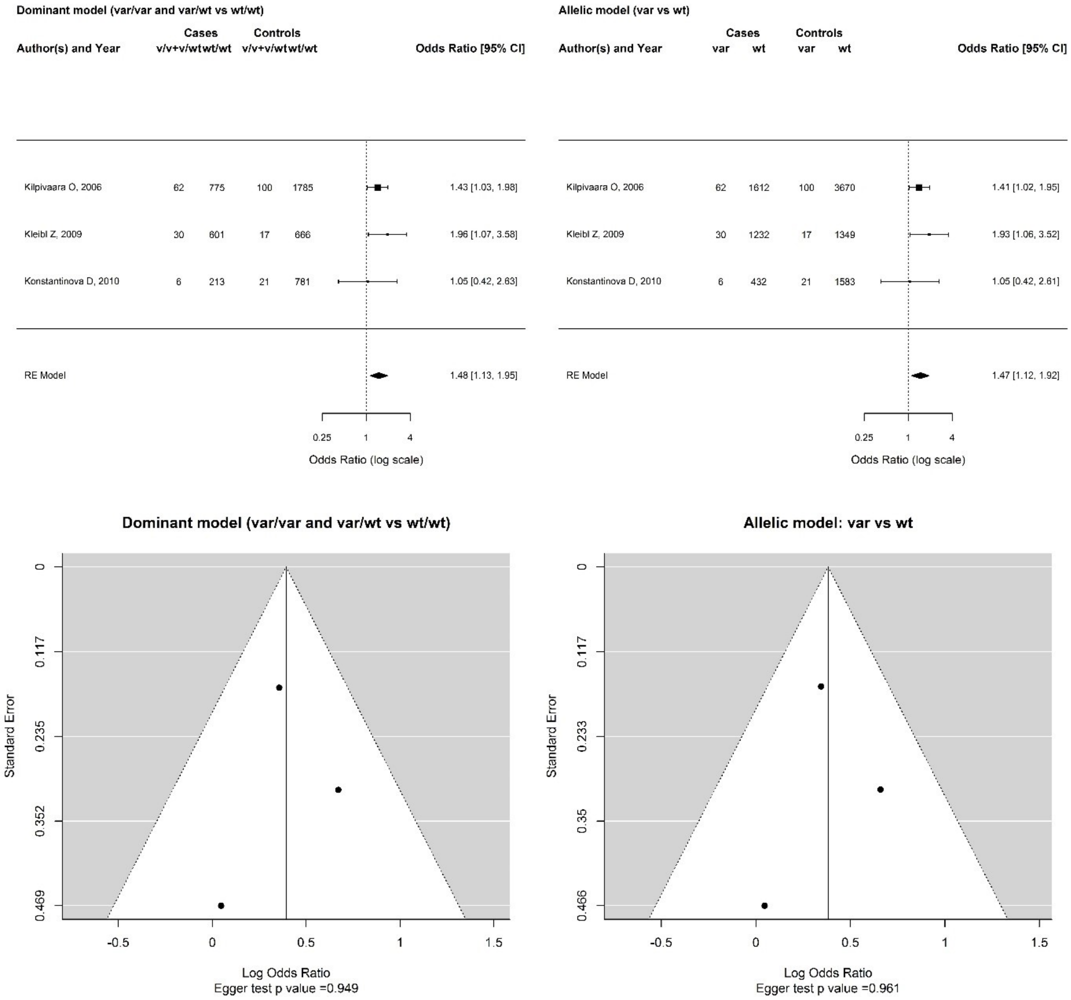

Supplementary Figure 72 Forest and Egger's plots for *CHEK2* (c.470T>C, rs1787996).

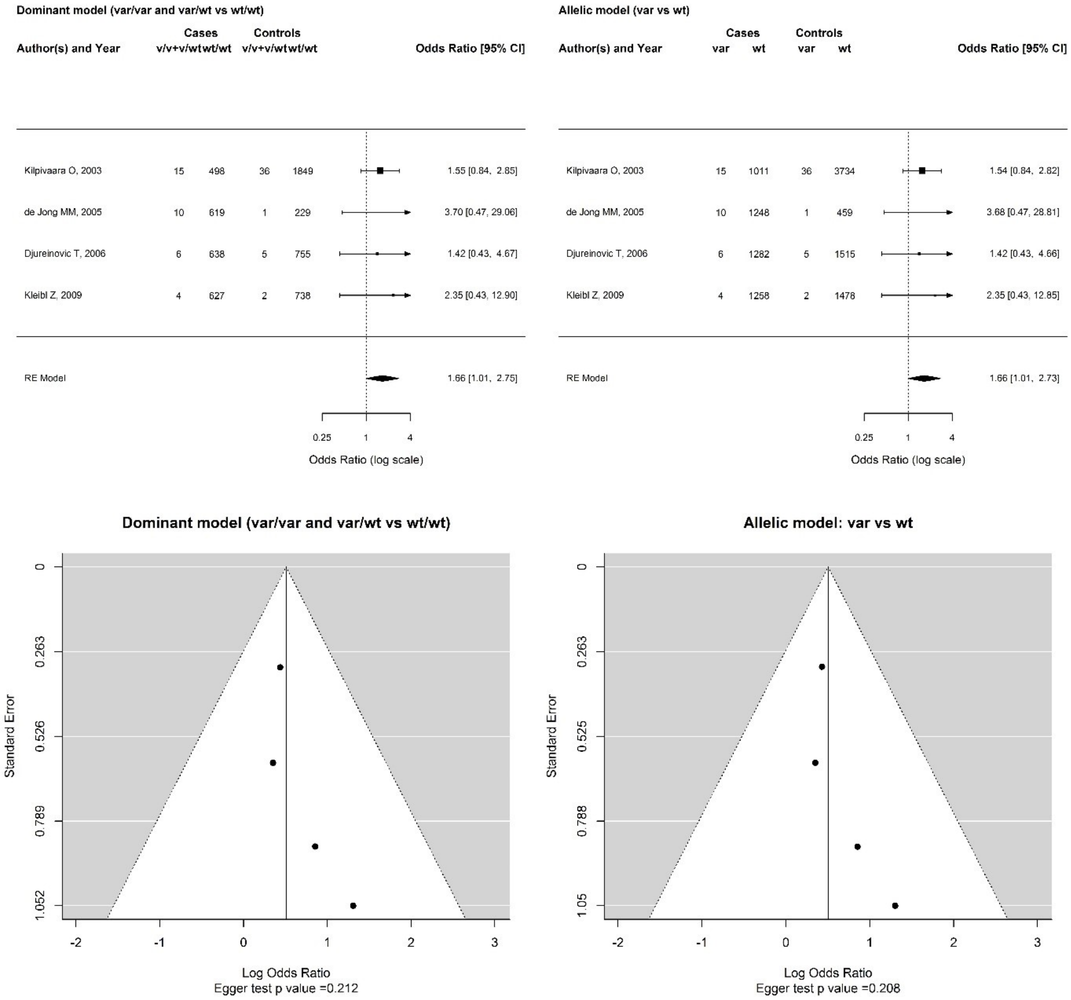

Supplementary Figure 73 Forest and Egger's plots for *CHEK2* (1100delC).

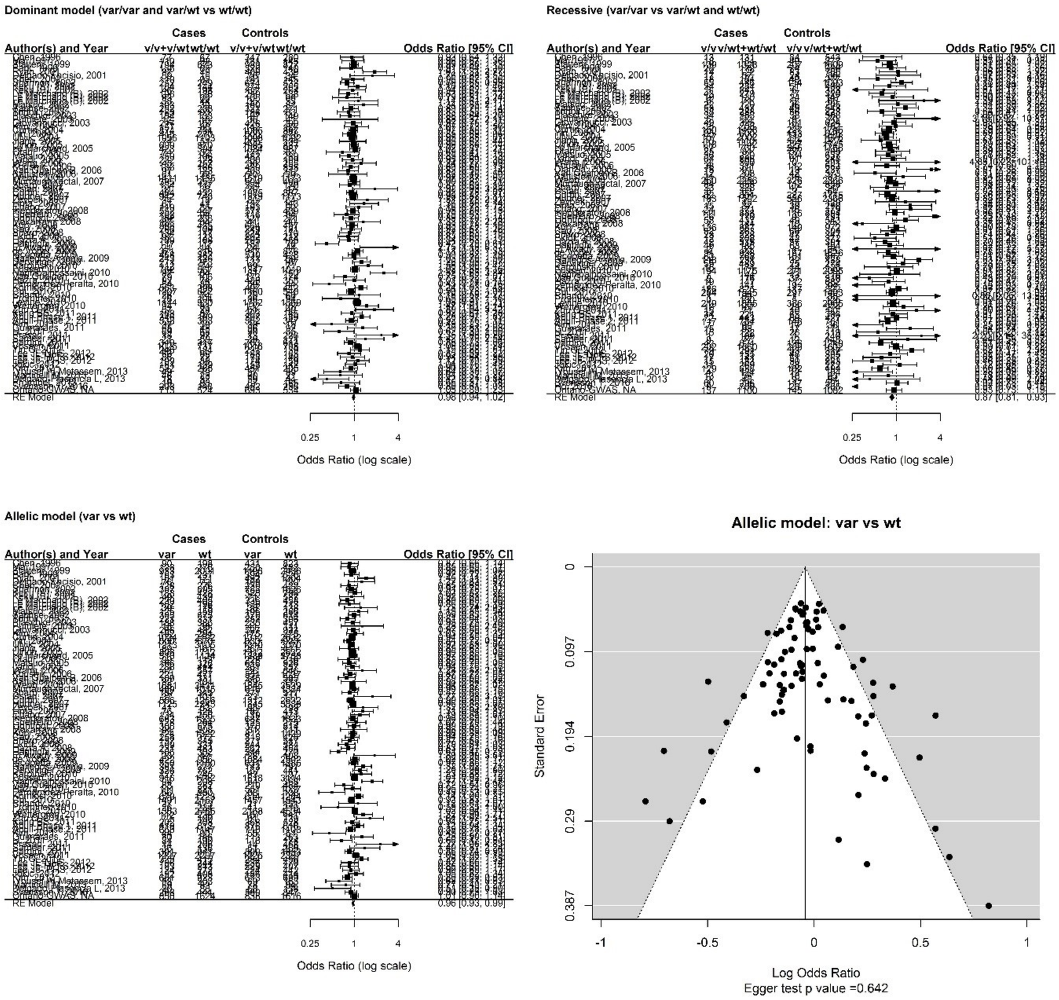

Supplementary Figure 74 Forest and Egger's plots for *MTHFR* (C677T, rs1801133).

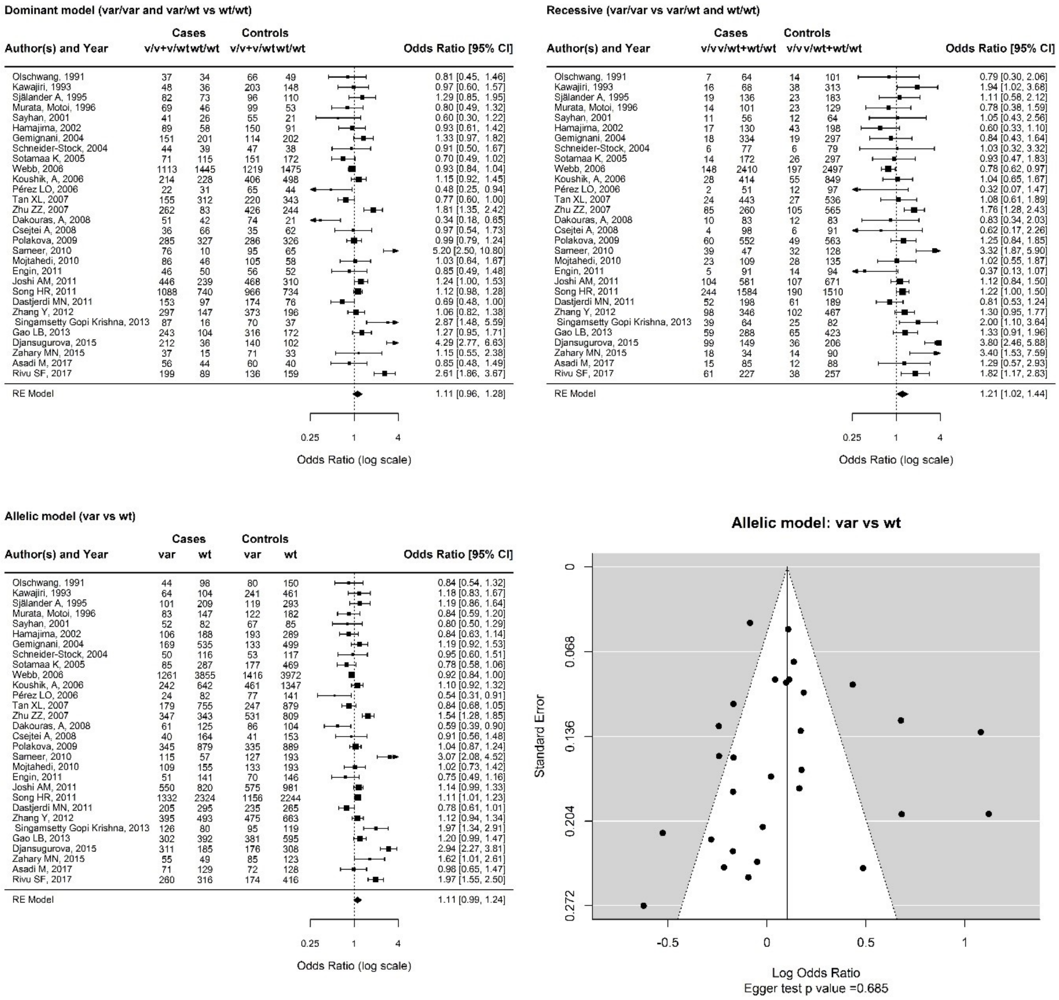

Supplementary Figure 75 Forest and Egger's plots for *TP53* (Arg72Pro, rs1042522).

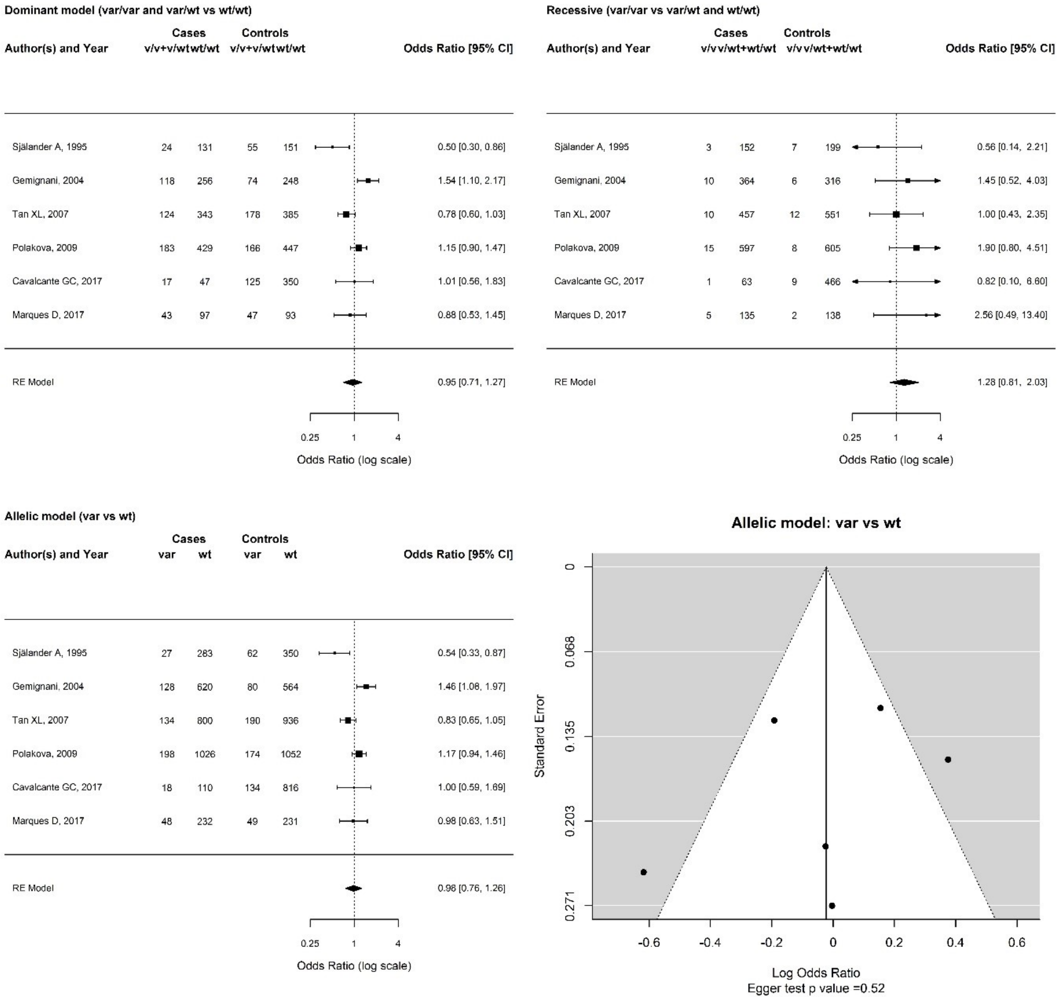

Supplementary Figure 76 Forest and Egger's plots for *TP53* (PIN3, rs17878362).

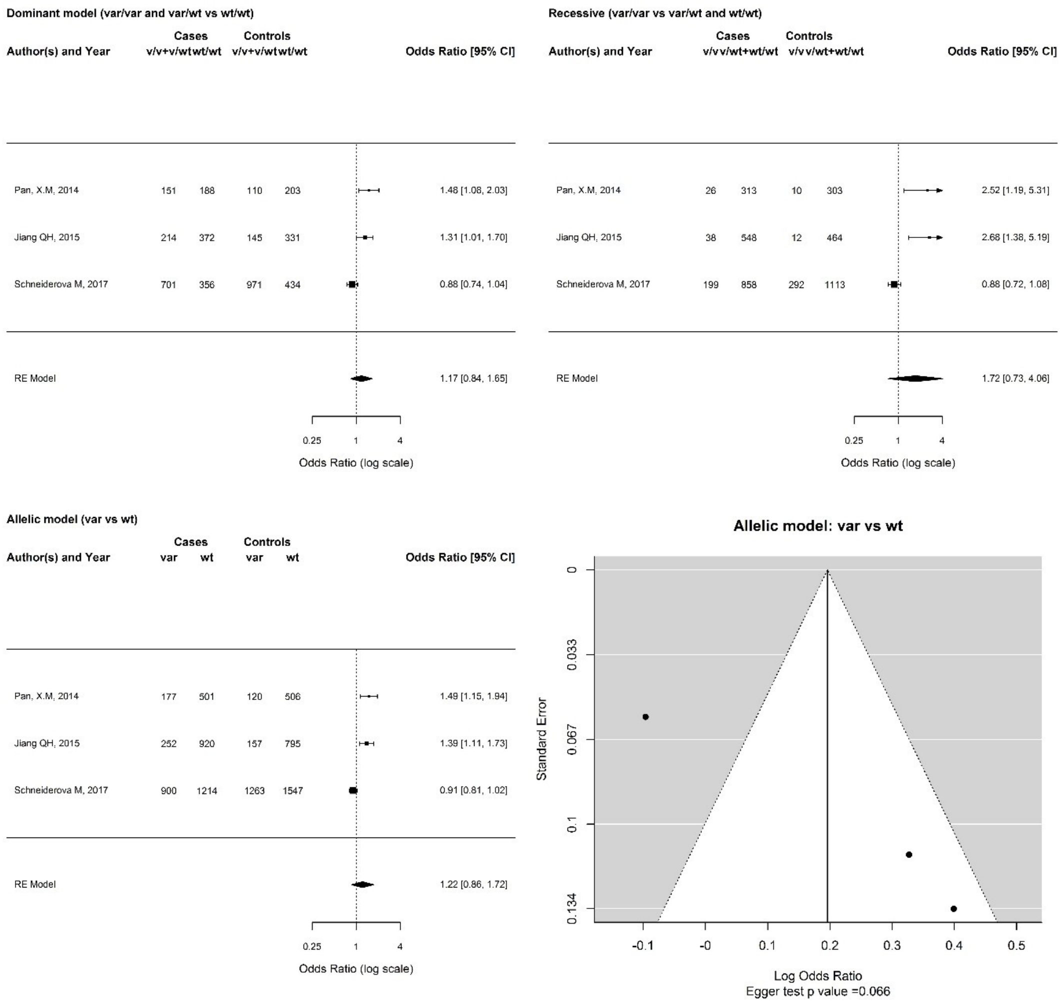

Supplementary Figure 77 Forest and Egger's plots for *KRAS* (rs712).
